# Direct estimates of cause-specific mortality fractions and rates of under-five deaths in the northern and southern regions of Nigeria by verbal autopsy interview

**DOI:** 10.1371/journal.pone.0178129

**Published:** 2017-05-31

**Authors:** Adeyinka Adewemimo, Henry D. Kalter, Jamie Perin, Alain K. Koffi, John Quinley, Robert E. Black

**Affiliations:** 1 Department of Planning, Research, and Statistics, Federal Ministry of Health, Abuja, Nigeria; 2 Institute for International Programs, Johns Hopkins Bloomberg School of Public Health, Baltimore, MD, United States of America; 3 Center for Child and Community Health Research, Department of Pediatrics, Johns Hopkins School of Medicine, Baltimore, MD, United States of America; 4 UNICEF, New York, NY, United States of America; Univesity of Iowa, UNITED STATES

## Abstract

Nigeria’s under-five mortality rate is the eighth highest in the world. Identifying the causes of under-five deaths is crucial to achieving Sustainable Development Goal 3 by 2030 and improving child survival. National and international bodies collaborated in this study to provide the first ever direct estimates of the causes of under-five mortality in Nigeria. Verbal autopsy interviews were conducted of a representative sample of 986 neonatal and 2,268 1–59 month old deaths from 2008 to 2013 identified by the 2013 Nigeria Demographic and Health Survey. Cause of death was assigned by physician coding and computerized expert algorithms arranged in a hierarchy. National and regional estimates of age distributions, mortality rates and cause proportions, and zonal- and age-specific mortality fractions and rates for leading causes of death were evaluated. More under-fives and 1–59 month olds in the South, respectively, died as neonates (N = 24.1%, S = 32.5%, p<0.001) and at younger ages (p<0.001) than in the North. The leading causes of neonatal and 1–59 month mortality, respectively, were sepsis, birth injury/asphyxia and neonatal pneumonia, and malaria, diarrhea and pneumonia. The preterm delivery (N = 1.2%, S = 3.7%, p = 0.042), pneumonia (N = 15.0%, S = 21.6%, p = 0.004) and malaria (N = 34.7%, S = 42.2%, p = 0.009) fractions were higher in the South, with pneumonia and malaria focused in the South East and South South; while the diarrhea fraction was elevated in the North (N = 24.8%, S = 13.2%, p<0.001). However, the diarrhea, pneumonia and malaria mortality rates were all higher in the North, respectively, by 222.9% (Z = -10.9, p = 0.000), 27.6% (Z = -2.3, p = 0.020) and 50.6% (Z = -5.7, p = 0.000), with the greatest excesses in older children. The findings support that there is an epidemiological transition ongoing in southern Nigeria, suggest the way forward to a similar transition in the North, and can help guide maternal, neonatal and child health programming and their regional and zonal foci within the country.

## Introduction

Globally, child mortality remains unacceptably high, with its reduction a prime target of Sustainable Development Goal (SDG) 3. In 2015, 5.9 million children around the world died before their fifth birthday, with 45% of these deaths occurring within the first month of life [1]. The under-five mortality rate in developing countries is twenty-nine times higher compared to developed countries [2]. About half of these deaths occur in just five countries: India, Nigeria, Pakistan, Democratic Republic of the Congo and China, with India accounting for more than a quarter and Nigeria about a tenth [3]. Three-quarters of these deaths are due to preventable causes that could be managed by proven cost-effective interventions [1] [4].

Despite being a signatory to the millennium declaration, with its Millennium Development Goal (MDG) of reducing under-five mortality by two-thirds by 2015, under-five mortality in Nigeria decreased by 48%, from 213 to 109 deaths per 1,000 live births, from 1990 to 2015 [3]. This compares to the MDG target of less than 67 deaths per 1,000 live births by 2015 [5]. Nigeria, with the eighth highest under-five mortality rate in the world, still ranks as the second major contributor of global under-five deaths. Although analyses of the 1990–2015 trend show that the country is making progress in reducing its under-five mortality rate, the pace remains too slow to achieve the SDG 3 target of reducing child mortality to 25 or less by 2030.

There are wide variations in the mortality rate in the six geopolitical zones of Nigeria [6] and also between the rural and urban settlements. Poverty, maternal characteristics, a weak health care system and socio-cultural barriers to care utilization have been linked to high under-five mortality rates [7] [8] [9] [10]. Studies conducted in Nigeria have examined disease prevalence and mortality patterns during the neonatal and post-neonatal periods in tertiary care hospitals [11] and found higher mortality in the northern than the southern region [12] [13] [14] [15]. The one hospital-based study reported on the causes of death but is not representative of the population since the majority of births and under-five deaths occur within the community. However, until now only the World Health Organization (WHO) [2] and Global Burden of Disease (GBD) [16] modelled estimates of the causes of neonatal and child mortality in Nigeria have been available. These estimates are derived by applying Nigeria covariates data (e.g., region, percent of children who are under weight, gross national per capita income) to input study data on causes of neonatal and child death from around the world. Hence, they may lack the desired level of country specificity.

In order to provide direct estimates of cause-specific under-five mortality, we conducted verbal/social autopsy (VASA) interviews as a follow up to deaths identified by the 2013 Nigeria Demographic and Health Survey (NDHS). Verbal autopsy of a child death is an enquiry that consists of a retrospective interview on the signs and symptoms of the fatal illness with the mother or other main caregiver of the child [17]. It is used to estimate the causes of death in a specific population with poor vital registration or medical certification of the cause of death. VA is now widely used in low and middle income countries (LMICs) to estimate cause-specific mortality, and is increasingly being used for disease surveillance and in sample registration systems [18]. It has proven to be useful in studying risk factors for specific diseases, infectious disease outbreaks, and the effects of public health interventions [19]. Most of the input data on causes of death utilized by the WHO and GBD modelled estimates for high child mortality countries in sub-Saharan Africa are from verbal autopsy studies. The VASA interviews conducted for the present study aimed to determine the cause distributions of death for neonates 0–27 days old and 1–59 month old children in the northern and southern regions of Nigeria. The findings are meant to help policy makers and development partners in the allocation of scarce resources for appropriate interventions and to the geographic areas where they are most highly needed to improve child health and reduce child morbidity and mortality.

## Methods

This was a follow up study to the 2013 NDHS, which interviewed 38,522 randomly selected households throughout the country. The lifetime birth history of all women of reproductive age was obtained to identify households with a history of one or more under-five deaths in the five years prior to the NDHS: 1,206 neonatal deaths and 2,779 child deaths were identified in the 38,522 households. The recall period was limited to five years in order to minimize the impact on the accuracy of the study findings while achieving a sufficient sample size.

### Study sample

The VASA study sought national and, if possible, regional samples of 300 neonatal (0–27 days) and 600 1–59 month old deaths, sufficient with alpha = 0.05, Z = 1.96, design effect = 1.4 and non-response rate = 0.1 to achieve precision of ±0.05 to ±0.07 in nominal 95% confidence intervals around an assumed proportion of 0.50 for the most common cause of death in each age group. Due to the possibility of overlap in the social and health system determinants of deaths within a household and to minimize the interview burden on households, the study included single deaths or randomly selected one neonatal or 1–59 month old death for interview in each household with more than one under-five deaths in the five years prior to the NDHS. Hence, a nationally representative sample of 3,254 deaths (986 neonates and 2,268 1–59 month olds) were included in the VASA study.

### Data collection instrument and methods

The data collection tool was developed by integrating the Population Health Metrics Research Consortium (PHMRC) VA questionnaire [20] with the Child Health Epidemiology Research Group (CHERG) social autopsy (SA) questionnaire [21]. The VASA instrument (S1 Appendix) has two components: the first consists of questions on characteristics of the household, while the second component includes the VA and SA questions for both neonatal and child deaths. The VA includes sections on the age of the deceased, signs and symptoms of the illness, and any available health records of the child; while the SA asks about preventive factors, caregivers’ perception of the illness that led to death, health care seeking for the illness and related constraints, quality of care provided by the health personnel at formal facilities, sociodemographic characteristics of the caregiver and social capital available to her. The tool was developed in English and translated for the interview into the three main languages of Nigeria: Yoruba, Igbo and Hausa. It was back translated by anthropologists to check for consistency and preserve meaning. Interviews were conducted using a CSProX [22] CAPI (computer-assisted personal interview) software application developed for VASA studies to assist interviewers to capture responses directly on a netbook computer with minimal data entry errors. Only the VA questions were used for the current analysis.

Forty-two interviewers and 14 supervisors who were familiar with the study area and fluent in English and at least one of the three local languages in which the interviews were conducted were recruited and underwent a 19-day training program, including 16 days of classroom work and 3 days of practice interviews. The training focused on the background to the VASA study, ethical principles for human subjects research, respondent eligibility criteria, interview methods, the data collection tools and conduct of the interview on a netbook computer.

Data collection was conducted from 1 November to 22 December, 2014. Informed consent was obtained from each respondent before the interview. The interviewers were trained to seek as the respondent the child’s main caregiver during the fatal illness, who in the case of a child illness is usually the mother. Secondary respondents were allowed if the mother was not familiar with the details of her child’s illness, for example, a neonatal death following a difficult delivery rendering the mother incapable of caring for her newborn. Any discrepancies in responses between the main and secondary respondents were resolved by accepting the main respondent’s answer.

### Analysis

The verbal autopsy data was analyzed using two methods: 1) physician-coded verbal autopsy (PCVA) and 2) computer- applied expert algorithms of illness signs and symptoms arranged in a hierarchy (EAVA). For the physician-coded verbal autopsy, one in-country pediatrician (AA) was trained in guidelines for coding of cause of death from a VA interview based on ICD-10 principles [23], and was provided with minimal diagnostic criteria required for each cause. This was to help ensure that the ascribed diagnoses were assigned consistently and in line with international standards, and to avoid diagnosis bias due to the physician’s knowledge of prevalent diseases and associated illness sign/symptom patterns in the study setting. In reviewing the VA data for each case, the pediatrician was expected to combine clinical judgement with the minimal diagnostic criteria and, using a standard death certificate, to ascribe the direct, underlying and contributing causes of death. Only the underlying causes of death were examined in the current analysis.

The expert algorithms for the neonatal and 1–59 month causes of death were based on those developed by verbal autopsy researchers for prior VA validation studies [24] [25] [26] [27] [28], further consultation and a literature review to identify illness signs and symptoms commonly associated with particular neonatal and child illnesses [29] [30] [31] [32]. Hierarchies were developed for neonatal and child diagnoses to select the EAVA primary cause of death for each child from among all possible co-morbidities identified by the algorithms. To the extent possible, the ordering of the hierarchies was based on principles incorporated in the ICD-10. The algorithms and hierarchies utilized in this study, and their development, are described in a prior publication [17].

We examined age-, sex- and cause-specific mortality fractions (CSMF = the proportion of deaths due to each cause) and age-specific rates for neonatal and child deaths in Nigeria and separately in the northern and southern regions of the country; and zonal- and age-specific mortality fractions and rates for leading causes of death. The Kappa statistic [33] was used to assess the level of agreement between the EAVA and PCVA diagnoses for each cause. All-cause mortality rates were calculated from NDHS birth histories, and age- and zonal- cause-specific rates were determined by applying VASA mortality fractions to NDHS all-cause rates. Allocation of resources and health interventions to the North and South is often based on their perceived needs related to cultural differences and higher mortality levels in the North. However, as can be seen in Table 1, there is not a strict association between mortality level and geography. Therefore, we examined any North-South differentials in causes of death both on the basis of the usual geographical division of the zones and according to two regroupings by their under-five mortality levels.

**Table 1:** Mortality rates and VASA study deaths in Nigeria’s northern and southern zones.

Geographic zone	States	2008–13 under-5 mortality*	VASA study		
			Neonatal deaths	1–59 month deaths	Total deaths
**North Nigeria**			**511**	**1616**	**2127**
North West	Jigawa, Kaduna, Kano, Katsina, Kebbi, Sokoto, Zamfara	185	309	1050	1359
North East	Adamawa, Bauchi, Borno, Gombe, Taraba, Yobe	160	107	364	471
North Central	Fct-Abuja, Benue, Kogi, Kwara, Nasarawa, Niger, Plateau	100	95	202	297
**South Nigeria**			**212**	**441**	**653**
South East	Abia, Anambra, Ebonyi, Enugu, Imo	131	56	186	242
South South	Akwa Ibom, Bayelsa, Cross River, Delta, Edo, Rivers	91	37	115	152
South West	Ekiti, Lagos, Ogun, Ondo, Osun, Oyo	90	119	140	259
**Total**		**128**	**723**	**2057**	**2780**

*_5_q_0_ reported by 2013 NDHS [5].

The data were analyzed using SAS version 9.4 for Windows [34]. Standardized sampling weights were employed to correct for non-proportional allocation of the NDHS sample to the states and urban/rural areas. The percent of “Don’t know” responses to verbal autopsy questions was examined as a quality check of the data. The Rao-Scott chi-square test was used to compare proportions of causes of death, age groups and sex, and the SAS Strata and Cluster options were used to account for the NDHS sampling design (3-stage stratification by state, urban/rural and cluster), with the sampling error computed using the Taylor linearization method of variance estimation for survey estimates. Equivalence of mortality rates computed from NDHS birth histories was tested with an observed Z statistic, with a normal approximation using the bootstrap of survey primary sampling units [35].

### Ethical clearance

Women whose NDHS birth history identified a death of an under-five years old child in the prior five years were requested to grant permission for a follow-up visit for a VASA interview. All the VASA study personnel were trained on ethical principles and practices for human subject research. Only women who gave permission for a return visit during the NDHS were approached for a VASA interview. Because illiteracy is highly prevalent among the study population, oral informed consent was sought from all study participants, who indicated their willingness to proceed with the interview by marking an ‘X’ on the consent form that included their unique study identification number. The form was then signed by the interviewer as witness to the consent. This procedure and all other aspects of the study were approved by the National Health Research Ethics Committee of the Nigeria Federal Ministry of Health and the Institutional Review Board of the Johns Hopkins Bloomberg School of Public Health.

## Results

Of the 3254 interviews attempted for the study, 185 and 125 cases, respectively, could not be visited due to a lack of security in the area of the concerned households and loss to follow-up. A total of 2,944 interviews were successfully completed, for an interview success rate of 90.5%, including 723 neonates, 2,057 children and 164 stillbirths apparently misclassified by the NDHS as neonatal deaths. The VASA questionnaire, unlike the NDHS birth history, includes several questions designed to distinguish stillbirths from neonatal deaths, so we accepted the VASA assessment of the birth status of these babies. Because a birth history is not designed to identify stillbirths, the stillbirths identified by the NDHS were not considered to constitute a representative sample and only the neonatal and child deaths were further examined by the current study. Of the total 2,780 under-five deaths, 2,126 occurred in the Northern region of the country and 654 were in the South.

The mother was the respondent for 95.5% and 94.6% of the neonatal and 1–59 month deaths, respectively; and the mean recall period from death to VA interview was 3.6 years and 3.8 years, respectively, with a range of 1 to 6 years for both age groups. There were fewer than 1% “Don’t know” responses to 65.3% and 80.0% of the 49 and 55 verbal autopsy questions asked for, respectively, neonatal and 1–59 month old deaths; and 2% or more such responses to 12.2% and 12.7% of these 49 and 55 questions, respectively, including only 4.1% (2) and 7.3% (4) questions for neonatal and 1–59 month deaths, respectively, with more than 3% “Don’t know” responses.

Table 2 shows the demographic profiles of the neonatal and 1–59 month deaths, for Nigeria as a whole and for the northern and southern regions of the country. The table reveals that neonatal deaths constituted a greater proportion of under-five mortality in the South (S) than in the North (N) (N = 511/2,127 = 24.1% vs. S = 212/653 = 32.5%, X^2^ = 12.3, p<0.001), and that both neonates (X^2^ = 1.8, p = 0.177) and 1–59 month olds (X^2^ = 63.4, p<0.001) in the South tended to die at younger ages than in the North, though not significantly so for neonates. Overall, in Nigeria as a whole, 73.9% of neonates died in the first week of life, 26.0% of under-fives died as neonates, and 61.5% of 1–59 month olds died before their second birthday. Table 3 displays age-specific mortality rates for neonates and 1–59 month olds, and demonstrates that the North-South differences in age-specific proportions of deaths were due to nearly equal neonatal mortality in the two regions but significantly higher child mortality in the North, with the age-specific North-South hazard ratios gradually increasing with older age.

**Table 2:** Demographic characteristics of deceased neonates and children in the northern and southern regions of Nigeria.

Characteristic	Northern region N (%)	Southern region N (%)	Total N (%)	X^2^, p-value*
**Neonates**				
Age (days)				
0–6	369 (72.3)	165 (77.8)	534 (73.9)	1.8, 0.177
7–27	142 (27.7)	47 (22.2)	189 (26.1)	
Sex^±^				
Male	292 (57.2)	119 (56.3)	411 (56.9)	0.0, 0.852
Female	218 (42.8)	93 (43.7)	311 (43.1)	
Total neonates	511 (70.7)	212 (29.3)	723 (100.0)	12.3, <0.001
**1–59 month olds**				
Age (months)				
1–5	228 (14.1)	99 (22.4)	327 (15.9)	63.4, <0.001
6–11	264 (16.4)	106 (24.1)	370 (18.0)	
12–23	434 (26.8)	135 (30.5)	569 (27.6)	
24–59	690 (42.7)	101 (23.0)	791 (38.5)	
Sex^±^				
Male	827 (51.2)	224 (50.8)	1051 (51.1)	0.0, 0.902
Female	788 (48.8)	217 (49.2)	1005 (48.9)	
Total 1–59 months	1616 (78.5)	441 (21.5)	2057 (100.0)	
**Total deaths**	2,127 (100.0)	653 (100.0)	2,780 (100.0)	

*All statistical tests are of North-South differences. The test of ‘Total neonates’ is for the proportion of under-five deaths that were neonatal

^±^1 with missing data in the northern region.

**Table 3:** Age-specific neonatal and child mortality rates* in the northern and southern regions of Nigeria.

Age	Northern region Estimate (95% CI)	Southern region Estimate (95% CI)	Total Estimate (95% CI)	Ratio NMR / SMR Estimate (95% CI)	Z, p-value^±^
**Neonates (days)**					
0–6	32.3 (29.9, 34.7)	30.2 (27.4, 33.1)	31.6 (29.8, 33.5)	1.1 (0.9, 1.2)	1.0, 0.307
7–27	6.5 (3.6, 9.3)	5.1 (0.7, 9.5)	6.0 (3.5, 8.5)	1.3 (0.3, 4.7)	0.4, 0.727
Total neonates^β^	38.5 (34.9, 42.1)	35.2 (30, 40.5)	37.5 (34.3, 40.5)	1.1 (0.9, 1.3)	1.0, 0.316
**1–59 month olds (months)**					
1–5	16.4 (14.2, 18.5)	13.9 (10.8, 16.9)	15.6 (13.8, 17.3)	1.2 (0.9, 1.5)	1.3, 0.204
6–11	18.5 (16.0, 21.1)	13.3 (10.2, 16.4)	16.9 (14.9, 18.9)	1.4 (1.1, 1.8)	2.4, 0.016
12–23	30.7 (24.4, 34.0)	18.2 (14.5, 21.9)	26.8 (24.1, 29.4)	1.7 (1.3, 2.1)	4.5, < 0.001
24–59	46.6 (41.5, 51.7)	15.0 (11.4, 18.7)	36.9 (32.9, 40.8)	3.1 (2.4, 4.1)	8.2, < 0.001
Total 1–59 month olds^β^	107.9 (99.7, 116.0)	59.0 (51.7, 66.4)	92.9 (86.5, 99.2)	1.8 (1.6, 2.1)	8.0, < 0.001

*All mortality rates are calculated from the NDHS birth history data for the same prior 5-year period as the VASA deaths

^**±**^Equivalence tested with an observed Z statistic, with a normal approximation using the bootstrap of survey primary sampling units [35]

^β^Total neonatal mortality rate per 1,000 live births; Total 1–59 month mortality rate, calculated as: 1-((1-U5MR)/(1-NMR)); Each age-specific rate estimated directly from the cohort that survived to that age.

Also seen in Table 2 is that, taking 50% as the expected proportion of male deaths, significantly more neonatal deaths were of males than females (56.9% vs. 43.1%, X^2^ = 10.8, p = 0.001); this was also true in the North (57.2% vs. 42.8%, X^2^ = 8.0, p = 0.005) and with the same levels in the South but statistically insignificant (56.3% vs. 43.7%, X^2^ = 2.8, p = 0.094) due to the smaller sample size. This was not true of the post-neonatal deaths, similar proportions of which were males and females, both in Nigeria and in the North and South of the country. The statistical tests displayed in Table 2 show that there were no differences between the North and South in the proportions of male and female deaths.

As seen in S2 Appendix, all the North-South differentials in the proportions of the sexes, age groups and their mortality rates held nearly constant with the zones allocated by their under-five mortality levels instead of purely on the basis of their geographic location. This was also true for the excess of male deaths among neonates within each area.

### Regional variation of causes of neonatal and 1–59 month deaths

**Fig 1:**
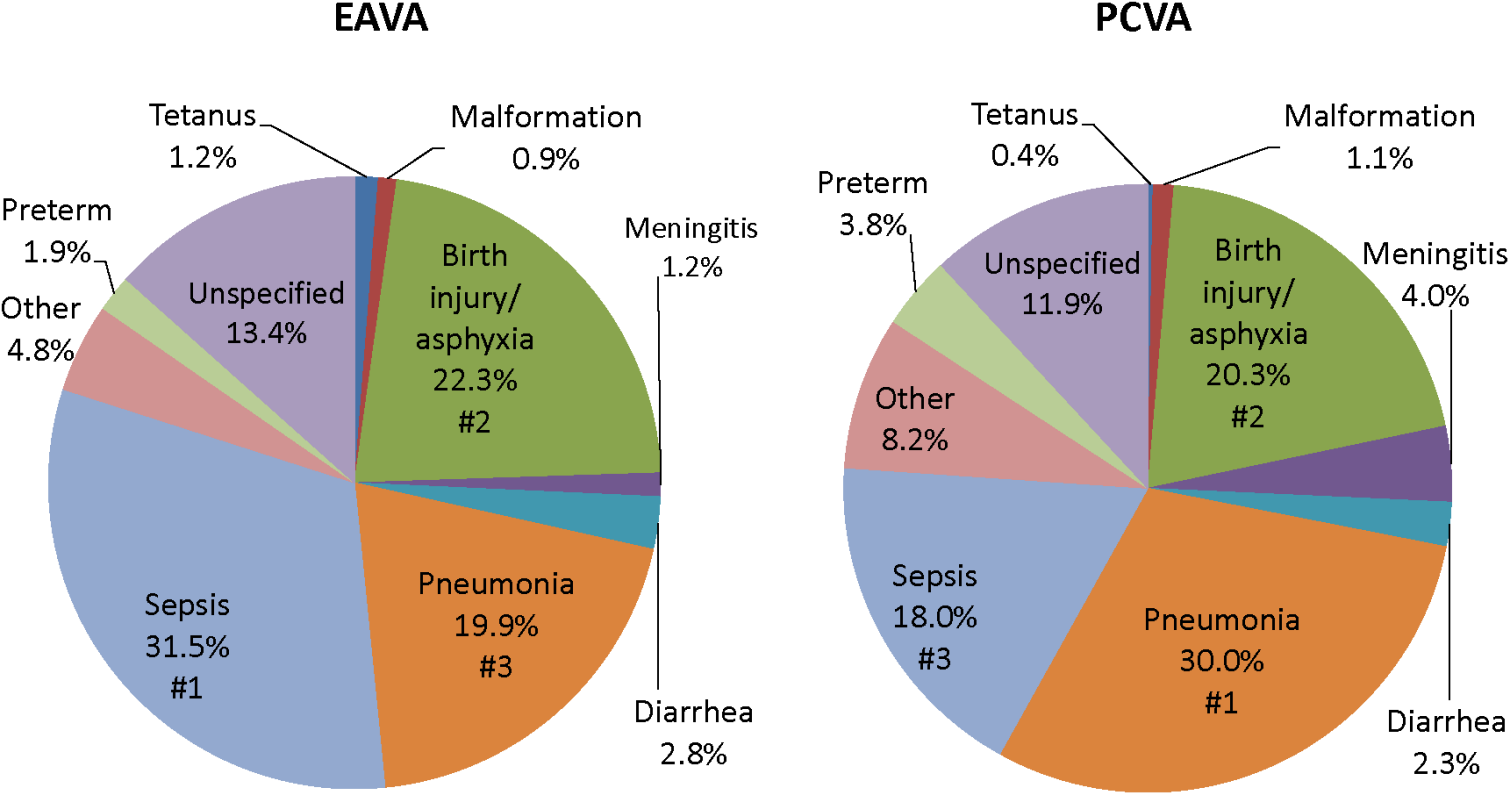
Causes of 723 neonatal deaths in Nigeria as assigned by expert algorithm and physician-coded verbal autopsy. EAVA = Expert Algorithm Verbal Autopsy, PCVA = Physician-Coded Verbal Autopsy.

Fig 1 depicts the causes of neonatal death for Nigeria as a whole, as determined by the EAVA and PCVA methods. Sepsis, birth injury/asphyxia and pneumonia were the three leading causes of death by both methods, though the order of sepsis and pneumonia was reversed. The Kappa level of agreement was 0.56, 0.85 and 0.61 for these three causes, respectively, and 0.62 or greater for three more causes (hemorrhagic fever, which is part of “other” causes), diarrhea and malformation). Kappa was less than 0.40 for preterm (0.35), meningitis (0.33), tetanus (0.24) and “other” (0.20). The physician’s “other” category included many different diagnoses, while EAVA “other” included only hemorrhagic fever, neonatal jaundice and sudden unexplained death, making the “other” category difficult to compare. Kappa for all 10 neonatal diagnoses together, including “other” and “unspecified,” was 0.55 (95% CI: 0.51, 0.59). Due to the similarity of the EAVA and PCVA results, and a prior study demonstrating the validity of the EAVA method [36], further analysis of the neonatal causes of death for this study examined only the EAVA findings.

**Fig 2:**
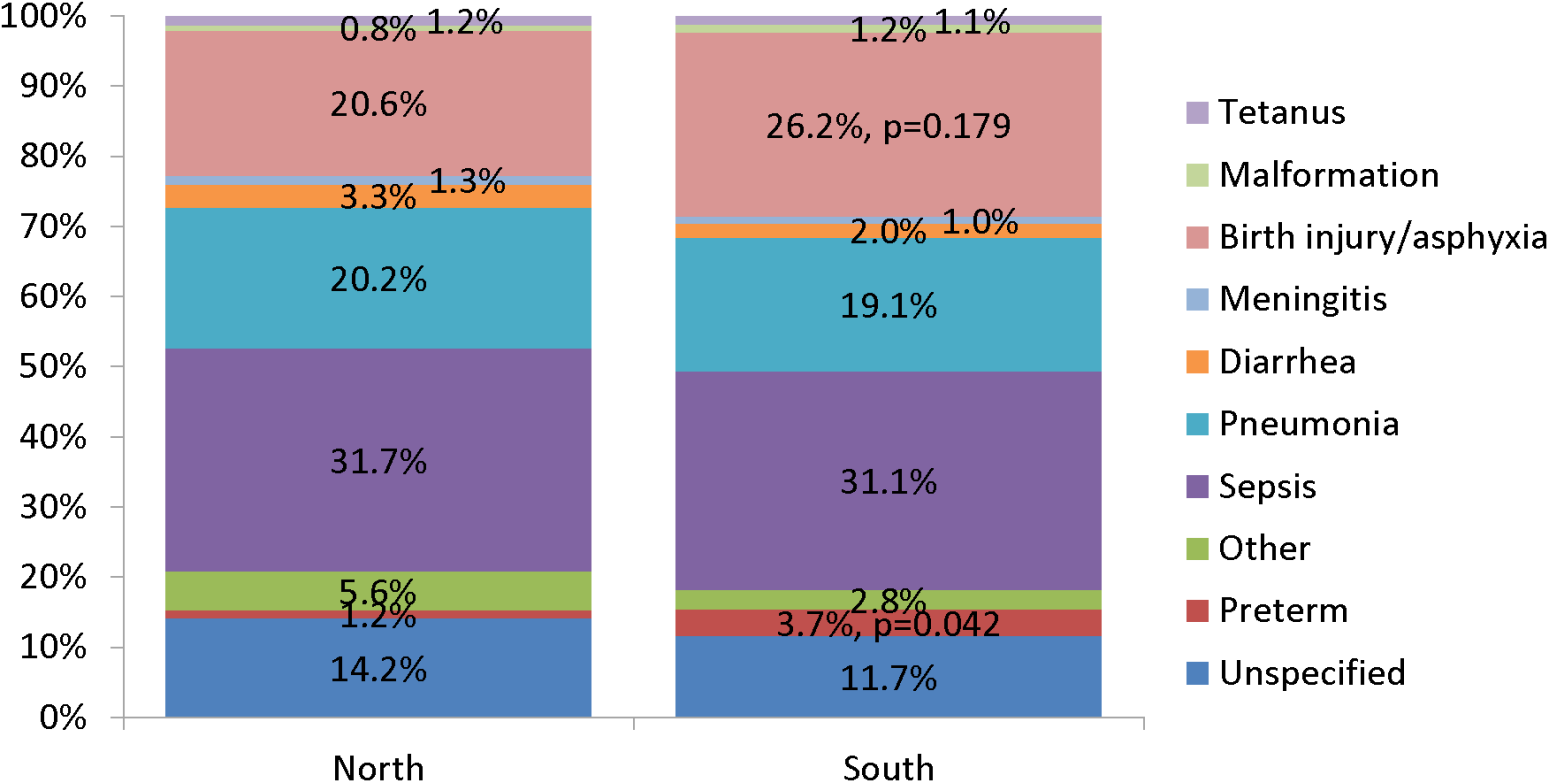
Expert algorithm causes of 511 and 212 neonatal deaths in northern and southern Nigeria.

Fig 2 illustrates the variation in cause-specific neonatal mortality in the northern and southern regions of Nigeria. Just as for Nigeria as a whole, the three leading causes of death in both regions were sepsis, pneumonia and birth injury/asphyxia. However, birth injury/asphyxia accounted for 26.2% of neonatal deaths in the South compared to 20.6% in the North (X^2^ = 1.81, p = 0.179). Similarly, the proportion of preterm deaths in the southern region was three times that of the northern region (N = 1.2% vs. S = 3.7%; X^2^ = 4.12, p = 0.042). There were no other significant differences between the two regions in their cause-specific mortality proportions, including that of early onset (days 0–1) severe neonatal infection from sepsis, meningitis or pneumonia (N = 20.6% vs. S = 22.5%, X^2^ = 0.27, p = 0.605).

**Fig 3:**
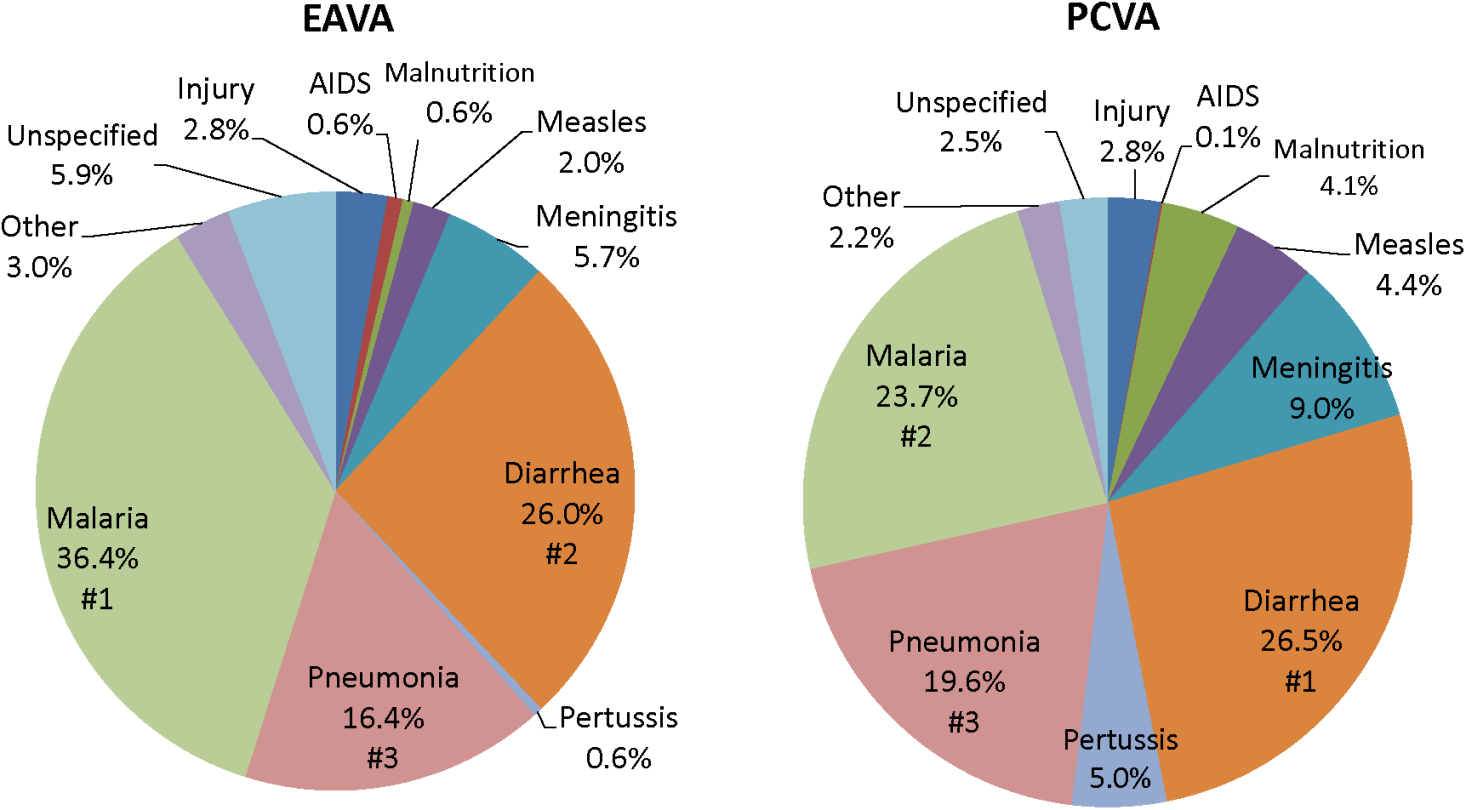
Causes of 2,057 1–59 month deaths in Nigeria as assigned by expert algorithm and physician-coded verbal autopsy. EAVA = Expert Algorithm Verbal Autopsy, PCVA = Physician-Coded Verbal Autopsy.

Fig 3 displays the causes of 1–59 month deaths for Nigeria as assigned by the EAVA and PCVA methods. The three leading causes by both methods were malaria, diarrhea and pneumonia, though the order of malaria and diarrhea was reversed for the two methods such that malaria was more common by EAVA (36.4%) than PCVA (23.7%) analysis. Despite this, there was moderate Kappa agreement between the two methods for pneumonia (0.48) and malaria (0.58); with very good agreement for diarrhea (0.73). The next leading cause of death, both by EAVA and PCVA, was meningitis; and although this diagnosis was almost twice as common by PCVA (9.0%) as EAVA (5.7%), there was moderate Kappa agreement (0.47) between the two methods. The pertussis (5.0%), measles (4.4%) and malnutrition (4.1%) proportions were the next highest by PCVA; all were much less common by EAVA, respectively 0.6%, 2.0% and 0.6%. The EAVA–PCVA Kappa agreement for measles was moderate at 0.50, but showed poor agreement for pertussis (0.11) and malnutrition (0.18). There were an equal proportion of injury deaths (2.8%) by both methods, and with excellent Kappa agreement (0.90). AIDS was uncommon by both methods, and with no agreement (Kappa = 0.00). Kappa for all 11 post-neonatal diagnoses together, including “other” and “unspecified,” was 0.55 (95% CI: 0.52, 0.57). As with the neonatal deaths, further analyses of the child deaths for this study examined only the EAVA findings.

**Fig 4:**
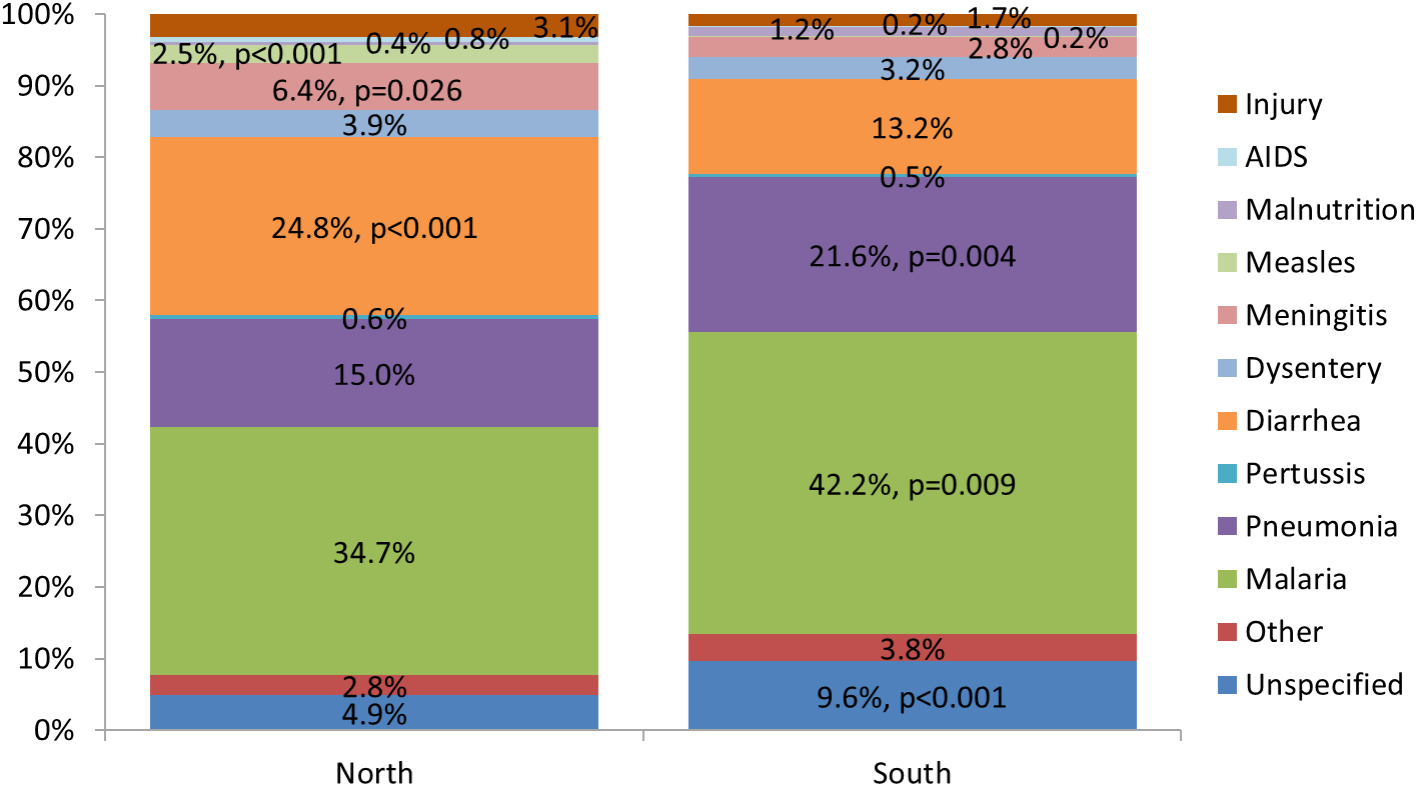
Expert algorithm causes of 1,616 and 441 1–59 month deaths in northern and southern Nigeria.

Fig 4 illustrates the causes of 1–59 month death in the northern and southern regions of Nigeria. As for Nigeria as a whole, the three leading causes of death in both regions were malaria, diarrhea/dysentery and pneumonia. Approximately, four-fifths of all children, 78.5% in the North and 80.1% in the South, died from one of these causes. However, diarrhea was more common in the North, with all of the differential due to watery diarrhea (N = 24.8% vs. S = 13.2%, X^2^ = 21.6, p<0.001), while malaria (N = 34.7% vs. S = 42.2%, X^2^ = 6.8, p = 0.009) and pneumonia (N = 15.0% vs. S = 21.6%, X^2^ = 8.2, p = 0.004) predominated in the South. As with the country as a whole, meningitis, injury, measles and malnutrition were the next most common causes of death in both regions, but the first three were proportionally about twice as common in the North as in the South and the last was three times as common in the South. The difference was statistically significant for meningitis (N = 6.4% vs. S = 2.8%, X^2^ = 4.9, p = 0.026) and measles (N = 2.6% vs. S = 0.2%, X^2^ = 14.5, p<0.001) and nearly so for injury (N = 3.1% vs. S = 1.7%, X^2^ = 2.9, p = 0.090) and malnutrition (N = 0.4% vs. S = 1.2%, X^2^ = 2.6, p = 0.109). Together, these four causes contributed 12.6% of the deaths in the North and 5.8% in the South. There were no significant differences between the regions in any other cause-specific mortality proportions.

S3 Appendix displays details of the mortality differentials for all the EAVA neonatal and 1–59 month old causes, all by the pure North-South geopolitical assignment of the six zones, as well as the two methods of reassigning the zones based on their under-five mortality rates. The only changes with reallocation of the zones were aligning of the neonatal birth injury/asphyxia and 1–59 month old pneumonia levels in one of the two scenarios, and reversal of the area-specific levels of 1–59 month old injury deaths in one scenario and equalization in the other.

### Zonal- and age-specific causes of neonatal and 1–59 month deaths

Table 4 presents the CSMFs and cause-specific mortality rates per 1000 live births for the most common causes of neonatal and 1–59 month deaths in the six geopolitical zones of Nigeria. The table shows that the southern region’s excess in birth injury/asphyxia deaths was concentrated in the South East and South West zones, with the country’s lowest birth injury/asphyxia-specific CSMF and neonatal mortality rate (NNMR) in the South South. In general, the CSMFs and NNMRs for birth injury/asphyxia conveyed a similar picture across the six zones. This was different for neonatal pneumonia/sepsis, for which the table demonstrates that although the pneumonia/sepsis CSMFs of the northern and southern regions were similar, due to the North’s slightly higher all-cause NNMR the pneumonia/sepsis-specific mortality rate was also somewhat higher in the North than in the South.

**Table 4:** Birth injury/asphyxia-, neonatal pneumonia/sepsis-, diarrhea-, pneumonia- and malaria-specific mortality fractions and rates* in Nigeria’s six geographic zones.

Zone	VASA neonatal deaths	All-cause NNMR^±^	Birth injury/asphyxia		Pneumonia/Sepsis		VASA 1–59 month old deaths	All-cause 159MR^£^	Diarrhea		Pneumonia		Malaria	
			CSMF^β^ (%) 95% CLs	NNMR^±^ 95% CLs	CSMF^β^ (%) 95% CLs	NNMR^±^ 95% CLs			CSMF^β^ (%) 95% CLs	159MR^£^ 95% CLs	CSMF^β^ (%) 95% CLs	159MR^£^ 95% CLs	CSMF^β^ (%) 95% CLs	159MR^£^ 95% CLs
North West	309	40.6	21.4 15.6, 27.2	8.8 6.4, 11.2	52.7 46.0, 59.3	21.6 18.9, 24.3	1050	125.9	26.3 23.1, 29.5	33.1 29.1, 37.2	12.9 10.6, 15.2	16.3 13.4, 19.2	33.4 29.9, 36.9	42.1 37.7, 46.5
North East	107	37.4	17.0 9.8, 24.3	6.4 3.7, 9.1	46.5 32.9, 60.0	17.4 12.3, 22.4	364	108.2	25.3 19.8, 30.8	27.3 21.4, 33.3	20.0 15.3, 24.6	21.6 16.5, 26.6	36.2 29.9, 42.4	39.1 32.3, 45.8
North Central	95	34.3	22.0 11.7, 32.3	7.5 4.0, 11.0	55.4 44.0, 66.8	19.4 6.8, 23.4	202	57.6	16.3 11.3, 21.3	9.5 6.6, 12.4	17.2 10.0, 24.3	10.0 5.8, 14.1	39.2 31.8, 46.6	22.7 18.4, 27.0
South East	56	36.8	35.1 19.8, 50.3	13.0 7.3, 18.6	50.4 36.4, 64.4	18.6 13.5, 23.8	186	87.3	9.2 5.2, 13.2	8.0 4.5, 11.5	26.4 19.4, 33.4	23.0 16.9, 29.1	44.8 37.9, 51.8	39.0 33.0, 45.1
South South	37	30.2	7.5 0.9, 14.1	2.3 0.3, 4.2	53.7 40.2, 67.3	17.2 12.9, 21.5	115	53.3	8.2 3.2, 13.3	4.3 1.7, 7.0	15.4 8.7, 22.2	8.2 4.6, 11.8	53.3 44.3, 62.3	28.2 23.5, 33.0
South West	119	37.7	28.0 17.5, 38.5	10.6 6.7, 14.6	49.0 38.8, 59.3	19.1 15.1, 23.1	140	44.6	22.4 14.1, 30.7	10.1 6.3, 13.8	20.4 11.9, 28.8	9.2 5.4, 13.0	29.5 20.0, 39.1	13.3 9.0, 17.6
**Total**	723	37.5					2057	92.9						

*All mortality rates are calculated from the NDHS birth history data for the same prior 5-year period as the VASA deaths

^±^Neonatal deaths per 1000 live births

^β^VASA cause-specific mortality fraction for the prior 5-year period

^£^1–59 month mortality rate, calculated as: 1-((1-U5MR)/(1-NMR)).

Regarding the 1–59 month olds, diarrhea constituted a lower proportion of all deaths in the southern region except in the South West, where the diarrhea CSMF was more like that of the North. Nevertheless, even with similar diarrhea-specific mortality fractions, due to their higher overall 1–59 month mortality rates (159MRs), diarrhea-specific mortality was about three times higher in the North East and North West than in the South West and up to seven times higher than in the other southern zones. Similarly, while the South West’s pneumonia fraction was equal to or higher than the highest mortality northern zones, its pneumonia-specific mortality rate was about half that of the North East and North West due to its lower overall mortality. The South East’s high pneumonia fraction, which led the South overall to outweigh the North in that measure, also brought its pneumonia mortality in line with that of the worst off North East and North West zones despite the South East’s lower all-cause 159MR. The picture for malaria was similar, with the highest mortality fractions in the South East and South South, but approximately equal malaria-specific mortality in the North East, North West and South East.

Table 5 provides the age-cause-specific mortality fractions and rates for the leading causes of neonatal and 1–59 month old deaths and preterm delivery deaths in the north and south of Nigeria. The age-specific patterns of the neonatal causes follow expectations, with preterm delivery and birth injury/asphyxia predominating in the 0–6 day group (early neonates), and the fraction of pneumonia/sepsis deaths being greater in late neonates but the mortality rate higher in early neonates, with little difference between the regions in any of these measures. While the fraction of preterm delivery deaths was greater in the South than the North (N = 1.2%, S = 3.7%, p = 0.042), there was no significant difference in their mortality rates (Z = 1.44, p = 0.150). On the other hand, there were vast differences between the North and the South in their 1–59 month age-cause-specific mortality patterns. The diarrhea-, pneumonia- and malaria-specific 1–5 month mortality rates in the South, respectively, were 32.0%, 13.2% and 17.2% lower than in the North. However, in the North, diarrhea and malaria mortality of 24–59 month olds, respectively, were 420.0% and 176.6% greater than that of 1–5 month olds; while in the South they were just 58.8% and 22.6% higher in 24–59 month olds. Pneumonia mortality of 24–59 month olds in the North was 36.8% higher than that of 1–5 month olds, but in the South was 3.0% lower in the older age group. The result was that by the age of 24–59 months, diarrhea, pneumonia and malaria mortality in the South, respectively, were 79.2%, 38.5% and 63.3% lower than in the North, or an increase of 47.2%, 25.3% and 46.1% over the South-North mortality gap in 1–5 month olds. The cumulative effect of these differences was that the total diarrhea, pneumonia and malaria mortality rates in the North, respectively, were 222.9% (Z = -10.9, p = 0.000), 27.6% (Z = -2.3, p = 0.020) and 50.6% (Z = -5.7, p = 0.000) higher than in the South.

**Table 5:** Age-cause-specific neonatal and child mortality rates* in the northern and southern regions of Nigeria.

Age group	North							South						
Neonates (days)	All-cause NNMR^±^	Preterm delivery		Birth injury/asphyxia		Pneumonia/sepsis			Preterm delivery		Birth injury/asphyxia		Pneumonia/sepsis	
		CSMF^β^ (%) 95% CLs	CSMR^£^ 95% CLs	CSMF^β^ (%) 95% CLs	CSMR^£^ 95% CLs	CSMF^β^ (%) 95% CLs	CSMR^£^ 95% CLs	All-cause NNMR^±^	CSMF^β^ (%) 95% CLs	CSMR^£^ 95% CLs	CSMF^β^ (%) 95% CLs	CSMR^£^ 95% CLs	CSMF^β^ (%) 95% CLs	CSMR^£^ 95% CLs
0–6	32.3 29.9, 34.7	1.6 0.4, 2.9	0.5 0.1, 0.9	27.2 21.4, 32.9	8.8 6.9, 10.6	45.9 39.7, 52.2	14.8 12.8, 16.9	30.2 27.4, 33.1	4.8 0.6, 9.0	1.5 0.2, 2.7	31.4 22.4, 40.3	9.5 6.6, 12.2	45.8 37.0, 54.6	13.8 11.2, 16.5
7–27	6.5 3.6, 9.3	0.0 0.0, 0.0	0.0 0.0, 0.0	3.6 0.4, 6.8	0.2 0.0, 0.4	67.4 57.8, 76.9	4.4 3.8, 5.0	5.1 0.7, 9.5	0.0 0.0, 0.0	0.0 0.0, 0.0	8.2 0.0, 16.4	0.4 0.0, 0.8	65.6 51.9, 79.3	3.3 2.6, 4.0
Total	38.5 34.9, 42.1	1.2 0.3, 2.1	0.5 0.1, 0.8	20.6 16.3, 24.9	7.9 6.3, 9.6	51.9 46.5, 57.2	20.0 17.9, 22.0	35.2 30._, 40.5	3.7 0.5, 7.0	1.3 0.2, 2.5	26.2 18.9, 33.6	9.2 6.6, 11.8	50.2 43.0, 57.5	17.7 15.1, 20.2
**1–59 month olds** (months)	**All-cause 159MR**^€^	**Diarrhea**		**Pneumonia**		**Malaria**		**All-cause 159MR**^€^	**Diarrhea**		**Pneumonia**		**Malaria**	
1–5	16.4 14.2, 18.5	15.5 9.9, 21.2	2.5 1.6, 3.5	23.3 17.2, 29.5	3.8 2.8, 4.8	38.9 31.9, 45.9	6.4 5.2, 7.5	13.9 10.8, 16.9	12.2 6.5, 17.9	1.7 0.9, 2.5	23.7 14.2, 33.1	3.3 2.0, 4.6	38.2 28.0, 48.3	5.3 3.9, 6.7
6–11	18.5 16.0, 21.1	29.3 22.5, 36.0	5.4 4.2, 6.7	14.8 10.8, 18.8	2.7 2.0, 3.5	32.6 26.9, 38.3	6.0 5.0, 7.1	13.3 10.2, 16.4	22.8 14.1, 31.5	3.0 1.9, 4.2	21.8 11.5, 32.1	2.9 1.5, 4.3	37.2 27.6, 46.7	4.9 3.7, 6.2
12–23	30.7 24.4, 34.0	36.7 31.1, 42.4	11.3 9.6, 13.0	16.8 12.9, 20.7	5.2 4.0, 6.9	28.6 23.9, 33.4	8.8 7.3, 10.3	18.2 14.5, 21.9	13.1 6.1, 20.0	2.4 1.1, 3.6	19.9 12.5, 27.3	3.6 2.3, 5.0	48.0 37.2, 58.8	8.7 6.8, 10.7
24–59	46.6 41.5, 51.7	27.8 23.8, 31.8	13.0 11.1, 14.8	11.2 8.6, 13.8	5.2 4.0, 6.4	38.0 33.5, 42.5	17.7 15.6, 19.8	15.0 11.4, 18.7	17.9 11.8, 24.0	2.7 1.8, 3.6	21.6 12.1, 31.2	3.2 1.8, 4.7	43.6 33.8, 53.5	6.5 5.1., 8.0
Total	107.9 99.7, 116.0	28.7 26.0, 31.4	31.0 28.0, 33.9	15.0 12.9, 17.1	16.2 14.0, 18.4	34.7 31.9, 37.5	37.5 34.4, 40.5	59.0 51.7, 66.4	16.3 12.3, 20.3	9.6 7.3, 12.0	21.6 17.2, 26.0	12.7 10.1, 15.4	42.2 37.2, 47.2	24.9 21.9, 27.9

*All mortality rates are calculated from the NDHS birth history data for the same prior 5-year period as the VASA deaths

^±^Total neonatal mortality rate per 1000 live births; Each age-specific rate estimated directly from the cohort that survived to the start of that age group

^β^VASA cause-specific mortality fraction for the prior 5-year period

^£^Cause-specific mortality rate

^€^Total 1–59 month mortality rate, calculated as: 1-((1-U5MR)/(1-NMR)); Each age-specific rate estimated directly from the cohort that survived to the start of that age group.

## Discussion

In Nigeria, the national civil registration system was introduced in 1979 through the Births and Deaths Compulsory Registration decree 39, and was modified in 1992 when the National Population Commission (NPC) was given the mandate to operate the system [37]. Currently, there are 2773 functional registration centers across the country operating under a passive hierarchical model. The system faces numerous challenges, including low level coverage and public awareness, inadequate funding, heavy bureaucracy and poor data management. An integrated National Health Management Information System (NHMIS) was developed in 1993 to improve data capture, analysis and report generation. To meet the resource and infrastructure needs required to strengthen the NHMIS, in 2013 the National Council on Health resolved to implement an integrated, decentralized national routine health database hosted at the Federal Ministry of Health (FMOH) and harmonized NHMIS tools for routine data collection and reporting by all programs and implementing partners [38]. The service statistics capture births, deaths of under-fives and causes of death. However, most community births and deaths, and all vital events that occur in private health facilities, are missed.

As an interim measure, the VASA study was conducted as a collaborative effort of the FMOH, NPC, the National Bureau of Statistics, and the Johns Hopkins University, with the purpose of providing direct estimates of the causes and determinants of neonatal and 1–59 month mortality in Nigeria to improve global, regional and country estimates, and for planning of appropriate interventions, national initiatives and strategies toward improving child survival.

The study found that, overall, one fourth of under-five deaths occurred during the neonatal period, but with a significantly higher proportion of neonatal deaths in the South than in the North; and that, while nearly three quarters of neonatal deaths occurred in the first week of life, again there was a trend for more neonatal deaths in the South to occur during the early neonatal period. These findings, while consistent with the 2013 NDHS report on the geographical variation of under-five mortality [5], add detail on the early neonatal to total neonatal ratio and on the statistical significance of the findings.

The three leading causes of neonatal death, both by EAVA and PCVA analysis, were sepsis, birth asphyxia and pneumonia. The percent of neonatal deaths from early onset severe infection (sepsis, meningitis or pneumonia) closely mirrored that of prior global estimates that 30% to 40% of neonatal sepsis deaths are due to infection vertically transmitted during childbirth and with early onset of symptoms [39] [40]. In addition, the VASA identified more neonatal deaths from preterm delivery and a trend toward more birth injury/asphyxia deaths in the south than in the north of Nigeria; but no North-South differential in the proportion of deaths from early onset severe neonatal infection. The three leading causes of 1–59 month deaths, both by EAVA and PCVA analysis, were malaria, diarrhea and pneumonia; with diarrhea (and the less frequent causes, measles and meningitis) predominating in the North, and pneumonia and malaria in the South. These North-South differentials in the proportions of deaths from various causes, both for neonates and 1–59 month olds, were similar whether the areas were defined strictly according to geography, or also taking into account the zonal under-five mortality levels, with the exception of the neonatal birth injury/asphyxia and 1–59 month old pneumonia levels being equalized between the areas in one of the two mortality scenarios, and the area-specific levels of 1–59 month old injury deaths being reversed in one scenario and equalized in the other (S3 Appendix).

These findings suggest that the south of Nigeria is in the early phase of an epidemiological transition, with a larger fraction of neonatal deaths and both neonates and 1–59 month olds dying at younger ages in the South than in the North, and more perinatal causes of death in the South and post-neonatal infectious causes in the North. One apparent anomaly to this cause of death pattern was the high pneumonia and malaria mortality in the South East, with its intermediate level 159MR driving a mortality distribution in some ways more similar to the North than the South. This higher than expected child mortality in the South East, despite its superior levels for several sociodemographic indicators, has been previously noted, but without adequate understanding or explanation [8] [9] [10]. The lack of a North-South differential in the proportion of neonatal deaths from early onset severe infection is also of interest. While one could theorize this would predominate in the South, preventing these deaths would require both antenatal and intrapartum infection control in mothers and immediately postnatal in newborns. Therefore, the contribution of deaths from early onset neonatal infection to an epidemiological transition might be expected to be less clear. This might be further explored by a thorough analysis of available (modelled) country-level data [41].

An epidemiological transition in the neonatal to 1–59 months deaths ratio and accompanying changes in the cause structure of mortality as a country develops is a well-known phenomenon. This occurs as the more easily tackled post-neonatal infectious causes of death are gradually overcome and perinatal causes due to maternal, fetal and neonatal complications that are more difficult to surmount become relatively more common [3]. An increasing ratio of neonatal to 1–59 months deaths in Nigeria from 1999–2013 is evident from available data [5]. However, we are not aware of any study that has examined the predominance of this indicator nor the increased ratio of early to late neonatal deaths in southern Nigeria as found by the current study. Protective (antenatal care and institutional delivery) and risk (short birth interval and small birth size) factors for neonatal mortality in Nigeria have been previously identified [9]. We plan to examine these and other possible reasons for the continued high level of neonatal deaths in Nigeria with a propensity scores analysis of mortality determinants; and have begun with an assessment of reasons for the increased levels of pneumonia, malaria and diarrhea deaths of 1–59 month olds in different regions of the country in a companion social autopsy study [42] to the current verbal autopsy analysis.

Cause-specific mortality fractions convey important public health information on the leading causes of death that is helpful in prioritizing the allocation of scarce resources. However, cause-specific mortality rates in combination with CSMFs can provide a more complete picture when comparing areas within a country with different all-cause mortality rates. The current study found that the mortality fractions and rates for birth asphyxia provided a similar view across the six geographic zones of Nigeria, with both metrics determining that this cause was most highly concentrated in the South East and South West. On the other hand, examining the CSMFs and mortality rates together for neonatal sepsis/pneumonia and 1–59 month diarrhea, pneumonia and malaria better identified which zones were most adversely affected by these causes than the CSMFs alone. These analyses revealed that neonatal pneumonia/sepsis mortality was somewhat more prominent in the North than in the South; that diarrhea mortality was three to seven times higher in the North West and North East than in the southern zones; and that pneumonia and malaria mortality in the South East were on a par with the worst affected of the northern zones, with the South South not far behind for malaria.

Examining age-cause-specific mortality rates and fractions can further focus public health efforts by identifying priority age groups within highly-affected regions and zones. As expected, preterm delivery and birth injury/asphyxia mortality occurred entirely or mainly in early neonates. And due to the concentration of neonatal deaths in days 0–6, pneumonia/sepsis mortality also was greater in early neonates, but as seen by its 7–27 day mortality fraction, pneumonia/sepsis also was the most common cause of late neonatal deaths. This mortality pattern, which mirrors the global picture [41], was similar in the North and the South, so can guide public health efforts throughout the country. The age-cause-specific data for 1–59 month olds revealed that, with the exception of pneumonia in 6–11 month olds, diarrhea, pneumonia and malaria mortality in all age groups were higher in the North than in the South. Also, diarrhea and malaria mortality of children one year and older in the North were considerably higher than that of infants, while the age-related elevation in pneumonia mortality was moderated; compared to which in the South diarrhea and pneumonia mortality held constant across the age groups and malaria in the oldest children was somewhat greater than in infants. These findings suggest that disease control efforts against diarrhea and malaria need to be dramatically boosted in the North, especially in older children, while in the South the focus should be on malaria control. The data also suggest that the epidemiological transition in the South has been strongly fueled by age-specific declines in diarrhea, pneumonia and, to a lesser extent, malaria; and that effective disease control efforts in the North could help propel a similar transition.

There were far fewer neonatal deaths from preterm delivery in the current study than estimated for Nigeria by the WHO and GBD causes-of-death models [2,16]. We previously conducted a VASA study in Niger, which identified a similarly low level of neonatal deaths caused by preterm delivery [17]. Possible reasons are discussed in detail in that paper, which, in brief, include: 1) our placement of preterm delivery without a specific condition attributable to prematurity at the bottom of the EAVA hierarchy, whereas the models accept the published causes of death in their VA study input data, which are usually PCVA diagnoses and without mention of any diagnostic criteria provided to the study physicians; 2) acceptance by the models of less rigorous case definitions, without documentation of pregnancy duration or birth weight; and 3) falsely low reporting of short pregnancy duration by the VASA study respondents. Only 9.1% of respondents in the current study reported that their pregnancy duration was seven or fewer months, and another 3.9% said it was eight months. Therefore, the maximum proportion of neonatal deaths caused by preterm delivery that could be identified by the Nigeria VASA study, if preterm were placed at the top of the EAVA hierarchy, would be 13.0%. This contrasts with the WHO and GBD models’ 2015 estimates for Nigeria of, respectively, 31.9% and 16.7% of neonatal deaths due to preterm delivery. One action that could be taken to help examine this discrepancy would be to conduct studies of the validity of retrospectively-reported pregnancy duration. This is important not only to determine the validity of the VA diagnosis of preterm delivery as a cause of neonatal death, but also to assess the accuracy of similar data collected by the pregnancy calendar of many demographic surveys.

Aside from the discrepancy in the proportion of neonatal deaths due to preterm delivery, the VASA study and the WHO and GBD models all identified sepsis, pneumonia and birth asphyxia as the main causes of neonatal death in Nigeria; and all determined that diarrhea, pneumonia and malaria were the leading causes of 1–59 month mortality. Although the three studies’ estimates for all these causes differed, overall there was more agreement between the VASA and GBD estimates for the causes. The VASA study and WHO and GBD modelled estimates for neonatal sepsis, pneumonia and birth asphyxia, respectively, were 31.5%, 16.0% and 31.7%, 19.9%, 7.3% and 6.5%, and 22.3%, 30.2% and 23.6%; and for diarrhea, pneumonia and malaria of 1–59 month olds were, respectively, 26.0%, 14.7% and 22,7%, 16.4%, 22.8% and 10.5%, and 36.4%, 20.1% and 37.2%. Clearly, the studies’ respective estimates for the neonatal causes of death were skewed by the large variation in their assessments of preterm delivery. The reason for the disparity in their estimates for the 1–59 month causes is less clear, but might be partly due to some of the contrasting diagnostic methods used by the studies that are discussed above, as well as the WHO and GBD’s different approaches to model building.

The main limitation of this study is that, due to the long recall period from the events that led to death of the deceased to the time of interview, there is the possibility that respondents may not have been able to accurately recall the signs and symptoms of the illness that led to their child’s death. However, this is mitigated by the fact that the data collectors were well-trained on how to elicit information from the respondents on the events that led to the death. The low percentage of interviews with a “Don’t know” response to verbal autopsy questions provides additional reassurance of the quality of the data.

In conclusion, the VASA study provided the first ever direct estimates at the national, regional and zonal levels of the causes of neonatal and 1–59 month mortality in Nigeria. The data can serve on their own to help guide the improvement of maternal, neonatal and child health policies and intervention programs, or might supplement the WHO and GBD modelled estimates. The age at death and cause of death findings suggest that the southern region of Nigeria is in the early phase of an epidemiological transition and suggest the way forward for the North to undergo a similar transition. This information, together with the study’s estimates of the individual causes of death in the northern and southern regions and in the regions’ six zones, including cause-specific mortality fractions and rates, renders the data more useful in its support of area-specific public health decisions than would be solely national-level data.

## Supporting information

S1 Appendix The VASA questionnaire in English, Hausa, Igbo and Yoruba. (DOCX)

S2 Appendix Age and sex distributions and mortality rates of neonatal and 1–59 month old deaths in Nigeria with the geographic zones re-distributed by under-five mortality levels. (DOCX)

S3 Appendix Expert algorithm verbal autopsy causes of neonatal and 1–59 month old deaths in the North and South of Nigeria and with the geographic zones re-distributed by under-five mortality levels. (DOCX)
