# Supplementary material for: Direct estimates of cause-specific mortality fractions and rates of under-five deaths in the northern and southern regions of Nigeria by verbal autopsy interview
Source: PLoS One. 2017 May 31;12(5):e0178129. doi: 10.1371/journal.pone.0178129 (PMC5451023; doi:10.1371/journal.pone.0178129)
Supplement: S1 Appendix — (DOCX) [file pone.0178129.s001.docx]

**Appendix 1: the VASA questionnaire in English, Hausa, Igbo and Yoruba**

**English VASA questionnaire**

| **VERBAL/SOCIAL AUTOPSY GENERAL INFORMATION (FOR SBs, NN & CHILD DEATHS 0—59 MONTHS OLD)**  **Section 1: Background about the deceased**  *Interviewer: Before going to the field to do the interview, fill in this section from the survey or surveillance record for the deceased*. | | | | | | | |
| --- | --- | --- | --- | --- | --- | --- | --- |
| G1.1 | Address of the household  *[Copy the household address]* | | State ____________________________  LGA _____________________________  Locality __________________________  EA ______________________________ | | | 🞎🞎🞎  🞎🞎🞎  🞎🞎🞎🞎  🞎🞎🞎🞎 | |
|  | Directions to the household  *[Copy the directions to the household]* | |  | | | | |
| G1.2 | Name of the deceased (if known)  *[Copy the name of the deceased]* | |  | | | | |
| G1.3 | Sex of deceased  *[Copy the sex of the deceased]* | | 1. Male 2. Female | | | 🞎 | |
| G1.4 | Date of birth of the deceased  *[Copy the day, month and year of birth of the deceased]* | | | | | **__ __/__ __/__ __ __ __**  D D M M Y Y Y Y  *(DK = 99/99/9999)* | |
| G1.5 | Date of death of the deceased  *[Copy the day, month and year of death of the deceased]* | | | | | **__ __/__ __/__ __ __ __**  *(DK = 99/99/9999)* | |
| G1.6 | Last known age of the deceased  *[Copy the last known age of the deceased: Record days if less than 28 days—if less than 24 hours, record “00” days; Record months if 28 days-11 months; Record years if 1 year or older.]* | | | | | **__ __** Days: ***1 or more → GQ1.7***  *(DK = 99)* | |
|  |  |  |  |  |  | **__ __** Months ***→ GQ1.7***  *(DK = 99)* | |
|  |  |  |  |  |  | **__ __** Years ***→ GQ1.7***  *(DK = 99)* | |
| G1.6.1 | Was this a stillbirth or neonatal death?  *[Copy this information from the record]* | 1. Stillbirth 2. Neonatal death   9. Not known from the record | | | | 🞎 | |
| G1.7 | Name of mother  *[Copy the name of the mother]* |  | | | | | |
| G1.8 | Name of father  *[Copy the name of the father]* |  | | | | | |
| **Section 2: Background about the interview**  *Interviewer: Before and after the interview, fill in this section. These questions should not be asked of the respondent.* | | | | | | | |
| G2.1 | Language of the interview |  | | | | | |
| G2.2 | Interviewer name and ID number | _________________________________ | | | | 🞎🞎🞎🞎🞎 | |
| G2.3 | Dates of attempted and successful interviews | **DATE** | | | | **RESULT OF THE INTERVIEW** | |
| G2.3.1 | Date of first interview attempt | **__ __/__ __/__ __ __ __**  D D M M Y Y Y Y | | | | **Interim result:**  🞎 | |
| G2.3.2 | Date of second interview attempt | **__ __/__ __/__ __ __ __**  D D M M Y Y Y Y | | | | **Interim result:**  🞎 | |
| G2.3.3 | Date of third interview attempt | **__ __/__ __/__ __ __ __**  D D M M Y Y Y Y | | | | **Interim result:**  🞎 | |
| G2.4 | Date interview started  *[Equals date of the last attempt]* | **__ __/__ __/__ __ __ __**  D D M M Y Y Y Y | | | |  | |
| G2.5 | Time interview started  *[Record hour 1-24 / minutes 1-60]* | **__ __/__ __**  H R M M | | | |  | |
| G2.6 | Date interview finished  *[Equals date started or a later date]* | **__ __/__ __/__ __ __ __**  D D M M Y Y Y Y | | | | **Final result:**  🞎 | |
| G2.7 | Time interview finished  *[Record hour 1-24 / minutes 1-60]* | **__ __/__ __**  H R M M | | | |  | |
|  | **Interview result codes:**  1. Completed (Final result code)  2. Partially completed (Final result code)  3. Eligible respondent postponed interview  4. No eligible respondent at home at time of visit  5. Eligible respondent refused interview | | | 6. No eligible respondent lives in household  7. No household member at home  8. Dwelling vacant / destroyed / not found  9. In progress (Interim result code)  10. Child reported dead in birth history is actually alive  11. Duplicate report of death – interview already conducted | | | |
| G2.8 | Date form checked by supervisor | **__ __/__ __/__ __ __ __**  D D M M Y Y Y Y | | | |  | |
| G2.9 | Date entered in computer | **__ __/__ __/__ __ __ __**  D D M M Y Y Y Y | | | |  | |
| **INTERVIEW BEGINS**  *Instructions to interviewer: Introduce yourself and explain the purpose of your visit. Ask to speak to the mother or to another adult who was the deceased’s main caregiver during the illness that led to death. If this is not possible, arrange a time to revisit the household when the caregiver will be home. (See example below.)*  “My name is [your name]. I am an interviewer with the ____________ project. I have been informed that a child death has occurred in your household. I am very sorry to hear this. Please accept my sympathies. For the purpose of improving health care, we are collecting information on recent child deaths in this area. I would like to talk to the mother or main caregiver of <NAME> and ask some questions about the events and any symptoms that <NAME> had during her/his illness before death.” | | | | | | | |
| **Section 3: Consent**  ***INTERVIEWER: Read the consent form to the respondent. Ask the respondent if he or she has any questions. Once any questions are answered, ask the respondent if he or she is willing to take part in the study.*** | | | | | | | |
| G3.1 | *INTERVIEWER: Did respondent give consent?* | 1. Yes 2. No | | | 🞎 ***2 → Thank respondent for their time and end the interview.*** | | |
| **Section 4: Information about the respondent**  *Read:* I would now like to ask you some general questions about yourself. | | | | | | | |
| G4.1 | What is your (the respondent’s) name? |  | | | | | |
| G4.2 | *INTERVIEWER: What is the sex of the respondent?* | 1. Male 2. Female | | | | 🞎 | |
| G4.3 | What is your relationship to the deceased child? | 1. Mother 2. Father 3. Grandmother 4. Grandfather 5. Aunt 6. Uncle 7. Brother 8. Sister 9. Birth attendant *(specify type)* 10. Other male *(specify)* 11. Other female *(specify)* | | | 🞎🞎  ___________________________  ___________________________  ___________________________ | | |
| G4.4 | How old are you? | | | | __ __ Years  *(DK = 99)* | | |
| G4.5 | Starting with the first year of primary school, how many years of school did you complete? | | | | **__ __** Years  *(<1 = 00; DK = 99)* | | ***>6 years***  ***→ GQ4.6*** |
| G4.5.1 | Now I would like you to read this sentence to me. (*Show card to respondent)*  *If she cannot read the whole sentence, probe:* Can you read any part of the sentence to me? | 1. Cannot read at all 2. Able to read only part of sentence 3. Able to read whole sentence 4. No card available to show mother | | | 🞎 | | |
| *Read:* I would now like to ask you some questions about (your / the family’s) household. Please remember that all information will be kept confidential.  *[Read "...the family's household.” if you are not conducting the interview at the household where the death was identified.]* | | | | | | | |
| G4.6 | How many people live at (this / that) household?  *[Read “…at that address?” if you are speaking of “the family’s household.”]* | | | | __ __ People  *(DK = 99)* | | |
| G4.7 | How many sleeping rooms are in the household? | | | | __ __ Rooms  *(DK = 99)* | | |
| G4.8 | Does the household have a separate room for cooking? | 1. Yes 2. No   9. Don’t know | | | 🞎 | | |
| G4.9 | Does the household have:  *[Ask about each possession, and mark each one “Yes,” “No” or “Don’t know.”]* | Does the household have:   1. electricity? 2. a radio? 3. a television? 4. a refrigerator? 5. a fixed line telephone?   Does it have:   1. a mobile telephone? 2. a computer? 3. a bicycle? 4. a car or truck?   Does the household have:   1. piped water inside the residence? 2. piped water outside the residence? 3. a well (protected or unprotected)? 4. a water vendor, water supplied by truck or bottled water? 5. surface water? | | | Yes No DK  1. □ 2. □ 9. □  1. □ 2. □ 9. □  1. □ 2. □ 9. □  1. □ 2. □ 9. □  1. □ 2. □ 9. □    1. □ 2. □ 9. □  1. □ 2. □ 9. □  1. □ 2. □ 9. □  1. □ 2. □ 9. □    1. □ 2. □ 9. □  1. □ 2. □ 9. □  1. □ 2. □ 9. □    1. □ 2. □ 9. □  1. □ 2. □ 9. □ | | |
| G4.10 | What type of toilet does the household have? | 1. Flush toilet 2. Improved pit toilet 3. Traditional pit toilet 4. Bush/field/beach 5. Other *(specify)*   9. Don’t know | | | 🞎  ___________________________ | | |
| G4.11 | What is the main kind of energy the household uses for cooking? | 1. Charcoal 2. Firewood 3. Kerosene 4. Electricity 5. Gas 6. Cow dung 7. Other *(specify)*   9. Don’t know | | | 🞎  ___________________________ | | |
| G4.12 | What is the main material used for the floor of the house?  *[If you are able to observe the floor, then mark the correct answer and do not ask this question.]* | 1. Natural/mud 2. Cement 3. Wood 4. Tiles 5. Other *(specify)*   9. Don’t know | | | 🞎  ___________________________ | | |

| **Section 5: Information about others at the interview** | | | | | | | | |
| --- | --- | --- | --- | --- | --- | --- | --- | --- |
| G5.7 | | *INTERVIEWER: Are there other people present during the interview?* | | 1. Yes 2. No | | 🞎 ***2 → GQ5.9*** | | |
| G5.8 | | *INTERVIEWER: In addition to the respondent, how many people are present during the interview?* | | | | __ __ Other people  *(DK = 99)* | | |
| G5. | 9 | *INTERVIEWER: Mark the respondent in the below table and whether s/he was present during the child’s illness and/or death. For each other person present at the interview, ask the respondent their relationship to the deceased and whether they were present during the child’s illness and/or at the death. For stillbirths and neonatal deaths, also ask if each person (other than the mother) was present during the mother’s pregnancy and delivery.* | | | | | | |
|  |  | Relationship of person to the deceased child | Mark (X)  if present at the interview | Stillbirths and neonatal deaths only | | | Neonatal & older child deaths only | |
|  |  |  |  | Present during  the pregnancy:  1. Yes / 2. No | Present at  the delivery:  1. Yes / 2. No | | Present during child’s illness:  1. Yes / 2. No | Present at the child’s death:  1. Yes / 2. No |
|  | .1 | Mother | 🞎 |  |  | | 🞎 | 🞎 |
|  | .2 | Father | 🞎 | 🞎 | 🞎 | | 🞎 | 🞎 |
|  | .3 | Grandmother | 🞎 | 🞎 | 🞎 | | 🞎 | 🞎 |
|  | .4 | Grandfather | 🞎 | 🞎 | 🞎 | | 🞎 | 🞎 |
|  | .5 | Aunt | 🞎 | 🞎 | 🞎 | | 🞎 | 🞎 |
|  | .6 | Uncle | 🞎 | 🞎 | 🞎 | | 🞎 | 🞎 |
|  | .7 | Brother | 🞎 | 🞎 | 🞎 | | 🞎 | 🞎 |
|  | .8 | Sister | 🞎 | 🞎 | 🞎 | | 🞎 | 🞎 |
|  | .9 | Traditional birth attendant | 🞎 | 🞎 | 🞎 | | 🞎 | 🞎 |
|  | .10 | Other male (specify:  __________________) | 🞎 | 🞎 | 🞎 | | 🞎 | 🞎 |
|  | .11 | Other female (specify  __________________) | 🞎 | 🞎 | 🞎 | | 🞎 | 🞎 |

| **VA Section 1: Background (FOR STILLBIRTHS, NEONATAL & CHILD DEATHS 0—59 MONTHS OLD)** | | | | |
| --- | --- | --- | --- | --- |
| V1.1 | Was the deceased a singleton or multiple birth?  *[If two or more children are born at the same time, it is counted as a multiple birth, even if one or more of the babies are born dead.]* | | 1. Singleton 2. Multiple   9. Don’t know | 🞎 ***1 or 9 → VQ1.3*** |
| V1.2 | Was this the first, second, or later in the birth order? | | 1. First 2. Second 3. Third or more   9. Don’t know | 🞎 |
| V1.3 | *If the mother is present, mark “Yes” and do not ask this question.*  Is the mother still alive? | | 1. Yes 2. No | 🞎 ***1 → VQ1.6*** |
| V1.4 | Did the mother die during or after the delivery? | | 1. During 2. After   9. Don’t know | 🞎 ***1 or 9 → VQ1.6*** |
| V1.5 | How long after the delivery did the mother die?  *[Record days if less than 28 days—if less than 24 hours, record “00” days;*  *Record months if 28 days or more]* | | | **__ __** Days  *(DK = 99)* |
|  |  |  |  | **__ __** Months  *(DK = 99)* |
| V1.6 | Where was the deceased born? | | 1. Hospital 2. Other health provider or facility 3. On route to a health provider or facility 4. Home 5. Other *(specify)*   9. Don’t know | 🞎  ___________________________ |
| V1.7 | At the time of the delivery was the deceased:  *[Read the question and slowly read the first four choices. Respondent should hear all four choices & then respond.]*  *[Show photos]* | | 1. Very small 2. Smaller than usual 3. About average 4. Larger than usual   9. Don’t know | 🞎 |
| V1.8 | What was the weight of the deceased at birth? | | | **__ __ __ __** Grams  *(DK = 9999)* |
| V1.9 | What was the sex of the deceased? | | 1. Male 2. Female   9. Don’t know | 🞎 |
| V1.10 | What was the delivery date?  *Compare the delivery date just stated by the respondent to the birth date from the prior record (GQ1.4). Discuss any inconsistency with the respondent to confirm or correct the stated delivery date. You cannot change the prior record’s date.* | | | **__ __/__ __/__ __ __ __**  D D M M Y Y Y Y  *(DK = 99/99/9999)* |
| V1.11 | Was the child born alive or dead? | | 1. Alive 2. Dead   9. Don’t know | 🞎 |
| V1.12 | Did the baby ever cry? | | 1. Yes 2. No   9. Don’t know | 🞎 |
| V1.13 | Did the baby ever move? | | 1. Yes 2. No   9. Don’t know | 🞎 |
| V1.14 | Did the baby ever breathe? | | 1. Yes 2. No   9. Don’t know | 🞎 |
| V1.15 | *Refer to VQ1.11–1.14. If “Dead” & no crying, movement or breathing, mark “Stillbirth.” If “Alive” & VQ1.12–1.14 = “No,” or if “Dead” and VQ1.12, 1.13 or 1.14 = “Yes,” then discuss & correct.* | | 1. Stillbirth 2. Live birth | 🞎 ***2 → VQ1.20*** |
| **Stillbirths** | | | | |
| V1.16 | Were there any bruises or signs of injury on the baby’s body at birth? | | 1. Yes 2. No   9. Don’t know | 🞎 |
| V1.17 | Was the baby’s body (skin and tissue) pulpy? | | 1. Yes 2. No   9. Don’t know | 🞎 |
| V1.18 | Was any part of the baby physically abnormal at the time of delivery? (for example: body part too large or too small, additional growth on body) | | 1. Yes 2. No   9. Don’t know | 🞎 ***2 or 9 → SQ3.1*** |
| V1.19 | What were the abnormalities?  *Ask for the following abnormalities*  *[Mark all that apply – Show photos]* | | 1. Was the head size very small at the time of birth 2. Was the head size very large at the time of birth 3. Was there a mass defect on the back of head or spine 4. Was there any other abnormality *(If “Yes,” then specify)* | Yes No  1. □ 2. □    1. □ 2. □  1. □ 2. □  1. □ 2. □ __________________ |
| ***Inst_1: STOP. After completing VQ1.19 → SQ3.1 (Maternal history)*** | | | | |
| **Live births** | | | | |
| V1.20 | | How old was the child when the illness started?  *[Record days if less than 28 days—if less than 24 hours, record “00” days;*  *Record months if 28 days-11 months;*  *Record years if 1 year or older.]* | | **__ __** Days  *(DK = 99)* |
|  |  |  |  | **__ __** Months  *(DK = 99)* |
|  |  |  |  | **__ __** Years  *(DK = 99)* |
| V1.21 | | How long did the illness last?  *[Record days if less than 28 days—if less than 24 hours, record “00” days;*  *Record months if 28 days or more.]* | | **__ __** Days  *(DK = 99)* |
|  |  |  |  | **__ __** Months  *(DK = 99)* |
| V1.22 | | Where did the deceased die? | 1. Hospital 2. Other health provider or facility 3. On route to a health provider or facility 4. Home 5. Other *(specify)*   9. Don’t know | 🞎  ___________________________ |
| V1.24 | | What was the date of death?  *Compare the date of death just stated by the respondent to the date of death from the prior record (GQ1.5). Discuss any inconsistency with the respondent to confirm or correct the stated date. You cannot change the prior record’s date.* | | **__ __/__ __/__ __ __ __**  D D M M Y Y Y Y  *(DK = 99/99/9999)* |
| V1.25 | | ***AGE AT DEATH***  ***Record only the calculated age OR the stated age. First try to calculate the age. If this is not possible, then ask the respondent for the child’s age at death.*** | | |
|  |  | *CALCULATE THE AGE AT DEATH*  *Record the delivery date from VQ1.10:* **__ __/__ __/__ __ __ __**  D D M M Y Y Y Y  *(Don’t Know = 99/99/9999)*  *Record the date of death from VQ1.24:* **__ __/__ __/__ __ __ __**  D D M M Y Y Y Y  *(Don’t Know = 99/99/9999)*  *Now, if possible, calculate the age at death (VQ1.24 – VQ1.10). If only the month and year are known, you may still be able to calculate the approximate age in months or years. Discuss the calculated age with the respondent:* I have calculated that the child was (about) <CALCULATED AGE> at death. Is this correct?  *If the respondent does not agree with the calculated age, then again discuss the delivery date and date of death to make sure that these are correct. If the calculated age at death cannot be resolved, then go below to the “STATED AGE” box.*  *Once the age at death is calculated, check VQ1.20 and VQ1.21 to make sure that the age at illness onset and the illness duration are consistent with the age at death. For example, the age at onset + duration cannot be greater than the age at death.*  *[Record days if less than 28 days—if less than 24 hours, record “00” days; Record months if 28 days-11 months; Record years if 1 year or older.]*  *After recording the calculated age* ***→ VQ1.26*** | | **__ __** Days ***(if < 28 days)***  *(DK = 99)* |
|  |  |  |  | **__ __** Months ***(if 1-11 months)***  *(DK = 99)* |
|  |  |  |  | **__ __** Years ***(if 1 year or older)***  *(DK = 99)* |
|  |  | *STATED AGE AT DEATH (Ask only if the calculated age cannot be determined)*  How old was the deceased at the time of death?  *Compare the age at death just stated by the respondent to the child’s last known age from the prior record (GQ1.6). Discuss any inconsistency with the respondent to confirm or correct the stated age. You cannot change the prior record’s age. Partly known delivery and death dates might help resolve the stated age. For example, if the child was born and died in the same month, then this is likely a neonatal death.*  *Once the age at death is determined, check VQ1.20 and VQ1.21 to make sure that the age at illness onset and the illness duration are consistent with the age at death. For example, the age at onset + duration cannot be greater than the age at death.*  *[Record days if less than 28 days—if less than 24 hours, record “00” days; Record months if 28 days-11 months; Record years if 1 year or older.]* | | **__ __** Days ***(if < 28 days)***  *(DK = 99)* |
|  |  |  |  | **__ __** Months ***(if 1-11 months)***  *(DK = 99)* |
|  |  |  |  | **__ __** Years ***(if 1 year or older)***  *(DK = 99)* |
| V1.26 | | *Mark the baby’s age at the time of death.*  *[Use the calculated age (VQ1.24 – VQ1.10) if known, or the stated age (VQ1.25). If both the calculated and stated ages are unknown, then use your best judgment to mark the child’s age at death.]* | 1. Less than 28 days old 2. 1-59 months old | 🞎 ***2 → SQ5b.1*** |

| **SA Module 3 and VA Section 2: Maternal history (FOR STILLBIRTHS AND NN DEATHS < 28 DAYS OLD)**  *Read:* Now, I would like to ask you some questions about (your / the mother’s) health and (your / her) pregnancy with <NAME>.  *Here and in the following questions, read “…the mother…,” “…her…” and “…she…” if the mother is not the respondent.* | | | | |
| --- | --- | --- | --- | --- |
| S3.1 | Before the pregnancy with <NAME>, did (you / the mother) suffer from any of the following known conditions:  *[Read out all options and check “Yes,” “No” or “Don’t know” for each.]*  *If “Yes,” then ask:* Did (you / she) undergo treatment for this condition during the pregnancy? | 1. High blood pressure 2. Heart disease 3. Diabetes 4. Epilepsy/convulsion 5. Other   *(specify other)* | Suffered from  Yes No DK  1. □ 2. □ 9. □  1. □ 2. □ 9. □  1. □ 2. □ 9. □  1. □ 2. □ 9. □  1. □ 2. □ 9. □ | Treatment  Yes No DK  1. □ 2. □ 9. □  1. □ 2. □ 9. □  1. □ 2. □ 9. □  1. □ 2. □ 9. □  1. □ 2. □ 9. □ |
|  |  |  | ___________________________ | |
| S3.2 | During the pregnancy, did (you / the mother) see anyone for antenatal care? | 1. Yes 2. No   9. Don’t know | 🞎 ***2 or 9 → SQ3.3*** | |
| S3.2.1 | Whom did (you / she) see? Anyone else?  *[Probe, and record all persons seen.]* | 1. Health care provider 2. TBA/Religious healer 3. Relative/neighbor/friend 4. Other *(specify)*   *(_____________________________)*  9. Don’t know | 1. □  2. □  **3. □**  4. □  ***SQ3.3***  9. □ | |
| S3.2.2 | How many times did (you / the mother) receive antenatal care from a health care provider during this pregnancy? | | __ __ Times  *(DK = 99)* | |
| S3.2.3 | During which month of the pregnancy did (you / the mother) last receive antenatal care from a health care provider? | | __ __ Month  *(DK = 99)* | |
| S3.2.4 | During this pregnancy, did the provider do any of the following for (you / the mother) at least once?  *[Read out all options and check “Yes,” “No” or “Don’t know” for each.]*  *[LOCAL ADAPTATION: Additional high energy and high protein foods to mention If the respondent asks]* | 1. Did the provider measure (your / her) blood pressure? 2. Did (you / she) give a urine sample? 3. Did (you / she) give a blood sample? 4. Did the provider tell (you / her) to eat more high energy foods like <HIGH ENERGY FOODS> and high protein foods like <HIGH PROTEIN FOODS> than when not pregnant? 5. Did the provider tell (you / her) about the danger signs during pregnancy? 6. Did the provider tell (you / her) where to go if (you / she) had any danger signs? | Yes No DK    1. □ 2. □ 9. □  1. □ 2. □ 9. □  1. □ 2. □ 9. □  1. □ 2. □ 9. □  1. □ 2. □ 9. □  1. □ 2. □ 9. □ | |
| S3.3 | Please tell me the danger signs during pregnancy or labor and delivery that you should seek care for immediately.  *Probe:* Tell me as many of the danger signs as you can.  *Probe:* Can you tell me any others?  *[Check each danger sign mentioned.]* | 1. Vaginal bleeding 2. Convulsions/fits 3. Severe headache with blurred vision 4. Fever and too weak to get out of bed 5. Severe abdominal pain 6. Fast or difficult breathing 7. Painful contractions every 20 minutes or less for 12 hours or more 8. Broken water for 12 hours or more 9. Bloody, sticky discharge 12 hrs or more 10. No immediate danger sign mentioned | 1. □  2. □  3. □  4. □  5. □ **___** Mentioned  6. □  7. □  8. □  9. □  10. □ | |
| S3.4 | During this pregnancy, (were you / was the mother) given an injection in the arm to prevent the baby from getting tetanus, that is, convulsions after birth? | 1. Yes 2. No   9. Don’t know | 🞎 ***2 or 9 → SQ3.5*** | |
| S3.4.1 | During this pregnancy, how many times did (you / she) get this injection? | | __ Times  *(DK = 9)* | |
| S3.5 | At any time before this pregnancy, did (you / the mother) receive any tetanus injection, either to protect yourself or another baby? | 1. Yes 2. No   9. Don’t know | 🞎 ***2 or 9 → SQ3.6*** | |
| S3.5.1 | Before this pregnancy, how many other times did (you / she) receive a tetanus injection?  *[If 7 or more time, record “7.”]* | | __ Times  *(DK = 9)* | |
| S3.6 | *Skip SQ3.6-3.7.1 in areas wo/malaria.*  During this pregnancy, did (you / the mother) sleep under an insecticide treated bednet? | 1. Yes, usually or always 2. Yes, sometimes 3. Never   9. Don’t know | 🞎 | |
| S3.7 | During this pregnancy, did (you / the mother) take any drug to prevent (you / her) from getting malaria? | 1. Yes 2. No   9. Don’t know | 🞎 ***2 or 9 → VQ2.1*** | |
| S3.7.1 | During this pregnancy, how many times did (you / she) take this drug? | | __ __ Times  *(DK = 99)* | |
| V2.1 | Now I’d like to ask you about any problems (you / the mother) might have had during the pregnancy. Was the late part of the pregnancy (defined as the last 3 months), labor or delivery complicated by any of the following problems that started before the baby was delivered?  *[Read each complication and mark “Yes,” “No” or “Don’t know” for each.]*  *[Read “…the mother…” if the mother is not the respondent.]* | Did (you / the mother) have:   1. convulsions? 2. high blood pressure? 3. severe anemia or pallor and shortness of breath? 4. diabetes? 5. severe headache? 6. blurred vision?   (Were you / Was she):   1. too weak to get out of bed?   Did (you / the mother) have:   1. severe abdominal pain? 2. fast or difficult breathing? 3. puffy face? 4. any vaginal bleeding before labor? 5. excessive bleeding during labor or delivery? 6. fever? 7. smelly vaginal discharge?   Was the:   1. child delivered not head first? 2. cord delivered first? 3. cord around the child’s neck?   Did (you / the mother) have:   1. any other complication?   *(specify the other complication)* | Yes No DK  1. □ 2. □ 9. □  1. □ 2. □ 9. □  1. □ 2. □ 9. □  1. □ 2. □ 9. □  1. □ 2. □ 9. □  1. □ 2. □ 9. □    1. □ 2. □ 9. □  1. □ 2. □ 9. □  1. □ 2. □ 9. □  1. □ 2. □ 9. □  1. □ 2. □ 9. □    1. □ 2. □ 9. □  1. □ 2. □ 9. □  1. □ 2. □ 9. □  1. □ 2. □ 9. □    1. □ 2. □ 9. □  1. □ 2. □ 9. □  1. □ 2. □ 9. □  *____________________________* | |
| V2.2A | Did (you / the mother) have any of the following problems that started after the delivery?  *[Read each complication and mark “Yes,” “No” or “Don’t know” for each.]*  *[Read “…the mother…” if the mother is not the respondent.]* | Did (you / the mother) have:   1. convulsions? 2. heavy bleeding? 3. Fever with smelly vaginal discharge or abdominal pain? | Yes No DK  1. □ 2. □ 9. □  1. □ 2. □ 9. □  1. □ 2. □ 9. □ | |
| V2.2 | How many months long was the pregnancy? | | **__ __** Months ***≠ 99 → VQ2.4***  *(DK = 99)* | |
| V2.3 | Did the pregnancy end early, on time, or late? | 1. Early 2. On time 3. Late   9. Don’t know | 🞎 | |
| V2.4 | Was the baby moving in the last few days before the birth? | 1. Yes 2. No   9. Don’t know | 🞎 | |
| V2.5 | When did (you / the mother) last feel the baby move?  *[Read “…the mother…” if the mother is not the respondent.]*  *[Record hours if less than 24 hours; Record days if 1 day or more.]* | | **__ __** Hours before delivery  *(DK = 99)* | |
|  |  |  | **__ __** Days before delivery  *(DK = 99)* | |
| V2.6 | Did the water break before labor or during labor?  *[Note: Labor begins when contractions are no more than 20 minutes apart.]* | 1. Before 2. During   9. Don’t know | 🞎 ***2 or 9 → VQ2.8*** | |
| V2.7 | How much time before labor did the water break?  *[Record “24” if 1 day or more.]* | | **__ __** Hours  *(DK = 99)* | |
| V2.8 | What was the color of the liquor when the water broke? | 1. Green or brown 2. Clear (normal) 3. Other *(specify)*   9. Don’t know | 🞎  ____________________________ | |
| V2.9 | Was the liquor foul smelling? | 1. Yes 2. No   9. Don’t know | 🞎 | |
| V2.10 | How much time did the labor and delivery take?  *[Record “00” if less than 1 hour.]* | | **__ __** Hours  *(DK = 99)* | |
| S3.8 | Where did the delivery occur? | 1. Hospital 2. Other health provider or facility 3. On route to a health provider or facility 4. Home 5. Other *(specify___________________)*   9. Don’t know | 🞎 1-3 = Health provider  ***9 → SQ3.11*** | |
| S3.9 | Who decided that this was the right place to deliver the baby?  *[Record the one main decision maker.]* | 1. The woman, herself 2. Her husband 3. Her mother 4. Her mother-in-law 5. Her father-in-law 6. Other *(specify)*   9. Don’t know | 🞎  ___________________________ | |
| S3.10 | *If she did not go to a health provider or facility (SQ3.8 = 4-5) for the delivery, ask:* Did (you / the mother) have any concerns or problems that kept (you / her) from going to a health provider or facility for the delivery?  *If she went or was on route to a health provider or facility (SQ3.8 = 1-3) for the delivery, ask:* Did (you / the mother) have to overcome any concerns or problems to go to health provider or facility for the delivery? | 1. Yes 2. No   9. Don’t know | 🞎 ***2 or 9 → SQ3.11*** | |
| S3.10.1 | What concerns or problems did (you / she) have?  *Prompt:* Was there anything else?  *[Multiple answers allowed.]* | 1. Did not think she was sick enough to need health care 2. No one available to go with her 3. Too much time from her regular duties 4. Someone else had to decide *(specify)* 5. Too far to travel 6. No transportation available 7. Cost (transport, health care, other) 8. Not satisfied with available health care 9. Symptom(s) required traditional care 10. Thought she was too sick to travel 11. Thought she/baby will die despite care 12. Was late at night (transportation or provider not available) 13. Fears exposure to male health provider 14. Other *(specify)*   99.Don’t know | 1. □  2. □  3. □  4. □ _______________________  5. □  6. □  7. □  8. □  9. □  10. □  11. □  12. □  13. □  14. □ ______________________  99. □ | |
| S3.11 | Who (at the facility) delivered the baby?  *[Read “...at the facility...” if she delivered at a health facility.]* | 1. Doctor 2. Nurse/midwife 3. Relative/neighbor/friend 4. Self (the mother) 5. Traditional birth attendant 6. Other *(specify)*   9. Don’t know | 🞎  ___________________________ | |
| S3.12 | How soon after labor started did the <BIRTH ATTENDANT> first attend the mother?  *[Discuss that labor starts with painful contractions every 20 minutes or less.]*  *[Mark days &/or hours as needed: e.g. 00 day, 06 hours]* | | __ __ Days  *(DK = 99)* | |
|  |  |  | __ __ Hours  *(DK = 99)* | |
| S3.13 | Did the birth attendant use a pictorial graph to follow the progress of (your / the mother’s) labor? | 1. Yes 2. No   9. Don’t know | 🞎 | |
| S3.14 | Did the birth attendant wash her hands with soap and water or wear surgical gloves before assisting with the birth? | 1. Yes, washed with soap and water 2. Yes, wore surgical gloves 3. No   9. Don’t know | 🞎 | |
| S3.15 | On what surface did (you / the mother) deliver? | 1. Labor bed 2. Solid floor with mackintosh/cover 3. Solid washed floor 4. Solid unwashed floor 5. Dirt/soil/mud/straw floor 6. Other *(specify)*   9. Don’t know | 🞎  ___________________________ | |
| V2.17 | Was the delivery...?  *[Read the choices and mark ONE.]* | 1. Vaginal with forceps 2. Vaginal without forceps 3. Vaginal (don’t know) 4. C-section   9. Don’t know | 🞎 | |
| V2.18 | During labor but before delivery, did (you / the mother) receive any kind of injection?  *[Read “…the mother…” if the mother is not the respondent.]* | 1. Yes 2. No   9. Don’t know | 🞎 | |

| **SA Module 4: Careseeking for maternal complications (FOR STILLBIRTHS AND NN DEATHS < 28 DAYS OLD)**  *Read:* Now, I would like to ask you some questions about (your / the mother’s) careseeking during the pregnancy with <NAME>. | | | | | | | |
| --- | --- | --- | --- | --- | --- | --- | --- |
| S4.1 | **Maternal symptoms:**  *First look back at the maternal VA symptoms in VQ2.1 (options 1-14), 2.2, 2.7 and 2.10 (options 15-17). Mark (“X”) these in the “Symptoms in the last 3 months” column.*  *If she had any symptom(s), then read:* Earlier, you mentioned that (you / the mother) had <SYMPTOM(S)> during the last 3 months of the pregnancy or during labor or delivery. Which of the symptoms started before labor? And which started with or during labor or delivery, including any that may have brought on the labor?  *[Remind the respondent that labor starts with painful contractions every 20 minutes or less. Then review each reported symptom with her to determine which started before labor and which started* with or during labor *or delivery. Do not include any symptoms here that started after the baby was delivered.]* | | 1. Convulsions 2. High blood pressure 3. Severe anemia or (pallor and SOB) 4. Diabetes 5. Severe headache 6. Blurred vision 7. Too weak to get out of bed 8. Severe abdominal pain (not labor pain) 9. Fast or difficult breathing 10. Puffy face 11. Any vaginal bleeding before labor 12. Excessive bleeding during labor or dlvr. 13. Fever 14. Smelly vaginal discharge 15. Early/preterm labor (less than 9 mnths) 16. Water broke 6 hrs or more before labor 17. Labor for 12 hours or more 18. Other *(specify)*   *(____________________________)*   1. No symptoms during last 3 months 2. No symptoms before labor | | Symptoms during last 3 months  Yes  □  □  □  □  □  □  □  □  □  □  □  □  □  □  □  □  □  □  □ ***→ Inst_8*** | | Started (related to labor/delivery)  Before W/D DK  1. □ 2. □ 9. □  1. □ 2. □ 9. □  1. □ 2. □ 9. □  1. □ 2. □ 9. □  1. □ 2. □ 9. □  1. □ 2. □ 9. □  1. □ 2. □ 9. □  1. □ 2. □ 9. □  1. □ 2. □ 9. □  1. □ 2. □ 9. □  1. □ 2. □ 9. □  1. □ 2. □ 9. □  1. □ 2. □ 9. □  1. □ 2. □ 9. □  1. □ 2. □ 9. □  1. □ 2. □ 9. □  1. □ 2. □ 9. □  1. □ 2. □ 9. □  □ ***→ SQ4.11*** |
| S4.2 | Did (you / the mother) seek care from any person or health facility for (any of) the pregnancy symptom(s) that started before labor?  *[Read “…for any of…” if she had more than one pregnancy symptom.]* | | 1. Yes 2. No   9. Don’t know | | 🞎 ***2 → SQ4.4***  ***9 → Inst_2*** | | |
| S4.2.1 | Where did (you / she) seek this care?  *Prompt:* Was there anywhere else?  *[Multiple answers allowed.]* | | 1. Hospital 2. NGO or government clinic 3. Private doctor/clinic 4. Community nurse or midwife 5. TBA/village doctor/quack/other non-formal or traditional provider 6. Relative, neighbor or friend 7. Other *(specify)*   *(*______________________________*)*  99. Don’t know | | 1. □  2. □  3. □ = Health provider  4. □  5. □  ***SQ4.4***  6. □  7. □  9. □ ***→ Inst_2*** | | |
| S4.3 | *If more than one symptom started before labor and she sought care from a health provider (SQ4.2.1 = 1-4), ask:*  For which symptom or symptoms that started before labor did (you / she) seek care from a health provider or facility? | | 1. Convulsions □ 2. High blood pressure □ 3. Severe anemia or (pallor and SOB) □ 4. Diabetes □ 5. Severe headache □ 6. Blurred vision □ 7. Too weak to get out of bed □ 8. Severe abdominal (not labor) pain □ 9. Fast or difficult breathing □ | | 1. Puffy face □ 2. Any bleeding before labor □ 3. – blank – 4. Fever □ 5. Smelly vaginal discharge □ 6. – blank – 7. Water broke >6 hrs bfr. labor □ 8. – blank – 9. Other *(specified in SQ4.1)* □ | | |
| S4.4 | *If she never went to a health provider (SQ4.2 = 2 or SQ4.2.1 ≠ 1-4) for any of the pregnancy symptoms, ask:* Did (you / the mother) have any concerns or problems that kept (you / her) from going to a health provider or facility for the symptom(s) that started before labor?  *If she went to health provider (SQ4.2.1 = 1-4) for any pregnancy symptom(s), ask:* Did (you / the mother) have to overcome any concerns or problems to go to a health provider or facility for the symptom(s) that started before labor? | | 1. Yes 2. No   9. Don’t know | | 🞎 ***2 or 9 → Inst_1*** | | |
| S4.4.1 | What concerns or problems did (you / she) have?  *Prompt:* Was there anything else?  *[Multiple answers allowed.]* | | 1. Did not think was sick enough to need health care 2. No one available to go with her 3. Too much time from her regular duties 4. Someone else *(specify)* had to decide 5. Too far to travel 6. No transportation available 7. Cost (transport, health care, other) 8. Not satisfied with available health care 9. Symptom(s) required traditional care 10. Thought she was too sick to travel 11. Thought she/baby will die despite care 12. Fears exposure to male health provider 13. Other *(specify)*   99.Don’t know | | 1. □  2. □  3. □  4. □ _______________________  5. □  6. □  7. □  8. □  9. □  10. □  11. □  12. □  13. □ ______________________  99. □ | | |
| ***Inst_1: If SQ4.2 = 2 or SQ4.2.1 ≠ 1-4 (Never went to a health provider for any pregnancy symptoms) →*** ***Inst_2*** | | | | | | | |
| S4.5 | Did any health provider or facility refer (you / her) to another health provider or facility for (any of) the symptom(s) that started before labor? | | 1. Yes 2. No   9. Don’t know | | 🞎 ***2 or 9 → SQ4.6*** | | |
| S4.5.1 | Did (you / she) go to the provider or facility to which (you were / she was) referred? | | 1. Yes 2. No   9. Don’t know | | 🞎 | | |
| S4.6 | How many different health providers or facilities did (you / the mother) see for the pregnancy symptom(s) that started before labor? | | | | **__ __** Health providers/facilities  *(DK = 99)* | | |
| S4.7 | (Were you / Was the mother) admitted to hospital for (any of) the symptom(s) that started before labor? | | 1. Yes 2. No   9. Don’t know | | 🞎 | | |
| S4.8 | Please tell me everything that the provider(s) suggested that (you / the mother) do for the pregnancy symptom(s) at home?  *Prompt:* Was there anything else?  *[Multiple answers allowed.]* | | 1. Take antibiotic by mouth 2. Take antimalarial by mouth 3. Take BP medicine by mouth 4. Take other medicine by mouth 5. Rest / bed rest / decrease work 6. Return for follow-up visit(s) 7. Return or referred if worse 8. Other *(specify)* 9. Nothing   99. Don’t know | | 1. □  2. □  3. □  4. □  5. □  6. □  7. □  8. □ _______________________  9. □ ***→ Inst_2***  99. □ ***→ Inst_2*** | | |
| S4.9 | (Were you / Was the mother) able to follow all this advice? | | 1. Yes 2. No   9. Don’t know | | 🞎 ***9 → Inst_2*** | | |
| S4.10 | *If not able to follow all the advice, ask:*  Did (you / she) have any concerns or problems that kept (you / her) from following the advice?  *If able to follow all the advice, ask:*  Did (you / she) have to overcome any concerns or problems to follow the advice? | | 1. Yes 2. No   9. Don’t know | | 🞎 ***2 or 9 → Inst_2*** | | |
| S4.10.1 | What concerns or problems did (you / she) have?  *Prompt:* Was there anything else?  *[Multiple answers allowed.]* | | 1. Did not understand instructions 2. Too much time from her regular duties 3. Someone else *(specify)* decided 4. Cost too much 5. Problem required traditional care 6. Advised care not needed or helpful 7. Advised care might harm unborn child 8. Thought she/baby will die despite care 9. Other *(specify)*   99.Don’t know | | 1. □  2. □  3. □ _______________________  4. □  5. □  6. □  7. □  8. □  9. □ _______________________  99. □ | | |
| ***Inst_2: Refer to SQ4.1: If no labor or delivery symptoms*** ***→******Inst_8*** | | | | | | | |
| S4.11 | Now let’s talk about the labor and delivery symptom(s). You said earlier that the symptom(s) that started with or during labor or delivery (was / were) <SYMPTOM(S)>.  *[Read and mark the SQ4.1 symptom(s) confirmed by the respondent. Correct the SQ4.1 responses if necessary.]* | | 1. Convulsions □ 2. High blood pressure □ 3. Severe anemia or (pallor and SOB) □ 4. – blank – 5. Severe headache □ 6. Blurred vision □ 7. Too weak to get out of bed □ 8. Severe abdominal (not labor) pain □ 9. Fast or difficult breathing □ | | 1. Puffy face □ 2. Any bleeding before labor □ 3. Excess bleed during L or D □ 4. Fever □ 5. Smelly vaginal discharge □ 6. Early/preterm labor (<9 mnth) □ 7. Water broke >6 hrs bfr. labor □ 8. Labor for 12 hours or more □ 9. Other *(specified in SQ4.1)* □ | | |
| S4.12 | Where (were you / was the mother) when (this / the first) symptom began?  *[Read “…the first…” if she had more than one labor or delivery symptom.]* | | 1. Home 2. On route to a health provider or facility 3. At the health provider or facility where she went for normal labor 4. Other *(specify)*   9. Don’t know | | 🞎 ***3 → SQ4.17***  ___________________________ | | |
| S4.13 | Did (you / she) ever receive, seek or try to seek any care or treatment for (any of) the labor or delivery symptom(s)?  *[Read “…any of the symptoms” if she had more than one symptom.]* | | 1. Yes 2. No   9. Don’t know | | 🞎***2 → SQ4.14***  ***9 → SQ4.17*** | | |
| S4.13.1 | What was the first thing (you / she) did for the symptom(s)?  *[Mark only the first action taken.]* | | 1. Home treatment (at her own home, or by a relative, neighbor, or friend)   Sought or tried to seek care from a:   1. Hospital 2. NGO or government clinic 3. Private doctor/clinic 4. Community nurse or midwife 5. Pharmacist or drug seller 6. TBA/village doctor/quack/other non-formal or traditional provider 7. Other *(specify)*   99. Don’t know | | 🞎🞎***99 → SQ4.16***  __________________________ | | |
| S4.14 | Who decided that this was the right thing to do (at that time)?  *[Read “…at that time” if she received or sought any care or treatment.]*  *[Only one response allowed. Record the main decision maker.]* | | 1. The woman, herself 2. Her husband 3. Her mother 4. Her mother-in-law 5. Her father-in-law 6. Other *(specify)*   9. Don’t know | | 🞎  ___________________________ | | |
| S4.15 | *If she did not go to a health provider (SQ4.13 = 2 or SQ4.13.1 = 1 or 6-8), ask:* Did (you / the mother) have any concerns or problems that kept (you / her) from going to a health provider (at that time)?  *Do not read “…at that time?” if she never received or sought any care or treatment (SQ4.13 = 2).*  *If she went to a health provider (SQ4.13.1 = 2-5), ask:* Did (you / the mother) have to overcome any concerns or problems to go to the <HEALTH PROVIDER> at that time? | | 1. Yes 2. No   9. Don’t know | | 🞎 ***2 or 9 →***  ***Inst_2.5 (if SQ4.13 = 2), or Inst_3 (if SQ4.13.1 = 2-5)*** | | |
| S4.15.1 | What concerns or problems did (you / she) have?  *Prompt:* Was there anything else?  *[Multiple answers allowed.]* | | 1. Did not think she was sick enough to need health care 2. No one available to go with her 3. Too much time from her regular duties 4. Someone else had to decide *(specify)* 5. Too far to travel 6. No transportation available 7. Cost (transport, health care, other) 8. Not satisfied with available health care 9. Symptom(s) required traditional care 10. Thought she was too sick to travel 11. Thought she/baby will die despite care 12. Was late at night (transportation or provider not available) 13. Fears exposure to male health provider 14. Other *(specify)*   99.Don’t know | | 1. □  2. □  3. □  4. □ _______________________  5. □  6. □  7. □  8. □  9. □  10. □  11. □  12. □  13. □  14. □ ______________________  99. □ | | |
| ***Inst_2.5: If SQ4.13 = 2 →*** ***SQ4.17***  ***Inst_3: If SQ4.13.1 = 2-5 (First went to a health provider or facility)******→*** ***SQ4.16.1*** | | | | | | | |
| S4.16 | Did (you / she) ever seek or try to seek care from a health provider or facility for (any of) the labor or delivery symptom(s)? | | 1. Yes 2. No   9. Don’t know | | 🞎***2 or 9 → SQ4.17*** | | |
| S4.16.1 | Please tell me all the types of health providers and facilities where (you / she) sought or tried to seek care for (any of) the labor or delivery symptom(s).  *Prompt:* Anywhere else?  *[Multiple answers allowed.]* | | 1. Hospital 2. NGO or government clinic 3. Private doctor/clinic 4. Community nurse or midwife   9. Don’t know | | 1. □  2. □  3. □  4. □  9. □ | | |
| S4.17 | *Refer to SQ3.8 to determine the delivery place. Discuss with respondent to confirm or correct the delivery place.*  *Discuss & resolve inconsistencies, for example, if SQ4.13 or 4.16 = “No,” but the mother delivered in a health facility.* | | 1. Hospital 2. Other health provider or facility 3. On route to a health provider or facility 4. Home 5. Other *(specify)*   9. Don’t know | | 🞎 1-3 = Health provider  ___________________________ | | |
| S4.18 | So, including where (you / the mother) went or tried to go for the labor or delivery symptom(s) and for the delivery, how many health providers or facilities did (you / she) go to?    *[If SQ4.16 = 2 and SQ4.17 = 4 or 5 → record ‘00’ health providers/facilities]*  *[If SQ4.16 = 2 and SQ4.17 = 1-3 → record ‘01’ health provider/facility]*  *[If SQ4.16 = 2 and SQ4.17 = 9 → record ‘99’ health providers/facilities]*  *[If SQ4.16 = 9 → record ‘99’ health providers/facilities]* | | | | **__ __** Health providers/facilities | | |
| ***Inst_4: If SQ4.12 = 3 (Symptoms began at the health provider where she went for normal labor) → SQ4.22*** | | | | | | | |
| ***Inst_5: If SQ4.16 = 2 or 9 & SQ4.17 = 4-9 (No health provider seen/sought for the symptoms/delivery) → Inst_8*** | | | | | | | |
| ***Inst_5.5: If SQ4.1 = only 1 labor or delivery symptom OR If SQ4.16 = 2 or 9*** ***→******SQ4.21*** | | | | | | | |
| S4.19 | Was there any particular symptom or symptoms for which (you / the mother) went to the (first) health provider?  *[Read “…the first health provider?” if she went to more than one provider.]* | | 1. Yes 2. No   9. Don’t know | | 🞎***2 or 9 → SQ4.21*** | | |
| S4.20 | For which symptom(s) did (you / she) go? | | 1. Convulsions □ 2. High blood pressure □ 3. Severe anemia or (pallor and SOB) □ 4. – blank – 5. Severe headache □ 6. Blurred vision □ 7. Too weak to get out of bed □ 8. Severe abdominal (not labor) pain □ 9. Fast or difficult breathing □ | | 1. Puffy face □ 2. Any bleeding before labor □ 3. Excess bleed during L or D □ 4. Fever □ 5. Smelly vaginal discharge □ 6. Early/preterm labor (<9 mnth) □ 7. Water broke >6 hrs bfr. labor □ 8. Labor for 12 hours or more □ 9. Other *(specified in SQ4.1)* □ | | |
| S4.21 | How long after the labor or delivery symptom(s) began was it decided to go to the (first) health provider?  *[Read “…to the first…” if she went or tried to go to more than one health provider.*  *[Mark days, hours &/or minutes as needed: e.g. 00 day, 02 hours, 10 minutes]* | | | | **__ __** Days  *(DK = 99)* | | |
|  |  |  |  |  | **__ __** Hours  *(DK = 99)* | | |
|  |  |  |  |  | **__ __** Minutes  *(DK = 99)* | | |
| ***Labor and delivery matrix instructions:*** *Ask the following questions for the first and last health providers where she sought/tried to seek care for the labor and delivery symptoms. If she delivered at a health provider/facility or at home or on route while trying to go to a health provider/facility, then that should be the first health provider (if she went to only one) or the last health provider. Ask all the questions for the first provider before going on to the last.*  *Before asking about the first health provider, read:*  Now I would like to ask about (your / the mother’s) visit to the (first) health provider. *[Read “first” if she went or tried to go to more than one provider.]*  *Before asking about the last health provider, read:*  Now I would like to ask about (your / the mother’s) visit to the last health provider. | | | | | | | |
| **– LABOR AND DELIVERY MATRIX QUESTIONS –** | | | | **FIRST HEALTH PROVIDER** | | **LAST HEALTH PROVIDER** | |
| What was the name of the (first / last) health provider or facility where (you / the mother) (sought care for the labor or delivery symptom(s) / delivered the baby / tried to deliver the baby)?  *Probe to identify the type of provider.* | | 1. Hospital (Government) 2. Hospital (NGO) 3. Hospital (Private) 4. Health center (Government) 5. Health center (NGO) 6. Health post (Government) 7. Health post (NGO) 8. Private doctor/clinic (Formal) 9. Private doctor/clinic (?Formal?) 10. Trained community nurse/midwife   99. Don’t know | | S4.22  🞎🞎  ___________________  (Name of Provider/Facility) | | S4.32  🞎🞎  ___________________  (Name of Provider/Facility) | |
| After (deciding to seek care / being referred), how much time passed before going to the <FIRST/LAST HEALTH PROVIDER>?  *[Discuss that this might include the time needed to arrange for transportation and money to go to the provider/facility, or to provide home care or go to a traditional provider before going to the health provider.]*  *[If she delivered at home, record the time from decision/referral to delivery.]*  *[Mark days, hours &/or minutes as needed: e.g. 00 days, 02 hours, 10 minutes]* | | | | S4.23  **__ __** Days  *(DK = 99)* | | S4.33  **__ __** Days  *(DK = 99)* | |
|  |  |  |  | **__ __** Hours  *(DK = 99)* | | **__ __** Hours  *(DK = 99)* | |
|  |  |  |  | **__ __** Minutes  *(DK = 99)* | | **__ __** Minutes  *(DK = 99)* | |
| Was there any cost to travel to the <FIRST/LAST HEALTH PROVIDER> or pay for (your / the mother’s) care there? | | 1. Yes 2. No   9. Don’t know | | S4.24  🞎 ***2 or 9 → SQ4.25*** | | S4.34  🞎 ***2 or 9 → SQ4.35*** | |
| How did (you / the mother) arrange for the money for these expenses?  *[Multiple answers allowed.]* | | 1. Had available 2. Borrowed 3. Sold assets 4. Help from kin/relatives 5. Community fund 6. Govt. scheme 7. Other   9. Don’t know | | S4.24.1  1. □  2. □  3. □  4. □  5. □  6. □  7. □  9. □ | | S4.34.1  1. □  2. □  3. □  4. □  5. □  6. □  7. □  9. □ | |
| What transportation method was used to go there?  *[Multiple answers allowed.]* | | 1. Walk 2. Bicycle/animal/cart/boat 3. Bus 4. Taxi/auto/trecker/motorcycle 5. Ambulance 6. Other 7. Could not arrange transport   9. Don’t know | | S4.25  1. □ ***If only walk***  2. □ ***→ SQ4.26.1***  3. □  4. □  5. □  6. □  7. □ ***→ SQ4.26.1***  9. □ | | S4.35  1. □ ***If only walk***  2. □ ***→ SQ4.36.1***  3. □  4. □  5. □  6. □  7. □ ***→ SQ4.36.1***  9. □ | |
| How much did the transportation cost? | | | | S4.26  **__ __ __ __** unit  *(DK = 9999)* | | S4.36  **__ __ __ __** unit  *(DK = 9999)* | |
| Did (you / the mother) reach the <FIRST/LAST HEALTH PROVIDER> before delivering the baby?  *If “No,” discuss with respondent to reach correct response: 2, 3 or 4.]* | | 1. Yes, reached before delivering 2. No, delivered before setting out 3. No, delivered on route to provider 4. No, could not reach this provider – did not set out/returned home/took other action   9. Don’t know | | S4.26.1  🞎 ***2, 3 → Inst_8***  ***4, 9 → Inst_7*** | | S4.36.1  🞎 ***2-9 → Inst_8*** | |
| How long did it take to travel to the <FIRST/LAST HEALTH PROVIDER>?  *[Mark hours &/or minutes as needed: e.g. 05 hours, 30 minutes]* | | | | S4.27  **__ __** Hours  *(DK = 99)* | | S4.37  **__ __** Hours  *(DK = 99)* | |
|  |  |  |  | **__ __** Minutes  *(DK = 99)* | | **__ __** Minutes  *(DK = 99)* | |
| What did the <FIRST/LAST HEALTH PROVIDER> do for (your / the mother’s) (labor or delivery symptom(s) / delivery)?  *Prompt:* Was there anything else?  *[Multiple answers allowed.]* | | 1. Gave oxygen for the baby 2. Gave antibiotics by mouth 3. Gave antimalarial by mouth 4. Gave BP medicine by mouth 5. Other medicine by mouth *(specify)* 6. Gave medicine to stop bleeding 7. Gave medicine to stop convulsions 8. Gave medicine to strengthen labor 9. Gave medicine to stop labor 10. Gave medicine for baby’s lungs 11. Gave IM medicine 12. Gave IV fluids or medicine 13. Blood transfusion 14. Advised to buy outside medicine 15. Uterine massage 16. Did a C-section 17. Did another operation *(specify)* 18. Admitted to hospital 19. Other *(specify)* 20. Nothing   99.Don’t know | | S4.28  1. □  2. □  3. □  4. □  5. □ _______________  6. □  7. □  8. □  9. □  10. □  11. □  12. □  13. □  14. □  15. □  16. □  17. □ ______________  18. □ stayed __ __ days  19. □ ______________  20. □ ***→ SQ4.30***  99. □ ***→ SQ4.30*** | | S4.38  1. □  2. □  3. □  4. □  5. □_______________  6. □  7. □  8. □  9. □  10. □  11. □  12. □  13. □  14. □  15. □  16. □  17. □ ______________  18. □ stayed __ __ days  19. □ ______________  20. □ ***→ SQ4.40***  99. □ ***→ SQ4.40*** | |
| How much did (you / the mother) pay for these treatments and other costs related to the health care, including any admission fee, consultation, lab tests, equipment, and room and food for companions? | | | | S4.29  **__ __ __ __ __** unit  *(DK = 99999)* | | S4.39  **__ __ __ __ __** unit  *(DK = 99999)* | |
| Did the <FIRST/LAST HEALTH PROVIDER> refer (you / the mother) to another health provider or facility? | | 1. Yes 2. No   9. Don’t know | | S4.30  🞎 ***2 or 9 → SQ4.30.2*** | | 4.40  🞎 ***2 or 9 → SQ4.40.2*** | |
| Why (were you / was the mother) referred?  *[Multiple answers allowed.]* | | 1. The provider was not capable of managing the problem 2. Required supplies (e.g., drugs, IV, oxygen, blood) not available 3. Required equipment (e.g., ultrasound) not available 4. Required facility (e.g., operation room) not available   9. Don’t know | | S4.30.1  1. □  2. □  3. □  4. □  9. □ | | S4.40.1  1. □  2. □  3. □  4. □  9. □ | |
| Was the baby delivered at the <FIRST/LAST HEALTH PROVIDER>? | | 1. Yes 2. No   9. Don’t know | | S4.30.2  🞎 ***1 → Inst_8*** | | S4.40.2  🞎 ***1 → Inst_8*** | |
| ***Inst_6: Check SQ4.18 to determine if she went to another health provider*** | | | | | |  | |
| *If did not go to another health provider, ask:* Did (you / the mother) have any concerns or problems that kept (you / her) from going to another provider?  *If went to another health provider, ask:* Did (you / the mother) have to overcome any concerns or problems to go to another provider? | | 1. Yes 2. No   9. Don’t know | | S4.31  🞎***2 or 9 → Inst_7*** | | S4.41  🞎***2 or 9 → Inst_8*** | |
| What concerns or problems did (you / she) have?  *Prompt:* Was there anything else?  *[Multiple answers allowed.]* | | 1. Thought no more care needed 2. No one available to go with her 3. Too much time from regular duties 4. Someone else *(specify)* decided 5. Too far to travel 6. No transportation available 7. Cost (transport, health care, other) 8. Not satisfied with available care 9. Problem required traditional care 10. Thought too sick to travel 11. Thought she/baby will die anyway 12. Was late at night 13. She delivered before going 14. Other *(specify)*   99. Don’t know | | S4.31.1  1. □  2. □  3. □  4. □ ______________  5. □  6. □  7. □  8. □  9. □  10. □  11. □  12. □  13. □ ***→ Inst_8***  14. □ ______________  99. □ | | S4.41.1  1. □  2. □  3. □  4. □ ______________  5. □  6. □  7. □  8. □  9. □  10. □  11. □  12. □  13. □  14. □ ______________  99. □ | |
| ***Inst_7: Check SQ4.18 → If she went to another health provider*** | | | | ***…go to SQ4.32 (LAST HEALTH PROVIDER)*** | |  | |
| ***Inst_8: STOP – If VQ1.15 = 1 (Stillbirth) → VQ5.4 (Section 5: Health records)*** | | | | | | | |

| **SA Module 5a: Care of the newborn; and VA Section 3: Neonatal deaths (FOR NN DEATHS <28 DAYS OLD)**  *Read:* Now I would like to ask you about the care of the newborn child. | | | | | | | |
| --- | --- | --- | --- | --- | --- | --- | --- |
| S5a.1 | What tool was used for cutting the cord? | 1. New/from delivery kit/boiled razor blade 2. Old razor blade 3. Scissors 4. Other *(specify)*   9. Don’t know | 🞎  ___________________________ | | | | |
| S5a.2 | What material was used for tying the cord? | 1. Clean/from delivery kit/boiled piece of thread 2. Unclean piece of thread 3. Cord clamp 4. Other *(specify)*   9. Don’t know | 🞎  ___________________________ | | | | |
| S5a.3 | Was anything applied to the umbilical cord stump after birth? | 1. Yes 2. No   9. Don’t know | 🞎 ***2 or 9 → VQ3.1*** | | | | |
| S5a.3.1 | What was it? | 1. Alcohol/other antiseptic 2. Antibiotic ointment/cream/powder 3. Castor oil, mustard oil or shea butter 4. Animal dung or dirt/mud 5. Other *(specify)*   9. Don’t know | 🞎  ___________________________ | | | | |
| V3.1 | Were there any bruises or signs of injury on the baby’s body at birth? | 1. Yes 2. No   9. Don’t know | 🞎 | | | | |
| V3.2 | Was any part of the baby physically abnormal at the time of delivery? (for example: body part too large or too small, additional growth on body) | 1. Yes 2. No   9. Don’t know | 🞎 ***2 or 9 → VQ3.4*** | | | | |
| V3.3 | What were the abnormalities?  *Ask for the following abnormalities:*  *[Mark all that apply – Show photos]* | 1. Was the head size very small at the time of birth? 2. Was the head size very large at the time of birth? 3. Was there a mass defect on the back of head or spine? 4. Was there any other abnormality?   *(If “Yes,” then specify)* | Yes No  1. □ 2. □    1. □ 2. □  1. □ 2. □  1. □ 2. □  ____________________________ | | | | |
| V3.4 | Did the baby breathe immediately after birth? | 1. Yes 2. No   9. Don’t know | 🞎 ***2 → VQ3.6*** | | | | |
| V3.5 | Did the baby have difficulty breathing? | 1. Yes 2. No   9. Don’t know | 🞎 | | | | |
| V3.6 | Was anything done to try to help the baby breathe at birth? | 1. Yes 2. No   9. Don’t know | 🞎 | | | | |
| V3.7 | Did the baby cry immediately after birth? | 1. Yes 2. No   9. Don’t know | 🞎 ***1 → VQ3.9*** | | | | |
| V3.8 | How long after birth did the baby first cry?  *[Mark ONE response]* | 1. Within 5 minutes 2. Within 6-30 minutes 3. More than 30 minutes 4. Never   9. Don’t know | 🞎 ***4 → SQ5a.4*** | | | | |
| V3.9 | Did the baby stop being able to cry? | 1. Yes 2. No   9. Don’t know | 🞎 ***2 or 9 → SQ5a.4*** | | | | |
| V3.10 | How long before the baby died did the baby stop crying? | 1. Less than one day 2. One day or more   9. Don’t know | 🞎 | | | | |
| S5a.4 | How long after birth was the baby first bathed? | 1. Less than 1 hour 2. 1-23 hours 3. 24-72 hours (1-3 days) 4. More than 72 hours (3 days) 5. Not bathed   9. Don’t know | 🞎 | | | | |
| S5a.5 | Was anything done to keep the baby warm on the first day after birth? | 1. Yes 2. No   9. Don’t know | 🞎 ***2 or 9 → SQ5a.6*** | | | | |
| S5a.5.1 | What was done?  *[Multiple answers allowed.]*  *For each mentioned, ask:*  How soon after birth was this done? | 1. Dried/wiped 2. Wrapped in a blanket 3. Skin-to-skin contact 4. Incubator 5. Other   *(specify other)* | Done  1. □  2. □  3. □  4. □  5. □ | | How soon after birth  <1hr <6 6-24 >24 DK  1.□ 2.□ 3.□ 4.□ 9.□ 1.□ 2.□ 3.□ 4.□ 8.□  1.□ 2.□ 3.□ 4.□ 9.□  1.□ 2.□ 3.□ 4.□ 9.□  1.□ 2.□ 3.□ 4.□ 9.□ | | |
|  |  |  | ____________________________ | | | | |
| S5a.6 | Did (you / the mother) or a wet nurse ever breastfeed the baby? | 1. Yes 2. No   9. Don’t know | 🞎***2 or 9 → SQ5a.7*** | | | | |
| S5a.6.1 | How long after birth was the baby first put to the breast?  *[If immediately or less than 1 hour, record ’00’ hours.]*  *[If less than 24 hours, record hours; otherwise record days.]* | | __ __ Days  *(DK = 99)*  OR | | | | |
|  |  |  | __ __ Hours  *(DK = 99)* | | | | |
| S5a.6.2 | Was the baby being breastfed at the time when the fatal illness began? | 1. Yes 2. No   9. Don’t know | 🞎 | | | | |
| S5a.7 | At the time the fatal illness began, was the baby being given any other liquid, including non-human milk or formula, fruit juice, tea or water, or any semisolid or soft foods such as cereal?  *[Multiple answers allowed. Probe, and record all liquids and foods given.]* | 1. Non-human milk or pre-mixed formula 2. Powdered formula mixed with a liquid 3. Juice, water and/or water-based drinks 4. ORS 5. Drops or syrups (vitamins, medicines) 6. Semi-solid or soft foods 7. Nothing else, only given breast milk   9. Don’t know | 1. □  2. □  3. □  4. □  5. □  6. □  7. □  9. □ | | | | |
| V3.11 | Was the baby able to suckle in a normal way during the first day of life? | 1. Yes 2. No   9. Don’t know | 🞎 ***1 → VQ3.13*** | | | | |
| V3.12 | Did the baby ever suckle in a normal way? | 1. Yes 2. No   9. Don’t know | 🞎 ***2 or 9 → VQ3.17*** | | | | |
| V3.13 | Did the baby stop being able to suckle in a normal way? | 1. Yes 2. No   9. Don’t know | 🞎 ***2 or 9 → VQ3.17*** | | | | |
| V3.14 | How long after birth did the baby stop suckling?  *[Less than 24 hours = “00” days]* | | **__ __** Days  *(DK = 99)* | | | | |
| V3.15 | How long before s/he died did the baby stop suckling? | 1. Less than one day 2. One day or more   9. Don’t know | 🞎 | | | | |
| V3.16 | Was the baby able to open her/his mouth at the time s/he stopped suckling? | 1. Yes 2. No   9. Don’t know | 🞎 | | | | |
| V3.17 | During the illness that led to death, did the baby have difficult breathing? | 1. Yes 2. No   9. Don’t know | 🞎 ***2 or 9 → VQ3.20*** | | | | |
| V3.18 | At what age did the difficult breathing start?  *[Less than 24 hours = “00” days]* | | **__ __** Days  *(DK = 99)* | | | | |
| V3.19 | For how many days did the difficult breathing last?  *[Less than 24 hours = “00” days]* | | **__ __** Days  *(DK = 99)* | | | | |
| V3.20 | During the illness that led to death, did the baby have fast breathing? | 1. Yes 2. No   9. Don’t know | 🞎 ***2 or 9 → VQ3.23*** | | | | |
| V3.21 | At what age did the fast breathing start?  *[Less than 24 hours = “00” days]* | | **__ __** Days  *(DK = 99)* | | | | |
| V3.22 | For how many days did the fast breathing last?  *[Less than 24 hours = “00” days]* | | **__ __** Days  *(DK = 99)* | | | | |
| V3.23 | During the illness that led to death, did the baby have indrawing of the chest?  *[Show photo]* | 1. Yes 2. No   9. Don’t know | 🞎 | | | | |
| V3.24 | During the illness that led to death, did the baby have grunting?  *[Demonstrate grunting]* | 1. Yes 2. No   9. Don’t know | 🞎 | | | | |
| V3.25 | During the illness that led to death, did the baby have spasms or convulsions? | 1. Yes 2. No   9. Don’t know | 🞎 | | | | |
| V3.26 | During the illness that led to death, did the baby have fever? | 1. Yes 2. No   9. Don’t know | 🞎 ***2 or 9 → VQ3.29*** | | | | |
| V3.27 | At what age did the fever start?  *[Less than 24 hours = “00” days]* | | **__ __** Days  *(DK = 99)* | | | | |
| V3.28 | How many days did the fever last?  *[Less than 24 hours = “00” days]* | | **__ __** Days  *(DK = 99)* | | | | |
| V3.29 | During the illness that led to death, did the baby become cold to touch? | 1. Yes 2. No   9. Don’t know | 🞎 ***2 or 9 → VQ3.32*** | | | | |
| V3.30 | At what age did the baby start feeling cold to touch?  *[Less than 24 hours = “00” days]* | | **__ __** Days  *(DK = 99)* | | | | |
| V3.31 | How many days did the baby feel cold to touch?  *[Less than 24 hours = “00” days]* | | **__ __** Days  *(DK = 99)* | | | | |
| V3.32 | During the illness that led to death, did the baby become lethargic, after a period of normal activity? | 1. Yes 2. No   9. Don’t know | 🞎 | | | | |
| V3.33 | During the illness that led to death, did the baby become unresponsive or unconscious? | 1. Yes 2. No   9. Don’t know | 🞎 | | | | |
| V3.34 | During the illness that led to death, did the baby have a bulging fontanelle?  *[Show photo]* | 1. Yes 2. No   9. Don’t know | 🞎 | | | | |
| V3.35 | During the illness that led to death, did the baby have pus drainage from the umbilical cord stump? | 1. Yes 2. No   9. Don’t know | 🞎 | | | | |
| V3.36 | During the illness that led to death, did the baby have redness of the umbilical cord stump? | 1. Yes 2. No   9. Don’t know | 🞎 ***2 or 9 → VQ3.38*** | | | | |
| V3.37 | Did the redness of the umbilical cord stump extend onto the abdominal skin? | 1. Yes 2. No   9. Don’t know | 🞎 | | | | |
| V3.38 | During the illness that led to death, did the baby have skin bumps containing pus or a single large area with pus? | 1. Yes 2. No   9. Don’t know | 🞎 | | | | |
| V3.39 | During the illness that led to death, did the baby have ulcer(s) (pits)? | 1. Yes 2. No   9. Don’t know | 🞎 | | | | |
| V3.40 | During the illness that led to death, did the baby have an area(s) of skin with redness and swelling? | 1. Yes 2. No   9. Don’t know | 🞎 | | | | |
| V3.41 | During the illness that led to death, did s/he have areas of the skin that turned black? | 1. Yes 2. No   9. Don’t know | 🞎 | | | | |
| V3.42 | During the illness that led to death, did the baby bleed from anywhere? | 1. Yes 2. No   9. Don’t know | 🞎 ***2 or 9 → VQ3.44*** | | | | |
| V3.43 | *Record from where did the baby bleed:* |  | | | | | |
| V3.44 | During the illness that led to death, did s/he have more frequent loose or liquid stools than usual? | 1. Yes 2. No   9. Don’t know | 🞎 ***2 or 9 → VQ3.46*** | | | | |
| V3.45 | How many stools did the baby have on the day that diarrhea/loose liquid stools were most frequent? | | **__ __** Stools  *(DK = 99)* | | | | |
| V3.46 | During the illness that led to death, did s/he vomit everything? | 1. Yes 2. No   9. Don’t know | 🞎 | | | | |
| V3.47 | During the illness that led to death, did s/he have yellow skin? | 1. Yes 2. No   9. Don’t know | 🞎 | | | | |
| V3.48 | During the illness that led to death, did the baby have yellow eyes? | 1. Yes 2. No   9. Don’t know | 🞎 | | | | |
| V3.49 | Did the infant appear to be healthy and then just die suddenly? | 1. Yes 2. No   9. Don’t know | 🞎 | | | | |
| S5a.8 | *Check SQ4.17 to determine if the baby was born in a health facility (codes 1-2):* | 1. Yes, born in a health facility 2. Not born in a health facility   9. Don’t know | 🞎***2 or 9 → SQ5a.10*** | | | | |
| S5a.8.1 | Did the baby leave the delivery facility alive or did s/he die in the facility? | 1. Yes, left alive 2. Died in the facility   9. Don’t know | 🞎***2 or 9 → SQ6.1*** | | | | |
| S5a.8.2 | How soon after birth did the baby leave the facility?  *[Record hours if less than 24 hours—if less than 1 hour, record ‘00’ hours; Record days if 1 day or more.]* | | **__ __** Days  *(DK = 99)*  OR | | | | |
|  |  |  | **__ __** Hours  *(DK = 99)* | | | | |
| S5a.8.3 | Was the child examined by a health worker prior to discharge? | 1. Yes 2. No   9. Don’t know | 🞎 | | | | |
| S5a.9 | Did (you / the mother) receive any counselling by a health worker prior to discharge? | 1. Yes 2. No   9. Don’t know | 🞎***2 or 9 → SQ5a.10*** | | | | |
| S5a.9.1 | What (were you / was she) counselled on?  *[Multiple answers allowed].*  *Probe:* Anything else? | 1. Breastfeeding 2. Immunization 3. Post-natal care attendance 4. Danger signs of newborn illness 5. Other (specify)   9. Don’t know | 1. □  2. □  3. □  4. □  5. □ ________________________  9. □ | | | | |
| S5a.10 | Was the baby ever seen by a health worker or nurse at home or in the community, or by a doctor or nurse at a health facility before the fatal illness began?  *[Multiple answers allowed.]*  *For each mentioned, ask:*  How many times was the baby seen by a <PROVIDER TYPE at PLACE> before the fatal illness began?  *Then ask:*  When was the baby first seen by (this / any of these) provider(s)? | 1. CHW or nurse at home/in community 2. Doctor or nurse at a health facility 3. Never seen   9. Don’t know | Seen  1. □  2. □  3. □  9. □ | Times  __ __  __ __ | | | First visit  __ __  Days old  *(<1 = 00;*  *DK = 99)* |
| S5a.11 | Before the fatal illness began, did <NAME> suffer from any of the following known conditions:  *[Read out all conditions and check “Yes,” “No” or “Don’t know” for each.]*  *If “Yes,” then ask:* Was s/he provided any treatment for this condition? | 1. Preterm birth    1. Was s/he given special nutrition?    2. Was s/he given “kangaroo care”? 2. Malformation (from the time of birth):    1. Head, neck and/or back    2. Mouth/palate    3. Heart    4. Arms and/or legs 3. Other   *(specify other)* | Suffered from  Yes No DK  1. □ 2. □ 9. □      1. □ 2. □ 9. □  1. □ 2. □ 9. □  1. □ 2. □ 9. □  1. □ 2. □ 9. □  1. □ 2. □ 9. □ | | | Treatment  Yes No DK  1. □ 2. □ 9. □  1. □ 2. □ 9. □  1. □ 2. □ 9. □  1. □ 2. □ 9. □  1. □ 2. □ 9. □  1. □ 2. □ 9. □  1. □ 2. □ 9. □ | |
|  |  |  | _____________________________ | | | | |
| ***Inst_1: STOP – If VQ1.26 = 1 (Neonatal death) →*** ***SQ6.1*** | | | | | | | |

| **SA Module 5b: Preventive care of post-neonates (FOR CHILD DEATHS 28 DAYS—59 MONTHS OLD)**  *Read:* Now I would like to ask you about the care of the child before the fatal illness began. | | | | | | | |
| --- | --- | --- | --- | --- | --- | --- | --- |
| S5b.1 | | Where (do you / does the mother) cook? | - 1. Inside the house   2. Outside the house   3. In a structure outside the house   9. Don’t know | | 🞎 | | |
| S5b.2 | | When (you / the mother) cooked, was <NAME> usually beside or carried by (you / her)? | 1. Yes 2. No   9. Don’t know | | 🞎 | | |
| S5b.3 | | *Skip SQ5b.3 in areas wo/malaria.*  Before (her / his) fatal illness began, did <NAME> sleep under an insecticide treated bednet? | 1. Yes, usually or always 2. Yes, sometimes 3. Never   9. Don’t know | | 🞎 | | |
| S5b.4 | | Did (you / the mother) or a wet nurse ever breastfeed <NAME>? | 1. Yes 2. No   9. Don’t know | | 🞎***2 or 9 → SQ5b.5*** | | |
| S5b.4.1 | | Was <NAME> being breastfed at the time (her / his) fatal illness began? | 1. Yes 2. No   9. Don’t know | | 🞎***1 or 9 → SQ5b.5*** | | |
| S5b.4.2 | | How old was <NAME> when s/he was last breastfed? | | | **__ __** Months  *(<1 = 00; DK = 99)* | | |
| S5b.5 | | At the time the fatal illness began, was <NAME> being given any other liquid, including non-human milk or formula, fruit juice, tea or water, or any solid, semisolid, or soft foods?  *[Multiple answers allowed. Probe, and record all liquids and foods given.]* | 1. Non-human milk or pre-mixed formula 2. Powdered formula mixed with a liquid 3. Juice, water and/or water-based drinks 4. ORS 5. Drops or syrups (vitamins, medicines) 6. Solid, semi-solid or soft foods 7. Nothing else, only given breast milk   9. Don’t know | | 1. □  2. □  3. □ ***SQ5b.6***  4. □  5. □  6. □  7. □  ***SQ5b.6***  9. □ | | |
| S5b.5.1 | | On most days before the illness began, how many times did <NAME> eat solid, semisolid, or soft foods other than liquids during the day or night? | | | __ __ Times  *(DK = 99)* | | |
| S5b.5.2 | | Which of the following food types did <NAME> typically eat every day?  *[Read out all options and check “Yes,” “No” or “Don’t know” for each.]* | 1. Grains, roots and tubers 2. Legumes and nuts 3. Dairy products (milk, yogurt, cheese) 4. Flesh foods (meat, fish, poultry, organs) 5. Eggs 6. Vitamin-A rich fruits and vegetables 7. Other fruits and vegetables | | Yes No DK  1. □ 2. □ 9. □  1. □ 2. □ 9. □  1. □ 2. □ 9. □  1. □ 2. □ 9. □  1. □ 2. □ 9. □  1. □ 2. □ 9. □  1. □ 2. □ 9. □ | | |
| S5b.6 | | Did <NAME> drink any liquids or semi-solid foods from a bottle with a nipple or teat? | 1. Yes 2. No   9. Don’t know | | 🞎 | | |
| S5b.7 | | Now I would like to ask about the chlid’s vaccinations. Do you have a card where <NAME>’s vaccinations are written down?  *If “Yes,” ask:* May I see it please? | 1. Yes, seen 2. Yes, but not seen 3. No card | | 🞎 ***2 or 3 → SQ5b.8*** | | |
| S5b.7.1 | | Did <NAME> receive any vaccinations that are not included on this card, including vaccinations received in a national immunization day campaign?  *If “Yes,” probe for vaccinations received but not recorded on the card.*  *[Record ‘Yes’ only if BCG, Polio 0-3, Hepatitis B1-3, DPT 1-3, PENTA 1-3, Measles or Yellow Fever vaccine(s) mentioned.]* | 1. Yes (received BCG, Polio 0-3, DPT 1-3 or PENTA 1-3, Measles, Yellow Fever and/or Hep B1-3 vaccinations that are not recorded on the card) 2. No   9. Don’t know | | 🞎 ***1 → Write ‘66’ in the corresponding day column below for each vaccination received but not recorded on the card.*** | | |
|  |  | *Copy vaccination date for each vaccine from the card. Record “99” or “9999” for partially unknown dates.*  *Write ‘88’ in ‘day’ column if card shows that a vaccination was given, but no date is recorded.*  *Do not leave any rows blank. Record “00” in the ‘day’ column for each vaccination that was not given.* | BCG  POLIO 0 (given at birth)  POLIO 1  POLIO 2  POLIO 3  Hepatitis B1  Hepatitis B2  Hepatitis B3  DPT 1  DPT 2  DPT 3  PENTA1  PENTA2  PENTA3  MEASLES  YELLOW FEVER | \|  \|  \|  \|  \|  \|  \|  \|  \| BCG \| \| --- \| --- \| --- \| --- \| --- \| --- \| --- \| --- \| --- \| \|  \|  \|  \|  \|  \|  \|  \|  \| P0 \| \|  \|  \|  \|  \|  \|  \|  \|  \| P1 \| \|  \|  \|  \|  \|  \|  \|  \|  \| P2 \| \|  \|  \|  \|  \|  \|  \|  \|  \| P3 \| \|  \|  \|  \|  \|  \|  \|  \|  \| HEP1 \| \|  \|  \|  \|  \|  \|  \|  \|  \| HEP2 \| \|  \|  \|  \|  \|  \|  \|  \|  \| HEP3 \| \|  \|  \|  \|  \|  \|  \|  \|  \| DPT1 \| \|  \|  \|  \|  \|  \|  \|  \|  \| DPT2 \| \|  \|  \|  \|  \|  \|  \|  \|  \| DPT3 \| \|  \|  \|  \|  \|  \|  \|  \|  \| PNT1 \| \|  \|  \|  \|  \|  \|  \|  \|  \| PNT2 \| \|  \|  \|  \|  \|  \|  \|  \|  \| PNT3 \| \|  \|  \|  \|  \|  \|  \|  \|  \| MSL \| \|  \|  \|  \|  \|  \|  \|  \|  \| YF \|   Day Month Year | | | |
| S5b.8 | | Did <NAME> ever receive any vaccinations to prevent her/him from getting diseases, including vaccinations received in a national immunization day campaign? | 1. Yes 2. No   9. Don’t know | | 🞎 ***2 or 9 → SQ5b.10*** | | |
|  |  | Please tell me if <NAME> received any of the following vaccinations: |  | | | | |
|  | .1 | A BCG vaccination against tuberculosis, that is, an injection in the arm or shoulder that usually causes a scar? | 1. Yes 2. No   9. Don’t know | | 🞎 | | |
|  | .2 | Polio vaccine, that is, drops in the mouth? | 1. Yes 2. No   9. Don’t know | | 🞎***2 or 9 → SQ5b.8.5*** | | |
|  | .3 | When was the first polio vaccine received, just after birth or later? | 1. Just after birth 2. Later   9. Don’t know | | 🞎 | | |
|  | .4 | How many times was the polio vaccine received? | | | **__ __** Times  *(DK = 99)* | | |
|  | .5 | A Hep B vaccination, that is, an injection in the right thigh, sometimes given just after birth or at the same time as polio drops? | 1. Yes 2. No   9. Don’t know | | 🞎***2 or 9 → SQ5b.8.7*** | | |
|  | .6 | How many times was a Hep B vaccination received? | | | **__ __** Times  *(DK = 99)* | | |
|  | .7 | A DPT vaccination, that is, an injection in the thighs or buttocks, sometimes given at the same time as polio drops or a Hep B vaccination? | 1. Yes 2. No   9. Don’t know | | 🞎***2 or 9 → SQ5b.8.9*** | | |
|  | .8 | How many times was a DPT vaccination received? | | | **__ __** Times  *(DK = 99)* | | |
|  | .9 | A PENTA vaccination, that is, an injection in the thighs or buttocks instead of a Hep B or DPT vaccination, sometimes given at the same time as polio drops? | 1. Yes 2. No   9. Don’t know | | 🞎***2 or 9 → SQ5b.8.11*** | | |
|  | .10 | How many times was a PENTA vaccination received? | | | **__ __** Times  *(DK = 99)* | | |
|  | .11 | A measles or MMR injection, that is, a shot in the arm at the age of 9 months or older, to prevent measles? | 1. Yes 2. No   9. Don’t know | | 🞎 | | |
|  | .12 | A yellow fever vaccination, that is, an injection given in the arm after the child is 9 months old? | 1. Yes 2. No   9. Don’t know | | 🞎 | | |
| S5b.9 | | Were any of the vaccinations <NAME> received given as part of a national immunization day campaign? | 1. Yes 2. No   9. Don’t know | | 🞎***2 or 9 → SQ5b.10*** | | |
| S5b.9.1 | | At which national immunization day campaigns did <NAME> receive vaccinations? | 1. National immunization campaign | | 1. □ | | |
| S5b.10 | | (Before / In the six months before) the fatal illness, did <NAME> receive one or more vitamin A doses like this?  *[Read “Before…” if the child lived less than 6 months.]*  *[Show ampoule/capsule/syrup]* | 1. Yes, 1 dose 2. Yes, 2 or more doses 3. No   9. Don’t know | | 🞎 | | |
| S5b.11 | | Before the fatal illness began, did <NAME> suffer from any of the following known conditions:  *[Read out all conditions and check “Yes,” “No” or “Don’t know” for each.]*  *If “Yes,” then ask:* Was s/he provided any treatment for this condition? | 1. Low height or weight (malnutrition) 2. Malformation (from the time of birth):    1. Head, neck and/or back    2. Mouth/palate    3. Heart    4. Arms and/or legs 3. Asthma 4. Heart disease 5. Tuberculosis 6. Epilepsy/convulsion 7. Other   *(specify other)* | | Suffered from  Yes No DK  1. □ 2. □ 9. □    1. □ 2. □ 9. □  1. □ 2. □ 9. □  1. □ 2. □ 9. □  1. □ 2. □ 9. □  1. □ 2. □ 9. □  1. □ 2. □ 9. □  1. □ 2. □ 9. □  1. □ 2. □ 9. □  1. □ 2. □ 9. □ | Treatment  Yes No DK  1. □ 2. □ 9. □    1. □ 2. □ 9. □  1. □ 2. □ 9. □  1. □ 2. □ 9. □  1. □ 2. □ 9. □  1. □ 2. □ 9. □  1. □ 2. □ 9. □  1. □ 2. □ 9. □  1. □ 2. □ 9. □  1. □ 2. □ 9. □ | |
|  |  |  |  |  | ___________________________ | | |
| **VA Section 4: Infant and child deaths (FOR CHILD DEATHS 28 DAYS—59 MONTHS OLD)**  *Read:* Now I’d like to ask you about <NAME>’s illness. | | | | | | | |
| V4.1 | | During the illness that led to death, did the <NAME> have a fever? | 1. Yes 2. No   9. Don’t know | | 🞎 ***2 or 9 → VQ4.6*** | | |
| V4.2 | | How many days did the fever last?  *[Less than 24 hours = “00” days]* | | | **__ __** Days  *(DK = 99)* | | |
| V4.3 | | Did the fever continue until death? | 1. Yes 2. No   9. Don’t know | | 🞎 ***2 or 9 → VQ4.6*** | | |
| V4.4 | | How severe was the fever? | 1. Mild 2. Moderate 3. Severe   9. Don’t know | | 🞎 | | |
| V4.5 | | What was the pattern of the fever? | 1. Continuous 2. On and off 3. Only at night   9. Don’t know | | 🞎 | | |
| V4.6 | | During the illness that led to death, did <NAME> have more frequent loose or liquid stools than usual? | 1. Yes 2. No   9. Don’t know | | 🞎 ***2 or 9 → VQ4.12*** | | |
| V4.7 | | How many stools did <NAME> have on the day that loose liquid stools were most frequent? | | | **__ __** Stools  *(DK = 99)* | | |
| V4.8 | | How many days before death did the frequent loose or liquid stools start?  *[Less than 24 hours = “00” days]* | | | **__ __** Days  *(DK = 99)* | | |
| V4.9 | | Did the frequent loose or liquid stools continue until death? | 1. Yes 2. No   9. Don’t know | | 🞎 ***1 or 9 → VQ4.11*** | | |
| V4.10 | | How many days before death did the loose or liquid stools stop?  *[Less than 24 hours = “00” days]* | | | **__ __** Days  *(DK = 99)* | | |
| V4.11 | | Was there visible blood in the loose or liquid stools? | 1. Yes 2. No   9. Don’t know | | 🞎 | | |
| V4.12 | | During the illness that led to death, did the child have a cough? | 1. Yes 2. No   9. Don’t know | | 🞎 ***2 or 9 → VQ4.16*** | | |
| V4.13 | | For how many days did the cough last?  *[Less than 24 hours = “00” days]* | | | **__ __** Days  *(DK = 99)* | | |
| V4.14 | | Was the cough very severe? | 1. Yes 2. No   9. Don’t know | | 🞎 | | |
| V4.15 | | Did the child vomit after s/he coughed? | 1. Yes 2. No   9. Don’t know | | 🞎 | | |
| V4.16 | | During the illness that led to death, did <NAME> have difficult breathing? | 1. Yes 2. No   9. Don’t know | | 🞎 ***2 or 9 → VQ4.18*** | | |
| V4.17 | | For how many days did the difficult breathing last?  *[Less than 24 hours = “00” days]* | | | **__ __** Days  *(DK = 99)* | | |
| V4.18 | | During the illness that led to death, did <NAME> have fast breathing? | 1. Yes 2. No   9. Don’t know | | 🞎 ***2 or 9 → VQ4.20*** | | |
| ***Inst_1: If both VQ4.16 and VQ4.18 = 2 or 9 → VQ4.25*** | | | | | | | |
| V4.19 | | For how many days did the fast breathing last?  *[Less than 24 hours = “00” days]* | | | **__ __** Days  *(DK = 99)* | | |
| V4.20 | | During the illness that led to death, did s/he have indrawing of the chest? | 1. Yes 2. No   9. Don’t know | | 🞎 | | |
| V4.21 | | During the illness that led to death, did her/his breathing sound like any of the following?  *[Demonstrate each sound]* |  | |  | | |
| V4.22 | | Stridor | 1. Yes 2. No   9. Don’t know | | 🞎 | | |
| V4.23 | | Grunting | 1. Yes 2. No   9. Don’t know | | 🞎 | | |
| V4.24 | | Wheezing | 1. Yes 2. No   9. Don’t know | | 🞎 | | |
| V4.25 | | Did <NAME> experience any generalized convulsions or fits during the illness that led to death? | 1. Yes 2. No   9. Don’t know | | 🞎 | | |
| V4.26 | | Was <NAME> unconscious during the illness that led to death? | 1. Yes 2. No   9. Don’t know | | 🞎 ***2 or 9 → VQ4.28*** | | |
| V4.27 | | How long before death did unconsciousness start? | 1. Less than 6 hours 2. 6-23 hours 3. 24 hours or more   9. Don’t know | | 🞎 | | |
| V4.28 | | Did <NAME> have a stiff neck during the illness that led to death?  *[Demonstrate]* | 1. Yes 2. No   9. Don’t know | | 🞎 | | |
| V4.29 | | Did <NAME> have a bulging fontanelle during the illness that led to death?  *[Show photo]* | 1. Yes 2. No   9. Don’t know | | 🞎 | | |
| V4.30 | | During the month before s/he died, did <NAME> have a skin rash? | 1. Yes 2. No   9. Don’t know | | 🞎 ***2 or 9 → VQ4.35*** | | |
| V4.31 | | Where was the rash? | 1. Face 2. Trunk/Abdomen 3. Extremities 4. Everywhere   9. Don’t know | | 🞎 | | |
| V4.32 | | Where did the rash start? | 1. Face 2. Trunk/Abdomen 3. Extremities 4. Everywhere   9. Don’t know | | 🞎 | | |
| V4.33 | | How many days did the rash last? | | | **__ __** Days  *(DK = 99)* | | |
| V4.34 | | Did the rash have blisters containing clear fluid? | 1. Yes 2. No   9. Don’t know | | 🞎 | | |
| V4.35 | | During the illness that led to death, did <NAME>’s limbs (legs, arms) become very thin?  *[Show photo]* | 1. Yes 2. No   9. Don’t know | | 🞎 | | |
| V4.36 | | During the illness that led to death, did <NAME> have swollen legs or feet? | 1. Yes 2. No   9. Don’t know | | 🞎 ***2 or 9 → VQ4.38*** | | |
| V4.37 | | How long did the swelling last?  *[Record days or weeks.]* | | | **__ __** Days  *(DK = 99)* | | |
|  |  |  |  |  | **__ __** Weeks  *(DK = 99)* | | |
| V4.38 | | During the illness that led to death, did <NAME>’s skin flake off in patches? | 1. Yes 2. No   9. Don’t know | | 🞎 | | |
| V4.39 | | Did <NAME>’s hair change in color to a reddish or yellowish color? | 1. Yes 2. No   9. Don’t know | | 🞎 | | |
| V4.40 | | Did <NAME> have a protruding belly? | 1. Yes 2. No   9. Don’t know | | 🞎 | | |
| V4.41 | | During the illness that led to death, did <NAME> suffer from “lack of blood” or “pallor”? | 1. Yes 2. No   9. Don’t know | | 🞎 | | |
| V4.42 | | During the illness that led to death, did <NAME> have swelling in the armpits? | 1. Yes 2. No   9. Don’t know | | 🞎 | | |
| V4.43 | | During the illness that led to death, did <NAME> have a whitish rash inside the mouth or on the tongue? | 1. Yes 2. No   9. Don’t know | | 🞎 | | |
| V4.44 | | During the illness that led to death, did <NAME> bleed from anywhere? | 1. Yes 2. No   9. Don’t know | | 🞎 ***2 or 9 → VQ4.46*** | | |
| V4.45 | | *Record from where s/he bled:* |  | | | | |
| V4.46 | | During the illness that led to death, did s/he have areas of the skin that turned black? | 1. Yes 2. No   8. Don’t know | | 🞎 | | |
| V4.47 | | Did <NAME> suffer from an injury or accident such as…?  *[Ask the respondent each in sequence and mark each as “Yes,” “No” or “Don’t know.”]* | 1. a road traffic crash/injury? 2. a fall? 3. drowning? 4. poisoning?   Did s/he suffer:   1. a bite or sting by a venomous animal? 2. a burn? 3. from violence (homicide, abuse)? 4. any other injury?   *(If “Yes,” then specify)* | | Yes No DK  1. □ 2. □ 9. □  1. □ 2. □ 9. □  1. □ 2. □ 9. □  1. □ 2. □ 9. □  1. □ 2. □ 9. □  1. □ 2. □ 9. □  1. □ 2. □ 9. □  1. □ 2. □ 9. □ | | ***All = 2 or 9***  ***→ SQ6.1*** |
|  |  |  |  |  | ___________________________ | | |
| V4.48 | | Was the injury or accident intentionally inflicted by someone else? | 1. Yes 2. No   8. Don’t know | | 🞎 | | |
| V4.49 | | How long did <NAME> survive after the injury or accident?  *[Record hours if less than 24 hours—Less than 1 hour = “00” hours;*  *Record days if 1 day or more.]* | | | **__ __** Hours  *(DK = 99)* | | |
|  |  |  |  |  | **__ __** Days  *(DK = 99)* | | |

| **SA Module 6: Care-seeking for the child’s fatal illness (FOR NN & CHILD DEATHS 0—59 MONTHS OLD)**  ***Read:*** Now, I’d like to ask you about <NAME>’s fatal illness and the care and treatments that s/he received. | | | | | | | | | | | | | | | | | |
| --- | --- | --- | --- | --- | --- | --- | --- | --- | --- | --- | --- | --- | --- | --- | --- | --- | --- |
| S6.1 | Who first noticed that <NAME> was ill? | | | | | | | 1. The respondent 2. Other relative, neighbor, friend 3. CHW or nurse at home or in community 4. Doctor or nurse at a health facility 5. Other *(specify)* | | | | | | | 🞎  ___________________________ | | |
| S6.2 | Earlier you said that <NAME> had <SYMPTOM(S)> during her/his illness.  *[Read back all the child’s symptoms from VA section 3 (for neonates) or VA section 4 (for children).]*  How did <SQ6.1 PERSON> first know that <NAME> was ill? Which of these symptoms did s/he have at that time?  What symptoms did s/he have next? On what day of the illness did these symptoms start?  *[Probe until all the symptoms are recorded in the order they appeared.]* | | | | | | | **Symptoms in order of appearance** | | | | | | | **Illness day the symptom started** | | |
|  |  |  |  |  |  |  |  | 1. | | | | | | |  | | |
|  |  |  |  |  |  |  |  | 2. | | | | | | |  | | |
|  |  |  |  |  |  |  |  | 3. | | | | | | |  | | |
|  |  |  |  |  |  |  |  | 4. | | | | | | |  | | |
|  |  |  |  |  |  |  |  | 5. | | | | | | |  | | |
|  |  |  |  |  |  |  |  | 6. | | | | | | |  | | |
|  |  |  |  |  |  |  |  | 7. | | | | | | |  | | |
| S6.3 | When <SQ6.1 PERSON> first noticed that <NAME> was ill, was s/he…  *[Read the choices for each condition.]* | | | | | | | 1. Feeding normally, poorly, or not at all 2. Alert, drowsy, or unconscious 3. Normally active, less active than normal, or not moving | | | | | | | Normal Medium Abnormal DK  1. □ 2. □ 3. □ 9. □  1. □ 2. □ 3. □ 9. □  1. □ 2. □ 3. □ 9. □ | | |
| S6.4 | Did <NAME> receive, or did you seek or try to seek, any care or treatment for the fatal illness? | | | | | | | 1. Yes 2. No—care not needed, given or sought 3. No— died immediately   9. Don’t know | | | | | | | 🞎***2 → SQ6.6***  ***3 or 9 → VQ5.10*** | | |
| S6.5 | Please tell me everything you did for <NAME>’s fatal illness inside the home and all the places outside the home you took or tried to take (her / him) for health care. Start with the first care or treatment <NAME> received and then, in order, tell me all the other care and treatments s/he received. Also tell me when and for what symptoms you took each action.  *[Include any provider <NAME> did not reach because s/he died before leaving home or on route.]*  *(1) Check one other care or health provider box for each action row. (2) For neonatal deaths only: If the illness began at the health provider where the child was delivered, then mark that as Action 1 and check the “illness began at provider” box. (3) Record the illness day each action was taken. (4) Ensure no action was taken for a symptom before it started (in SQ6.2).* | | | | | | | | | | | | | | | | |
| **Action #** | **(1)**  **Other care** | | | **(1)**  **Health Providers** | | | | | | | | | **(3)**  **Illness day the action was taken** | | | **(4)**  **For what symptom(s) was the action taken?** | |
|  | **Home care (own, relative, neigh-bor, friend)** | **Tradi-tional or non-formal provider** | **Phar-macist or drug seller** | **Trained CH**  **Worker, nurse, or midwife** | | **Private doctor**  **(formal/unsure)** | | | **NGO or govt. clinic** | **Hospital** | **(2)**  **Illness began at provider where child was delivered** | |  |  |  |  |  |
| 1. | 🞎 | 🞎 | 🞎 | 🞎 | | 🞎 | | | 🞎 | 🞎 | 🞎 | | **__ __**  *(DK = 99)* | | |  | |
| 2. | 🞎 | 🞎 | 🞎 | 🞎 | | 🞎 | | | 🞎 | 🞎 |  | | **__ __**  *(DK = 99)* | | |  | |
| 3. | 🞎 | 🞎 | 🞎 | 🞎 | | 🞎 | | | 🞎 | 🞎 |  | | **__ __**  *(DK = 99)* | | |  | |
| 4. | 🞎 | 🞎 | 🞎 | 🞎 | | 🞎 | | | 🞎 | 🞎 |  | | **__ __**  *(DK = 99)* | | |  | |
| 5. | 🞎 | 🞎 | 🞎 | 🞎 | | 🞎 | | | 🞎 | 🞎 |  | | **__ __**  *(DK = 99)* | | |  | |
| 6. | 🞎 | 🞎 | 🞎 | 🞎 | | 🞎 | | | 🞎 | 🞎 |  | | **__ __**  *(DK = 99)* | | |  | |
| 7. | 🞎 | 🞎 | 🞎 | 🞎 | | 🞎 | | | 🞎 | 🞎 |  | | **__ __**  *(DK = 99)* | | |  | |
| ***Inst_1: (For neonatal deaths only) If illness began at health provider where child was delivered:***  ***And did not fill L&D matrix (module 4) → SQ6.10; And filled L&D matrix (module 4) → SQ6.16*** | | | | | | | | | | | | | | | | | |
| S6.6 | *If no care given or sought, ask:* Who decided that <NAME> did not need any care or treatment for the illness?  *If any care given or sought, ask:* Who decided that <ACTION 1> was the first thing to do for <NAME>’s illness?  *[Record the one main decision maker.]* | | | | | | | 1. Child’s mother 2. Child’s father 3. Child’s aunt 4. Child’s uncle 5. Child’s grandmother 6. Child’s paternal grandfather 7. Child’s maternal grandfather 8. Other *(specify)*   9.Don’t know | | | | | | | 1. □  2. □  3. □  4. □  5. □  6. □  7. □  8. □ _______________________  9. □ | | |
| S6.7 | *If never taken to a health provider, ask:* Did you have any concerns or problems that kept you from taking <NAME> to a health provider during his/her illness?  *If taken to a health provider, ask:* Did you have to overcome any concerns or problems to take <NAME> to the (first) health provider? | | | | | | | 1. Yes 2. No   9. Don’t know | | | | | | | 🞎 ***2 or 9 → Inst_2*** | | |
| S6.7.1 | What concerns or problems did you have?  *Prompt:* Was there anything else?  *[Multiple answers allowed.]* | | | | | | | 1. Did not think child was sick enough to need health care 2. No one available to go with caregiver 3. Too much time from her regular duties 4. Someone else *(specify)* had to decide 5. Too far to travel 6. No transportation available 7. Cost (transport, health care, other) 8. Not satisfied with available health care 9. Problem required traditional care 10. Thought child was too sick to travel 11. Thought child will die no matter what 12. Was late at night (transportation or provider not available) 13. Other *(specify)*   99.Don’t know | | | | | | | 1. □  2. □  3. □  4. □ _______________________  5. □  6. □  7. □  8. □  9. □  10. □  11. □  12. □  13. □ ______________________  99. □ | | |
| ***Inst_2: If SQ6.4 = 2 (No care given) or***  ***If SQ6.5 ≠ “Health Provider” (Never took and never tried to take to a health provider) → SQ6.39*** | | | | | | | | | | | | | | | | | |
| S6.8 | *Refer to SQ6.5 for the first health provider and related symptoms:*  You mentioned that you took <NAME> to the (first) health provider, I mean the <FIRST HEALTH PROVIDER> for <SYMPTOM(S)>. How long had <NAME> had (this / these) symptom(s) when it was decided to take him/her to the <FIRST HEALTH PROVIDER>?  *[Read “…to the first…” if took or tried to take to more than one health provider.]*  *[Mark days, hours &/or minutes as needed: e.g. 00 day, 02 hours, 10 minutes]* | | | | | | | | | | | | | | **__ __** Days  *(DK = 99)* | | |
|  |  |  |  |  |  |  |  |  |  |  |  |  |  |  | **__ __** Hours  *(DK = 99)* | | |
|  |  |  |  |  |  |  |  |  |  |  |  |  |  |  | **__ __** Minutes  *(DK = 99)* | | |
| ***Child illness matrix instructions:*** *Ask the following questions for the first and last health providers where care was sought or tried to be sought for the fatal illness. Ask all the questions for the first provider before going on to the last.*  *Before asking about the first health provider, read:*  Now I would like to ask you about your visit to the (first) health provider. *[Read “first” if went or tried to go to more than one provider.]*  *Before asking about the last health provider, read:*  Now I would like to ask you about your visit to the last health provider, I mean the <LAST HEALTH PROVIDER>. | | | | | | | | | | | | | | | | | |
| **– CHILD ILLNESS MATRIX QUESTIONS –** | | | | | | | | | | | | **FIRST HEALTH PROVIDER** | | | | | **LAST HEALTH PROVIDER** |
| At the time when it was decided to take <NAME> to the <FIRST/LAST HEALTH PROVIDER>, was s/he…  *[Read the choices for each condition.]* | | | | | 1. Feeding normally, poorly, or not at all 2. Alert, drowsy, or unconscious 3. Normally active, less active than normal, or not moving | | | | | | | S6.9  Nrml Med Abnrm DK  1. □ 2. □ 3. □ 9. □  1. □ 2. □ 3. □ 9. □  1. □ 2. □ 3. □ 9. □ | | | | | S6.24  Nrml Med Abnrm DK  1. □ 2. □ 3. □ 9. □  1. □ 2. □ 3. □ 9. □  1. □ 2. □ 3. □ 9. □ |
| What was the name of the <FIRST/LAST HEALTH PROVIDER> where you took <NAME>?  *Probe to identify the type of provider.* | | | | | 1. Hospital (Government) 2. Hospital (NGO) 3. Hospital (Private) 4. Health center (Government) 5. Health center (NGO) 6. Health post (Government) 7. Health post (NGO) 8. Private doctor/clinic (Formal) 9. Private doctor/clinic (?Formal?) 10. Trained community health worker, nurse, or midwife   99. Don’t know | | | | | | | S6.10  🞎🞎  ___________________  (Name of Provider or Facility) | | | | | S6.25  🞎🞎  ___________________  (Name of Provider or Facility) |
| After (deciding to seek care / being referred), how much time passed before going to the <FIRST/LAST HEALTH PROVIDER>?  *[Discuss that this might include the time needed to arrange for transportation and money to go to the provider/facility, or to provide home care or go to a traditional provider before going to the health provider.]*  *[If the child died at home, record the time from decision/referral to death.]*  *[Mark days, hours &/or minutes as needed: e.g. 01 day, 05 hours, 30 minutes]* | | | | | | | | | | | | S6.11  **__ __** Days  *(DK = 99)* | | | | | S6.26  **__ __** Days  *(DK = 99)* |
|  |  |  |  |  |  |  |  |  |  |  |  | **__ __** Hours  *(DK = 99)* | | | | | **__ __** Hours  *(DK = 99)* |
|  |  |  |  |  |  |  |  |  |  |  |  | **__ __** Minutes  *(DK = 99)* | | | | | **__ __** Minutes  *(DK = 99)* |
| Was there any cost to travel to the <FIRST/LAST HEALTH PROVIDER> or pay for the child’s care there? | | | | | 1. Yes 2. No   9. Don’t know | | | | | | | S6.12  🞎 ***2 or 9 → SQ6.13*** | | | | | S6.27  🞎 ***2 or 9 → SQ6.28*** |
| How did you arrange for the money for these expenses?  *[Multiple answers allowed.]* | | | | | 1. Had available 2. Borrowed 3. Sold assets 4. Help from kin/relatives 5. Community fund 6. Govt. scheme 7. Other   9. Don’t know | | | | | | | S6.12.1  1. □  2. □  3. □  4. □  5. □  6. □  7. □  9. □ | | | | | S6.27.1  1. □  2. □  3. □  4. □  5. □  6. □  7. □  9. □ |
| What transportation method was used to go there?  *[Multiple answers allowed.]* | | | | | 1. Walk 2. Bicycle/animal/cart/ boat 3. Bus 4. Taxi/auto/trecker/motorcycle 5. Ambulance 6. Other 7. Could not arrange transport   9. Don’t know | | | | | | | S6.13  1. □ ***If only walk***  2. □ ***→ SQ6.14.1***  3. □  4. □  5. □  6. □  7. □ ***→ SQ6.14.1***  9. □ | | | | | S6.28  1. □ ***If only walk***  2. □ ***→ SQ6.29.1***  3. □  4. □  5. □  6. □  7. □ ***→ SQ6.29.1***  9. □ |
| How much did the transportation cost? | | | | | | | | | | | | S6.14  **__ __ __ __** unit  *(DK=9999)* | | | | | S6.29  **__ __ __ __** unit  *(DK = 9999)* |
| Did the child reach the <FIRST/LAST HEALTH PROVIDER> before s/he died?  *[If “No,” discuss with respondent to determine correct response: 2, 3 or 4.]* | | | | | 1. Yes, reached before child died 2. No, died before setting out 3. No, died on route to this provider 4. No, could not reach this provider – did not set out/returned home/took other action   9. Don’t know | | | | | | | S6.14.1  🞎 ***2, 3 → SQ6.39***  ***4, 9 → Inst_4*** | | | | | S6.29.1  🞎 ***2-9 → SQ6.39*** |
| How long did it take to travel to the <FIRST/LAST HEALTH PROVIDER>?  *[Mark hours &/or minutes as needed: e.g. 02 hours, 10 minutes]* | | | | | | | | | | | | S6.15  **__ __** Hours  *(DK = 99)* | | | | | S6.30  **__ __** Hours  *(DK = 99)* |
|  |  |  |  |  |  |  |  |  |  |  |  | **__ __** Minutes  *(DK = 99)* | | | | | **__ __** Minutes  *(DK = 99)* |
| What did the <FIRST/LAST HEALTH PROVIDER> do for <NAME>’s problem?  *Prompt:* Was there anything else?  *[Multiple answers allowed.]* | | | | | 1. Gave oxygen 2. Helped breathe with bag or mask 3. Gave fluids by mouth 4. Gave antibiotics by mouth 5. Gave antimalarial by mouth 6. Gave ORS 7. Gave Vitamin A 8. Gave other medicine by mouth 9. Gave IM medicine 10. Gave IV fluids or medicine 11. Advised to buy outside medicine 12. Did an operation *(specify)* 13. Admitted to hospital 14. Other *(specify)* 15. Nothing   99.Don’t know | | | | | | | S6.16  1. □  2. □  3. □  4. □  5. □  6. □  7. □  8. □ _______________  9. □  10. □  11. □  12. □ ______________  13. □ stayed __ __ days  14. □ ______________  15. □ ***→ SQ6.18***  99. □ ***→ SQ6.18*** | | | | | S6.31  1. □  2. □  3. □  4. □  5. □  6. □  7. □  8. □ _______________  9. □  10. □  11. □  12. □ ______________  13. □ stayed __ __ days  14. □ ______________  15. □ ***→ SQ6.33***  99. □ ***→ SQ6.33*** |
| How much did you pay for these treatments and other costs related to the health care, including the admission fee, consultation, lab tests, equipment, and room and food for companions? | | | | | | | | | | | | S6.17  **__ __ __ __ __** unit  *(DK = 99999)* | | | | | S6.32  **__ __ __ __ __** unit  *(DK = 99999)* |
| Did the <FIRST/LAST HEALTH PROVIDER> refer <NAME> to another health provider or facility? | | | | | 1. Yes 2. No   9. Don’t know | | | | | | | S6.18  🞎 ***2 or 9 → SQ6.19*** | | | | | S6.33  🞎 ***2 or 9 → SQ6.34*** |
| Why was <NAME> referred?  *[Multiple answers allowed.]* | | | | | 1. The provider was not capable of managing the problem 2. Required supplies (e.g., drugs, IV, oxygen) not available 3. Required equipment (e.g., xray machine) not available   9. Don’t know | | | | | | | S6.18.1  1. □  2. □  3. □  9. □ | | | | | S6.33.1  1. □  2. □  3. □  9. □ |
| Did <NAME> leave the <FIRST/LAST HEALTH PROVIDER> alive? | | | | | 1. Yes, left alive 2. No, died at this provider | | | | | | | S6.19  🞎 ***2 → VQ5.4*** | | | | | S6.34  🞎 ***2 → VQ5.4*** |
| Did the <FIRST/LAST HEALTH PROVIDER> suggest that you do anything for <NAME>’s illness after leaving? | | | | | 1. Yes 2. No   9. Don’t know | | | | | | | S6.20  🞎 ***2 or 9 → SQ6.22*** | | | | | S6.35  🞎 ***2 or 9 → SQ6.37*** |
| What did the <FIRST/LAST HEALTH PROVIDER> suggest that you do?  *Prompt:* Was there anything else?  *[Multiple answers allowed.]* | | | | | 1. Increase breastfeeding 2. Give extra fluids 3. Continue feeding 4. Give ORS 5. Give antibiotic by mouth 6. Give antimalarial by mouth 7. Give vitamin A by mouth 8. Return for follow-up visit 9. Return or referred if worse 10. Complete the present referral 11. Other *(specify)*   99. Don’t know | | | | | | | S6.20.1  1. □  2. □  3. □  4. □  5. □  6. □  7. □  8. □  9. □  10. □  11. □ ______________  99. □ ***→ SQ6.22*** | | | | | S6.35.1  1. □  2. □  3. □  4. □  5. □  6. □  7. □  8. □  9. □  10. □  11. □ ______________  99. □ ***→ SQ6.37*** |
| Were you able to follow all the advice? | | | | | 1. Yes 2. No   9. Don’t know | | | | | | | S6.21  🞎 ***9 → SQ6.22*** | | | | | S6.36  🞎 ***9 → SQ6.37*** |
| *If not able to follow all the advice, ask:* Did you have any concerns or problems that kept you from following the advice?  *If able to follow all the advice, ask:* Did you have to overcome any concerns or problems to follow the advice? | | | | | 1. Yes 2. No   9. Don’t know | | | | | | | S6.21.1  🞎 ***2 or 9 → SQ6.22*** | | | | | S6.36.1  🞎 ***2 or 9 → SQ6.37*** |
| What concerns or problems did you have?  *Prompt:* Was there anything else?  *[Multiple answers allowed.]* | | | | | 1. Did not understand instructions 2. Too much time from regular duties 3. Someone else *(specify)* decided 4. Cost too much 5. Problem required traditional care 6. Thought adivised care not needed 7. Thought care might harm the child 8. Thought child will die despite care 9. No time before go to next provider 10. The child died too soon 11. Other *(specify)*   99.Don’t know | | | | | | | S6.21.2  1. □  2. □  3. □ _______________  4. □  5. □  6. □  7. □  8. □  9. □  10. □  11. □ ______________  99. □ | | | | | S6.36.2  1. □  2. □  3. □ _______________  4. □  5. □  6. □  7. □  8. □  9. □  10. □  11. □ ______________  99. □ |
| At the time of leaving the (<FIRST HEALTH PROVIDER> / <LAST HEALTH PROVIDER>), was <NAME>…  *[Read the choices for each condition.]* | | | | | 1. Feeding normally, poorly, or   not at all   1. Alert, drowsy, or unconscious 2. Normally active, less active than normal, or not moving | | | | | | | S6.22  Nrml Med Abnrm DK  1. □ 2. □ 3. □ 9. □  1. □ 2. □ 3. □ 9. □  1. □ 2. □ 3. □ 9. □ | | | | | S6.37  Nrml Med Abnrm DK  1. □ 2. □ 3. □ 9. □  1. □ 2. □ 3. □ 9. □  1. □ 2. □ 3. □ 9. □ |
| *If not taken to another health provider, ask:* Did you have any concerns or problems that kept you from taking <NAME> to another health provider?  *If taken to another health provider, ask:* Did you have to overcome any concerns or problems to take <NAME> to another health provider? | | | | | 1. Yes 2. No   9. Don’t know | | | | | | | S6.23  🞎 ***2 or 9 → Inst_4*** | | | | | S6.38  🞎 ***2 or 9 → SQ6.39*** |
| What concerns or problems did you have?  *Prompt:* Was there anything else?  *[Multiple answers allowed.]* | | | | | 1. Thought no more care needed 2. No one available to go with her 3. Too much time from regular duties 4. Someone else *(specify)* decided 5. Too far to travel 6. No transportation available 7. Cost (transport, health care, other) 8. Not satisfied with available care 9. Problem required traditional care 10. Thought child too sick to travel 11. Thought child will die despite care 12. Was late at night 13. The child died before going 14. Other *(specify)*   99.Don’t know | | | | | | | S6.23.1  1. □  2. □  3. □  4. □ _______________  5. □  6. □  7. □  8. □  9. □  10. □  11. □  12. □  13. □ ***→ SQ6.39***  14. □ ______________  99. □ | | | | | S6.38.1  1. □  2. □  3. □  4. □ _______________  5. □  6. □  7. □  8. □  9. □  10. □  11. □  12. □  13. □  14. □ ______________  99. □ |
| ***Inst_4: Check SQ6.5 → If taken to another health provider…*** | | | | | | | | | | | | ***…go to SQ6.24***  ***(LAST PROVIDER)*** | | | | |  |
| S6.39 | How many days after (first noticing the illness / <LAST ACTION SQ6.5> / leaving the first/last health provider) did <NAME> die?  *[If SQ6.4 = 2 (No care given), then read: “…first noticing the illness…”]* | | | | | | | | | | | | | **__ __** Days  *(<1 = 00; DK = 99)* | | | |
| ***Inst_5: If SQ6.4 = 2 (No care given) or***  ***if SQ6.5 ≠ “Health Provider” (Never took and never tried to take to a health provider) → VQ5.10*** | | | | | | | | | | | | | | | | | |
| **VA Section 5: Health records (FOR STILLBIRTHS, NEONATAL & CHILD DEATHS 0—59 MONTHS OLD)** | | | | | | | | | | | | | | | | | |
| V5.4 | Do you have any health records that belonged to the deceased? | | | | | | 1. Yes 2. No   9. Don’t know | | | | | | | | 🞎 ***2 or 9 → VQ5.10*** | | |
| V5.5 | Can I see the health records? | | | | | | 1. Yes 2. No | | | | | | | | 🞎 ***2 → VQ5.10*** | | |
| V5.6 | *Record the dates of the two most recent visits* | | | | | | | | | | | | | | **__ __/__ __/__ __ __ __**  D D M M Y Y Y Y  *(DK = 99/99/9999)* | | |
|  |  |  |  |  |  |  |  |  |  |  |  |  |  |  | **__ __/__ __/__ __ __ __**  D D M M Y Y Y Y  *(DK = 99/99/9999)* | | |
| V5.7 | *Record the two most recent weights on those dates* | | | | | | | | | | | | | | **__ __ __ __** Grams  *(DK = 9999)* | | |
|  |  |  |  |  |  |  |  |  |  |  |  |  |  |  | **__ __ __ __** Grams  *(DK = 9999)* | | |
| V5.8 | *Record the date of the last note* | | | | | | | | | | | | | | **__ __/__ __/__ __ __ __**  D D M M Y Y Y Y  *(DK = 99/99/9999)* | | |

| V5.9 | *Transcribe the note* | | | | |
| --- | --- | --- | --- | --- | --- |
|  | ________________________________________________________________________________________________  ________________________________________________________________________________________________  ________________________________________________________________________________________________  ________________________________________________________________________________________________  ________________________________________________________________________________________________  ________________________________________________________________________________________________  ________________________________________________________________________________________________ | | | | |
| V5.10 | Was a death certificate issued? | 1. Yes 2. No   9. Don’t know | | 🞎 ***2 or 9 → SQ1.1*** | |
| V5.11 | Can I see the death certificate? | 1. Yes 2. No | | 🞎 ***2 → SQ1.1*** | |
| V5.12 | *Record the immediate cause of death from the death certificate* |  | | | |
| V5.13 | *Record the first underlying cause of death from the death certificate* |  | | | |
| V5.14 | *Record the second underlying cause of death from the death certificate* |  | | | |
| V5.15 | *Record the third underlying cause of death from the death certificate* |  | | | |
| V5.16 | *Record the contributing cause of death from the death certificate* |  | | | |
| **SA Module 1: The mother and her household (FOR STILLBIRTHS, NN & CHILD DEATHS 0—59 MONTHS OLD)**  *Read:* Now I would like to ask you some other questions about (yourself / the child’s mother).  *[Read “…the child’s mother.” If the respondent is not the mother.* | | | | | |
| ***Inst_1: If GQ4.3 = 1 (Respondent is the mother) →*** ***SQ1.4*** | | | | | |
| S1.2 | How old (is the child’s mother / was the child’s mother when she died)?  *[Read “…was the child’s mother…” if she died.]* | | | __ __ Years  *(DK = 99)* | |
| S1.3 | How many years of school did the mother complete? | | | **__ __** Years  *(<1 = 00; DK = 99)* |  |
| S1.4 | (Are you / Is/Was the child’s mother)…  *[Read “…Is/Was the child’s mother…” if the respondent is not the mother.]*    *[Read the choices to the respondent.]* | 1. Married? 2. Living with a man? 3. Widowed? 4. Divorced, separated, or deserted? 5. Single (never married/lived w/a man)?   9. Don’t know | | 🞎 ***5 or 9 → Inst_2*** | |
| S1.4.1 | How old (were you when you / was she when she) first married (or lived with a man)?  *[Read “…was she when she…” if the respondent is not the mother.]*  *[Read “…married or lived with a man?” if SQ1.4 = “2. Living with a man”]* | | | __ __ Years  *(DK = 99)* | |
| S1.4.2 | How many years of school did (your / her) (husband / partner) complete?  *[Read “…her…” if the respondent is not the mother.]*  *[Read “…partner…” if she is living with a man.]* | | | **__ __** Years  *(<1 = 00; DK = 99)* | |
| ***Inst_2:*** *Read:* Now I would like to ask you some questions about (your / the mother’s) household. Please remember that all information will be kept confidential.    *[SBs & NN deaths: If the respondent is not the mother, read “…the mother’s…;” and ask SQ1.5–1.11 about the mother’s household.*  *Older deaths: Always read “…your…;” and ask SQ1.5–1.11 about the respondent’s household.]* | | | | | |
| S1.5 | Who was the main breadwinner of (your / the mother’s) family during the (last days of the pregnancy / child’s fatal illness)  *[SBs~~/~~NN deaths: Read “…last days…”;*  *Older deaths: Read “…child's…”]* | 1. Child’s father 2. Child’s mother 3. Other   9. Don’t know | | 🞎 ***9 → SQ1.7*** | |
| S1.6 | At that time, what kind of work did the main breadwinner mostly do? | 1. Farmer/agricultural worker 2. Poultry or cattle raising 3. Domestic servant 4. Home-based manufacturing 5. Unskilled laborer 6. Semi-skilled laborer/service provider 7. Factory worker, blue collar service 8. Business owner 9. Professional/technician 10. Other *(specify)* 11. Overseas worker   99. Don’t know | | 🞎🞎  ___________________________ | |
| S1.7 | Is this the house (where we are now) where (you / the mother) stayed during the (last days of the pregnancy / child’s fatal illness)?  *[SBs/NN deaths: Read “…last days…”*  *Older deaths: Read “…child's…”]*  *[Read “…where we are now…” if needed to clarify which house you are talking about.]* | 1. Yes 2. No   9. Don’t know | | 🞎 ***1 → SQ1.10***  ***9 → VQ5.17*** | |
| S1.8 | Where did (you / the mother) stay at that time?    *Probe:* Where did (you / the mother) stay during the illness events?  *[Mark “1” only if her usual residence was not her in-laws or other relatives.]* | 1. Her own home at that time (other than with her in-laws) *(Interviewer: Use this code just if she moved after the death.)* 2. Her in-law’s home 3. Her parent’s home 4. Her brother’s home 5. Other *(specify)*   9. Don’t know | | 🞎 ***9 → VQ5.17***  ___________________________ | |
| S1.9 | What is the address of the place where (you / she) stayed? | State ____________________________  LGA _____________________________ | | 🞎🞎🞎  🞎🞎🞎 | |
| S1.10 | At the time of the illness events, how long had (you / the mother / your <RELATIVES> / the mother’s <RELATIVES>) been living continuously in (this / that) community?  *[Read “…<RELATIVES…” if SQ1.8 = 2-5 (s/he stayed with her/his relatives)].* | | | __ __ Years  *(<1 = 00; DK =99)* | |
| S1.11 | How long does it take to reach the health provider or facility where (you / the mother) normally (go(es) / went) from (this / that) place?  *[Mark hours &/or minutes as needed: e.g. 01 hour, 30 minutes]* | | | **__ __** Hours  *(DK = 99)* | |
|  |  |  |  | **__ __** Minutes  *(DK = 99)* | |
| ***Inst_3 → SQ2.1.1 (if including optional Module 2) or VQ5.17*** | | | | | |
| **SA Module 2: Social capital (OPTIONAL MODULE—FOR SBs, NN & CHILD DEATHS 0–59 MONTHS OLD)**  *Read:* Now, I have some questions about (your / the mother’s / your <RELATIVES’> / the mother’s <RELATIVES’>) community.  *[SBs and NN deaths: If the respondent is not the mother, read “…the mother’s…” or “…the mothers’ <RELATIVES’>...;” and ask SQ2.1.1–SQ2.3.1 about the mother and her community or her relatives’ community.*  *Older deaths: Always read “…your…” or “…your <RELATIVES’>…;” and ask SQ2.1.1–SQ2.3.1 about the respondent and her/his community or her/his relatives’ community.*  *All deaths: Ask about the relatives’ community if s/he stayed with her/his relatives during the illness events.]* | | | | | |
| S2.1.1 | In the last 3 years, did the people in the (village / neighborhood) work together on any of the following issues that affect the entire community or part of the community?  *Read all the issues and mark (“X”) Yes, No or DK for each one; then enter the code.]* | | 1. Education/schools 2. Health services/clinics 3. Paid job opportunities 4. Credit/finance 5. Roads 6. Public transportation 7. Water distribution 8. Sanitation services 9. Agriculture 10. Justice/conflict resolution 11. Security/police services 12. Mosque/church/temple 13. Other   *(specify)* | Yes No DK  1. □ 2. □ 9. □  1. □ 2. □ 9. □  1. □ 2. □ 9. □  1. □ 2. □ 9. □  1. □ 2. □ 9. □  1. □ 2. □ 9. □  1. □ 2. □ 9. □  1. □ 2. □ 9. □  1. □ 2. □ 9. □  1. □ 2. □ 9. □  1. □ 2. □ 9. □  1. □ 2. □ 9. □  1. □ 2. □ 9. □  ___________________________ | |
|  |  |  | *Code:*   1. One or more issues identified 2. No issue identified | 🞎 | |
| S2.2 | (Were you / Was the mother) able to turn to any persons, groups or organizations in the community for help during (the pregnancy / (or) the child’s fatal illness)?  *[Read “…the pregnancy?” for SBs; or “…the pregnancy or the child’s fatal illness?” for NN deaths; or “…the child’s fatal illness for older deaths.]* | | 1. Yes 2. No   9. Don’t know | 🞎 ***2 or 9 → SQ2.3.1*** | |
| S2.2.1 | Did (you / she) turn to any of the following for help?  *[Read all the options and mark (“X”) Yes, No or DK for each; then enter the code.]* | | 1. Family 2. Neighbors 3. Friends 4. Religious leader or group 5. Community leader 6. Police 7. Patron/employer/benefactor 8. Political leader 9. Mutual support group s/he belongs to 10. Assistance organization to which s/he does not belong 11. Other   *(specify)* | Yes No DK  1. □ 2. □ 9. □  1. □ 2. □ 9. □  1. □ 2. □ 9. □  1. □ 2. □ 9. □  1. □ 2. □ 9. □  1. □ 2. □ 9. □  1. □ 2. □ 9. □  1. □ 2. □ 9. □  1. □ 2. □ 9. □  1. □ 2. □ 9. □  1. □ 2. □ 9. □  1. □ 2. □ 9. □  ___________________________ | |
|  |  |  | *Code:*   1. One person/group identified 2. Two or more persons/groups identified 3. No person/group identified | 🞎***3 → SQ2.3.1*** | |
| S2.2.2 | (Is this / Are these) the same person(s) or group(s) (you / she) would usually turn to for help with a serious problem? | | 1. Yes 2. No   9. Don’t know | 🞎 | |
| S2.3.1 | (Have you or your / Has the mother or her) family ever been denied any of the following community services?  *Read all the options and mark (“X”) Yes, No or DK for each; then enter the code.]* | | 1. Education/schools 2. Health services/clinics 3. Paid job opportunities 4. Credit/finance 5. Transportation 6. Water distribution 7. Sanitation services 8. Agricultural extension 9. Justice/conflict resolution 10. Security/police services 11. Other   *(specify)* | Yes No DK  1. □ 2. □ 9. □  1. □ 2. □ 9. □  1. □ 2. □ 9. □  1. □ 2. □ 9. □  1. □ 2. □ 9. □  1. □ 2. □ 9. □  1. □ 2. □ 9. □  1. □ 2. □ 9. □  1. □ 2. □ 9. □  1. □ 2. □ 9. □  1. □ 2. □ 9. □  ___________________________ | |
|  |  |  | *Code:*   1. One service denied 2. Two or more services denied 3. No denied service identified | 🞎 | |
| V5.165 | *Read:* Now I have four last questions about the child and the child’s mother.  Before the fatal illness began, did the child suffer from HIV/AIDS? | | 1. Yes 2. No 3. Refused to answer 4. Don’t know | 🞎 | |
| V5.17 | (Have you / Has the deceased’s biological mother) ever been tested for “HIV”? | | 1. Yes 2. No   8. Refused to answer  9. Don’t know | 🞎 ***2-9 → VQ5.19*** | |
| V5.18 | Was the “HIV” test ever positive? | | 1. Yes 2. No   8. Refused to answer  9. Don’t know | 🞎 | |
| V5.19 | (Have you / Has the deceased’s biological mother) ever been told she had “AIDS” by a health worker? | | 1. Yes 2. No   8. Refused to answer  9. Don’t know | 🞎 | |
| **VA Section 6 & SA Module 7: Open ended response & interviewer comments/observations (FOR ALL DEATHS)**  *Read:* Thank you for answering the many questions that I’ve asked. Would you like to tell me about <NAME>’s illness in your own words? Also, is there anything else about her/his illness that I did not ask and you would like to tell me about?  *After the respondent(s) finishes, ask*: Is there anything else?  *Write the respondent’s exact words. After s/he has finished, read this back and ask her to correct any errors in what you wrote.* | | | | | |
| _____________________________________________________________________________________________________  _____________________________________________________________________________________________________  _____________________________________________________________________________________________________  _____________________________________________________________________________________________________  _____________________________________________________________________________________________________  _____________________________________________________________________________________________________  _____________________________________________________________________________________________________  _____________________________________________________________________________________________________  _____________________________________________________________________________________________________  _____________________________________________________________________________________________________  _____________________________________________________________________________________________________  _____________________________________________________________________________________________________  _____________________________________________________________________________________________________  _____________________________________________________________________________________________________ | | | | | |

**END OF INTERVIEW**

**THANK RESPONDENT FOR HER/HIS PARTICIPATION**

| *Interviewer: Use this space to write down your comments and observations about the interview.* |
| --- |
| _____________________________________________________________________________________________________  _____________________________________________________________________________________________________  _____________________________________________________________________________________________________  _____________________________________________________________________________________________________  _____________________________________________________________________________________________________  _____________________________________________________________________________________________________  _____________________________________________________________________________________________________ |

**Hausa VASA questionnaire**

| **VERBAL/SOCIAL AUTOPSY GENERAL INFORMATION (FOR SBs, NN & CHILD DEATHS 0—59 MONTHS OLD)**  **Section 1: Background about the deceased**  *Interviewer: Before going to the field to do the interview, fill in this section from the survey or surveillance record for the deceased*. | | | | | | |
| --- | --- | --- | --- | --- | --- | --- |
| G1.1 | Address of the household  *[Copy the household address]* | State ____________________________  LGA ______________________  Locality _____________________  EA ________________________ | | | 🞎🞎🞎  🞎🞎🞎  🞎🞎🞎  🞎🞎🞎🞎 | |
|  | Directions to the household  *[Copy the directions to the household]* |  | | | | |
|  | Sketch a map if needed | | | | | |
| G1.2 | Name of the deceased (if known)  *[Copy the name of the deceased]* |  | | | | |
| G1.3 | Sex of deceased  *[Copy the sex of the deceased]* | 1. Male 2. Female | | | 🞎 | |
| G1.4 | Date of birth of the deceased  *[Copy the day, month and year of birth of the deceased]* | | | | **__ __/__ __/__ __ __ __**  D D M M Y Y Y Y  *(DK = 99/99/9999)* | |
| G1.5 | Date of death of the deceased  *[Copy the day, month and year of death of the deceased]* | | | | **__ __/__ __/__ __ __ __**  *(DK = 99/99/9999)* | |
| G1.6 | Last known age of the deceased  *[Copy the last known age of the deceased: Record days if less than 28 days—if less than 24 hours, record “00” days; Record months if 28 days-11 months; Record years if 1 year or older.]* | | | | **__ __** Days: ***1 or more → GQ1.7***  *(DK = 99)* | |
|  |  |  |  |  | **__ __** Months ***→ GQ1.7***  *(DK = 99)* | |
|  |  |  |  |  | **__ __** Years ***→ GQ1.7***  *(DK = 99)* | |
| G1.6.1 | Was this a stillbirth or neonatal death?  *[Copy this information from the record]* | 1. Stillbirth 2. Neonatal death   9. Not known from the record | | | 🞎 | |
| G1.7 | Name of mother  *[Copy the name of the mother]* |  | | | | |
| G1.8 | Name of father  *[Copy the name of the father]* |  | | | | |
| **Section 2: Background about the interview**  *Interviewer: Before and after the interview, fill in this section. These questions should not be asked of the respondent.* | | | | | | |
| G2.1 | Language of the interview |  | | | | |
| G2.2 | Interviewer name and ID number | _________________________________ | | | 🞎🞎🞎🞎🞎 | |
| G2.3 | Dates of attempted and successful interviews | **DATE** | | | **RESULT OF THE INTERVIEW** | |
| G2.3.1 | Date of first interview attempt | **__ __/__ __/__ __ __ __**  D D M M Y Y Y Y | | | **Interim result:**  🞎 | |
| G2.3.2 | Date of second interview attempt | **__ __/__ __/__ __ __ __**  D D M M Y Y Y Y | | | **Interim result:**  🞎 | |
| G2.3.3 | Date of third interview attempt | **__ __/__ __/__ __ __ __**  D D M M Y Y Y Y | | | **Interim result:**  🞎 | |
| G2.4 | Date interview started  *[Equals date of the last attempt]* | **__ __/__ __/__ __ __ __**  D D M M Y Y Y Y | | |  | |
| G2.5 | Time interview started  *[Record hour 1-24 / minutes 1-60]* | **__ __/__ __**  H R M M | | |  | |
| G2.6 | Date interview finished  *[Equals date started or a later date]* | **__ __/__ __/__ __ __ __**  D D M M Y Y Y Y | | | **Final result:**  🞎 | |
| G2.7 | Time interview finished  *[Record hour 1-24 / minutes 1-60]* | **__ __/__ __**  H R M M | | |  | |
|  | **Interview result codes:**  1. Completed (Final result code)  2. Partially completed (Final result code)  3. Eligible respondent postponed interview  4. No eligible respondent at home at time of visit  5. Eligible respondent refused interview | | 6. No eligible respondent lives in household  7. No household member at home  8. Dwelling vacant / destroyed / not found  9. In progress (Interim result code)  10. Child reported dead in birth history is actually alive  11. Duplicate report of death – interview already conducted | | | |
| G2.8 | Date form checked by supervisor | **__ __/__ __/__ __ __ __**  D D M M Y Y Y Y | | |  | |
| G2.9 | Date entered in computer | **__ __/__ __/__ __ __ __**  D D M M Y Y Y Y | | |  | |
| **INTERVIEW BEGINS**  *Instructions to interviewer: Introduce yourself and explain the purpose of your visit. Ask to speak to the mother or to another adult who was the deceased’s main caregiver during the illness that led to death. If this is not possible, arrange a time to revisit the household when the caregiver will be home. (See example below.)*  “My name is [your name]. I am an interviewer with the ____________ project. I have been informed that a child death has occurred in your household. I am very sorry to hear this. Please accept my sympathies. For the purpose of improving health care, we are collecting information on recent child deaths in this area. I would like to talk to the mother or main caregiver of <NAME> and ask some questions about the events and any symptoms that <NAME> had during her/his illness before death.”  Suna na <YOUR NAME>.Na zo ne daga Hukumar Kidayar Jama’a ta Kasa. An shaida mana a zuwan mu na karshe cewa kun yi rashin yaro/yarinya awannan gidan. Muna yi maku ta’aziyya. Domin tabbatar da ingancin kiwon lafiya, muna tattara bayanai kan rasuwar yara a wannan gari.  Ina son in yi Magana da mahaifiyar ko ainihin mai kula da <SUNA> in yi wadansu yan tambayoyi kan wasu abubuwa da alamomin da <SUNA> ya/ ta ke dasu lokacin rashin lafiyar kafin rasuwar | | | | | | |
| **Section 3: Consent**  ***INTERVIEWER: Read the consent form to the respondent. Ask the respondent if he or she has any questions. Once any questions are answered, ask the respondent if he or she is willing to take part in the study.*** | | | | | | |
| G3.1 | *INTERVIEWER: Did respondent give consent?* | 1. Yes 2. No | | 🞎 ***2 → Thank respondent for their time and end the interview.*** | | |
| **Section 4: Information about the respondent**  *Read:* I would now like to ask you some general questions about yourself.  Ina son yanzu in tambaya ki wadansu yan tamboyoyi game da kan ki? | | | | | | |
| G4.1 | What is your (the respondent’s) name? Menene sunan ki? |  | | | | |
| G4.2 | *INTERVIEWER: What is the sex of the respondent?* | 1. Male 2. Female | | | 🞎 | |
| G4.3 | What is your relationship to the deceased child?  Shin menene dangantakar ki da mai rasuwar? | 1. Mother 2. Father 3. Grandmother 4. Grandfather 5. Aunt 6. Uncle 7. Brother 8. Sister 9. Birth attendant *(specify type)* 10. Other male *(specify)* 11. Other female *(specify)* | | 🞎🞎  ___________________________  ___________________________  ___________________________ | | |
| G4.4 | How old are you? Shin shakarun ki nawa? | | | __ __ Years  *(DK = 99)* | | |
| G4.5 | How many years of school did you complete? *[Do not include repeated years.]*  *Aji ko azuzuwa nawa ki ka kamala a mkakaranta?* | | | **__ __** Years  *(<1 = 00; DK = 99)* | | ***>6 years***  ***→ GQ4.6*** |
| G4.5.1 | Now I would like you to read this sentence to me. (*Show card to respondent)*  *Yanzu ina son ki karanta mun wanan shadaran?*  *If she cannot read the whole sentence, probe:* Can you read any part of the sentence to me? | 1. Cannot read at all 2. Able to read only part of sentence 3. Able to read whole sentence 4. No card available to show mother | | 🞎 | | |
| *Read:* I would now like to ask you some questions about (your / the family’s) household. Please remember that all information will be kept confidential.  Yanzu ina son in tambayaki wadansu yan tamboyoyi game da (naki / nasu ) gidan  *[Read "...the family's household.” if you are not conducting the interview at the household where the death was identified.]* | | | | | | |
| G4.6 | How many people live at (this / that) household?  Shin mutane nawa ne ke zama a (wannan/ wancan) gidan ?  *[Read “…at that household?” if you are speaking of “the family’s household.”]* | | | __ __ People  *(DK = 99)* | | |
| G4.7 | How many sleeping rooms are in the household? Shin dakunan kwana nawa ne a gidan? | | | __ __ Rooms  *(DK = 99)* | | |
| G4.8 | Does the household have a separate room for cooking? Shin a gidan nan Kuna da dakin girki daban? | 1. Yes 2. No   9. Don’t know | | 🞎 | | |
| G4.9 | Does the household have:  Gidan na da akwai  *[Ask about each possession, and mark each one “Yes,” “No” or “Don’t know.”]* | Does the household have:   1. electricity?Wutar Lantarki ? 2. a radio? Rediyo ? 3. a television? Telebijin? 4. a refrigerator? Friji? 5. a fixed line telephone? Wayar gida?   Does it have:   1. a mobile telephone? Wayar Hanu? 2. a computer? Naura mai kwakwalwa? 3. a bicycle? Keke ? 4. a car or truck?Mota ko tirela?   Does the household have:   1. piped water outside the residence?   Ruwan pompo a wajen gidan?   1. a well (protected or unprotected)?   Rijiya (rufeffe ko budedde)?   1. a water vendor, water supplied by truck or bottled water?   Mai moya, ruwa daga tankin mota, rowan kwalba?   1. surface water?   Ruwa a kwance? | | Yes No DK  1. □ 2. □ 9. □  1. □ 2. □ 9. □  1. □ 2. □ 9. □  1. □ 2. □ 9. □  1. □ 2. □ 9. □    1. □ 2. □ 9. □  1. □ 2. □ 9. □  1. □ 2. □ 9. □  1. □ 2. □ 9. □    1. □ 2. □ 9. □  1. □ 2. □ 9. □    1. □ 2. □ 9. □  1. □ 2. □ 9. □ | | |
| G4.10 | What type of toilet does the household have?  Wane irin wurin bahaya kuka fi amfani dashi a wannan gidan? | 1. Flush toilet 2. Improved pit toilet 3. Traditional pit toilet 4. Bush/field/beach 5. Other *(specify)*   9. Don’t know | | 🞎  ___________________________ | | |
| G4.11 | What is the main kind of energy the household uses for cooking?  Me kuka fi yin amfani da shi wajen dafa abinci a wannan gidan? | 1. Charcoal 2. Firewood 3. Kerosene 4. Electricity 5. Gas 6. Cow dung 7. Other *(specify)*   9. Don’t know | | 🞎  ___________________________ | | |
| G4.12 | What is the main material used for the floor of the house?  Menene ainihin abin da aka yi amfani das hi wajen gini kasan daki? | 1. Natural/mud 2. Cement 3. Wood 4. Tiles 5. Other *(specify)*   9. Don’t know | | 🞎  ___________________________ | | |

| **Section 5: Information about others at the interview** | | | | | | | | |
| --- | --- | --- | --- | --- | --- | --- | --- | --- |
| G5.7 | | *INTERVIEWER: Are there other people present during the interview?* | | 1. Yes 2. No | | 🞎 ***2 → GQ5.9*** | | |
| G5.8 | | *INTERVIEWER: In addition to the respondent, how many people are present during the interview?* | | | | __ __ Other people  *(DK = 99)* | | |
| G5. | 9 | *INTERVIEWER: Mark the respondent in the below table and whether s/he was present during the child’s illness and/or death. For each other person present at the interview, ask the respondent their relationship to the deceased and whether they were present during the child’s illness and/or at the death. For stillbirths and neonatal deaths, also ask if each person (other than the mother) was present during the mother’s pregnancy and delivery.* | | | | | | |
|  |  | Relationship of person to the deceased child | Mark (X)  if present at the interview | Stillbirths and neonatal deaths only | | | Neonatal & older child deaths only | |
|  |  |  |  | Present during  the pregnancy:  1. Yes / 2. No | Present at  the delivery:  1. Yes / 2. No | | Present during child’s illness:  1. Yes / 2. No | Present at the child’s death:  1. Yes / 2. No |
|  | .1 | Mother | 🞎 |  |  | | 🞎 | 🞎 |
|  | .2 | Father | 🞎 | 🞎 | 🞎 | | 🞎 | 🞎 |
|  | .3 | Grandmother | 🞎 | 🞎 | 🞎 | | 🞎 | 🞎 |
|  | .4 | Grandfather | 🞎 | 🞎 | 🞎 | | 🞎 | 🞎 |
|  | .5 | Aunt | 🞎 | 🞎 | 🞎 | | 🞎 | 🞎 |
|  | .6 | Uncle | 🞎 | 🞎 | 🞎 | | 🞎 | 🞎 |
|  | .7 | Brother | 🞎 | 🞎 | 🞎 | | 🞎 | 🞎 |
|  | .8 | Sister | 🞎 | 🞎 | 🞎 | | 🞎 | 🞎 |
|  | .9 | Traditional birth attendant | 🞎 | 🞎 | 🞎 | | 🞎 | 🞎 |
|  | .10 | Other male (specify:  __________________) | 🞎 | 🞎 | 🞎 | | 🞎 | 🞎 |
|  | .11 | Other female (specify  __________________) | 🞎 | 🞎 | 🞎 | | 🞎 | 🞎 |

| **VA Section 1: Background (FOR STILLBIRTHS, NEONATAL & CHILD DEATHS 0—59 MONTHS OLD)** | | | | |
| --- | --- | --- | --- | --- |
| V1.1 | Was the deceased a singleton or multiple birth?  Shin haihuwar marigayin/marigayiyar tagwaye ne ko daya ne ?  *[If two or more children are born at the same time, it is counted as a multiple birth, even if one or more of the babies are born dead.]* | | 1. Singleton 2. Multiple   9. Don’t know | 🞎 ***1 or 9 → VQ1.3*** |
| V1.2 | Was this the first, second, or later in the birth order?  Wannan Haihuwar ta farko ne, ta biyu ko daga bisani? | | 1. First 2. Second 3. Third or more   9. Don’t know | 🞎 |
| V1.3 | *If the mother is present, mark “Yes” and do not ask this question.*  Is the mother still alive?  Mahaifiyar tana da rai ? | | 1. Yes 2. No | 🞎 ***1 → VQ1.6*** |
| V1.4 | Did the mother die during or after the delivery?  Mahaifiyar ta rasu lokacin haihuwar ne ko bayan haihuwar? | | 1. During 2. After   9. Don’t know | 🞎 ***1 or 9 → VQ1.6*** |
| V1.5 | How long after the delivery did the mother die?  *Bayan haihuwar har tsawon wane lokaci ne kafin, mahaifiyar ta rasu ?*  *[Record days if less than 28 days—if less than 24 hours, record “00” days;*  *Record months if 28 days or more]* | | | **__ __** Days  *(DK = 99)* |
|  |  |  |  | **__ __** Months  *(DK = 99)* |
| V1.6 | Where was the deceased born?  A ina aka haifi marigayi /marigayiyar ? | | 1. Hospital 2. Other health provider or facility 3. On route to a health provider or facility 4. Home 5. Other *(specify)*   9. Don’t know | 🞎  ___________________________ |
| V1.7 | At the time of the delivery was the deceased:  *Lokacin haihuwar yaya misalin nauyin marigayi/marigayiyar ?*  *[Read the question and slowly read the first four choices. Respondent should hear all four choices & then respond.]*  *Show photos]* | | 1. Very small -Ta/ya yi kankanta kwarai 2. Smaller than usual- kankanuwa fiye da madaidaci 3. About average - madaidaci 4. Larger than usual-Girma fiiye da kima   9. Don’t know | 🞎 |
| V1.8 | What was the weight of the deceased at birth? *[Ask to see the child’s health card. If the card is available, record the birth weight from the card.]*  *Lokacin haihuwar menene nauyin marigayin/marigayar ?* | | | **__ __ __ __** Grams  *(DK = 9999)* |
| V1.9 | What was the sex of the deceased?  Marigayin namiji ne ko mace? | | 1. Male 2. Female   9. Don’t know | 🞎 |
| V1.10 | What was the delivery date?  Menene ranar haihuwar?  *Compare the delivery date just stated by the respondent to the birth date from the prior record (GQ1.4). Discuss any inconsistency with the respondent to confirm or correct the stated delivery date. You cannot change the prior record’s date.* | | | **__ __/__ __/__ __ __ __**  D D M M Y Y Y Y  *(DK = 99/99/9999)* |
| V1.11 | Was the child born alive or dead?  An haife ‘dan/ ‘yar da rai ko ba rai? | | 1. Alive 2. Dead   9. Don’t know | 🞎 |
| V1.12 | Did the baby ever cry?  ‘Dan/ ‘Yar ta/ya yi kuka bayan haihuwa? | | 1. Yes 2. No   9. Don’t know | 🞎 |
| V1.13 | Did the baby ever move?  Dan/ ‘Yar ta/ya yi motsi bayan haihuwa? | | 1. Yes 2. No   9. Don’t know | 🞎 |
| V1.14 | Did the baby ever breathe?  ‘Dan/ ‘Yar ta/ya yi nunfashi bayan haihuwa? | | 1. Yes 2. No   9. Don’t know | 🞎 |
| V1.15 | *Refer to VQ1.11–1.14. If “Dead” & no crying, movement or breathing, mark “Stillbirth.” If “Alive” & VQ1.12–1.14 = “No,” or if “Dead” and VQ1.12, 1.13 or 1.14 = “Yes,” then discuss & correct.* | | 1. Stillbirth 2. Live birth | 🞎 ***2 → VQ1.20*** |
| **Stillbirths** | | | | |
| V1.16 | Were there any bruises or signs of injury on the baby’s body at birth?  Akwai wasu alamomin jin ciwo ko rauni a jikin ‘dan/’yar lokacin haihuwa? | | 1. Yes 2. No   9. Don’t know | 🞎 |
| V1.17 | Was the baby’s body (skin and tissue) pulpy?  Jikin jariri /jaririyar (fatan jiki) da alamar kumburi | | 1. Yes 2. No   9. Don’t know | 🞎 |
| V1.18 | Was any part of the baby physically abnormal at the time of delivery? (for example: body part too large or too small, additional growth on body)  Akwai wani nakasu a jikin ‘dan/ ‘yar lokacin haihuwa? (Misali: wani sashe daga jikin yai girma ko kankanta, ko wani abu ya fito/tsira a jikin? | | 1. Yes 2. No   9. Don’t know | 🞎 ***2 or 9 → SQ3.1*** |
| V1.19 | What were the abnormalities?  *Wanne irin nakassa ne?*  *Ask for the following abnormalities*  *[Mark all that apply – Show photos]* | | 1. Was the head size very small at the time of birth-kan ta/shi yayi kankanta sosai a lokacin haihuwar 2. Was the head size very large at the time of birth – kan ta/shi yayi girma sosai a lokacin haihuwar 3. Was there a mass defect on the back of head or spine- shin akwai nakasa a keyan ko gadon bayan 4. Was there any other abnormality -shin akwai wata nakasan kuma. *(If “Yes,” then specify)* | Yes No  1. □ 2. □    1. □ 2. □  1. □ 2. □  1. □ 2. □ __________________ |
| ***Inst_1: STOP. After completing VQ1.19 → SQ3.1 (Maternal history)*** | | | | |
| **Live births** | | | | |
| V1.20 | | How old was the child when the illness started?  *Shekarun ‘dan/ ‘yar nawa lokacin da rashin lafiyar ya fara?*  *[Record days if less than 28 days—if less than 24 hours, record “00” days;*  *Record months if 28 days-11 months;*  *Record years if 1 year or older.]* | | **__ __** Days  *(DK = 99)* |
|  |  |  |  | **__ __** Months  *(DK = 99)* |
|  |  |  |  | **__ __** Years  *(DK = 99)* |
| V1.21 | | How long did the illness last?  *Rashin lafiyar ya kai har tsawon wanne lokaci ?*  *[Record days if less than 28 days—if less than 24 hours, record “00” days;*  *Record months if 28 days or more.]* | | **__ __** Days  *(DK = 99)* |
|  |  |  |  | **__ __** Months  *(DK = 99)* |
| V1.22 | | Where did the deceased die?  A ina yaron ko yarinyar ya/ta rasu? | 1. Hospital , Asibiti 2. Other health provider or facility wani jamiin kiwon lafiya ko cibian kiwon lafiya 3. On route to a health provider or facility, hanyar zuwa wajen jamiin kiwon lafiya ko cibian kiwon lafiya 4. Home , Gida 5. Other *(specify)*   9. Don’t know | 🞎  ___________________________ |
| V1.24 | | What was the date of death?  Menene ranar rasuwar?  *Compare the date of death just stated by the respondent to the date of death from the prior record (GQ1.5). Discuss any inconsistency with the respondent to confirm or correct the stated date. You cannot change the prior record’s date.* | | **__ __/__ __/__ __ __ __**  D D M M Y Y Y Y  *(DK = 99/99/9999)* |
| V1.25 | | ***AGE AT DEATH***  ***Record only the calculated age OR the stated age. First try to calculate the age. If this is not possible, then ask the respondent for the child’s age at death.*** | | |
|  |  | *CALCULATE THE AGE AT DEATH*  *Record the delivery date from VQ1.10:* **__ __/__ __/__ __ __ __**  D D M M Y Y Y Y  *(Don’t Know = 99/99/9999)*  *Record the date of death from VQ1.24:* **__ __/__ __/__ __ __ __**  D D M M Y Y Y Y  *(Don’t Know = 99/99/9999)*  *Now, if possible, calculate the age at death (VQ1.24 – VQ1.10). If only the month and year are known, you may still be able to calculate the approximate age in months or years. Discuss the calculated age with the respondent:* I have calculated that the child was (about) <CALCULATED AGE> at death. Is this correct?  *If the respondent does not agree with the calculated age, then again discuss the delivery date and date of death to make sure that these are correct. If the calculated age at death cannot be resolved, then go below to the “STATED AGE” box.*  *Once the age at death is calculated, check VQ1.20 and VQ1.21 to make sure that the age at illness onset and the illness duration are consistent with the age at death. For example, the age at onset + duration cannot be greater than the age at death.*  *[Record days if less than 28 days—if less than 24 hours, record “00” days; Record months if 28 days-11 months; Record years if 1 year or older.]*  *After recording the calculated age* ***→ VQ1.26*** | | **__ __** Days ***(if < 28 days)***  *(DK = 99)* |
|  |  |  |  | **__ __** Months ***(if 1-11 months)***  *(DK = 99)* |
|  |  |  |  | **__ __** Years ***(if 1 year or older)***  *(DK = 99)* |
|  |  | *STATED AGE AT DEATH (Ask only if the calculated age cannot be determined)*  How old was the deceased at the time of death?  Shekarun marigayin/marigayiyar nawa ne lokacin rasuwar ?  *Compare the age at death just stated by the respondent to the child’s last known age from the prior record (GQ1.6). Discuss any inconsistency with the respondent to confirm or correct the stated age. You cannot change the prior record’s age. Partly known delivery and death dates might help resolve the stated age. For example, if the child was born and died in the same month, then this is likely a neonatal death.*  *Once the age at death is determined, check VQ1.20 and VQ1.21 to make sure that the age at illness onset and the illness duration are consistent with the age at death. For example, the age at onset + duration cannot be greater than the age at death.*  *[Record days if less than 28 days—if less than 24 hours, record “00” days; Record months if 28 days-11 months; Record years if 1 year or older.]* | | **__ __** Days ***(if < 28 days)***  *(DK = 99)* |
|  |  |  |  | **__ __** Months ***(if 1-11 months)***  *(DK = 99)* |
|  |  |  |  | **__ __** Years ***(if 1 year or older)***  *(DK = 99)* |
| V1.26 | | *Mark the baby’s age at the time of death.*  *[Use the calculated age (VQ1.24 – VQ1.10) if known, or the stated age (VQ1.25). If both the calculated and stated ages are unknown, then use your best judgment to mark the child’s age at death.]* | 1. Less than 28 days old 2. 1-59 months old | 🞎 ***2 → SQ5b.1*** |

| **SA Module 3 and VA Section 2: Maternal history (FOR STILLBIRTHS AND NN DEATHS < 28 DAYS OLD)**  *Read:* Now, I would like to ask you some questions about (your / the mother’s) health and (your / her) pregnancy with <NAME>.  *Here and in the following questions, read “…the mother…,” “…her…” and “…she…” if the mother is not the respondent.*  *Yanzu, ina son in yi maki tambayoyi game da ( lafiyar ki/ mahaifiyar ) yayin cikin* <NAME>. | | | | |
| --- | --- | --- | --- | --- |
| S3.1 | Before the pregnancy with <NAME>, did (you / the mother) suffer from any of the following known conditions:  Kafin samun cikin <NAME>, Shin (kin /mahaifiyar ta) samu wasu daga cikin wadannan matsalolin?  *[Read out all options and check “Yes,” “No” or “Don’t know” for each.]*  *If “Yes,” then ask:* Did (you / she) undergo treatment for this condition during the pregnancy?  Shin a yayin cikin (Kin /Ta) nemi kiwon lafiya domin wadanan matsololin ? | 1. High blood pressure-Hawan Jini 2. Heart disease- Ciwon Zuciya 3. Diabetes- Ciwon Suga 4. Epilepsy/convulsion- Farfaduwa 5. Other –Wadansu kuma   *(specify other)* | Suffered from  Yes No DK  1. □ 2. □ 9. □  1. □ 2. □ 9. □  1. □ 2. □ 9. □  1. □ 2. □ 9. □  1. □ 2. □ 9. □ | Treatment  Yes No DK  1. □ 2. □ 9. □  1. □ 2. □ 9. □  1. □ 2. □ 9. □  1. □ 2. □ 9. □  1. □ 2. □ 9. □ |
|  |  |  | ___________________________ | |
| S3.2 | During the pregnancy, did (you / the mother) see anyone for antenatal care?  A yayin wannan cikin shin (kin /mahaifiyar ta) ga jami’an kiwon lafiya don awon ciki? | 1. Yes 2. No   9. Don’t know | 🞎 ***2 or 9 → SQ3.3*** | |
| S3.2.1 | Whom did (you / she) see? Anyone else?  *[Probe, and record all persons seen.]*  *Wanene (ki ka /mahaifiyar ta) gani?*  *Akwai wani kuma?* | 1. Health care provider-Jamian kiwon lafiya 2. TBA/Religious healer-Ungozoma/ mallaman addini 3. Relative/neighbor/friend –yan uwa/makwabta/saurayi 4. Other *(specify) –Wanene kuma*   *(_____________________________)*  9. Don’t know | 1. □  2. □  **3. □**  4. □  ***SQ3.3***  9. □ | |
| S3.2.2 | How many times did (you / the mother) receive antenatal care from a health care provider during this pregnancy?  A yayin wannan cikin sau nawa (ki ka/ mahaifiyar ta) ga jami’an kiwon lafiya domin awon ciki? | | __ __ Times  *(DK = 99)* | |
| S3.2.3 | During which month of the pregnancy did (you / the mother) last receive antenatal care from a health care provider?  Cikin na da wata nawa ne (ki ka/mahaifiyar ta) ga jami’an kiwon lafiya domin awon ciki na karshe? | | __ __ Month  *(DK = 99)* | |
| S3.2.4 | During this pregnancy, did the provider do any of the following for (you / the mother) at least once?  A yayin *wannan cikin, jami’in kiwon lafiya ya/ta yi (maki / ma mahaifiyar) daya daga cikin wadannan abubuwan a kalla sau daya?*  *[Read out all options and check “Yes,” “No” or “Don’t know” for each.]*  *[LOCAL ADAPTATION: Additional high energy and high protein foods to mention If the respondent asks]*  *[Read out all options and check “Yes,” “No” or “Don’t know” for each.]*  *[LOCAL ADAPTATION: Additional high energy and high protein foods to mention If the respondent asks]* | 1. Did the provider measure (your / her) blood pressure-Gwajin hawan jini 2. Did (you / she) give a urine sample? Gwajin fitsari 3. Did (you / she) give a blood sample? Gwajin jini 4. FOODS> and high protein Did the provider tell (you / her) to eat more high energy foods like <HIGH ENERGY foods like <HIGH PROTEIN FOODS> than when not pregnant? 5. Did the provider tell (you / her) about the danger signs during pregnancy? –Fada maki *hatsororin* goyon ciki 6. Did the provider tell (you / her) where to go if (you / she) had any danger signs?   Fada maki wurin da za aje idan an samu *hatsororin.* | Yes No DK    1. □ 2. □ 9. □  1. □ 2. □ 9. □  1. □ 2. □ 9. □  1. □ 2. □ 9. □  1. □ 2. □ 9. □  1. □ 2. □ 9. □ | |
| S3.3 | Please tell me the danger signs during pregnancy or labor and delivery that you should seek care for immediately.  *Ki fada mani wasu hatsarorin dake tare da samun ciki ko nakuda da haihuwa da ke bukatar kulawa da gaggawa.*  *Probe:* Tell me as many of the danger signs as you can.  *Fada mani hatsororin da ki ka sani?*  *Probe:* Can you tell me any others?  Zaki iya fada mani wadansu kuma?  *[Check each danger sign mentioned.]* | 1. Vaginal bleeding 2. Convulsions/fits 3. Severe headache with blurred vision 4. Fever and too weak to get out of bed 5. Severe abdominal pain 6. Fast or difficult breathing 7. Painful contractions every 20 minutes or less for 12 hours or more 8. Broken water for 12 hours or more 9. Bloody, sticky discharge 12 hrs or more 10. No immediate danger sign mentioned | 1. □  2. □  3. □  4. □  5. □ **___** Mentioned  6. □  7. □  8. □  9. □  10. □ | |
| S3.4 | During this pregnancy, (were you / was the mother) given an injection in the arm to prevent the baby from getting tetanus, that is, convulsions after birth?  A yayin wannan cikin, anyi ( maki /ma mahaifiyar) allura a gefen kafada domin kare jariri daga cutar sarke hakora (tetanus) ko tsinka-tsinka bayan haihuwa? | 1. Yes 2. No   9. Don’t know | 🞎 ***2 or 9 → SQ3.5*** | |
| S3.4.1 | During this pregnancy, how many times did (you / she) get this injection?  A yayin wannan cikin, sau nawa aka yi ( maki/ ma mahaifiyar) wannan allurar? | | __ Times  *(DK = 9)* | |
| S3.5 | At any time before this pregnancy, did (you / the mother) receive any tetanus injection, either to protect yourself or another baby?  Kafin ki samu wannan cikin an taba yi (maki/ ma mahaifiyar) allurar cutar sarke hakora domin kare kan ki/ta ko wani 'da/’yar? | 1. Yes 2. No   9. Don’t know | 🞎 ***2 or 9 → SQ3.6*** | |
| S3.5.1 | Before this pregnancy, how many other times did (you / she) receive a tetanus injection?  Kafin wannan cikin sau nawa aka yi (maki/ ma mahaifiyar) allurar sarke hakora?  *[If 7 or more time, record “7.”]* | | __ Times  *(DK = 9)* | |
| S3.6 | *Skip SQ3.6-3.7.1 in areas wo/malaria.*  During this pregnancy, did (you / the mother) sleep under an insecticide treated bednet?  A yayin wannan cikin shin (kin/maifiyar ta) kwana a karkasin gidan sauron da aka sa masa magani? | 1. Yes, usually or always 2. Yes, sometimes 3. Never   9. Don’t know | 🞎 | |
| S3.7 | During this pregnancy, did (you / the mother) take any drug to prevent (you / her) from getting malaria?  A yayin wannan cikin shin (kin/maifiyar ta)  sha kwayoyin maganin kariya daga zazzabin cizon sauro? | 1. Yes 2. No   9. Don’t know | 🞎 ***2 or 9 → VQ2.1*** | |
| S3.7.1 | During this pregnancy, how many times did (you / she) take this drug?  A yayin wannan cikin shin  sau nawa (ki ka/mahaifiyar ta) sha wadannan kwayoyin? | | __ __ Times  *(DK = 99)* | |
| V2.1 | Now I’d like to ask you about any problems (you / the mother) might have had during the pregnancy. Was the late part of the pregnancy (defined as the last 3 months), labor or delivery complicated by any of the following problems that started before the baby was delivered?  *Yanzu ina son in tambayeki game da matsalolin da (ki /mahaifiyar ta) ke da su* a yayin wannan cikin .  *Lokacin karshe na wannan cikin (ana iya cewa watanni ukkun karshe)Ko wadannan matsalolin na nakuda da haihuwa sun fara kafin haihuwar jariri?*  *[Read each complication and mark “Yes,” “No” or “Don’t know” for each.]*  *[Read “…the mother…” if the mother is not the respondent.]* | Did (you / the mother) have:   1. convulsions? 2. high blood pressure? 3. severe anemia or pallor and shortness of breath? 4. diabetes? 5. severe headache? 6. blurred vision?   (Were you / Was she):   1. too weak to get out of bed?   Did (you / the mother) have:   1. severe abdominal pain? 2. fast or difficult breathing? 3. puffy face? 4. any vaginal bleeding before labor? 5. excessive bleeding during labor or delivery? 6. fever? 7. smelly vaginal discharge?   Was the:   1. child delivered not head first? 2. cord delivered first? 3. cord around the child’s neck?   Did (you / the mother) have:   1. any other complication?   *(specify the other complication)* | Yes No DK  1. □ 2. □ 9. □  1. □ 2. □ 9. □  1. □ 2. □ 9. □  1. □ 2. □ 9. □  1. □ 2. □ 9. □  1. □ 2. □ 9. □    1. □ 2. □ 9. □  1. □ 2. □ 9. □  1. □ 2. □ 9. □  1. □ 2. □ 9. □  1. □ 2. □ 9. □    1. □ 2. □ 9. □  1. □ 2. □ 9. □  1. □ 2. □ 9. □  1. □ 2. □ 9. □    1. □ 2. □ 9. □  1. □ 2. □ 9. □  1. □ 2. □ 9. □  *____________________________* | |
| V2.2**^*^** | Did (you / the mother) have any of the following problems that started after the delivery?  Shin (kin/ *mahaifiyar ta ) samu wasu daga cikin wadannan matsalolin da suka fara bayan haihuwar?*  *[Read each complication and mark “Yes,” “No” or “Don’t know” for each.]*  *[Read “…the mother…” if the mother is not the respondent.]* | Did (you / the mother) have:   1. convulsions?- Farfadiya 2. heavy bleeding?Zuban Jini mai yawa 3. Fever with smelly vaginal discharge or abdominal pain? Zazzabi da jiwon mara ko ruwa mai warin gadke yana fitowa daga farji. | Yes No DK  1. □ 2. □ 9. □  1. □ 2. □ 9. □  1. □ 2. □ 9. □ | |
| V2.2 | How many months long was the pregnancy?  Shin cikin har tsawon *watanni nawa ne yakai?* | | **__ __** Months ***≠ 99 → VQ2.4***  *(DK = 99)* | |
| V2.3 | Did the pregnancy end early, on time, or late?  Shin cikin yazo karshe da sauri, kan lokaci ko daga baya (latti)? | 1. Early 2. On time 3. Late   9. Don’t know | 🞎 | |
| V2.4 | Was the baby moving in the last few days before the birth?  Ko cikin na motsi a kwanakin karshe kafin haihuwa? | 1. Yes 2. No   9. Don’t know | 🞎 | |
| V2.5 | When did (you / the mother) last feel the baby move?  *Yaushe ne ( ki ka /mahaifiyar ta) ji cikin yai motsi na karshe?*  *[Read “…the mother…” if the mother is not the respondent.]*  *[Record hours if less than 24 hours; Record days if 1 day or more.]* | | **__ __** Hours before delivery  *(DK = 99)* | |
|  |  |  | **__ __** Days before delivery  *(DK = 99)* | |
| V2.6 | Did the water break before labor or during labor?  *Ko faya ta fashe, kafin fara nakuda ko lokacin nakuda?*  *[Note: Labor begins when contractions are no more than 20 minutes apart.]* | 1. Before 2. During   9. Don’t know | 🞎 ***2 or 9 → VQ2.8*** | |
| V2.7 | How much time before labour did the water break?  *Har tsawon wanne lokacin ne faya ta fashe kafin nakuda ya fara?*  *[Record “24” if 1 day or more.]* | | **__ __** Hours  *(DK = 99)* | |
| V2.8 | What was the colour of the liquor when the water broke?  Menene kalar ruwan da faya ta fashe? | 1. Green or brown- kore ko rowan kasa 2. Clear (normal) daidai 3. Other *(specify)*   9. Don’t know | 🞎  ____________________________ | |
| V2.9 | Was the liquor foul smelling?  Ko ruwan fayar ya na wari? | 1. Yes 2. No   9. Don’t know | 🞎 | |
| V2.10 | How much time did the labor and delivery take?  *Har tsawon wanne lokaci ne nakudar da haihuwa yakai?*  *[Record “00” if less than 1 hour.]* | | **__ __** Hours  *(DK = 99)* | |
| S3.8 | Where did the delivery occur?  A ina aka yi haihuwar? | 1. Hospital 2. Other health provider or facility 3. On route to a health provider or facility 4. Home 5. Other *(specify___________________)*   9. Don’t know | 🞎 1-3 = Health provider  ***9 → SQ3.11*** | |
| S3.9 | Who decided that this was the right place to deliver the baby?  *Wanene ya yanke shawarar wurin da ya fi dacewa a haifi jaririn/jaririyar?*  *[Record the one main decision maker.]* | 1. The woman, herself 2. Her husband 3. Her mother 4. Her mother-in-law 5. Her father-in-law 6. Other *(specify)*   9. Don’t know | 🞎  ___________________________ | |
| S3.10 | *If she did not go to a health provider or facility (SQ3.8 = 4-5) for the delivery, ask:* Did (you / the mother) have any concerns or problems that kept (you / her) from going to a health provider or facility for the delivery?  *Shin (kin/mahaifiyar ta) samu uzurori ko matsaloli da ya sa ba (ki /ta) je wajen jami’in kiwon lafiya ba, ko cibiyar kiwon lafiya domin haihuwar?*  *If she went or was on route to a health provider or facility (SQ3.8 = 1-3) for the delivery, ask:* Did (you / the mother) have to overcome any concerns or problems to go to health provider or facility for the delivery?  *Shin (kin/mahaifiyar ta) ciwo kan wasu uzurori ko matsaloli kafin (ki ka /ta) je wajen jami’in kiwon lafiya ko cibiyar kiwon lafiya domin haihuwar?* | 1. Yes 2. No   9. Don’t know | 🞎 ***2 or 9 → SQ3.11*** | |
| S3.10.1 | What concerns or problems did (you / she) have?  *Wadanne uzurori ko matsaloli (ki ka/ mahaifiyar ta) samu?*  *Prompt:* Was there anything else?    Akwai wani abu kuma?  *[Multiple answers allowed.]* | 1. Did not think she was sick enough to need health care 2. No one available to go with her 3. Too much time from her regular duties 4. Someone else had to decide *(specify)* 5. Too far to travel 6. No transportation available 7. Cost (transport, health care, other) 8. Not satisfied with available health care 9. Symptom(s) required traditional care 10. Thought she was too sick to travel 11. Thought she/baby will die despite care 12. Was late at night (transportation or provider not available) 13. Fears exposure to male health provider 14. Other *(specify)*   99.Don’t know | 1. □  2. □  3. □  4. □ _______________________  5. □  6. □  7. □  8. □  9. □  10. □  11. □  12. □  13. □  14. □ ______________________  99. □ | |
| S3.11 | Who (at the facility) delivered the baby?  *Wanene (a’asibitin) ya amshi haihuwar*  *[Read “...at the facility...” if she delivered at a health facility.]* | 1. Doctor 2. Nurse/midwife 3. Relative/neighbor/friend 4. Self (the mother) 5. Traditional birth attendant 6. Other *(specify)*   9. Don’t know | 🞎  ___________________________ | |
| S3.12 | How soon after labor started did the <BIRTH ATTENDANT> first attend the mother?  *Menene tsawon lokacin da nakuda ta fara da* <BIRTH ATTENDANT> ta fara kulawa da mahaifiyar?  *[Discuss that labor starts with painful contractions every 20 minutes or less.]*  *[Mark days &/or hours as needed: e.g. 00 day, 06 hours]* | | __ __ Days  *(DK = 99)* | |
|  |  |  | __ __ Hours  *(DK = 99)* | |
| S3.13 | Did the birth attendant use a pictorial graph to follow the progress of (your / the mother’s) labour?  Shin mai kula da haihuwa tayi amfani da wasu hotuna domin bin diddikin nakudan (ki/mahaifiyar ? | 1. Yes 2. No   9. Don’t know | 🞎 | |
| S3.14 | Did the birth attendant wash her hands with soap and water or wear surgical gloves before assisting with the birth?  Shin mai kula da haihuwar ta wanke hannunta da ruwa da sabulu ko sanya safar hannu kafin taimakawa wajen haihuwar? | 1. Yes, washed with soap and water 2. Yes, wore surgical gloves 3. No   9. Don’t know | 🞎 | |
| S3.15 | On what surface did (you / the mother) deliver?  A wane irin wuri (ki ka /mahaifiyar ta) haihu? | 1. Labor bed 2. Solid floor with mackintosh/cover 3. Solid washed floor 4. Solid unwashed floor 5. Dirt/soil/mud/straw floor 6. Other *(specify)*   9. Don’t know | 🞎  ___________________________ | |
| V2.17 | Was the delivery...?  *Haihuwar ta kasance da wani taimako---?*  *[Read the choices and mark ONE.]* | 1. Vaginal with forceps -farji da taimako 2. Vaginal without forceps –farji ba taimako 3. Vaginal (don’t know) 4. C-section- aikin tiata   9. Don’t know | 🞎 | |
| V2.18 | During labour but before delivery, did (you / the mother) receive any kind of injection?  *Lokacin nakuda kafin haihuwa, ko anyi (maki/ ma mahaifiyar) wata allura?*  *[Read “…the mother…” if the mother is not the respondent.]* | 1. Yes 2. No   9. Don’t know | 🞎 | |

| **SA Module 4: Careseeking for maternal complications (FOR STILLBIRTHS AND NN DEATHS < 28 DAYS OLD)**  *Read:* Now, I would like to ask you some questions about (your / the mother’s) careseeking during the pregnancy with <NAME>.  Yanzu, Ina son in yi maki wadansu tambayyoyi kan lura da kiwon lafiyar (ki/mahaifiyar) yayin cikin<NAME>. | | | | | | | |
| --- | --- | --- | --- | --- | --- | --- | --- |
| S4.1 | **Maternal symptoms:**  *First look back at the maternal VA symptoms in GQ1.9. Mark (“X”) these in the “Symptoms in the last 3 months” column.*  *If she had any symptom(s), then read:* Earlier, you mentioned that (you / the mother) had <SYMPTOM(S)> during the last 3 months of the pregnancy or during labor or delivery. Which of the symptoms started before labor? And which started with or during labor or delivery, including any that may have brought on the labor?    Dazu kin ce ( kin/mahaifiyar ta) samu  <SYMPTOM(S)> cikin wattani 3 na karshe yayin wannan cikin ko lokacin nakuda ko lokacin haihuwa. Wadan ne matsololin ne ya fara kafin nakuda? Kuma wadan ne matsololin ne ya fara tare ko lokacin nakuda ko haihuwa, harda wanda ya janyo nakudan?  *[Remind the respondent that labor starts with painful contractions every 20 minutes or less. Then review each reported symptom with her to determine which started before labor and which started* with or during labor *or delivery. Do not include any symptoms here that started after the baby was delivered.]* | | 1. Convulsions 2. High blood pressure 3. Severe anemia or (pallor and SOB) 4. Diabetes 5. Severe headache 6. Blurred vision 7. Too weak to get out of bed 8. Severe abdominal pain (not labor pain) 9. Fast or difficult breathing 10. Puffy face 11. Any vaginal bleeding before labor 12. Excessive bleeding during labor or dlvr. 13. Fever 14. Smelly vaginal discharge 15. Early/preterm labor (less than 9 mnths) 16. Water broke 6 hrs or more before labor 17. Labor for 12 hours or more 18. Other *(specify)*   *(____________________________)*   1. No symptoms during last 3 months 2. No symptoms before labor | | Symptoms during last 3 months  Yes  □  □  □  □  □  □  □  □  □  □  □  □  □  □  □  □  □  □  □ ***→ Inst_8*** | | Started (related to labor/delivery)  Before W/D DK  1. □ 2. □ 9. □  1. □ 2. □ 9. □  1. □ 2. □ 9. □  1. □ 2. □ 9. □  1. □ 2. □ 9. □  1. □ 2. □ 9. □  1. □ 2. □ 9. □  1. □ 2. □ 9. □  1. □ 2. □ 9. □  1. □ 2. □ 9. □  1. □ 2. □ 9. □  1. □ 2. □ 9. □  1. □ 2. □ 9. □  1. □ 2. □ 9. □  1. □ 2. □ 9. □  1. □ 2. □ 9. □  1. □ 2. □ 9. □  1. □ 2. □ 9. □  □ ***→ SQ4.11*** |
| S4.2 | Did (you / the mother) seek care from any person or health facility for (any of) the pregnancy symptom(s) that started before labor?  *Shin (kin/mahaifiyar ta) nemi kulawa daga wani ko cibiyar kiwon lafiya akan matsala/ matsololin ciki dake faruwa kafin nakuda?*  *[Read “…for any of…” if she had more than one pregnancy symptom.]* | | 1. Yes 2. No   9. Don’t know | | 🞎 ***2 → SQ4.4***  ***9 → Inst_2*** | | |
| S4.2.1 | Where did (you / she) seek this care?  *A wane wurin (ki ka /mahaifiyar ta) nemi kulawar?*  *Prompt:* Was there anywhere else?  Da akwai wani wurin kuma?  *[Multiple answers allowed.]* | | 1. Hospital 2. NGO or government clinic 3. Private doctor/clinic 4. Community nurse or midwife 5. TBA/village doctor/quack/other non-formal or traditional provider 6. Relative, neighbor or friend 7. Other *(specify)*   *(*______________________________*)*  99. Don’t know | | 1. □  2. □  3. □ = Health provider  4. □  5. □  ***SQ4.4***  6. □  7. □  9. □ ***→ Inst_2*** | | |
| S4.3 | *If more than one symptom started before labor and she sought care from a health provider (SQ4.2.1 = 1-4), ask:*  For which symptom or symptoms that started before labor did (you / she) seek care from a health provider or facility?  Shin wane matsala/matsololin ne (ki ka/mahaifiyar ta) nemi kula daga jami’in kiwon lafiya ko cibiyar kiwon lafiya? | | 1. Convulsions □ 2. High blood pressure □ 3. Severe anemia or (pallor and SOB) □ 4. Diabetes □ 5. Severe headache □ 6. Blurred vision □ 7. Too weak to get out of bed □ 8. Severe abdominal (not labor) pain □ 9. Fast or difficult breathing □ | | 1. Puffy face □ 2. Any bleeding before labor □ 3. – blank – 4. Fever □ 5. Smelly vaginal discharge □ 6. – blank – 7. Water broke >6 hrs bfr. labor □ 8. – blank – 9. Other *(specified in SQ4.1)* □ | | |
| S4.4 | *If she never went to a health provider (SQ4.2 = 2 or SQ4.2.1 ≠ 1-4) for any of the pregnancy symptoms, ask:* Did (you / the mother) have any concerns or problems that kept (you / her) from going to a health provider or facility for the symptom(s) that started before labor?  Shin ( kin/ mahaifiyar ta) samu wani uzurori ko lallurai da ya hana (ki/ta) zuwa wajen jami’in kiwon lafiya ko cibiyar kiwon lafiya don da ya fara/faru kafin nakuda?  *If she went to health provider (SQ4.2.1 = 1-4) for any pregnancy symptom(s), ask:* Did (you / the mother) have to overcome any concerns or problems to go to a health provider or facility for the symptom(s) that started before labor?  Shin (kin/mahaifiyat ta) samu kin /ta ciwo kan uzurorin ko lalluran da (ki ka/ mahaifiyar ta) samu na zuwa wajen jami’in kiwon lafiya ko cibiyar kiwon lafiya don matsala/matsololin da ya fara/faru kafin nakuda? | | 1. Yes 2. No   9. Don’t know | | 🞎 ***2 or 9 → Inst_1*** | | |
| S4.4.1 | What concerns or problems did (you / she) have?  *Wadanne uzurori ko lallurai (ki ka/mahaifiyar ta)samu?*  *Prompt:* Was there anything else?  Da akwai wani abu kuma?  *[Multiple answers allowed.]* | | 1. Did not think was sick enough to need health care 2. No one available to go with her 3. Too much time from her regular duties 4. Someone else *(specify)* had to decide 5. Too far to travel 6. No transportation available 7. Cost (transport, health care, other) 8. Not satisfied with available health care 9. Symptom(s) required traditional care 10. Thought she was too sick to travel 11. Thought she/baby will die despite care 12. Fears exposure to male health provider 13. Other *(specify)*   99.Don’t know | | 1. □  2. □  3. □  4. □ _______________________  5. □  6. □  7. □  8. □  9. □  10. □  11. □  12. □  13. □ ______________________  99. □ | | |
| ***Inst_1: If SQ4.2 = 2 or SQ4.2.1 ≠ 1-4 (Never went to a health provider for any pregnancy symptoms) →*** ***Inst_2*** | | | | | | | |
| S4.5 | Did any health provider or facility refer (you / her) to another health provider or facility for (any of) the symptom(s) that started before labor?  Shin wani jami’in kiwon lafiya ko cibiyar kiwon lafiya sun tura (ki/ta) wajen wani jami’in kiwon lafiya ko cibiyar kiwon lafiya domin wata matsala/ matsalolin da suka faru kafin farawar nakuda? | | 1. Yes 2. No   9. Don’t know | | 🞎 ***2 or 9 → SQ4.6*** | | |
| S4.5.1 | Did (you / she) go to the provider or facility to which (you were / she was) referred?  Shin (kin/mahaifiyar ta) je wajen jami’in kiwon lafiyar ko cibiyar kiwon lafiyar da aka tura (ki/ ta)? | | 1. Yes 2. No   9. Don’t know | | 🞎 | | |
| S4.6 | How many different health providers or facilities did (you / the mother) see for the pregnancy symptom(s) that started before labor?  Shin jami’an kiwon lafiya ko cibiyoyin kiwon lafiya nawa (ki ka/ mahaifiyar ta) gani/je domin matsala/ matsololin ciki da ya faru kafin nakuda? | | | | **__ __** Health providers/facilities  *(DK = 99)* | | |
| S4.7 | (Were you / Was the mother) admitted to hospital for (any of) the symptom(s) that started before labor?  Shin an kwantar da (ke/mahaifiyar) a asibiti domin wannan matsalar/ matsololin da suka fara kafin nakuda? | | 1. Yes 2. No   9. Don’t know | | 🞎 | | |
| S4.8 | Please tell me everything that the provider(s) suggested that (you / the mother) do for the pregnancy symptom(s) at home?  Ki gaya mani dukkan shawarwarin da jami’in/ jami’an kiwon lafiya ya/ta ba (ki/mahaifiyar) da za ki/ta yi a gida, domin matsalar/ matsololin ciki  *Prompt:* Was there anything else?  Da akwai wani abun kuma ?  *[Multiple answers allowed.]* | | 1. Take antibiotic by mouth 2. Take antimalarial by mouth 3. Take BP medicine by mouth 4. Take other medicine by mouth 5. Rest / bed rest / decrease work 6. Return for follow-up visit(s) 7. Return or referred if worse 8. Other *(specify)* 9. Nothing   99. Don’t know | | 1. □  2. □  3. □  4. □  5. □  6. □  7. □  8. □ _______________________  9. □ ***→ Inst_2***  99. □ ***→ Inst_2*** | | |
| S4.9 | (Were you / Was the mother) able to follow all this advice?  Shin (kin/mahaifiyar ta) bi wadannan shawarwarin? | | 1. Yes 2. No   9. Don’t know | | 🞎 ***9 → Inst_2*** | | |
| S4.10 | *If not able to follow all the advice, ask:*  Did (you / she) have any concerns or problems that kept (you / her) from following the advice?  Shin (kin/ta) samu wadansu uzurori ko lallurai da ya sa ba (ki/ta) bi wadannan shawarwarin ba?  *If able to follow all the advice, ask:*  Did (you / she) have to overcome any concerns or problems to follow the advice?  Shin sai da (ki ka/mahaifiyar ta) ciwo kan wasu uzurorin ko lallurai kafin ki ka/ ta iya bin shawarwarin? | | 1. Yes 2. No   9. Don’t know | | 🞎 ***2 or 9 → Inst_2*** | | |
| S4.10.1 | What concerns or problems did (you / she) have?  Wadanne uzurori ko lallurai ne (ki ka /mahaifiyar ta) samu?  *Prompt:* Was there anything else?  Da akwai wani abu kuma?  *[Multiple answers allowed.]* | | 1. Did not understand instructions 2. Too much time from her regular duties 3. Someone else *(specify)* decided 4. Cost too much 5. Problem required traditional care 6. Advised care not needed or helpful 7. Advised care might harm unborn child 8. Thought she/baby will die despite care 9. Other *(specify)*   99.Don’t know | | 1. □  2. □  3. □ _______________________  4. □  5. □  6. □  7. □  8. □  9. □ _______________________  99. □ | | |
| ***Inst_2: Refer to SQ4.1: If no labor or delivery symptoms*** ***→******Inst_8*** | | | | | | | |
| S4.11 | Now let’s talk about the labor and delivery symptom(s). You said earlier that the symptom(s) that started with or during labor or delivery (was / were) <SYMPTOM(S)>.  Yanzu bari muyi magana kan nakuda da matsololin haihuwa. Kin shaida mani da farko cewa matsololin da suka fara tare ko kafin nakuda ko haihuwa sune <SYMPTOM(S)>.  *[Read and mark the SQ4.1 symptom(s) confirmed by the respondent. Correct the SQ4.1 responses if necessary.]* | | 1. Convulsions □ 2. High blood pressure □ 3. Severe anemia or (pallor and SOB) □ 4. – blank – 5. Severe headache □ 6. Blurred vision □ 7. Too weak to get out of bed □ 8. Severe abdominal (not labor) pain □ 9. Fast or difficult breathing □ | | 1. Puffy face □ 2. Any bleeding before labor □ 3. Excess bleed during L or D □ 4. Fever □ 5. Smelly vaginal discharge □ 6. Early/preterm labor (<9 mnth) □ 7. Water broke >6 hrs bfr. labor □ 8. Labor for 12 hours or more □ 9. Other *(specified in SQ4.1)* □ | | |
| S4.12 | Where (were you / was the mother) when (this / the first) symptom began?  *A ina (ki /mahaifiyar ta ) ke lokacin da (wannan/wadannan matsalan/matsololin) ya fara na farko?*  *[Read “…the first…” if she had more than one labor or delivery symptom.]* | | 1. Home 2. On route to a health provider or facility 3. At the health provider or facility where she went for normal labor 4. Other *(specify)*   9. Don’t know | | 🞎 ***3 → SQ4.17***  ___________________________ | | |
| S4.13 | Did (you / she) receive, seek or try to seek any care or treatment for (any of) the labor or delivery symptom(s)?  *[Read “…any of the symptoms” if she had more than one symptom.]*  *Shin (kin/mahaifiyar ta )samu ,nemi,ko*  *an kula da ke/ita ko anyi wani kokarin kulawa da ke/ita kan wani matsalar/ matsololin nakuda ko alamomin haihuwa?* | | 1. Yes 2. No   9. Don’t know | | 🞎***2 or 9 → SQ4.17*** | | |
| S4.13.1 | What was the first thing (you / she) did for the symptom(s)?  Menene abu na farko da (ki ka /ta) yi domin wannan matsalar/matsololin *?*  *[Mark only the first action taken.]* | | 1. Home treatment (at her own home, or by a relative, neighbor, or friend)   Sought or tried to seek care from a:   1. Hospital 2. NGO or government clinic 3. Private doctor/clinic 4. Community nurse or midwife 5. Pharmacist or drug seller 6. TBA/village doctor/quack/other non-formal or traditional provider 7. Other *(specify)*   99. Don’t know | | 🞎🞎***99 → SQ4.16***  __________________________ | | |
| S4.14 | Who decided that this was the right thing to do at that time?  *Wanene ya yanke shawarar cewa abunda ya kamata ayi kenan a wannan lokacin?*  *[Only one response allowed. Record the main decision maker.]* | | 1. The woman, herself 2. Her husband 3. Her mother 4. Her mother-in-law 5. Her father-in-law 6. Other *(specify)*   9. Don’t know | | 🞎  ___________________________ | | |
| S4.15 | *If she did not go to a health provider (SQ4.13.1 = 1 or 6-8), ask:* Did (you / the mother) have any concerns or problems that kept (you / her) from going to a health provider at that time?  Shin (kin/mahaifiyar ta) samu uzurorin ko lallurai da ya sa ba (ki/ta) je wajen jami’in kiwon lafiya ba a wannan lokacin?  *If she went to a health provider (SQ4.13.1 = 2-5), ask:* Did (you / the mother) have to overcome any concerns or problems to go to the <HEALTH PROVIDER> at that time?  Shin sai da (ki ka/mahaifyar ta) ciwo kan wasu uzurorin ko lallurai kafin ki ka/ta je <HEALTH PROVIDER> a wannan lokacin? | | 1. Yes 2. No   9. Don’t know | | 🞎 ***2 or 9 → Inst_3*** | | |
| S4.15.1 | What concerns or problems did (you / she) have?    Shin menene uzurorin ko lalluran da (ki ka /ta) samu ?  *Prompt:* Was there anything else?  Da akwai wani abun kuma?  *[Multiple answers allowed.]* | | 1. Did not think she was sick enough to need health care 2. No one available to go with her 3. Too much time from her regular duties 4. Someone else had to decide *(specify)* 5. Too far to travel 6. No transportation available 7. Cost (transport, health care, other) 8. Not satisfied with available health care 9. Symptom(s) required traditional care 10. Thought she was too sick to travel 11. Thought she/baby will die despite care 12. Was late at night (transportation or provider not available) 13. Fears exposure to male health provider 14. Other *(specify)*   99.Don’t know | | 1. □  2. □  3. □  4. □ _______________________  5. □  6. □  7. □  8. □  9. □  10. □  11. □  12. □  13. □  14. □ ______________________  99. □ | | |
| ***Inst_3: If SQ4.13.1 = 2-5 (First went to a health provider or facility)*** ***→******SQ4.16.1*** | | | | | | | |
| S4.16 | Did (you / she) ever seek or try to seek care from a health provider or facility for (any of) the labor or delivery symptom(s)?  Shin (kin/mahaifiyar ta) taba neman kulawar ko kokarin neman kulawar lafiya daga jami’in kiwon lafiya ko cibiyar kiwon lafiya domin matsalar ko (daya daga cikin) matsololin nakuda ko haihuwa? | | 1. Yes 2. No   9. Don’t know | | 🞎***2 or 9 → SQ4.17*** | | |
| S4.16.1 | Please tell me all the types of health providers and facilities where (you / she) sought or tried to seek care for (any of) the labour or delivery symptom(s).  *Ki gaya mani dukkan jami’an kiwon lafiya ko cibiyoyin kiwon lafiya da (ki ka/ta )samu kulawar su ko kokarin samun kulawarsu domin nakuda ko (daya daga cikin) alamomin haihuwa?*  *Prompt:* Anywhere else?  Da akwai wan wuri kuma?  *[Multiple answers allowed.]* | | 1. Hospital 2. NGO or government clinic 3. Private doctor/clinic 4. Community nurse or midwife   9. Don’t know | | 1. □  2. □  3. □  4. □  9. □ | | |
| S4.17 | *Refer to SQ3.8 to determine the delivery place. Discuss with respondent to confirm or correct the delivery place.*  *Discuss & resolve inconsistencies, for example, if SQ4.13 or 4.16 = “No,” but the mother delivered in a health facility.* | | 1. Hospital 2. Other health provider or facility 3. On route to a health provider or facility 4. Home 5. Other *(specify)*   9. Don’t know | | 🞎 1-3 = Health provider  ___________________________ | | |
| S4.18 | So, including where (you / the mother) went or tried to go for the labor or delivery symptom(s) and for the delivery, how many health providers or facilities did (you / she) go to?  Shin, harda inda (ki ka/ mahaifiyar ta) je ko neman zuwa domin nakuda ko alamomin haihuwa da kuma haihuwar, jami’an kula da lafiya ko cibiyoyin kiwon lafiya nawa (ki ka/mahaifiyar ta) je ?    *[If SQ4.16 = 2 and SQ4.17 = 4 or 5 → record ‘00’ health providers/facilities]*  *[If SQ4.16 = 2 and SQ4.17 = 1-3 → record ‘01’ health provider/facility]*  *[If SQ4.16 = 2 and SQ4.17 = 9 → record ‘99’ health providers/facilities]*  *[If SQ4.16 = 9 → record ‘99’ health providers/facilities]* | | | | **__ __** Health providers/facilities | | |
| ***Inst_4: If SQ4.12 = 3 (Symptoms began at the health provider where she went for normal labor) → SQ4.22*** | | | | | | | |
| ***Inst_5: If SQ4.16 = 2 or 9 & SQ4.17 = 4-9 (No health provider seen/sought for the symptoms/delivery) → Inst_8*** | | | | | | | |
| ***Inst_5.5: If SQ4.1 = only 1 labor or delivery symptom OR If SQ4.16 = 2 or 9*** ***→******SQ4.21*** | | | | | | | |
| S4.19 | Was there any particular symptom or symptoms for which (you / the mother) went to the (first) health provider?  Shin da akwai wata matsala ko matsololin da suka sa (ki ka/mahaifiyar ta) je wajen jami’in kiwon lafiya na (farkon)?  *[Read “…the first health provider?” if she went to more than one provider.]* | | 1. Yes 2. No   9. Don’t know | | 🞎***2 or 9 → SQ4.21*** | | |
| S4.20 | For which symptom(s) did (you / she) go?  Domin wacce matsalar/matsololin (ki ka /ta) je? | | 1. Convulsions □ 2. High blood pressure □ 3. Severe anemia or (pallor and SOB) □ 4. – blank – 5. Severe headache □ 6. Blurred vision □ 7. Too weak to get out of bed □ 8. Severe abdominal (not labor) pain □ 9. Fast or difficult breathing □ | | 1. Puffy face □ 2. Any bleeding before labor □ 3. Excess bleed during L or D □ 4. Fever □ 5. Smelly vaginal discharge □ 6. Early/preterm labor (<9 mnth) □ 7. Water broke >6 hrs bfr. labor □ 8. Labor for 12 hours or more □ 9. Other *(specified in SQ4.1)* □ | | |
| S4.21 | How long after the labour or delivery symptom(s) began was it decided to go to the (first) health provider?  *Menene tsawon lokacin da aka dauka bayan nakudan ko (alamomin) haihuwa ya fara kafin aka yanke shawarar zuwa wajen jami’in kiwon lafiya na (farkon)?*  *[Read “…to the first…” if she went or tried to go to more than one health provider.*  *[Mark days, hours &/or minutes as needed: e.g. 00 day, 02 hours, 10 minutes]* | | | | **__ __** Days  *(DK = 99)* | | |
|  |  |  |  |  | **__ __** Hours  *(DK = 99)* | | |
|  |  |  |  |  | **__ __** Minutes  *(DK = 99)* | | |
| ***Labor and delivery matrix instructions:*** *Ask the following questions for the first and last health providers where she sought/tried to seek care for the labor and delivery symptoms. If she delivered at a health provider/facility or at home or on route while trying to go to a health provider/facility, then that should be the first health provider (if she went to only one) or the last health provider. Ask all the questions for the first provider before going on to the last.*  *Before asking about the first health provider, read:*  Now I would like to ask about (your / the mother’s) visit to the (first) health provider. *[Read “first” if she went or tried to go to more than one provider.]*  Yanzu ina son na tambaya batun zuwan (ki/mahaifiyar) na (farko) wajen jami’in kiwon lafiya.  *Before asking about the last health provider, read:*  Now I would like to ask about (your / the mother’s) visit to the last health provider.  Yanzu ina son na tambaya batun zuwan (ki/mahaifiyar) na karshe wajen jami’in kiwon lafiya. | | | | | | | |
| **– LABOR AND DELIVERY MATRIX QUESTIONS –** | | | | **FIRST HEALTH PROVIDER** | | **LAST HEALTH PROVIDER** | |
| What was the name of the (first / last) health provider or facility where (you / the mother) (sought care for the labor or delivery symptom(s) / delivered the baby / tried to deliver the baby)?  *Probe to identify the type of provider.*  *Menene sunan jami’in kiwon lafiya/ cibiyar kiwon lafiya ( na farko ko na karshe) da (ki ka/ mahaifiyar ta) nemi kulawar kiwon lafiya domin nakuda ko alamar/ (alamomin) haihuwa da aka haifi jaririn?*  *Probe to identify the type of provider.* | | 1. Hospital (Government) 2. Hospital (NGO) 3. Hospital (Private) 4. Health center (Government) 5. Health center (NGO) 6. Health post (Government) 7. Health post (NGO) 8. Private doctor/clinic (Formal) 9. Private doctor/clinic (?Formal?) 10. Trained community nurse/midwife   99. Don’t know | | S4.22  🞎🞎  ___________________  (Name of Provider/Facility) | | S4.32  🞎🞎  ___________________  (Name of Provider/Facility) | |
| After (deciding to seek care / being referred), how much time passed before going to the <FIRST/LAST HEALTH PROVIDER>?  *Bayan (yanke shawarar neman kulawa kan lafiya ko turawa zuwa wata cibiyar kiwon lafiya), menene tsawon lokacin da aka dauka kafin aje wajen* <FIRST/LAST HEALTH PROVIDER>?  *[Discuss that this might include the time needed to arrange for transportation and money to go to the provider/facility, or to provide home care or go to a traditional provider before going to the health provider.]*  *[If she delivered at home, record the time from decision/referral to delivery.]*  *[Mark days, hours &/or minutes as needed: e.g. 00 days, 02 hours, 10 minutes]* | | | | S4.23  **__ __** Days  *(DK = 99)* | | S4.33  **__ __** Days  *(DK = 99)* | |
|  |  |  |  | **__ __** Hours  *(DK = 99)* | | **__ __** Hours  *(DK = 99)* | |
|  |  |  |  | **__ __** Minutes  *(DK = 99)* | | **__ __** Minutes  *(DK = 99)* | |
| Was there any cost to travel to the <FIRST/LAST HEALTH PROVIDER> or pay for (your / the mother’s) care there?  Akwai kudin sufuri/abun hawa da aka biya zuwa wajen <FIRST/LAST HEALTH PROVIDER> ko da aka biya (ma ki/ ma mahaifiyar) a wurin? | | 1. Yes 2. No   9. Don’t know | | S4.24  🞎 ***2 or 9 → SQ4.25*** | | S4.34  🞎 ***2 or 9 → SQ4.35*** | |
| How did (you / the mother) arrange for the money for these expenses?  *[Multiple answers allowed.]*  *Ta yaya (ki ka /mahaifiyar ta) yi dabarar biyan wadannan kudin aikin?* | | 1. Had available 2. Borrowed 3. Sold assets 4. Help from kin/relatives 5. Community fund 6. Govt. scheme 7. Other   9. Don’t know | | S4.24.1  1. □  2. □  3. □  4. □  5. □  6. □  7. □  9. □ | | S4.34.1  1. □  2. □  3. □  4. □  5. □  6. □  7. □  9. □ | |
| What transportation method was used to go there?  *[Multiple answers allowed.]*  *Wacce hanyar sufuri/abun hawa ku kayi amfani da ita wajen zuwa?*  *Multiple answers allowed.]* | | 1. Walk 2. Bicycle/rickshaw/cart boat 3. Bus 4. Taxi/auto/trecker 5. Ambulance (auto or motorcycle) 6. Other 7. Could not arrange transport   9. Don’t know | | S4.25  1. □ ***If only walk***  2. □ ***→ SQ4.26.1***  3. □  4. □  5. □  6. □  7. □ ***→ SQ4.26.1***  9. □ | | S4.35  1. □ ***If only walk***  2. □ ***→ SQ4.36.1***  3. □  4. □  5. □  6. □  7. □ ***→ SQ4.36.1***  9. □ | |
| How much did the transportation cost?  Nawa ne kudin sufurin/ abun hawa da ku ka kashe? | | | | S4.26  **__ __ __ __** unit  *(DK = 9999)* | | S4.36  **__ __ __ __** unit  *(DK = 9999)* | |
| Did (you / the mother) reach the <FIRST/LAST HEALTH PROVIDER> before delivering the baby?  *If “No,” discuss with respondent to reach correct response: 2, 3 or 4.]*  *Shin (kin /mahaifiyar ta) samu isa wajen* <FIRST/LAST HEALTH PROVIDER> kafin haihuwar jaririn?  *If “No,” discuss with respondent to reach correct response: 2, 3 or 4.]* | | 1. Yes, reached before delivering 2. No, delivered before setting out 3. No, delivered on route to provider 4. No, could not reach this provider – did not set out/returned home/took other action   9. Don’t know | | S4.26.1  🞎 ***2, 3 → Inst_8***  ***4, 9 → Inst_7*** | | S4.36.1  🞎 ***2-9 → Inst_8*** | |
| How long did it take to travel to the <FIRST/LAST HEALTH PROVIDER>?  *Har tsawon wane lokaci aka dauka wajen tafiya wurin* <FIRST/LAST HEALTH PROVIDER>?  *[Mark hours &/or minutes as needed: e.g. 05 hours, 30 minutes]* | | | | S4.27  **__ __** Hours  *(DK = 99)* | | S4.37  **__ __** Hours  *(DK = 99)* | |
|  |  |  |  | **__ __** Minutes  *(DK = 99)* | | **__ __** Minutes  *(DK = 99)* | |
| What did the <FIRST/LAST HEALTH PROVIDER> do for (your / the mother’s) (labor or delivery symptom(s) / delivery)?  *Wanne irin abu* ne aka yi ma (ki/ mahaifiyar) a wajen FIRST/LAST HEALTH PROVIDER> domin nakuda ko alamar/ (alamomin) haihuwa ko domin haihuwar?  *Prompt:* Was there anything else?  Da akwai wani abu kuma?  *[Multiple answers allowed.]* | | 1. Gave oxygen for the baby 2. Gave antibiotics by mouth 3. Gave antimalarial by mouth 4. Gave BP medicine by mouth 5. Other medicine by mouth *(specify)* 6. Gave medicine to stop bleeding 7. Gave medicine to stop convulsions 8. Gave medicine to strengthen labor 9. Gave medicine to stop labor 10. Gave medicine for baby’s lungs 11. Gave IM medicine 12. Gave IV fluids or medicine 13. Blood transfusion 14. Advised to buy outside medicine 15. Uterine massage 16. Did a C-section 17. Did another operation *(specify)* 18. Admitted to hospital 19. Other *(specify)* 20. Nothing   99.Don’t know | | S4.28  1. □  2. □  3. □  4. □  5. □ _______________  6. □  7. □  8. □  9. □  10. □  11. □  12. □  13. □  14. □  15. □  16. □  17. □ ______________  18. □ stayed __ __ days  19. □ ______________  20. □ ***→ SQ4.30***  99. □ ***→ SQ4.30*** | | S4.38  1. □  2. □  3. □  4. □  5. □_______________  6. □  7. □  8. □  9. □  10. □  11. □  12. □  13. □  14. □  15. □  16. □  17. □ ______________  18. □ stayed __ __ days  19. □ ______________  20. □ ***→ SQ4.40***  99. □ ***→ SQ4.40*** | |
| How much did (you / the mother) pay for these treatments and other costs related to the health care, including any admission fee, consultation, lab tests, equipment, and room and food for companions?  Nawa (ki ka/mahaifiyar ta) biya domin amsar magani da sauran kudaden kiwon lafiya da suka hada da kudin kati, dubawa, gwaji, kudin daki da abincin majinyata ko ‘yanuwa?. | | | | S4.29  **__ __ __ __ __** unit  *(DK = 99999)* | | S4.39  **__ __ __ __ __** unit  *(DK = 99999)* | |
| Did the <FIRST/LAST HEALTH PROVIDER> refer (you / the mother) to another health provider or facility?  Shin ko a<FIRST/LAST HEALTH PROVIDER> ya/ta tura (ki/mahaifiyar) zuwa wajen wata cibiyar kiwon lafiya? | | 1. Yes 2. No   9. Don’t know | | S4.30  🞎 ***2 or 9 → SQ4.30.2*** | | 4.40  🞎 ***2 or 9 → SQ4.40.2*** | |
| Why (were you / was the mother) referred?  *Shin menene ya sa aka tura (ki/mahaifiyar) wata cibiyar kiwon lafiya?*  *[Multiple answers allowed.]* | | 1. The provider was not capable of managing the problem 2. Required supplies (e.g., drugs, IV, oxygen, blood) not available 3. Required equipment (e.g., ultrasound) not available 4. Required facility (e.g., operation room) not available   9. Don’t know | | S4.30.1  1. □  2. □  3. □  4. □  9. □ | | S4.40.1  1. □  2. □  3. □  4. □  9. □ | |
| Was the baby delivered at the <FIRST/LAST HEALTH PROVIDER>?  An haifi jaririn a <FIRST/LAST HEALTH PROVIDER>? | | 1. Yes 2. No   9. Don’t know | | S4.30.2  🞎 ***1 → Inst_8*** | | S4.40.2  🞎 ***1 → Inst_8*** | |
| ***Inst_6: Check SQ4.18 to determine if she went to another health provider*** | | | | | |  | |
| *If did not go to another health provider, ask:* Did (you / the mother) have any concerns or problems that kept (you / her) from going to another provider?  Shin (kin/mahaifiyar ta ) samu uzurori ko lalluran da ya sa ba (ki/ta) je wani jami’in/cibiyar kiwon lafiya ba?  *If went to another health provider, ask:* Did (you / the mother) have to overcome any concerns or problems to go to another provider?  Shin (kin/mahaifiyar ta) ciwo kan wasu uzurori ko lalluran kafin (ki/ta) je wani jami’in/cibiyar kiwon lafiya ? | | 1. Yes 2. No   9. Don’t know | | S4.31  🞎***2 or 9 → Inst_7*** | | S4.41  🞎***2 or 9 → Inst_8*** | |
| What concerns or problems did (you / she) have?  Shin menene uzurori ko lalluran da (ki ka /mahaifiyar ta) samu?  *Prompt:* Was there anything else?  *Da akwai wani abun kuma?*  *[Multiple answers allowed.]* | | 1. Thought no more care needed 2. No one available to go with her 3. Too much time from regular duties 4. Someone else *(specify)* decided 5. Too far to travel 6. No transportation available 7. Cost (transport, health care, other) 8. Not satisfied with available care 9. Problem required traditional care 10. Thought too sick to travel 11. Thought she/baby will die anyway 12. Was late at night 13. She delivered before going 14. Other *(specify)*   99. Don’t know | | S4.31.1  1. □  2. □  3. □  4. □ ______________  5. □  6. □  7. □  8. □  9. □  10. □  11. □  12. □  13. □ ***→ Inst_8***  14. □ ______________  99. □ | | S4.41.1  1. □  2. □  3. □  4. □ ______________  5. □  6. □  7. □  8. □  9. □  10. □  11. □  12. □  13. □  14. □ ______________  99. □ | |
| ***Inst_7: Check SQ4.18 → If she went to another health provider*** | | | | ***…go to SQ4.32 (LAST HEALTH PROVIDER)*** | |  | |
| ***Inst_8: STOP – If VQ1.15 = 1 (Stillbirth) → VQ5.4 (Section 5: Health records)*** | | | | | | | |

| **SA Module 5a: Care of the newborn; and VA Section 3: Neonatal deaths (FOR NN DEATHS <28 DAYS OLD)**  *Read:* Now I would like to ask you about the care of the newborn child.  Yanzu ina son in tambaye ki gameda kulawar da jaririn ya /ta samu. | | | | | | | |
| --- | --- | --- | --- | --- | --- | --- | --- |
| S5a.1 | What tool was used for cutting the cord?  Da menene aka yanke cibiyar? | 1. New/from delivery kit/boiled razor blade 2. Old razor blade 3. Scissors 4. Other *(specify)*   9. Don’t know | 🞎  ___________________________ | | | | |
| S5a.2 | What material was used for tying the cord?  Da menene aka daure cibiyar? | 1. Clean/from delivery kit/boiled piece of thread 2. Unclean piece of thread 3. Cord clamp 4. Other *(specify)*   9. Don’t know | 🞎  ___________________________ | | | | |
| S5a.3 | Was anything applied to the umbilical cord stump after birth?  Shin an shafa wani abu akan cibiyar bayan an yanke ? | 1. Yes 2. No   9. Don’t know | 🞎 ***2 or 9 → VQ3.1*** | | | | |
| S5a.3.1 | What was it?  Menene aka sa? | 1. Alcohol/other antiseptic 2. Antibiotic ointment/cream/powder 3. Castor oil, mustard oil or ghee 4. Animal dung or dirt/mud/ash 5. Other *(specify)*   9. Don’t know | 🞎  ___________________________ | | | | |
| V3.1 | Were there any bruises or signs of injury on the baby’s body at birth?  Akwai wasu alamomin jin ciwo ko rauni a jikin ‘da/’yar lokacin haihuwa? | 1. Yes 2. No   9. Don’t know | 🞎 | | | | |
| V3.2 | Was any part of the baby physically abnormal at the time of delivery? (for example: body part too large or too small, additional growth on body)  Akwai wani nakasu a jikin ‘dan/ ‘yar lokacin haihuwar? (Misali: wani sashe daga jikin ya yi girma ko kankanta, ko wani abu ya fito/tsira a jikin? | 1. Yes 2. No   9. Don’t know | 🞎 ***2 or 9 → VQ3.4*** | | | | |
| V3.3 | What were the abnormalities?  *Shin menene nakasu?*  *Ask for the following abnormalities:*  *[Mark all that apply – Show photos]* | 1. Was the head size very small at the time of birth? Shin kan yayai kankanta sosai a lokacin haihuwar.? 2. Was the head size very large at the time of birth? Shin kan yayai girma sosai a lokacin haihuwar.? 3. Was there a mass defect on the back of head or spine? Shin da akwai nakasa a keyan ko a gadon bayan? 4. Was there any other abnormality? Shin d akawi wata nakassan kuma?   *(If “Yes,” then specify)* | Yes No  1. □ 2. □    1. □ 2. □  1. □ 2. □  1. □ 2. □  ____________________________ | | | | |
| V3.4 | Did the baby breathe immediately after birth?  ‘Dan/ ‘Yar ta/ya yi nunfashii daidai bayan haihuwa? | 1. Yes 2. No   9. Don’t know | 🞎 ***2 → VQ3.6*** | | | | |
| V3.5 | Did the baby have difficulty breathing?  ‘Dan/’yar ta/ya samu matsalar nunfashi? | 1. Yes 2. No   9. Don’t know | 🞎 | | | | |
| V3.6 | Was anything done to try to help the baby breathe at birth?  Akwai kokarin da aka yi domin taimaka ma jaririn yin nunfashi? | 1. Yes 2. No   9. Don’t know | 🞎 | | | | |
| V3.7 | Did the baby cry immediately after birth?  ‘Dan/ ‘Yar ta/ya yi kuka daidai bayan haihuwa? | 1. Yes 2. No   9. Don’t know | 🞎 ***1 → VQ3.9*** | | | | |
| V3.8 | How long after birth did the baby first cry?  *Tsawon wane lokaci jaririn ya dauka kafin ya yi kukan farko?*  *[Mark ONE response]* | 1. Within 5 minutes 2. Within 6-30 minutes 3. More than 30 minutes 4. Never   9. Don’t know | 🞎 ***4 → SQ5a.4*** | | | | |
| V3.9 | Did the baby stop being able to cry?  Akwai lokacin da jaririn ya/ta kasa kuka? | 1. Yes 2. No   9. Don’t know | 🞎 ***2 or 9 → SQ5a.4*** | | | | |
| V3.10 | How long before the baby died did the baby stop crying?  Kafin rasuwar jaririn tsawon wanne lokacin ne ya daina kuka? | 1. Less than one day 2. One day or more   9. Don’t know | 🞎 | | | | |
| S5a.4 | How long after birth was the baby first bathed?  Har tsawon wanne lokaci ne bayan haihuwa aka yi ma jaririn wankan farko? | 1. Less than 1 hour 2. 1-23 hours 3. 24-72 hours (1-3 days) 4. More than 72 hours (3 days) 5. Not bathed   9. Don’t know | 🞎 | | | | |
| S5a.5 | Was anything done to keep the baby warm on the first day after birth?  Menene aka yi domin a dunduma jikin jaririn ranar da aka haifeshi? | 1. Yes 2. No   9. Don’t know | 🞎 ***2 or 9 → SQ5a.6*** | | | | |
| S5a.5.1 | What was done?  Menene aka yi?  *[Multiple answers allowed.]*  *For each mentioned, ask:*  How soon after birth was this done?  shin tsawon wane lokaci bayan haihuwar aka yi hakan? | 1. Dried/wiped 2. Wrapped in a blanket 3. Skin-to-skin contact 4. Incubator 5. Other   *(specify other)* | Done  1. □  2. □  3. □  4. □  5. □ | | How soon after birth  <1hr <6 6-24 >24 DK  1.□ 2.□ 3.□ 4.□ 9.□ 1.□ 2.□ 3.□ 4.□ 8.□  1.□ 2.□ 3.□ 4.□ 9.□  1.□ 2.□ 3.□ 4.□ 9.□  1.□ 2.□ 3.□ 4.□ 9.□ | | |
|  |  |  | ____________________________ | | | | |
| S5a.6 | Did (you / the mother) or a wet nurse ever breastfeed the baby?  Shin (kin/mahaifiyar ta) ko mai shayarwa ta, taba shayar da jaririn?. | 1. Yes 2. No   9. Don’t know | 🞎***2 or 9 → SQ5a.7*** | | | | |
| S5a.6.1 | How long after birth was the baby first put to the breast?  Har tsawon wane lokaci ne bayan haihuwar aka fara shayar da jaririn nono?  *[If immediately or less than 1 hour, record ’00’ hours.]*  *[If less than 24 hours, record hours; otherwise record days.]* | | __ __ Days  *(DK = 99)*  OR | | | | |
|  |  |  | __ __ Hours  *(DK = 99)* | | | | |
| S5a.6.2 | Was the baby being breastfed at the time when the fatal illness began?  Ana shayar da jaririn nono a lokacin da mummunan rashin lafiyar ya fara? | 1. Yes 2. No   9. Don’t know | 🞎 | | | | |
| S5a.7 | At the time the fatal illness began, was the baby being given any other liquid, including non-human milk or formula, fruit juice, tea or water, or any semisolid or soft foods such as cereal?  *[Multiple answers allowed. Probe, and record all liquids and foods given.]*  *A lokacin da mummunan rashin lafiyar ya fara, an ba jaririn wani abincin mai ruwa-ruwa da ya hada da madarar gwangwani ko sanadari,ruwan ‘ya’yan itatuwa,shayi ko ruwa, ko wani abinci mai dan kauri ko laushi kamar tsaiwa ?*    *Multiple answers allowed. Probe, and record all liquids and foods given.]* | 1. Non-human milk or pre-mixed formula 2. Powdered formula mixed with a liquid 3. Juice, water and/or water-based drinks 4. ORS 5. Drops or syrups (vitamins, medicines) 6. Semi-solid or soft foods 7. Nothing else, only given breast milk   9. Don’t know | 1. □  2. □  3. □  4. □  5. □  6. □  7. □  9. □ | | | | |
| V3.11 | Was the baby able to suckle in a normal way during the first day of life?  Shin Jaririn yana shan nono yadda ya kamata ranar da aka haife shi/ta? | 1. Yes 2. No   9. Don’t know | 🞎 ***1 → VQ3.13*** | | | | |
| V3.12 | Did the baby ever suckle in a normal way?  Shin Jaririn ya taba shan nono yadda ya kamata? | 1. Yes 2. No   9. Don’t know | 🞎 ***2 or 9 → VQ3.17*** | | | | |
| V3.13 | Did the baby stop being able to suckle in a normal way?  Shin Jaririn ya daina shan nono yadda ya kamata? | 1. Yes 2. No   9. Don’t know | 🞎 ***2 or 9 → VQ3.17*** | | | | |
| V3.14 | How long after birth did the baby stop suckling?  *Har tsawon wanne lokaci ne bayan haihuwar jaririn ya daina shan nono?*  *[Less than 24 hours = “00” days]* | | **__ __** Days  *(DK = 99)* | | | | |
| V3.15 | How long before s/he died did the baby stop suckling?  Shin har tsawon wane lokaci ne kafin rasuwar sa/ta , jaririn ya/ta daina shan nono? | 1. Less than one day 2. One day or more   9. Don’t know | 🞎 | | | | |
| V3.16 | Was the baby able to open her/his mouth at the time s/he stopped suckling?  Jaririn ya/ta na iya bude baki a lokacin da ya/ta daina shan nonon? | 1. Yes 2. No   9. Don’t know | 🞎 | | | | |
| V3.17 | During the illness that led to death, did the baby have difficult breathing?  Lokacin rashin lafiyar daya kawo rasuwar, jaririn ya/ta samu matsalar nunfashi? | 1. Yes 2. No   9. Don’t know | 🞎 ***2 or 9 → VQ3.20*** | | | | |
| V3.18 | At what age did the difficult breathing start?  *[Less than 24 hours = “00” days]*  *A wanne lokaci ko a shekara nawa ne nunfashin mai wahalar ya fara?* | | **__ __** Days  *(DK = 99)* | | | | |
| V3.19 | For how many days did the difficult breathing last?  *Shin kwanaki nawa ne aka yi da matsalar nunfashin?*  *[Less than 24 hours = “00” days]* | | **__ __** Days  *(DK = 99)* | | | | |
| V3.20 | During the illness that led to death, did the baby have fast breathing?  Lokacin rashin lafiyar da yayi sanadiyyar rasuwar, ko jaririn na nunfashi akai-akai? | 1. Yes 2. No   9. Don’t know | 🞎 ***2 or 9 → VQ3.23*** | | | | |
| V3.21 | At what age did the fast breathing start?  *[Less than 24 hours = “00” days]*  *A wanne lokacin ko a shekara nawa ne ya/ta fara nunfashin akai-akai?* | | **__ __** Days  *(DK = 99)* | | | | |
| V3.22 | For how many days did the fast breathing last?  *[Less than 24 hours = “00” days]*  *Har kwana nawa ne ya/ta yi nunfashin akai-akai?* | | **__ __** Days  *(DK = 99)* | | | | |
| V3.23 | During the illness that led to death, did the baby have indrawing of the chest?  *[Show photo]*  *Lokacin rashin lafiyar da tayi sanadiyyar rasuwar, ko jaririn ya/ta samu fadawar kirjin sa/ta?*  *[Show photo]* | 1. Yes 2. No   9. Don’t know | 🞎 | | | | |
| V3.24 | During the illness that led to death, did the baby have grunting?  *Lokacin rashin lafiyar da tayi sanadiyyar rasuwar, ko jaririn yana shakuwa/kakari?*  *[Demonstrate grunting]* | 1. Yes 2. No   9. Don’t know | 🞎 | | | | |
| V3.25 | During the illness that led to death, did the baby have spasms or convulsions?  *Lokacin rashin lafiyar da tayi sanadiyyar rasuwar, ko jaririn yana tsinka - tsinka?* | 1. Yes 2. No   9. Don’t know | 🞎 | | | | |
| V3.26 | During the illness that led to death, did the baby have fever?  *Lokacin rashin lafiyar da tayi sanadiyyar rasuwar, jaririn ya/ta na zazzabi?* | 1. Yes 2. No   9. Don’t know | 🞎 ***2 or 9 → VQ3.29*** | | | | |
| V3.27 | At what age did the fever start?  *A wanne lokacin ko a shekara nawa ne ya/ta fara zazzabin?*  *[Less than 24 hours = “00” days]* | | **__ __** Days  *(DK = 99)* | | | | |
| V3.28 | How many days did the fever last?  *Zazzabin yakai Har tsawon kwana nawa ne ?*  *[Less than 24 hours = “00” days]* | | **__ __** Days  *(DK = 99)* | | | | |
| V3.29 | During the illness that led to death, did the baby become cold to touch?  *Lokacin rashin lafiyar da tayi sanadiyyar rasuwar, jikin jaririn ya yi sanyi sosai in an taba?* | 1. Yes 2. No   9. Don’t know | 🞎 ***2 or 9 → VQ3.32*** | | | | |
| V3.30 | At what age did the baby start feeling cold to touch?  *A wanne lokacin ko a shekara nawa ne jikin jaririn ya/ta fara sanyi idan an taba?*  *[Less than 24 hours = “00” days]* | | **__ __** Days  *(DK = 99)* | | | | |
| V3.31 | How many days did the baby feel cold to touch?  *Har tsawon kwana nawa ne jikin jaririn yake da sanyi idan an taba?*  *[Less than 24 hours = “00” days]* | | **__ __** Days  *(DK = 99)* | | | | |
| V3.32 | During the illness that led to death, did the baby become lethargic, after a period of normal activity?  *Lokacin rashin lafiyar da tayi sanadiyyar rasuwar, jikin jaririn bashi da kuzari bayan ya/ta yi ko dan wasa?* | 1. Yes 2. No   9. Don’t know | 🞎 | | | | |
| V3.33 | During the illness that led to death, did the baby become unresponsive or unconscious?  *Lokacin rashin lafiyar da tayi sanadiyyar rasuwar, jikin jaririn bashi jin motsi ko ya suma?* | 1. Yes 2. No   9. Don’t know | 🞎 | | | | |
| V3.34 | During the illness that led to death, did the baby have a bulging fontanelle?  A yayin rashin lafiyar da ta yi sanadiyar rasuwar, shin madigan jaririn ya kumburi?  *[Show photo]* | 1. Yes 2. No   9. Don’t know | 🞎 | | | | |
| V3.35 | During the illness that led to death, did the baby have pus drainage from the umbilical cord stump?  *Lokacin rashin lafiyar da tayi sanadiyyar rasuwar, ruwan gyanbo na fitowa daga cibiyar jaririn?* | 1. Yes 2. No   9. Don’t know | 🞎 | | | | |
| V3.36 | During the illness that led to death, did the baby have redness of the umbilical cord stump?  *Lokacin rashin lafiyar da tayi sanadiyyar rasuwar, cibiyar jaririn tayi jajir?* | 1. Yes 2. No   9. Don’t know | 🞎 ***2 or 9 → VQ3.38*** | | | | |
| V3.37 | Did the redness of the umbilical cord stump extend onto the abdominal skin?  Yanayin yadda cibiyar tayi ja ya shafi fatar cikin ? | 1. Yes 2. No   9. Don’t know | 🞎 | | | | |
| V3.38 | During the illness that led to death, did the baby have skin bumps containing pus or a single large area with pus?  *Lokacin rashin lafiyar da tayi sanadiyyar rasuwar, jaririn ya/ta na da kuraje dake fidda ruwa ko wanni babban gyanbo mai fidda ruwan gyanbo?* | 1. Yes 2. No   9. Don’t know | 🞎 | | | | |
| V3.39 | During the illness that led to death, did the baby have ulcer(s) (pits)?  *Lokacin rashin lafiyar da tayi sanadiyyar rasuwar, jaririn ya/ta na da (ramukan) gyanbo/(gyanbuna) ?* | 1. Yes 2. No   9. Don’t know | 🞎 | | | | |
| V3.40 | During the illness that led to death, did the baby have an area(s) of skin with redness and swelling?  *Lokacin rashin lafiyar da tayi sanadiyyar rasuwar, jaririn ya/ta na da wani ko wadansu sashen jiki da yayi ja kuma ya kumbura?* | 1. Yes 2. No   9. Don’t know | 🞎 | | | | |
| V3.41 | During the illness that led to death, did s/he have areas of the skin that turned black?  *Lokacin rashin lafiyar da tayi sanadiyyar rasuwar, jaririn ya/ta na da wani sashen jiki da ya canja launi zuwa baki?* | 1. Yes 2. No   9. Don’t know | 🞎 | | | | |
| V3.42 | During the illness that led to death, did the baby bleed from anywhere?  *Lokacin rashin lafiyar da tayi sanadiyyar rasuwar, jaririn ya/ta na da wani sashen jiki da yake fidda jini?* | 1. Yes 2. No   9. Don’t know | 🞎 ***2 or 9 → VQ3.44*** | | | | |
| V3.43 | Record from where did the baby bleed: |  | | | | | |
| V3.44 | During the illness that led to death, did s/he have more frequent loose or liquid stools than usual?  *Lokacin rashin lafiyar da tayi sanadiyyar rasuwar, jaririn ya/ta na kashi akai-akai ko ruwa-ruwa da bai saba ba ?* | 1. Yes 2. No   9. Don’t know | 🞎 ***2 or 9 → VQ3.46*** | | | | |
| V3.45 | How many stools did the baby have on the day that diarrhea/loose liquid stools were most frequent?  Kamar sau nawa jaririn ya/ta yi kashi a rana da gudawar/ zawo akai-akai? | | **__ __** Stools  *(DK = 99)* | | | | |
| V3.46 | During the illness that led to death, did s/he vomit everything?  *Lokacin rashin lafiyar da tayi sanadiyyar rasuwar, jaririn ya/ta amayyar da komai ?* | 1. Yes 2. No   9. Don’t know | 🞎 | | | | |
| V3.47 | During the illness that led to death, did s/he have yellow skin?  *Lokacin rashin lafiyar da tayi sanadiyyar rasuwar, launin jikin jaririn ya canja zuwa ruwan dorawa?*  *Lokacin rashin lafiyar da tayi sanadiyyar rasuwar, launin jikin jaririyar ya canja zuwa ruwan dorawa?* | 1. Yes 2. No   9. Don’t know | 🞎 | | | | |
| V3.48 | During the illness that led to death, did the baby have yellow eyes?  *Lokacin rashin lafiyar da tayi sanadiyyar rasuwar, launin idon jaririn ya/ta canja zuwa ruwan dorawa?* | 1. Yes 2. No   9. Don’t know | 🞎 | | | | |
| V3.49 | Did the infant appear to be healthy and then just die suddenly?  Shin jaririn ya/ta yi kamar mai koshin lafiya kafin kawai ya/ta rasu? | 1. Yes 2. No   9. Don’t know | 🞎 | | | | |
| S5a.8 | *Check SQ4.17 to determine if the baby was born in a health facility (codes 1-2):* | 1. Yes, born in a health facility 2. Not born in a health facility   9. Don’t know | 🞎***2 or 9 → SQ5a.10*** | | | | |
| S5a.8.1 | Did the baby leave the delivery facility alive or did s/he die in the facility?  Shin an bar cibiyar kiwon lafiya da jaririn da rai ko ya/ta rasu kafin abar cibiyar kiwon lafiyar? | 1. Yes, left alive 2. Died in the facility   9. Don’t know | 🞎***2 or 9 → SQ6.1*** | | | | |
| S5a.8.2 | How soon after birth did the baby leave the facility?  *Har tsawon wanne lokaci ne bayan haihuwa kafin jaririn ya/ta bar cibiyar kiwon*  *lafiya ?*  *[Record hours if less than 24 hours—if less than 1 hour, record ‘00’ hours; Record days if 1 day or more.]* | | **__ __** Days  *(DK = 99)*  OR | | | | |
|  |  |  | **__ __** Hours  *(DK = 99)* | | | | |
| S5a.8.3 | Was the child examined by a health worker prior to discharge?  Shin jami’in kiwon lafiya ya/ta duba jaririn kafin a sallame su daga cibiyar kiwon lafiya? | 1. Yes 2. No   9. Don’t know | 🞎 | | | | |
| S5a.9 | Did (you / the mother) receive any counselling by a health worker prior to discharge?  Shin (kin/mahaifiyar ta) samu wani bayani daga jami’in kiwon lafiya kafin sallama daga cibiyar kiwon lafiyar? | 1. Yes 2. No   9. Don’t know | 🞎***2 or 9 → SQ5a.10*** | | | | |
| S5a.9.1 | What (were you / was she) counselled on?  Shin akan menene aka yi (maki/mata) bayanin?  *[Multiple answers allowed].*  *Probe:* Anything else?  Da akwai wani abun kuma? | 1. Breastfeeding 2. Immunization 3. Post-natal care attendance 4. Danger signs of newborn illness 5. Other (specify)   9. Don’t know | 1. □  2. □  3. □  4. □  5. □ ________________________  9. □ | | | | |
| S5a.10 | Was the baby ever seen by a health worker or nurse at home or in the community, or by a doctor or nurse at a health facility before the fatal illness began?  Ko wani jami’in kiwon lafiya ko malamar asibiti ya/ta taba ganin jaririn a gida ko kuma Likitaa ko malamar asibiti a cibiyar kiwon lafiya kafin mummunar rashin lafiyar?  *[Multiple answers allowed.]*  *For each mentioned, ask:*  How many times was the baby seen by a <PROVIDER TYPE at PLACE> before the fatal illness began?  Har sau nawa aka gan jaririn a <PROVIDER TYPE at PLACE> kafin mummunar rashin lafiyar ya fara?  *Then ask:*  When was the baby first seen by (this / any of these) provider(s)?  Shin yaushe wannan /wani cikin wadannan jami’an ya/ta fara ganin jaririn? | 1. CHW or nurse at home/in community 2. Doctor or nurse at a health facility 3. Never seen   9. Don’t know | Seen  1. □  2. □  3. □  9. □ | Times  __ __  __ __ | | | First visit  __ __  Days old  *(<1 = 00;*  *DK = 99)* |
| S5a.11 | Before the fatal illness began, did <NAME> suffer from any of the following known conditions:  Kafin farawar mummunar rashin lafiyar, ko <NAME> ya/ta yi fama da daya daga cikin wadannan lallurorin?  *[Read out all conditions and check “Yes,” “No” or “Don’t know” for each.]*  *If “Yes,” then ask:* Was s/he provided any treatment for this condition?  Shin an ba shi/ta magani domin wannan lallurar? | 1. Preterm birth Haihuwan bakwai ni    1. Was s/he given special nutrition? Shin an ba ta/ shi abin sha na musamman?    2. Was s/he given “kangaroo care”? An ba ta /shi kula wa na musamman na dunduma wa a jiikin babba? 2. Malformation (from the time of birth): Nakasa tun daga haihuwa?    1. Head, neck and/or back Kai, Wuya da/ko baya.    2. Mouth/palate Baki /cikin bakin    3. Heart Zuciya    4. Arms and/or legs hannaye da/ko kafafu 3. Other   *(specify other)* | Suffered from  Yes No DK  1. □ 2. □ 9. □      1. □ 2. □ 9. □  1. □ 2. □ 9. □  1. □ 2. □ 9. □  1. □ 2. □ 9. □  1. □ 2. □ 9. □ | | | Treatment  Yes No DK  1. □ 2. □ 9. □  1. □ 2. □ 9. □  1. □ 2. □ 9. □  1. □ 2. □ 9. □  1. □ 2. □ 9. □  1. □ 2. □ 9. □  1. □ 2. □ 9. □ | |
|  |  |  | _____________________________ | | | | |
| ***Inst_1: STOP – If VQ1.26 = 1 (Neonatal death) →*** ***SQ6.1*** | | | | | | | |

| **SA Module 5b: Preventive care of post-neonates (FOR CHILD DEATHS 28 DAYS—59 MONTHS OLD)**  *Read:* Now I would like to ask you about the care of the child before the fatal illness began.  Yanzu ina son in tambayeki gameda kular da aka yi wa dan/’yar kafin mummunar rashin lafiyar? | | | | | | | | |
| --- | --- | --- | --- | --- | --- | --- | --- | --- |
| S5b.1 | | Where (do you / does the mother) cook?  A wanne wuri (ki /mahaifiyar ta) ke dafa abinci? | | - 1. Inside the house   2. Outside the house   3. In a structure outside the house   9. Don’t know | | 🞎 | | |
| S5b.2 | | When (you / the mother) cooked, was <NAME> usually beside or carried by (you / her)?  Shin lokacin da (ki/mahaifiyar ta) ke dafa abinci, <NAME> yawanji ya/ta na gefen (ki/ta) ne ko ana goya shi/ta? | | 1. Yes 2. No   9. Don’t know | | 🞎 | | |
| S5b.3 | | *Skip SQ5b.3 in areas wo/malaria.*  Before (her / his) fatal illness began, did <NAME> sleep under an insecticide treated bednet?  Kafin mummunar rashin lafiyar ta fara, ko <NAME> ya/ta na kwanciya cikin gidan sauro mai magani? | | 1. Yes, usually or always 2. Yes, sometimes 3. Never   9. Don’t know | | 🞎 | | |
| S5b.4 | | Did (you / the mother) or a wet nurse ever breastfeed <NAME>?  Shin (kin/mahaifiyar ta) taba, ko wata mai shayar da nono ta, taba shayar da <NAME> nono? | | 1. Yes 2. No   9. Don’t know | | 🞎***2 or 9 → SQ5b.5*** | | |
| S5b.4.1 | | Was <NAME> being breastfed at the time (her / his) fatal illness began?  Shin ko ana shayar da<NAME> nono lokacin da mummunar rashin lafiyar na ta /shi ya fara? | | 1. Yes 2. No   9. Don’t know | | 🞎***1 or 9 → SQ5b.5*** | | |
| S5b.4.2 | | How old was <NAME> when s/he was last breastfed?  Shin Shekarar <NAME> nawa lokacin da aka shayar da shi/ita nonon na karshe ? | | | | **__ __** Months  *(<1 = 00; DK = 99)* | | |
| S5b.5 | | At the time the fatal illness began, was <NAME> being given any other liquid, including non-human milk or formula, fruit juice, tea or water, or any solid, semisolid, or soft foods?  *A lokacin da mummunan rashin lafiyar ya fara, an ba* <NAME> *wani abincin mai ruwa-ruwa, da ya hada da madarar gwangwani ko sanadari,ruwan ‘ya’yan itatuwa,shayi ko ruwa, ko wani abinci mai dan kauri ko taushi/ laushi?*  *[Multiple answers allowed. Probe, and record all liquids and foods given.]* | | 1. Non-human milk or pre-mixed formula 2. Powdered formula mixed with a liquid 3. Juice, water and/or water-based drinks 4. ORS 5. Drops or syrups (vitamins, medicines) 6. Solid, semi-solid or soft foods 7. Nothing else, only given breast milk   9. Don’t know | | 1. □  2. □  3. □ ***SQ5b.6***  4. □  5. □  6. □  7. □  ***SQ5b.6***  9. □ | | |
| S5b.5.1 | | On most days before the illness began, how many times did <NAME> eat solid, semisolid, or soft foods other than liquids during the day or night?  Akasarin kwanaki, kafin rashin lafiyar ya fara, sau nawa <NAME> ya/ta ci abinci mai kauri sosai ko mai dan kauri ko mai laushi/taushi wanda ba ruwa-ruwa bane da rana ko dadaddare? | | | | __ __ Times  *(DK = 99)* | | |
| S5b.5.2 | | Which of the following food types did <NAME> typically eat every day?  *Shin cikin waddan nan abincin wadan ne ne* <NAME> ya/ta kan ci a kullum?  *[Read out all options and check “Yes,” “No” or “Don’t know” for each.]* | | 1. Grains, roots and tubers 2. Legumes and nuts 3. Dairy products (milk, yogurt, cheese) 4. Flesh foods (meat, fish, poultry, organs) 5. Eggs 6. Vitamin-A rich fruits and vegetables 7. Other fruits and vegetables | | Yes No DK  1. □ 2. □ 9. □  1. □ 2. □ 9. □  1. □ 2. □ 9. □  1. □ 2. □ 9. □  1. □ 2. □ 9. □  1. □ 2. □ 9. □  1. □ 2. □ 9. □ | | |
| S5b.6 | | Did <NAME> drink any liquids or semi-solid foods from a bottle with a nipple or teat?  Shin ko <NAME> ya/ta kan ci wani abu mai ruwa-ruwa ko mai kauri daga kwalbar shayarda jarirai ? | | 1. Yes 2. No   9. Don’t know | | 🞎 | | |
| S5b.7 | | Now I would like to ask about the chlid’s vaccinations. Do you have a card where <NAME>’s vaccinations are written down?  Yanzu ina son in yi maki tambaya akan allurar rigakafin yara. Kina da katin da aka yi ma <NAME> allurar rigakafi?  *If “Yes,” ask:* May I see it please?  Zan iya gani? | | 1. Yes, seen 2. Yes, but not seen 3. No card | | 🞎 ***2 or 3 → SQ5b.8*** | | |
| S5b.7.1 | | Did <NAME> receive any vaccinations that are not included on this card, including vaccinations received in a national immunization day campaign?  *Akwai wata allurar rigakafi da aka ba <SUNA> wanda ba a rubuta ba cikin katin asibitin sa/ta, misali allurar rigakafi na kasa da aka kaddamar da gangamin sa?*  *If “Yes,” probe for vaccinations received but not recorded on the card.*  *[Record ‘Yes’ only if BCG, Polio 0-3 , DPT 1-3 or PENTA 1-3, Measles, Yellow Fever and/or Hepatitis B1-3 vaccine(s) mentioned.]* | | 1. Yes (received BCG, Polio 0-3, DPT 1-3 or PENTA 1-3, Measles, Yellow Fever and/or Hep B1-3 vaccinations that are not recorded on the card) 2. No   9. Don’t know | | 🞎 ***1 → Write ‘66’ in the corresponding day column below for each vaccination received but not recorded on the card.*** | | |
|  |  | *Copy vaccination date for each vaccine from the card. Record “99” or “9999” for partially unknown dates.*  *Write ‘88’ in ‘day’ column if card shows that a vaccination was given, but no date is recorded.*  *Do not leave any rows blank. Record “00” in the ‘day’ column for each vaccination that was not given.* | | BCG  POLIO 0 (given at birth)  POLIO 1  POLIO 2  POLIO 3  DPT 1 / PENTA 1  DPT 2 / PENTA 2  DPT 3 / PENTA 3  MEASLES  YELLOW FEVER  HEPATITIS B1  HEPATITIS B2  HEPATITIS B3 | \|  \|  \|  \|  \|  \|  \|  \|  \| BCG \| \| --- \| --- \| --- \| --- \| --- \| --- \| --- \| --- \| --- \| \|  \|  \|  \|  \|  \|  \|  \|  \| P0 \| \|  \|  \|  \|  \|  \|  \|  \|  \| P1 \| \|  \|  \|  \|  \|  \|  \|  \|  \| P2 \| \|  \|  \|  \|  \|  \|  \|  \|  \| P3 \| \|  \|  \|  \|  \|  \|  \|  \|  \| DPT1 \| \|  \|  \|  \|  \|  \|  \|  \|  \| DPT2 \| \|  \|  \|  \|  \|  \|  \|  \|  \| DPT2 \| \|  \|  \|  \|  \|  \|  \|  \|  \| MSL \| \|  \|  \|  \|  \|  \|  \|  \|  \| YLFV \| \|  \|  \|  \|  \|  \|  \|  \|  \| HEP1 \| \|  \|  \|  \|  \|  \|  \|  \|  \| HEP2 \| \|  \|  \|  \|  \|  \|  \|  \|  \| HEP3 \|   Day Month Year | | | |
| S5b.8 | | Did <NAME> ever receive any vaccinations to prevent her/him from getting diseases, including vaccinations received in a national immunization day campaign?  An taba yiwa <SUNA> wani rigakafi da zai hana ta/shi kamuwa da cututuka harda allurar rigakafin da aka karba a ranar gangamin kaddamar da rigakafi na kasa? | | 1. Yes 2. No   9. Don’t know | | 🞎 ***2 or 9 → SQ5b.10*** | | |
|  |  | Please tell me if <NAME> received any of the following vaccinations:  Ki fada mini idan <SUNA> ya /ta taba karban daya daga cikin wadannan rigakafin? | |  | | | | |
|  | .1 | A BCG vaccination against tuberculosis, that is, an injection in the arm or shoulder that usually causes a scar?  Allurar BCG, wadda zai kare shi  ko kare ta daga kamuwa da tarin fuka wato irin allurar da ake yi, a kafada ko a dantse wadda ke sa tabo? | | 1. Yes 2. No   9. Don’t know | | 🞎 | | |
|  | .2 | Polio vaccine, that is, drops in the mouth?  Allurar shan inna wadda ake digawa a baki? | | 1. Yes 2. No   9. Don’t know | | 🞎***2 or 9 → SQ5b.8.5*** | | |
|  | .3 | When was the first polio vaccine received, just after birth or later?  Yaushe ne aka yi allurar shan inna (polio), daidai bayan haihuwa ko can daga baya? | | 1. Just after birth 2. Later   9. Don’t know | | 🞎 | | |
|  | .4 | How many times was the polio vaccine received?  Sau nawa akayi allurar shan inna (polio)? | | | | **__ __** Times  *(DK = 99)* | | |
|  | .5 | A DPT vaccination, that is, an injection given in the thighs or buttocks, sometimes at the same time as polio drops?  Rigakafin DPT irin wanda a ke allurar a cinya/duwawu wani lokaci daidai lokacin da akan yi allurar shan inna? | | 1. Yes 2. No   9. Don’t know | | 🞎***2 or 9 → SQ5b.8.7*** | | |
|  | .6 | How many times?  Sau nawa aka yi? | | | | **__ __** Times  *(DK = 99)* | | |
|  | .7 | A measles or MMR injection, that is, a shot in the arm at the age of 9 months or older, to prevent measles?  Allurar Kyanda (bakon dauro) (MMR injection), watau lamba a hannu a wata 9 ko fiye da haka domin kariya daga cutar Kyanda (bakon dauro)? | | 1. Yes 2. No   9. Don’t know | | 🞎 | | |
|  | .75 | A yellow fever vaccination, that is, an injection given in the arm after the child is 9 months old? | | 1. Yes 2. No   9. Don’t know | | 🞎 | | |
|  | .8 | A Hep B vaccination, that is, an injection in the right thigh, sometimes given at the same time as DPT?  Rigakafin HEP B irin wanda a ke allurar a cinya/duwawu daidai lokacin da akan yi allurar DPT? | | 1. Yes  2. No  9. Don’t know | | 🞎***2 or 9 → SQ5b.9*** | | |
|  | .9 | How many times was a Hep B vaccination received?  Sau nawa aka yi allurar Hep B? | | | | **__ __** Times  *(DK = 99)* | | |
| S5b.9 | | Were any of the vaccinations <NAME> received given as part of a national immunization day campaign?  Shin akwai allurar rigakafi da <NAME> ya/ta karba a ranar gangamin kaddamar da rigakafi na kasa ? | | 1. Yes 2. No   9. Don’t know | | 🞎***2 or 9 → SQ5b.10*** | | |
| S5b.9.1 | | At which national immunization day campaigns did <NAME> receive vaccinations?  Shin a *wane* ranar gangamin kaddamar da rigakafi na kasa ne <NAME> ya/ta karbi alluran?  *[Record all campaigns mentioned.]* | | 1. <CAMPAIGN 1> (TYPE/DATE) 2. <CAMPAIGN 1> (TYPE/DATE) 3. <CAMPAIGN 1> (TYPE/DATE) 4. <CAMPAIGN 1> (TYPE/DATE) | | 1. □  2. □  3. □  4. □ | | |
| S5b.10 | | (Before / In the six months before) the fatal illness, did <NAME> receive one or more vitamin A doses like this?  *Shin (Kafin /A cikin watanni 6 da suka shige kafin) farawar mummunan rashin lafiyar, ko* <NAME> ya/ta karbi sanadarin vitamin A kariyar ido kamar haka?  *[Read “Before…” if the child lived less than 6 months.]*  *[Show ampoule/capsule/syrup]* | | 1. Yes, 1 dose 2. Yes, 2 or more doses 3. No   9. Don’t know | | 🞎 | | |
| S5b.11 | | Before the fatal illness began, did <NAME> suffer from any of the following known conditions:  Kafin farawar mummunar rashin lafiyar, ko <NAME> ya/ta yi fama da daya daga cikin wadannan lallurorin?  *[Read out all conditions and check “Yes,” “No” or “Don’t know” for each.]*  *If “Yes,” then ask:* Was s/he provided any treatment for this condition? | | 1. Low height or weight (malnutrition) 2. Malformation (from the time of birth):    1. Head, neck and/or back    2. Mouth/palate    3. Heart    4. Arms and/or legs 3. Asthma 4. Heart disease 5. Tuberculosis 6. Epilepsy/convulsion 8. Other   *(specify other)* | | Suffered from  Yes No DK  1. □ 2. □ 9. □    1. □ 2. □ 9. □  1. □ 2. □ 9. □  1. □ 2. □ 9. □  1. □ 2. □ 9. □  1. □ 2. □ 9. □  1. □ 2. □ 9. □  1. □ 2. □ 9. □  1. □ 2. □ 9. □  1. □ 2. □ 9. □ | Treatment  Yes No DK  1. □ 2. □ 9. □    1. □ 2. □ 9. □  1. □ 2. □ 9. □  1. □ 2. □ 9. □  1. □ 2. □ 9. □  1. □ 2. □ 9. □  1. □ 2. □ 9. □  1. □ 2. □ 9. □  1. □ 2. □ 9. □  1. □ 2. □ 9. □ | |
|  |  |  |  |  |  | ___________________________ | | |
| **VA Section 4: Infant and child deaths (FOR CHILD DEATHS 28 DAYS—59 MONTHS OLD)**  *Read:* Now I’d like to ask you about <NAME>’s illness.  Yanzu ina son in tambayeki gameda rashin lafiyar <NAME> | | | | | | | | |
| V4.1 | | During the illness that led to death, did the <NAME> have a fever?  *Lokacin rashin lafiyar da tayi sanadiyyar rasuwar,* <NAME> ya/ta na zazzabi? | | 1. Yes 2. No   9. Don’t know | | 🞎 ***2 or 9 → VQ4.6*** | | |
| V4.2 | | How many days did the fever last?  *Kwana nawa ya/ta yi da zazzabin?*  *[Less than 24 hours = “00” days]* | | | | **__ __** Days  *(DK = 99)* | | |
| V4.3 | | Did the fever continue until death?  Zazzabin ya ci gaba har lokacin rasuwa? | | 1. Yes 2. No   9. Don’t know | | 🞎 ***2 or 9 → VQ4.6*** | | |
| V4.4 | | How severe was the fever?  Yaya tsananin zazzabin? | | 1. Mild 2. Moderate 3. Severe   9. Don’t know | | 🞎 | | |
| V4.5 | | What was the pattern of the fever?  Yaya yanayin zazzabin? | | 1. Continuous 2. On and off 3. Only at night   9. Don’t know | | 🞎 | | |
| V4.6 | | During the illness that led to death, did <NAME> have more frequent loose or liquid stools than usual?  *Lokacin rashin lafiyar da tayi sanadiyyar rasuwar,* <NAME> *ya/ta na gudawa akai-akai ko zawo mai ruwa-ruwa da ba a saba ba?* | | 1. Yes 2. No   9. Don’t know | | 🞎 ***2 or 9 → VQ4.12*** | | |
| V4.7 | | How many stools did <NAME> have on the day that loose liquid stools were most frequent?  Sau nawa <NAME> yayi gudawa kamar zawo a wannan ranar da gudawar yayi tsanani? | | | | **__ __** Stools  *(DK = 99)* | | |
| V4.8 | | How many days before death did the frequent loose or liquid stools start?  *Kwana nawa kafin rasuwar sa/ta, ya/ta fara gudawa ko zawo mai ruwa-ruwa?*  *[Less than 24 hours = “00” days]* | | | | **__ __** Days  *(DK = 99)* | | |
| V4.9 | | Did the frequent loose or liquid stools continue until death?  Shin gudawar akai-akai taci gaba har lokacin rasuwa? | | 1. Yes 2. No   9. Don’t know | | 🞎 ***1 or 9 → VQ4.11*** | | |
| V4.10 | | How many days before death did the loose or liquid stools stop?  *Kwana nawa kafin rasuwar sa/ta gudawar ta tsaya?*  *[Less than 24 hours = “00” days]* | | | | **__ __** Days  *(DK = 99)* | | |
| V4.11 | | Was there visible blood in the loose or liquid stools?  Akwai alamun jini a cikin kashin ko gudawar? | | 1. Yes 2. No   9. Don’t know | | 🞎 | | |
| V4.12 | | During the illness that led to death, did the child have a cough?  Lokacin rashin lafiyar da ya yi sanadiyyar rasuwar, jaririn ya/ta na tari? | | 1. Yes 2. No   9. Don’t know | | 🞎 ***2 or 9 → VQ4.16*** | | |
| V4.13 | | For how many days did the cough last?  *Har Kwana nawa ya/ta ke tarin?*  *[Less than 24 hours = “00” days]* | | | | **__ __** Days  *(DK = 99)* | | |
| V4.14 | | Was the cough very severe?  Tarin ya yi tsanani sosai? | | 1. Yes 2. No   9. Don’t know | | 🞎 | | |
| V4.15 | | Did the child vomit after s/he coughed?  Shin ko jaririn ya/ta kan yi amai bayan tarin? | | 1. Yes 2. No   9. Don’t know | | 🞎 | | |
| V4.16 | | During the illness that led to death, did <NAME> have difficult breathing?  Lokacin rashin lafiyar da yayi sanadiyyar rasuwar, ko <NAME> ya/ ta na da matsalar yin nunfashi? | | 1. Yes 2. No   9. Don’t know | | 🞎 ***2 or 9 → VQ4.18*** | | |
| V4.17 | | For how many days did the difficult breathing last?  *Har kwana nawa ne aka yi da matsalar yin nunfashi?*  *[Less than 24 hours = “00” days]* | | | | **__ __** Days  *(DK = 99)* | | |
| V4.18 | | During the illness that led to death, did <NAME> have fast breathing?  Lokacin rashin lafiyar da yayi sanadiyyar rasuwar, ko <NAME> ya/ ta na yin nunfashi akai-akai? | | 1. Yes 2. No   9. Don’t know | | 🞎 ***2 or 9 → VQ4.20*** | | |
| ***Inst_1: If both VQ4.16 and VQ4.18 = 2 or 9 → VQ4.25*** | | | | | | | | |
| V4.19 | | For how many days did the fast breathing last?  *[Less than 24 hours = “00” days]*  *Har kwana nawa ya/ta yi nunfashin akai-akai?* | | | | **__ __** Days  *(DK = 99)* | | |
| V4.20 | | During the illness that led to death, did s/he have indrawing of the chest?  Lokacin rashin lafiyar da yayi sanadiyyar rasuwar, ko kan kirjin sa/ta ya fada ? | 1. Yes 2. No   9. Don’t know | | | 🞎 | | |
| V4.21 | | During the illness that led to death, did her/his breathing sound like any of the following?  Lokacin rashin lafiyar da yayi sanadiyyar rasuwar, ko nunfashin sa/ta na kara daya daga cikin wadanna ?  *[Demonstrate each sound/show video]* |  | | |  | | |
| V4.22 | | Stridor | 1. Yes 2. No   9. Don’t know | | | 🞎 | | |
| V4.23 | | Grunting  Kakari yin numfashi cikin wahala | 1. Yes 2. No   9. Don’t know | | | 🞎 | | |
| V4.24 | | Wheezing  Numfashi ta kirji ? | 1. Yes 2. No   9. Don’t know | | | 🞎 | | |
| V4.25 | | Did <NAME> experience any generalized convulsions or fits during the illness that led to death?  Shin ko <NAME> ya/ta sami matsalar tsika-tsinka ko rashin kuzari lokacin rashin lafiyar da ya/ta yi sanadiyyar rasuwar? | 1. Yes 2. No   9. Don’t know | | | 🞎 | | |
| V4.26 | | Was <NAME> unconscious during the illness that led to death?  Ko <NAME> ya/ta suma lokacin rashin lafiyar da ya/ta yi sanadiyyar rasuwar? | 1. Yes 2. No   9. Don’t know | | | 🞎 ***2 or 9 → VQ4.28*** | | |
| V4.27 | | How long before death did unconsciousness start?  Har tsawon wanne lokaci ne kafin rasuwar ne suman ya fara? | 1. Less than 6 hours 2. 6-23 hours 3. 24 hours or more   9. Don’t know | | | 🞎 | | |
| V4.28 | | Did <NAME> have a stiff neck during the illness that led to death?  Shin ko wuyan <NAME> ya sankare lokacin rashin lafiyar da tayi sanadiyar rasuwar?  *[Demonstrate/show video]* | 1. Yes 2. No   9. Don’t know | | | 🞎 | | |
| V4.29 | | Did <NAME> have a bulging fontanelle during the illness that led to death?  A yayin rashin lafiyar da ta yi sana diyar rasuwar <NAME> shin madigan ya kumbura?  *[Show photo]* | 1. Yes 2. No   9. Don’t know | | | 🞎 | | |
| V4.30 | | During the month before s/he died, did <NAME> have a skin rash?  A cikin watan da <NAME> ya/ta rasu, ko akwai kuraje a jikin? | 1. Yes 2. No   9. Don’t know | | | 🞎 ***2 or 9 → VQ4.35*** | | |
| V4.31 | | Where was the rash?  A wanne wuri kurajen suke a jikin? | 1. Face 2. Trunk/Abdomen 3. Extremities 4. Everywhere   9. Don’t know | | | 🞎 | | |
| V4.32 | | Where did the rash start?  A wanne wuri kurajen suka fara fitowa? | 1. Face 2. Trunk/Abdomen 3. Extremities 4. Everywhere   9. Don’t know | | | 🞎 | | |
| V4.33 | | How many days did the rash last?  Har kwanaki nawa kurajen su ka yi? | | | | **__ __** Days  *(DK = 99)* | | |
| V4.34 | | Did the rash have blisters containing clear fluid?  *Shin ko kurajen sunyi bororo kuma su kumshi ruwa?* | | 1. Yes 2. No   9. Don’t know | | 🞎 | | |
| V4.35 | | During the illness that led to death, did <NAME>’s limbs (legs, arms) become very thin?  *Lokacin rashin lafiyar da tayi sanadiyar rasuwar, shin ko kafafu da hannuwan <*NAME> *sun rame sosai?*  *[Show photo]* | | 1. Yes 2. No   9. Don’t know | | 🞎 | | |
| V4.36 | | During the illness that led to death, did <NAME> have swollen legs or feet?  *Lokacin rashin lafiyar da tayi sanadiyar rasuwar, shin ko kafafu da hannuwan <*NAME> *sun kumbura?* | | 1. Yes 2. No   9. Don’t know | | 🞎 ***2 or 9 → VQ4.38*** | | |
| V4.37 | | How long did the swelling last?  Har kwanaki nawa kumburin kafan ya yi?  *[Record days or weeks.]* | | | | **__ __** Days  *(DK = 99)* | | |
|  |  |  |  |  |  | **__ __** Weeks  *(DK = 99)* | | |
| V4.38 | | During the illness that led to death, did <NAME>’s skin flake off in patches?  Lokacin rashin lafiyar da tayi sanadiyar rasuwar, shin ko fatar jikin <NAME> ya yi tsaba yana zuba? | | 1. Yes 2. No   9. Don’t know | | 🞎 | | |
| V4.39 | | Did <NAME>’s hair change in color to a reddish or yellowish color?  Shin ko gashin <NAME> ya canja launi zuwa ja ko ruwan dorawa? | | 1. Yes 2. No   9. Don’t know | | 🞎 | | |
| V4.40 | | Did <NAME> have a protruding belly?  Shin ko cikin <NAME> ya kumbura? | | 1. Yes 2. No   9. Don’t know | | 🞎 | | |
| V4.41 | | During the illness that led to death, did <NAME> suffer from “lack of blood” or “pallor”?  Lokacin rashin lafiyar da tayi sanadiyar rasuwar, shin ko <NAME> ya/ta yi fama da karancin jini? | | 1. Yes 2. No   9. Don’t know | | 🞎 | | |
| V4.42 | | During the illness that led to death, did <NAME> have swelling in the armpits?  Lokacin rashin lafiyar da tayi sanadiyar rasuwar, shin ko <NAME> ya/ta yi fama da kumburin kasan hammata? | | 1. Yes 2. No   9. Don’t know | | 🞎 | | |
| V4.43 | | During the illness that led to death, did <NAME> have a whitish rash inside the mouth or on the tongue?  Lokacin rashin lafiyar da tayi sanadiyar rasuwar, shin <NAME> ya/ta na da kuraje fari a cikin bakin ko kan harshe? | | 1. Yes 2. No   9. Don’t know | | 🞎 | | |
| V4.44 | | During the illness that led to death, did <NAME> bleed from anywhere?  Lokacin rashin lafiyar da tayi sanadiyar rasuwar, shin ko <NAME> ya zubar da jini daga wani wuri a jiki? | | 1. Yes 2. No   9. Don’t know | | 🞎 ***2 or 9 → VQ4.46*** | | |
| V4.45 | | *Record from where s/he bled:* | |  | | | | |
| V4.46 | | During the illness that led to death, did s/he have areas of the skin that turned black?  Lokacin rashin lafiyar da tayi sanadiyar rasuwar, ko akwai wani wuri daga jikin sa/ta da ya canja launi zuwa baki? | | 1. Yes 2. No   8. Don’t know | | 🞎 | | |
| V4.47 | | Did <NAME> suffer from an injury or accident such as…?  *Shin ko* <NAME>ya/ta ji rauni ko wani hatsari kamar?  *[Ask the respondent each in sequence and mark each as “Yes,” “No” or “Don’t know.”]* | | 1. a road traffic crash/injury? 2. a fall? 3. drowning? 4. poisoning?   Did s/he suffer:   1. a bite or sting by a venomous animal? 2. a burn? 3. from violence (homicide, abuse)? 4. any other injury?   *(If “Yes,” then specify)* | | Yes No DK  1. □ 2. □ 9. □  1. □ 2. □ 9. □  1. □ 2. □ 9. □  1. □ 2. □ 9. □  1. □ 2. □ 9. □  1. □ 2. □ 9. □  1. □ 2. □ 9. □  1. □ 2. □ 9. □ | | ***All = 2 or 9***  ***→ SQ6.1*** |
|  |  |  |  |  |  | ___________________________ | | |
| V4.48 | | Was the injury or accident intentionally inflicted by someone else?  Shin raunin ko hatsarin wani ne yayi shi? | | 1. Yes 2. No   8. Don’t know | | 🞎 | | |
| V4.49 | | How long did <NAME> survive after the injury or accident?  *Tsawon wanne lokaci ne* <NAME> ya rayu bayan jin raunin ko hatsarin?  *[Record hours if less than 24 hours—Less than 1 hour = “00” hours;*  *Record days if 1 day or more.]* | | | | **__ __** Hours  *(DK = 99)* | | |
|  |  |  |  |  |  | **__ __** Days  *(DK = 99)* | | |

| **SA Module 6: Care-seeking for the child’s fatal illness (FOR NN & CHILD DEATHS 0—59 MONTHS OLD)**  ***Read:*** Now, I’d like to ask you about <NAME>’s fatal illness and the care and treatments that s/he received.  Yanzu ina son in tambayeki gameda mummunar rashin lafiyar da <NAME> ya/ta yi da samun kulawa da magunguna da aka samu. | | | | | | | | | | | | | | | | | | | |
| --- | --- | --- | --- | --- | --- | --- | --- | --- | --- | --- | --- | --- | --- | --- | --- | --- | --- | --- | --- |
| S6.1 | Who first noticed that <NAME> was ill?  Wanene ya fara lura cewa <NAME> ba lafiya? | | | | | | | | 1. The respondent 2. Other relative, neighbor, friend 3. CHW or nurse at home or in community 4. Doctor or nurse at a health facility 5. Other *(specify)* | | | | | | | 🞎  ___________________________ | | | |
| S6.2 | Earlier you said that <NAME> had <SYMPTOM(S)> during her/his illness.  *[Read back all the child’s symptoms from VA section 3 (for neonates) or VA section 4 (for children).]*  *Dazu kin fada mun cewa* <NAME> ya/ta na da <SYMPTOM(S)> yayin rashin lafiyar sa/ta.  How did <SQ6.1 PERSON> first know that <NAME> was ill? Which of these symptoms did s/he have at that time?  Ta ya ya <SQ6.1 PERSON> ta/ta fara sanin cewa <NAME> na rashin lafiya? Wadanne alamomin nan ne ya/ta samu a lokacin?  What symptoms did s/he have next? On what day of the illness did these symptoms start?  Wadanne alamomin ne ya/ta samu daga baya kuma?  A wanne ranar rashin lafiyar ne wadannan alamomin suka fara?  *[Probe until all the symptoms are recorded in the order they appeared.]* | | | | | | | | **Symptoms in order of appearance** | | | | | | | **Illness day the symptom started** | | | |
|  |  |  |  |  |  |  |  |  | 1. | | | | | | |  | | | |
|  |  |  |  |  |  |  |  |  | 2. | | | | | | |  | | | |
|  |  |  |  |  |  |  |  |  | 3. | | | | | | |  | | | |
|  |  |  |  |  |  |  |  |  | 4. | | | | | | |  | | | |
|  |  |  |  |  |  |  |  |  | 5. | | | | | | |  | | | |
|  |  |  |  |  |  |  |  |  | 6. | | | | | | |  | | | |
|  |  |  |  |  |  |  |  |  | 7. | | | | | | |  | | | |
| S6.3 | When <SQ6.1 PERSON> first noticed that <NAME> was ill, was s/he…  Lokacin da <SQ6.1 PERSON> ya/ta fara lura cewa <NAME> ba shi/ta da lafiyar, ya/ta na  *[Read the choices for each condition.]* | | | | | | | | 1. Feeding normally, poorly, or not at all   Ci dadai da kullum, ci kadan,ko babu ci sam   1. Alert, drowsy, or unconscious kuzari,babu kuzari ko na nan a sume 2. Normally active, less active than normal, or not moving da karfi daidai da kullum, kasa da kullum ko babu motsi | | | | | | | Normal Medium Abnormal DK  1. □ 2. □ 3. □ 9. □  1. □ 2. □ 3. □ 9. □  1. □ 2. □ 3. □ 9. □ | | | |
| S6.4 | Did <NAME> receive, or did you seek or try to seek, any care or treatment for the fatal illness?  Shin ko <NAME> ya/ta samu, ko kin nemi ko kin yi kokarin samun wata kulawa ko magunguna domin wannan mummunar rashin lafiyar? | | | | | | | | 1. Yes 2. No—care not needed, given or sought ba a buka ci kular ba, ba a bada kular ba ko ba a nemi kular ba. 3. No— died immediately. Ya/ ta rasu nan da nan.   9. Don’t know ban sani ba. | | | | | | | 🞎***2 → SQ6.6***  ***3 or 9 → VQ5.10*** | | | |
| S6.5 | Please tell me everything you did for <NAME>’s fatal illness inside the home and all the places outside the home you took or tried to take (her / him) for health care. Start with the first care or treatment <NAME> received and then, in order, tell me all the other care and treatments s/he received.  Now, what was the first thing you did or tried to do for <NAME>’s illness?  Yanzu, menene ki/ ta yin a farko ko aka yi kokarin yi wa rashin lafiyar <NAME>’  *For neonatal deaths only:* Did the illness begin at the health provider where the child was delivered?  Shin rashin lafiyar ya fara a wajen kiwon Lafiyar da aka haifi jaririn?  On what day of the illness was the action taken?  A wanne rana rashin lafiyar ne aka dauki matakin?  For what symptoms was the action taken?  Wanne lallurar ne aka daukan ma mataki.  Da Allah fada mani duk abubuwan da kukayi ma <NAME> a gida da wasu wuraren domin kulawa da neman lafiyar sa/ta. Ki fara da kulawar farko da magungunan da ya/ta amsa. Kuma da Allah a shaida mani a wanne lokaci kuma wanne mataki aka daukar ma ko wacce matsalar.  *[Include any provider <NAME> did not reach because s/he died before leaving home or on route.]*  *Hada da sunan jami’ai masu kulawa wadanda ba a samu zuwa wajensu ba domin ya/ta rasu kafin abar gida ko kuma kan hanyar zuwa.*  *(1) Check one other care or health provider box for each action row. (2) For neonatal deaths only: If the illness began at the health provider where the child was delivered, then mark that as Action 1 and check the “illness began at provider” box. (3) Record the illness day each action was taken. (4) Ensure no action was taken for a symptom before it started (in SQ6.2).* | | | | | | | | | | | | | | | | | | |
| **Action #** | **(1)**  **Other care** | | | **(1)**  **Health Providers** | | | | | | | | | | **(3)**  **Illness day the action was taken** | | | **(4)**  **For what symptom(s) was the action taken?** | | |
|  | **Home care (own, relative, neigh-bor, friend)** | **Tradi-tional or non-formal provider** | **Phar-macist or drug seller** | **Trained CH**  **Worker, nurse, or midwife** | | **Private doctor**  **(formal/unsure)** | | | | **NGO or govt. clinic** | **Hospital** | **(2)**  **Illness began at provider where child was delivered** | |  |  |  |  |  |  |
| 1. | 🞎 | 🞎 | 🞎 | 🞎 | | 🞎 | | | | 🞎 | 🞎 | 🞎 | | **__ __**  *(DK = 99)* | | |  | | |
| 2. | 🞎 | 🞎 | 🞎 | 🞎 | | 🞎 | | | | 🞎 | 🞎 |  | | **__ __**  *(DK = 99)* | | |  | | |
| 3. | 🞎 | 🞎 | 🞎 | 🞎 | | 🞎 | | | | 🞎 | 🞎 |  | | **__ __**  *(DK = 99)* | | |  | | |
| 4. | 🞎 | 🞎 | 🞎 | 🞎 | | 🞎 | | | | 🞎 | 🞎 |  | | **__ __**  *(DK = 99)* | | |  | | |
| 5. | 🞎 | 🞎 | 🞎 | 🞎 | | 🞎 | | | | 🞎 | 🞎 |  | | **__ __**  *(DK = 99)* | | |  | | |
| 6. | 🞎 | 🞎 | 🞎 | 🞎 | | 🞎 | | | | 🞎 | 🞎 |  | | **__ __**  *(DK = 99)* | | |  | | |
| 7. | 🞎 | 🞎 | 🞎 | 🞎 | | 🞎 | | | | 🞎 | 🞎 |  | | **__ __**  *(DK = 99)* | | |  | | |
| ***Inst_1: (For neonatal deaths only) If illness began at health provider where child was delivered:***  ***And did not fill L&D matrix (module 4) → SQ6.10; And filled L&D matrix (module 4) → SQ6.16*** | | | | | | | | | | | | | | | | | | | |
| S6.6 | *If no care given or sought, ask:* Who decided that <NAME> did not need any care or treatment for the illness?  *Wanene ya yanke shawarar cewa* <NAME> ba ya/ta bukatar kulawa ko magungunan rashin lafiyar sa/ta?  *If any care given or sought, ask:* Who decided that <ACTION 1> was the first thing to do for <NAME>’s illness?  Wanene ya yanke shawarar cewa <ACTION 1> shi ne abun da za ayi na farko don rashin lafiyar <NAME>  *[Record the one main decision maker.]* | | | | | | | | 1. Child’s mother 2. Child’s father 3. Child’s aunt 4. Child’s uncle 5. Child’s grandmother 6. Child’s paternal grandfather 7. Child’s maternal grandfather 8. Other *(specify)*   9.Don’t know | | | | | | | 1. □  2. □  3. □  4. □  5. □  6. □  7. □  8. □ _______________________  9. □ | | | |
| S6.7 | *If never taken to a health provider, ask:* Did you have any concerns or problems that kept you from taking <NAME> to a health provider during his/her illness?  Ki na da wata damuwa ko matsala da ta hana ki kai <NAME> wurin jami’an kiwon lafiya lokacin rashin lafiyar sa/ta?  *If taken to a health provider, ask:* Did you have to overcome any concerns or problems to take <NAME> to the (first) health provider?  Sai da ki ka ciwo kan matsalolin da damuwar kafin ki ka kai <NAME> wurin jami’an kiwon lafiya na farko? | | | | | | | | 1. Yes 2. No   9. Don’t know | | | | | | | 🞎 ***2 or 9 → Inst_2*** | | | |
| S6.7.1 | What concerns or problems did you have?  *Wadanne zarafi ko matsaloli ki ka samu?*  *Prompt:* Was there anything else?  *Da akwai wani abu kuma?*  *[Multiple answers allowed.]* | | | | | | | | 1. Did not think child was sick enough to need health care 2. No one available to go with caregiver 3. Too much time from her regular duties 4. Someone else *(specify)* had to decide 5. Too far to travel 6. No transportation available 7. Cost (transport, health care, other) 8. Not satisfied with available health care 9. Problem required traditional care 10. Thought child was too sick to travel 11. Thought child will die no matter what 12. Was late at night (transportation or provider not available) 13. Other *(specify)*   99.Don’t know | | | | | | | 1. □  2. □  3. □  4. □ _______________________  5. □  6. □  7. □  8. □  9. □  10. □  11. □  12. □  13. □ ______________________  99. □ | | | |
| ***Inst_2: If SQ6.4 = 2 (No care given) or***  ***If SQ6.5 ≠ “Health Provider” (Never took and never tried to take to a health provider) → SQ6.39*** | | | | | | | | | | | | | | | | | | | |
| S6.8 | *Refer to SQ6.5 for the first health provider and related symptoms:*  You mentioned that you took <NAME> to the (first) health provider, I mean the <FIRST HEALTH PROVIDER> for <SYMPTOM(S)>. How long had <NAME> had (this / these) symptom(s) when it was decided to take him/her to the <FIRST HEALTH PROVIDER>? Tsawon wanne lokaci ne <NAME>ya/ta ke da (wannan /wadannan) matsalar /matsololin kafin aka yi shawarar kai shi/ta wajen <FIRST HEALTH PROVIDER>?  *[Read “…to the first…” if took or tried to take to more than one health provider.]*  *[Mark days, hours &/or minutes as needed: e.g. 00 day, 02 hours, 10 minutes]* | | | | | | | | | | | | | | | **__ __** Days  *(DK = 99)* | | | |
|  |  |  |  |  |  |  |  |  |  |  |  |  |  |  |  | **__ __** Hours  *(DK = 99)* | | | |
|  |  |  |  |  |  |  |  |  |  |  |  |  |  |  |  | **__ __** Minutes  *(DK = 99)* | | | |
| ***Child illness matrix instructions:*** *Ask the following questions for the first and last health providers where care was sought or tried to be sought for the fatal illness. Ask all the questions for the first provider before going on to the last.*  *Before asking about the first health provider, read:*  Now I would like to ask you about your visit to the (first) health provider. *[Read “first” if went or tried to go to more than one provider.]*  *Before asking about the last health provider, read:*  Now I would like to ask you about your visit to the last health provider, I mean the <LAST HEALTH PROVIDER>. Yanzu ina son na tambaye ki game da zuwan ki wajen jami’in kiwon lafiya na karshe, ina nufin <LAST HEALTH PROVIDER> | | | | | | | | | | | | | | | | | | | |
| **– CHILD ILLNESS MATRIX QUESTIONS –** | | | | | | | | | | | | | **FIRST HEALTH PROVIDER** | | | | | **LAST HEALTH PROVIDER** | |
| At the time when it was decided to take <NAME> to the <FIRST/LAST HEALTH PROVIDER>, was s/he…  *[Read the choices for each condition.]*  *A lokacin da aka yanke shawarar kai*<NAME>  *wajen <*FIRST/LAST HEALTH PROVIDER>, ko ya/ta yi?  *Read the choices for each condition.]* | | | | | 1Feeding normally, poorly, or not at all  Ci dadai da kullum, ci kadan,ko babu ci sam  2 Alert, drowsy, or unconscious kuzari,babu kuzari ko na nan a sume  3 Normally active, less active than normal, or not moving da karfi daidai da kullum, kasa da kullum ko babu motsi | | | | | | | | S6.9  Nrml Med Abnrm DK  1. □ 2. □ 3. □ 9. □  1. □ 2. □ 3. □ 9. □  1. □ 2. □ 3. □ 9. □ | | | | | S6.24  Nrml Med Abnrm DK  1. □ 2. □ 3. □ 9. □  1. □ 2. □ 3. □ 9. □  1. □ 2. □ 3. □ 9. □ | |
| What was the name of the <FIRST/LAST HEALTH PROVIDER> where you took <NAME>?  *Probe to identify the type of provider.*  *Menene sunan* <FIRST/LAST HEALTH PROVIDER> *da ku ka kai* <NAME>?  *Probe to identify the type of provider.* | | | | | 1. Hospital (Government) 2. Hospital (NGO) 3. Hospital (Private) 4. Health center (Government) 5. Health center (NGO) 6. Health post (Government) 7. Health post (NGO) 8. Private doctor/clinic (Formal) 9. Private doctor/clinic (?Formal?) 10. Trained community health worker, nurse, or midwife   99. Don’t know | | | | | | | | S6.10  🞎🞎  ___________________  (Name of Provider or Facility) | | | | | S6.25  🞎🞎  ___________________  (Name of Provider or Facility) | |
| After (deciding to seek care / being referred), how much time passed before going to the <FIRST/LAST HEALTH PROVIDER>?  Bayan yanke shawarar cewa a nemi kula / tura wa wajen cibiyar kiwon lafiya, tsawon wanne lokaci aka dauka kafin zuwa <FIRST/LAST HEALTH PROVIDER>?  *[Discuss that this might include the time needed to arrange for transportation and money to go to the provider/facility, or to provide home care or go to a traditional provider before going to the health provider.]*  *[If the child died at home, record the time from decision/referral to death.]*  *[Mark days, hours &/or minutes as needed: e.g. 01 day, 05 hours, 30 minutes]* | | | | | | | | | | | | | S6.11  **__ __** Days  *(DK = 99)* | | | | | S6.26  **__ __** Days  *(DK = 99)* | |
|  |  |  |  |  |  |  |  |  |  |  |  |  | **__ __** Hours  *(DK = 99)* | | | | | **__ __** Hours  *(DK = 99)* | |
|  |  |  |  |  |  |  |  |  |  |  |  |  | **__ __** Minutes  *(DK = 99)* | | | | | **__ __** Minutes  *(DK = 99)* | |
| Was there any cost to travel to the <FIRST/LAST HEALTH PROVIDER> or pay for the child’s care there?  Akwai wasu kudin sufuri da za’a kashe wajen zuwa <FIRST/LAST HEALTH PROVIDER> ko wanda za’a biya domin samun kulawar yaron a wajen? | | | | | 1. Yes 2. No   9. Don’t know | | | | | | | | S6.12  🞎 ***2 or 9 → SQ6.13*** | | | | | S6.27  🞎 ***2 or 9 → SQ6.28*** | |
| How did you arrange for the money for these expenses?  *Tayaya ki ka samu wadannan kudaden da ki ka kashe?*  *[Multiple answers allowed.]* | | | | | 1. Had available 2. Borrowed 3. Sold assets 4. Help from kin/relatives 5. Community fund 6. Govt. scheme 7. Other   9. Don’t know | | | | | | | | S6.12.1  1. □  2. □  3. □  4. □  5. □  6. □  7. □  9. □ | | | | | S6.27.1  1. □  2. □  3. □  4. □  5. □  6. □  7. □  9. □ | |
| What transportation method was used to go there?  *[Multiple answers allowed.]*  *Wacce irin hanyar sufuri ki ka yi amfani da ita wajen zuwa?* | | | | | 1. Walk 2. Bicycle/rickshaw/cart/ boat 3. Bus 4. Taxi/auto/trecker 5. Ambulance (auto or motorcycle) 6. Other 7. Could not arrange transport   9. Don’t know | | | | | | | | S6.13  1. □ ***If only walk***  2. □ ***→ SQ6.14.1***  3. □  4. □  5. □  6. □  7. □ ***→ SQ6.14.1***  9. □ | | | | | S6.28  1. □ ***If only walk***  2. □ ***→ SQ6.29.1***  3. □  4. □  5. □  6. □  7. □ ***→ SQ6.29.1***  9. □ | |
| How much did the transportation cost?  Nawa ne kudin sufurin? | | | | | | | | | | | | | S6.14  **__ __ __ __** unit  *(DK=9999)* | | | | | S6.29  **__ __ __ __** unit  *(DK = 9999)* | |
| Did the child reach the <FIRST/LAST HEALTH PROVIDER> before s/he died?  *[If “No,” discuss with respondent to determine correct response: 2, 3 or 4.]*  *An samu kai yaron/yarinyar wajen* <FIRST/LAST HEALTH PROVIDER> kafin ya/ta rasu?  *[If “No,” discuss with respondent to determine correct response: 2, 3 or 4.]* | | | | | 1. Yes, reached before child died 2. No, died before setting out 3. No, died on route to this provider 4. No, could not reach this provider – did not set out/returned home/took other action   9. Don’t know | | | | | | | | S6.14.1  🞎 ***2, 3 → SQ6.39***  ***4, 9 → Inst_4*** | | | | | S6.29.1  🞎 ***2-9 → SQ6.39*** | |
| How long did it take to travel to the <FIRST/LAST HEALTH PROVIDER>?  *Tsawon wanne lokaci a ka dauka zuwa wajen* <FIRST/LAST HEALTH PROVIDER>?  *[Mark hours &/or minutes as needed: e.g. 02 hours, 10 minutes]* | | | | | | | | | | | | | S6.15  **__ __** Hours  *(DK = 99)* | | | | | S6.30  **__ __** Hours  *(DK = 99)* | |
|  |  |  |  |  |  |  |  |  |  |  |  |  | **__ __** Minutes  *(DK = 99)* | | | | | **__ __** Minutes  *(DK = 99)* | |
| What did the <FIRST/LAST HEALTH PROVIDER> do for <NAME>’s problem?  *Menene abin da* FIRST/LAST HEALTH PROVIDER> yayi akan matsalar <NAME>?  *Prompt:* Was there anything else?  Da akwai wani abu kuma?  *[Multiple answers allowed.]* | | | | | 1. Gave oxygen 2. Helped breathe with bag or mask 3. Gave fluids by mouth 4. Gave antibiotics by mouth 5. Gave antimalarial by mouth 6. Gave ORS 7. Gave Vitamin A 8. Gave other medicine by mouth 9. Gave IM medicine 10. Gave IV fluids or medicine 11. Advised to buy outside medicine 12. Did an operation *(specify)* 13. Admitted to hospital 14. Other *(specify)* 15. Nothing   99.Don’t know | | | | | | | | S6.16  1. □  2. □  3. □  4. □  5. □  6. □  7. □  8. □ _______________  9. □  10. □  11. □  12. □ ______________  13. □ stayed __ __ days  14. □ ______________  15. □ ***→ SQ6.18***  99. □ ***→ SQ6.18*** | | | | | S6.31  1. □  2. □  3. □  4. □  5. □  6. □  7. □  8. □ _______________  9. □  10. □  11. □  12. □ ______________  13. □ stayed __ __ days  14. □ ______________  15. □ ***→ SQ6.33***  99. □ ***→ SQ6.33*** | |
| How much did you pay for these treatments and other costs related to the health care, including the admission fee, consultation, lab tests, equipment, and room and food for companions?  Nawa ne kudin da ki ka biya domin magunguna da kulawa da suka hada, da kati, gwaji, kudin gado, daki da abincin da ‘yan uwa suka ci? | | | | | | | | | | | | | S6.17  **__ __ __ __ __** unit  *(DK = 99999)* | | | | | S6.32  **__ __ __ __ __** unit  *(DK = 99999)* | |
| Did the <FIRST/LAST HEALTH PROVIDER> refer <NAME> to another health provider or facility?  Shin <FIRST/LAST HEALTH PROVIDER> suntura<NAME> wajen wani jami’an kiwon lafiya ko cibiyar kiwon lafiya? | | | | | 1. Yes 2. No   9. Don’t know | | | | | | | | S6.18  🞎 ***2 or 9 → SQ6.19*** | | | | | S6.33  🞎 ***2 or 9 → SQ6.34*** | |
| Why was <NAME> referred?  Menene ya sa aka tura <NAME>  *[Multiple answers allowed.]*  *Menene dalilin da suka tura* <NAME> *wannan wurin?* | | | | | 1. The provider was not capable of managing the problem 2. Required supplies (e.g., drugs, IV, oxygen) not available 3. Required equipment (e.g., xray machine) not available   9. Don’t know | | | | | | | | S6.18.1  1. □  2. □  3. □  9. □ | | | | | S6.33.1  1. □  2. □  3. □  9. □ | |
| Did <NAME> leave the <FIRST/LAST HEALTH PROVIDER> alive?  Shin <NAME> ya bar wajen <FIRST/LAST HEALTH PROVIDER> da rai? | | | | | 1. Yes, left alive 2. No, died at this provider | | | | | | | | S6.19  🞎 ***2 → VQ5.4*** | | | | | S6.34  🞎 ***2 → VQ5.4*** | |
| Did the <FIRST/LAST HEALTH PROVIDER> suggest that you do anything for <NAME>’s illness after leaving?  Shin <FIRST/LAST HEALTH PROVIDER> sun ba ki shawarar ki yi wani abu gameda rashin lafiyar <NAME> bayan kun bar wurin? | | | | | 1. Yes 2. No   9. Don’t know | | | | | | | | S6.20  🞎 ***2 or 9 → SQ6.22*** | | | | | S6.35  🞎 ***2 or 9 → SQ6.37*** | |
| What did the <FIRST/LAST HEALTH PROVIDER> suggest that you do?  *Wacce shawara* <FIRST/LAST HEALTH PROVIDER> suka ba ki?  *Prompt:* Was there anything else?  Da akwai wani abu kuma?  *[Multiple answers allowed.]* | | | | | 1. Increase breastfeeding 2. Give extra fluids 3. Continue feeding 4. Give ORS 5. Give antibiotic by mouth 6. Give antimalarial by mouth 7. Give vitamin A by mouth 8. Return for follow-up visit 9. Return or referred if worse 10. Complete the present referral 11. Other *(specify)*   99. Don’t know | | | | | | | | S6.20.1  1. □  2. □  3. □  4. □  5. □  6. □  7. □  8. □  9. □  10. □  11. □ ______________  99. □ ***→ SQ6.22*** | | | | | S6.35.1  1. □  2. □  3. □  4. □  5. □  6. □  7. □  8. □  9. □  10. □  11. □ ______________  99. □ ***→ SQ6.37*** | |
| Were you able to follow all the advice?  Kin iya kin bi duk shawarwarin da aka ba ki? | | | | | 1. Yes 2. No   9. Don’t know | | | | | | | | S6.21  🞎 ***9 → SQ6.22*** | | | | | S6.36  🞎 ***9 → SQ6.37*** | |
| *If not able to follow all the advice, ask:* Did you have any concerns or problems that kept you from following the advice?  Shin kina da wasu uzurori ko lalluran da suka hana ki bin shawarwarin?  *If able to follow all the advice, ask:* Did you have to overcome any concerns or problems to follow the advice?  Shin sai da ki ka ciwo kan wasu uzurori ko lalluran kafin ki ka iya bin shawarwarin? | | | | | 1. Yes 2. No   9. Don’t know | | | | | | | | S6.21.1  🞎 ***2 or 9 → SQ6.22*** | | | | | S6.36.1  🞎 ***2 or 9 → SQ6.37*** | |
| What concerns or problems did you have?  Shin wadanne uzurori ko lalluran ki ka samu?  *Prompt:* Was there anything else?  *Da akwai wani abun kuma?*  *[Multiple answers allowed.]* | | | | | 1. Did not understand instructions 2. Too much time from regular duties 3. Someone else *(specify)* decided 4. Cost too much 5. Problem required traditional care 6. Thought adivised care not needed 7. Thought care might harm the child 8. Thought child will die despite care 9. No time before go to next provider 10. The child died too soon 11. Other *(specify)*   99.Don’t know | | | | | | | | S6.21.2  1. □  2. □  3. □ _______________  4. □  5. □  6. □  7. □  8. □  9. □  10. □  11. □ ______________  99. □ | | | | | S6.36.2  1. □  2. □  3. □ _______________  4. □  5. □  6. □  7. □  8. □  9. □  10. □  11. □ ______________  99. □ | |
|  | | | | | | | | | | | | | | | | | |  | |
| At the time of leaving the a lokacin barin wajen (<FIRST HEALTH PROVIDER> / <LAST HEALTH PROVIDER>), was <NAME>…  *[Read the choices for each condition.]* | | | | | 1Feeding normally, poorly, or not at all  Ci dadai da kullum, ci kadan,ko babu ci sam  2Alert, drowsy, or unconscious kuzari,babu kuzari ko na nan a sume  3Normally active, less active than normal, or not moving da karfi daidai da kullum, kasa da kullum ko babu motsi | | | | | | | | S6.22  Nrml Med Abnrm DK  1. □ 2. □ 3. □ 9. □  1. □ 2. □ 3. □ 9. □  1. □ 2. □ 3. □ 9. □ | | | | | S6.37  Nrml Med Abnrm DK  1. □ 2. □ 3. □ 9. □  1. □ 2. □ 3. □ 9. □  1. □ 2. □ 3. □ 9. □ | |
| *If not taken to another health provider, ask:* Did you have any concerns or problems that kept you from taking <NAME> to another health provider?  Shin kina da wasu uzurori ko lalluran da suka hana ki kai <NAME> wata cibiyar kiwon lafiyar?  *If taken to another health provider, ask:* Did you have to overcome any concerns or problems to take <NAME> to another health provider?    Shin sai da ki ka ciwo kan wasu uzurori ko lalluran kafin ki ka kai <NAME> wata cibiyar kiwon lafiyar? | | | | | 1. Yes 2. No   9. Don’t know | | | | | | | | S6.23  🞎 ***2 or 9 → Inst_4*** | | | | | S6.38  🞎 ***2 or 9 → SQ6.39*** | |
| What concerns or problems did you have?    Shin wadanne uzurori ko lalluran ki ka samu?  *Prompt:* Was there anything else?  *Da akwai wani abun kuma?*  *[Multiple answers allowed.]* | | | | | 1. Thought no more care needed 2. No one available to go with her 3. Too much time from regular duties 4. Someone else *(specify)* decided 5. Too far to travel 6. No transportation available 7. Cost (transport, health care, other) 8. Not satisfied with available care 9. Problem required traditional care 10. Thought child too sick to travel 11. Thought child will die despite care 12. Was late at night 13. The child died before going 14. Other *(specify)*   99.Don’t know | | | | | | | | S6.23.1  1. □  2. □  3. □  4. □ _______________  5. □  6. □  7. □  8. □  9. □  10. □  11. □  12. □  13. □ ***→ SQ6.39***  14. □ ______________  99. □ | | | | | S6.38.1  1. □  2. □  3. □  4. □ _______________  5. □  6. □  7. □  8. □  9. □  10. □  11. □  12. □  13. □  14. □ ______________  99. □ | |
| ***Inst_4: Check SQ6.5 → If taken to another health provider…*** | | | | | | | | | | | | | ***…go to SQ6.24***  ***(LAST PROVIDER)*** | | | | |  | |
| S6.39 | How many days after (first noticing the illness / <LAST ACTION SQ6.5> / leaving the first/last health provider) did <NAME> die?  Shin bayan kwana nawa da ( aka fara ganin rashin lafiyar / <LAST ACTION SQ6.5>/ barin wajen first/last health provider) <NAME> ya/ta rasu?  *[If SQ6.4 = 2 (No care given), then read: “…first noticing the illness…”]* | | | | | | | | | | | | | | **__ __** Days  *(<1 = 00; DK = 99)* | | | | |
| ***Inst_5: If SQ6.4 = 2 (No care given) or***  ***if SQ6.5 ≠ “Health Provider” (Never took and never tried to take to a health provider) → VQ5.10*** | | | | | | | | | | | | | | | | | | | |
| **VA Section 5: Health records (FOR STILLBIRTHS, NEONATAL & CHILD DEATHS 0—59 MONTHS OLD)** | | | | | | | | | | | | | | | | | | | |
| V5.4 | Do you have any health records that belonged to the deceased?  Shin kina da wani katin asibitin marigayi/marigayiyar? | | | | | | 1. Yes 2. No   9. Don’t know | | | | | | | | | 🞎 ***2 or 9 → VQ5.10*** | | | |
| V5.5 | Can I see the health records?  Zan iya ganin katin asibitin? | | | | | | 1. Yes 2. No | | | | | | | | | 🞎 ***2 → VQ5.10*** | | | |
| V5.6 | *Record the dates of the two most recent visits* | | | | | | | | | | | | | | | **__ __/__ __/__ __ __ __**  D D M M Y Y Y Y  *(DK = 99/99/9999)* | | | |
|  |  |  |  |  |  |  |  |  |  |  |  |  |  |  |  | **__ __/__ __/__ __ __ __**  D D M M Y Y Y Y  *(DK = 99/99/9999)* | | | |
| V5.7 | *Record the two most recent weights on those dates* | | | | | | | | | | | | | | | **__ __ __ __** Grams  *(DK = 9999)* | | | |
|  |  |  |  |  |  |  |  |  |  |  |  |  |  |  |  | **__ __ __ __** Grams  *(DK = 9999)* | | | |
| V5.8 | *Record the date of the last note* | | | | | | | | | | | | | | | **__ __/__ __/__ __ __ __**  D D M M Y Y Y Y  *(DK = 99/99/9999)* | | | |
| V5.9 | *Transcribe the note* | | | | | | | | | | | | | | | | | | |
|  | ________________________________________________________________________________________________  ________________________________________________________________________________________________  ________________________________________________________________________________________________  ________________________________________________________________________________________________  ________________________________________________________________________________________________  ________________________________________________________________________________________________  ________________________________________________________________________________________________ | | | | | | | | | | | | | | | | | | |
| V5.10 | Was a death certificate issued?  An bada kati mai nuna shaidar ya/ta rasu? | | | | | | | 1. Yes 2. No   9. Don’t know | | | | | | | | 🞎 ***2 or 9 → SQ1.1*** | | | |
| V5.11 | Can I see the death certificate?  Zan iya ganin katin mai nuna shaidar ya/ta rasu? | | | | | | | 1. Yes 2. No | | | | | | | | 🞎 ***2 → SQ1.1*** | | | |
| V5.12 | *Record the immediate cause of death from the death certificate* | | | | | | |  | | | | | | | | | | | |
| V5.13 | *Record the first underlying cause of death from the death certificate* | | | | | | |  | | | | | | | | | | | |
| V5.14 | *Record the second underlying cause of death from the death certificate* | | | | | | |  | | | | | | | | | | | |
| V5.15 | *Record the third underlying cause of death from the death certificate* | | | | | | |  | | | | | | | | | | | |
| V5.16 | *Record the contributing cause of death from the death certificate* | | | | | | |  | | | | | | | | | | | |
| **SA Module 1: The mother and her household (FOR STILLBIRTHS, NN & CHILD DEATHS 0—59 MONTHS OLD)**  *Read:* Now I would like to ask you some other questions about (yourself / the child’s mother).  *[Read “…the child’s mother.” If the respondent is not the mother.*  *Yanzu ina son in yi maki tambayoyi gameda (ke kan ki/mahaifiyar)?* | | | | | | | | | | | | | | | | | | | |
| ***Inst_1: If GQ4.3 = 1 (Respondent is the mother) →*** ***SQ1.4*** | | | | | | | | | | | | | | | | | | | |
| S1.2 | How old (is the child’s mother / was the child’s mother when she died)?  Shin shekarun (ki nawa ne/mahaifiyar nawa ne a lokacin da ta rasu)?  *[Read “…was the child’s mother…” if she died.]* | | | | | | | | | | | | | | | __ __ Years  *(DK = 99)* | | | |
| S1.3 | How many years of school did the mother complete?  Shin shekaru nawa mahaifiyar ta kamala a makaranta? | | | | | | | | | | | | | | | **__ __** Years  *(<1 = 00; DK = 99)* | | |  |
| S1.4 | (Are you / Is/Was the child’s mother)…  *(Ko kina da/ Mahaifiyar ta na da)*    *[Read “…Is/Was the child’s mother…” if the respondent is not the mother.]*    *[Read the choices to the respondent.]* | | | | | | | 1. Married? Aure 2. Living with a man? Zama da na miji 3. Widowed? Mijin ya rasu 4. Divorced, separated, or deserted? Aure yua mutu, an rabu ko mijin ya tafi kawai? 5. Single (never married/lived w/a man)? Ba aure (ba a taba yin aure ba/ zama da na miji)   9. Don’t know | | | | | | | | 🞎 ***5 or 9 → Inst_2*** | | | |
| S1.4.1 | How old (were you when you / was she when she) first married (or lived with a man)?  Shin shekarun (ki nawa ne lokacin da ki ka/ta nawa ne lokacin da ta) fara aure (ko zama tare da na miji)?  *[Read “…was she when she…” if the respondent is not the mother.]*  *[Read “…married or lived with a man?” if SQ1.4 = “2. Living with a man”]* | | | | | | | | | | | | | | | __ __ Years  *(DK = 99)* | | | |
| S1.4.2 | How many years of school did (your / her) (husband / partner) complete?  Shin shekaru nawa ne ( mijn ki/mijin mahaifiyar) (abokin zaman ki/ta) ya kamala a makaranta?  *[Read “…her…” if the respondent is not the mother.]*  *[Read “…partner…” if she is living with a man.]* | | | | | | | | | | | | | | | **__ __** Years  *(<1 = 00; DK = 99)* | | | |
| ***Inst_2:*** *Read:* Now I would like to ask you some questions about (your / the mother’s) household. Please remember that all information will be kept confidential.  Yanzu ina son in tambayeki gameda gidan( ki/mahaifiyar). Muna sanar da ke cewa dukkan bayanan da muka rubuta za’a aje su cikin sirri.  *[SBs & NN deaths: If the respondent is not the mother, read “…the mother’s…;” and ask SQ1.5–1.11 about the mother’s household.*  *Older deaths: Always read “…your…;” and ask SQ1.5–1.11 about the respondent’s household.]* | | | | | | | | | | | | | | | | | | | |
| S1.5 | Who was the main breadwinner of (your / the mother’s) family during the (last days of the pregnancy / child’s fatal illness)  *Wanene ke kula da iyalin (ki/mahaifiyar) a yayin (karshen cikin/lokacin mummunar rashin lafiyar dan/yar)*  *[SBs~~/~~NN deaths: Read “…last days…”;*  *Older deaths: Read “…child's…”]* | | | | | | | 1. Child’s father 2. Child’s mother 3. Other   9. Don’t know | | | | | | | | 🞎 ***9 → SQ1.7*** | | | |
| S1.6 | At that time, what kind of work did the main breadwinner mostly do?  A wancan lokacin, wanne irin aiki mai kulawa da iyalin yafi yi? | | | | | | | 1. Farmer/agricultural worker 2. Poultry or cattle raising 3. Domestic servant 4. Home-based manufacturing 5. Unskilled laborer 6. Semi-skilled laborer/service provider 7. Factory worker, blue collar service 8. Business owner 9. Professional/technician 10. Other *(specify)* 11. Overseas worker   99. Don’t know | | | | | | | | 🞎🞎  ___________________________ | | | |
| S1.7 | Is this the house (where we are now) where (you / the mother) stayed during the (last days of the pregnancy / child’s fatal illness)?  *A wannan (*gidan da muke ciki yanzu,) anan ne (ki ke/mahaifiyar ke) da zama (a yayin karshen cikin, /lokacin mummunar rashin lafiyar dan/yar)?  *[SBs/NN deaths: Read “…last days…”*  *Older deaths: Read “…child's…”]*  *[Read “…where we are now…” if needed to clarify which house you are talking about.]* | | | | | | | 1. Yes 2. No   9. Don’t know | | | | | | | | 🞎 ***1 → SQ1.10***  ***9 → VQ5.17*** | | | |
| S1.8 | Where did (you / the mother) stay at that time?  *A ina (ki /mahaifiyar) ke da zama a wannan lokacin?*    *Probe:* Where did (you / the mother) stay during the illness events?  *A ina (ki /mahaifiyar) ke zama a lokacin lallurorin rashin lafiyar?*  *[Mark “1” only if her usual residence was not her in-laws or other relatives.]* | | | | | | | 1. Her own home at that time (other than with her in-laws) *(Interviewer: Use this code just if she moved after the death.)* 2. Her in-law’s home 3. Her parent’s home 4. Her brother’s home 5. Other *(specify)*   9. Don’t know | | | | | | | | 🞎 ***9 → VQ5.17***  ___________________________ | | | |
| S1.9 | What is the address of the place where (you / she) stayed?  Menene adireshin wurin da (ki ka/mahaifiyar ta) zauna? | | | | | | | State ____________________________  LGA________________________ | | | | | | | | 🞎🞎🞎  🞎🞎🞎 | | | |
| S1.10 | At the time of the illness events, how long had (you / the mother / your <RELATIVES> / the mother’s <RELATIVES>) been living continuously in (this / that) community?  *A lokacin lallurorin rashin lafiyar, har tsawon wanne lokaci ne (ki ka/mahaifiyar ta ke <dangin> ki ke>/ <dangin> mahaifiyar ke )zama a cikin (wannan/wancan) al’ummar ?*  *[Read “…<RELATIVES…” if SQ1.8 = 2-5 (s/he stayed with her/his relatives)].* | | | | | | | | | | | | | | | __ __ Years  *(<1 = 00; DK =99)* | | | |
| S1.11 | How long does it take to reach the health provider or facility where (you / the mother) normally (go(es) / went) from (this / that) place?  *[Mark hours &/or minutes as needed: e.g. 01 hour, 30 minutes]*  *Menene tsawon lokacin da za’a iya dauka zuwa wajen jami’in kiwon lafiya ko cibiyar kiwon lafiya da (ki ka/mahaifiyar ta,( ke (je/zuwa) daga (wannan/wancan) wurin?*  *Mark hours &/or minutes as needed: e.g. 01 hour, 30 minutes]* | | | | | | | | | | | | | | | **__ __** Hours  *(DK = 99)* | | | |
|  |  |  |  |  |  |  |  |  |  |  |  |  |  |  |  | **__ __** Minutes  *(DK = 99)* | | | |
| ***Inst_3 → SQ2.1.1 (if including optional Module 2) or VQ5.17*** | | | | | | | | | | | | | | | | | | | |

| **SA Module 2: Social capital (FOR SBs, NN & CHILD DEATHS 0–59 MONTHS OLD)**  *Read:* Now, I have some questions about (your / the mother’s / your <RELATIVES’> / the mother’s <RELATIVES’>) community.    Yanzu ina son in yi maki tambayoyi game da al’ummar ( ki/mahaifiyar/ <dangin ki>/dangin < mahaifiyar>)  *[SBs and NN deaths: If the respondent is not the mother, read “…the mother’s…” or “…the mothers’ <RELATIVES’>...;” and ask SQ2.1.1–SQ2.3.1 about the mother and her community or her relatives’ community.*  *Older deaths: Always read “…your…” or “…your <RELATIVES’>…;” and ask SQ2.1.1–SQ2.3.1 about the respondent and her/his community or her/his relatives’ community.*  *All deaths: Ask about the relatives’ community if s/he stayed with her/his relatives during the illness events.]* | | | |
| --- | --- | --- | --- |
| S2.1.1 | In the last 3 years, did the people in the (village / neighborhood) work together on any of the following issues that affect the entire community or part of the community?  *A cikin shekaru 3 da suka wuce, ko mutanen wannan (kauyen/makwafta) sun yi aiki akan wadannan al’amurran da suka shafi dukkan wannan al’ummar ko wani bangaren al’ummar?*  *Read all the issues and mark (“X”) Yes, No or DK for each one; then enter the code.]* | 1. Education/schools- kan ilimi/makarantu 2. Health services/clinics kiwon lafiya/ cibiyar kiwon lafiya 3. Paid job opportunities –hanyar aiyukan biyan kudi 4. Credit/finance – bashi/ da makamantan su 5. Roads- titi 6. Public transportation –Sufuri hukuma 7. Water distribution- rabiyar ruwa 8. Sanitation services –kwashan shara 9. Agriculture- Noma 10. Justice/conflict resolution –Adalci/raba rigingimu 11. Security/police services –tsaro/yan sanda 12. Mosque/church/temple-Masallaci/Coci/ wajen addu’a. 13. Other   *(specify)* | Yes No DK  1. □ 2. □ 9. □  1. □ 2. □ 9. □  1. □ 2. □ 9. □  1. □ 2. □ 9. □  1. □ 2. □ 9. □  1. □ 2. □ 9. □  1. □ 2. □ 9. □  1. □ 2. □ 9. □  1. □ 2. □ 9. □  1. □ 2. □ 9. □  1. □ 2. □ 9. □  1. □ 2. □ 9. □  1. □ 2. □ 9. □  ___________________________ |
|  |  | *Code:*   1. One or more issues identified 2. No issue identified | 🞎 |
| S2.2 | (Were you / Was the mother) able to turn to any persons, groups or organizations in the community for help during (the pregnancy / (or) the child’s fatal illness)?  Shin ( kin/mahaifiyar ta) iya samun wadansu mutanen, yan agaji ko kungiyoyi cikin al’umma domin taimakawa a yayin (cikin/ ko lokacin mummunar rashin lafiyar?  *[Read “…the pregnancy?” for SBs; or “…the pregnancy or the child’s fatal illness?” for NN deaths; or “…the child’s fatal illness for older deaths.]* | 1. Yes 2. No   9. Don’t know | 🞎 ***2 or 9 → SQ2.3.1*** |
| S2.2.1 | Did (you / she) turn to any of the following for help?  Shin kin/ta nemi taimaiko daga wajen wadannan?  *[Read all the options and mark (“X”) Yes, No or DK for each; then enter the code.]* | 1. Family -Dangi 2. Neighbors- Makwabta 3. Friends-Abokai 4. Religious leader or group –Shuagabannin addini ko kungiya 5. Community leader- Shuagabannin hukuma 6. Police-Yan sanda 7. Patron/employer/benefactor Shugaba/ Shugaban wajen aiki, 8. Political leader- Shuagabannin siyasa 9. Mutual support group s/he belongs to-kungiyar gwadoko da ki/ka ke ciki 10. Assistance organization to which s/he does not belong- tamaika wa kungiyar gwadoko da ki/ka ke ciki 11. Other   *(specify)* | Yes No DK  1. □ 2. □ 9. □  1. □ 2. □ 9. □  1. □ 2. □ 9. □  1. □ 2. □ 9. □  1. □ 2. □ 9. □  1. □ 2. □ 9. □  1. □ 2. □ 9. □  1. □ 2. □ 9. □  1. □ 2. □ 9. □  1. □ 2. □ 9. □  1. □ 2. □ 9. □  1. □ 2. □ 9. □  ___________________________ |
|  |  | *Code:*   1. One person/group identified 2. Two or more persons/groups identified 3. No person/group identified | 🞎***3 → SQ2.3.1*** |
| S2.2.2 | (Is this / Are these) the same person(s) or group(s) (you / she) would usually turn to for help with a serious problem?  Shin (wanene ko wadanne ) mutumin (mutane) ne ( ki/ta) ke neman taimakonsu idan akwai wata babbar matsala? | 1. Yes 2. No   9. Don’t know | 🞎 |
| S2.3.1 | (Have you or your / Has the mother or her) family ever been denied any of the following community services?  *An taba hana (Ki ko ‘yar uwarki / uwar yaron/yarinyar ko wani dangin ta) ko, wani daga cikin wadannan abubuwan da al’umma ke gudanarwa?*  *Read all the options and mark (“X”) Yes, No or DK for each; then enter the code.]* | 1. Education/schools 2. Health services/clinics 3. Paid job opportunities 4. Credit/finance 5. Transportation 6. Water distribution 7. Sanitation services 8. Agricultural extension 9. Justice/conflict resolution 10. Security/police services 11. Other   *(specify)* | Yes No DK  1. □ 2. □ 9. □  1. □ 2. □ 9. □  1. □ 2. □ 9. □  1. □ 2. □ 9. □  1. □ 2. □ 9. □  1. □ 2. □ 9. □  1. □ 2. □ 9. □  1. □ 2. □ 9. □  1. □ 2. □ 9. □  1. □ 2. □ 9. □  1. □ 2. □ 9. □  ___________________________ |
|  |  | *Code:*   1. One service denied 2. Two or more services denied 3. No denied service identified | 🞎 |
| V5.165 | *Read:* Now I have four last questions about the child and the child’s mother. Before the fatal illness began, did <NAME> suffer from HIV/AIDS? | 1. Yes 2. No 3. Refused to answer 4. Don’t know | 🞎 |
| V5.17 | Yanzu ina da tambayoyi ukku da nake so inyi maki gameda mahaifiyar?  (Have you / Has the deceased’s biological mother) ever been tested for “HIV”?  (An taba yi maki ko mahaifiyar yaron/yarinyar gwanjin cutar kanjamau?) | 1. Yes  2. No  8. Refused to answer  9. Don’t know | 🞎 ***2-9 → VQ5.19*** |
| V5.18 | Was the “HIV” test ever positive?  Gwanjin kanjamau din da aka yi ya nuna cewa akwai cutar? | 1. Yes 2. No   8. Refused to answer  9. Don’t know | 🞎 |
| V5.19 | (Have you / Has the deceased’s biological mother) ever been told (you / she) had “AIDS” by a health worker?  Jami’in kiwon lafiya ya taba fadi (maki ko mahaifiyar marigayin) cewa (kina/tana) da cutar kanjamau? | 1. Yes 2. No   8. Refused to answer  9. Don’t know | 🞎 |

| **VA Section 6 & SA Module 7: Open ended response & interviewer comments/observations (FOR ALL DEATHS)**  *Read:* Thank you for answering the many questions that I’ve asked. Would you like to tell me about <NAME>’s illness in your own words? Also, is there anything else about her/his illness that I did not ask and you would like to tell me about?  *After the respondent(s) finishes, ask*: Is there anything else?  *Write the respondent’s exact words. After s/he has finished, read this back and ask her to correct any errors in what you wrote.*  *Ina godiya kwarai da gaske da amsa tambayoyin da nayi maki. Zan so ki fada mani gameda da ciwon da <*NAME> a cikin kalmominki? Akwai wani abu da yai saura gameda ciwon sa/ta da ban tambaya ba kuma ki ke so ki shaida mani.  Akwai wani abu kuma? |
| --- |
| _____________________________________________________________________________________________________  _____________________________________________________________________________________________________  _____________________________________________________________________________________________________  _____________________________________________________________________________________________________  _____________________________________________________________________________________________________  _____________________________________________________________________________________________________  _____________________________________________________________________________________________________  _____________________________________________________________________________________________________  _____________________________________________________________________________________________________ |

**END OF INTERVIEW**

**THANK RESPONDENT FOR HER/HIS PARTICIPATION**

| *Interviewer: Use this space to write down your comments and observations about the interview.* |
| --- |
| _____________________________________________________________________________________________________  _____________________________________________________________________________________________________  _____________________________________________________________________________________________________  _____________________________________________________________________________________________________ |

**Igbo VASA questionnaire**

| **VERBAL/SOCIAL AUTOPSY GENERAL INFORMATION (FOR SBs, NN & CHILD DEATHS 0—59 MONTHS OLD)**  **Section 1: Background about the deceased**  *Interviewer: Before going to the field to do the interview, fill in this section from the survey or surveillance record for the deceased*. | | | | | | |
| --- | --- | --- | --- | --- | --- | --- |
| G1.1 | Address of the household  *[Copy the household address]*  Kedu ebe ezinaulo a bi?  *[Deputa otu esi ga enweta ebe obibi ezinuola a ]* | State ____________________________  LGA ______________________  Locality _____________________  EA ________________________ | | | 🞎🞎🞎  🞎🞎🞎  🞎🞎🞎  🞎🞎🞎🞎 | |
|  | Directions to the household  *[Copy the directions to the household*  Kedu ebe esi aga na ezinauloa?  *[Deputa uzo aga esi enweta ebe obibi ezinuloa a ]* |  | | | | |
|  | Sketch a map if needed | | | | | |
| G1.2 | Name of the deceased (if known)  *[Copy the name of the deceased]*  Aha onye a nwuru anwu (o buru na ama)  *[Deputa Aha onye nwuru anwu]* |  | | | | |
| G1.3 | Sex of deceased  *[Copy the sex of the deceased]*  O bu nwoke ka o bu nwanyi?  *[Deputa ma onye a o bu nwoke ka o bu nwanyi]* | 1. Male 2. Female | | | 🞎 | |
| G1.4 | Date of birth of the deceased  *[Copy the day, month and year of birth of the deceased]*  Kedu mgbe a muru onye a nwuru anwu?  *[Deputa ubochi, onwa na afo muru onye a]* | | | | **__ __/__ __/__ __ __ __**  D D M M Y Y Y Y  *(DK = 99/99/9999)* | |
| G1.5 | Date of death of the deceased  *[Copy the day, month and year of death of the deceased]*  Kedu mgbe o nwuru?  *[Deputa ubochi, onwa na afo onye a nwuru]* | | | | **__ __/__ __/__ __ __ __**  *(DK = 99/99/9999)* | |
| G1.6 | Last known age of the deceased  Kedu afo ikpeazu o gbara tupu onwu a?  *[Copy the last known age of the deceased: Record days if less than 28 days—if less than 24 hours, record “00” days; Record months if 28 days-11 months; Record years if 1 year or older.]* | | | | **__ __** Days: ***1 or more → GQ1.7***  *(DK = 99)* | |
|  |  |  |  |  | **__ __** Months ***→ GQ1.7***  *(DK = 99)* | |
|  |  |  |  |  | **__ __** Years ***→ GQ1.7***  *(DK = 99)* | |
| G1.6.1 | Was this a stillbirth or neonatal death?  O nwuru na-afo tupu amuputa ya,ka o nwuru mgbe a muputachara ya?  *[Copy this information from the record]* | 1. Stillbirth 2. Neonatal death   9. Not known from the record | | | 🞎 | |
| G1.7 | Name of mother  Kedu Aha nne muru ya?  *[Deputa aha nne ya]* |  | | | | |
| G1.8 | Name of father  Kedu Aha nna muru ya?  *[Deputa aha nna ya ]* |  | | | | |
| **Section 2: Background about the interview**  *Interviewer: Tupu aju ajuju ma mgbe aju Chiara ajuju,debanye ihe ndia. Igaghi aju ya aajuju ndia.* | | | | | | |
| G2.1 | Language of the interview  Asusu e jiri juo ajuju? |  | | | | |
| G2.2 | Interviewer name and ID number  Aha na akara onye juru ajuju? | _________________________________ | | | 🞎🞎🞎🞎🞎 | |
| G2.3 | Dates of attempted and successful interviews  Ubochi mbu inwara ma azaghi ajuju a? | **DATE** | | | **RESULT OF THE INTERVIEW** | |
| G2.3.1 | Date of first interview attempt  Ubochi mbu inwara a juru ajuju? | **__ __/__ __/__ __ __ __**  D D M M Y Y Y Y | | | **Interim result:**  🞎 | |
| G2.3.2 | Date of second interview attempt  Ubochi nke abuo inwara iju ajuju a? | **__ __/__ __/__ __ __ __**  D D M M Y Y Y Y | | | **Interim result:**  🞎 | |
| G2.3.3 | Date of third interview attempt  Ubochi nke ato inwara iju ajuju a? | **__ __/__ __/__ __ __ __**  D D M M Y Y Y Y | | | **Interim result:**  🞎 | |
| G2.4 | Date interview started  Ubochi iju ajuju malitere?  *[Equals date of the last attempt]* | **__ __/__ __/__ __ __ __**  D D M M Y Y Y Y | | |  | |
| G2.5 | Time interview started  Oge iju ajuju malitere?  *[Record hour 1-24 / minutes 1-60]* | **__ __/__ __**  H R M M | | |  | |
| G2.6 | Date interview finished  Ubochi iju ajuju kwusiri?  *[Equals date started or a later date]* | **__ __/__ __/__ __ __ __**  D D M M Y Y Y Y | | | **Final result:**  🞎 | |
| G2.7 | Time interview finished  Oge iju ajuju a kwusiri?  *[Record hour 1-24 / minutes 1-60]* | **__ __/__ __**  H R M M | | |  | |
|  | **Interview result codes:**  1. Completed (Final result code)  2. Partially completed (Final result code)  3. Eligible respondent postponed interview  4. No eligible respondent at home at time of visit  5. Eligible respondent refused interview | | 6. No eligible respondent lives in household  7. No household member at home  8. Dwelling vacant / destroyed / not found  9. In progress (Interim result code)  10. Child reported dead in birth history is actually alive  11. Duplicate report of death – interview already conducted | | | |
| G2.8 | Date form checked by supervisor  Ubochi onye na enyocha ulo tulere akwukwo ijri juo ajuju a? | **__ __/__ __/__ __ __ __**  D D M M Y Y Y Y | | |  | |
| G2.9 | Date entered in computer  Ubochi edebara ajuju juru na komputa? | **__ __/__ __/__ __ __ __**  D D M M Y Y Y Y | | |  | |
| **INTERVIEW BEGINS**  *Instructions to interviewer: Introduce yourself and explain the purpose of your visit. Ask to speak to the mother or to another adult who was the deceased’s main caregiver during the illness that led to death. If this is not possible, arrange a time to revisit the household when the caregiver will be home. (See example below.)*  “My name is [your name]. I am an interviewer with the ____________ project. I have been informed that a child death has occurred in your household. I am very sorry to hear this. Please accept my sympathies. For the purpose of improving health care, we are collecting information on recent child deaths in this area. I would like to talk to the mother or main caregiver of <NAME> and ask some questions about the events and any symptoms that <NAME> had during her/his illness before death.”  *Instructions to interviewer: Kowa onwe gi ma kowakwa ihe mere iji biaJuo otuiga esi okwola nne ya ma obu okenye ozo na elekota onye nwuru anwu mgbe ona aria oria nke mere ka onwuo.Oburu na nke ekweghi hazie mgbe ozo I ga abiahachie,mgbe onye elokotara onye nwuru anwu ga anon a ulo.. (See example below.)*  Aha m bu ____________ a bu m onye o ju ajuju nke ndi------------. A gwara m na o dila mgbe nwata nwuru na ezinaulo gi.. O wutere m nke nkwuu.. Biko nabata nkasi obi m. I ji were kwado ihe gbasara ahuike, anyi na aju ajuju gbasara onwu umuntakiri n,obodo a nke mere na mgbe na adighi anya. O ga amasi m I ju nne o bu onye nlezi anya (AHA) ufodu ajuju gbasara ahu riara AHA tupu o nwu o.” | | | | | | |
| **Section 3: Consent**  ***INTERVIEWER: Read the consent form to the respondent. Ask the respondent if he or she has any questions. Once any questions are answered, ask the respondent if he or she is willing to take part in the study.***  ***INTERVIEWER: Guputa akwukwo iji gosi ma o nabatara iza ajuju ichoro iju ya.Ju o ya ma onwere ajuju obula ochoro iju gi.O buru na onweghi ajuju obula juo ya ma ochoro iso me nchoputa?*** | | | | | | |
| G3.1 | *INTERVIEWER: Did respondent give consent?*  *INTERVIEWER: O nabatara iza ajuju I choro iju ya?* | 1. Yes 2. No | | 🞎 ***2 → Thank respondent for their time and end the interview.*** | | |
| **Section 4: Information about the respondent**  *Read:* I would now like to ask you some general questions about yourself.  **Section 4: Ihe gbasra onye iga aju ajuju**  *Read:* O ga amasi m i ju gi ufodu ajuju gbasara onwe gi?. | | | | | | |
| G4.1 | What is your (the respondent’s) name?  Gini bu Aha gi? |  | | | | |
| G4.2 | *INTERVIEWER: What is the sex of the respondent?*  *INTERVIEWER: Onye na aza ajuju o bu nwoke ka o bu nwanyi?* | 1. Male 2. Female | | | 🞎 | |
| G4.3 | What is your relationship to the deceased child?  Gini jikoro gi na nwata a nwuru anwu? | 1. Mother 2. Father 3. Grandmother 4. Grandfather 5. Aunt 6. Uncle 7. Brother 8. Sister 9. Birth attendant *(specify type)* 10. Other male *(specify)* 11. Other female *(specify)* | | 🞎🞎  ___________________________  ___________________________  ___________________________ | | |
| G4.4 | How old are you?  Afo ole ka I gbara? | | | __ __ Years  *(DK = 99)* | | |
| G4.5 | How many years of school did you complete? *[Do not include repeated years.]*  Afo ole ka i guchara na ulo akwukwo? *[E tinyela nke iguagariri aguhari.]* | | | **__ __** Years  *(<1 = 00; DK = 99)* | | ***>6 years***  ***→ GQ4.6*** |
| G4.5.1 | Now I would like you to read this sentence to me. (*Show card to respondent)*  Ugbua o ga amasi m ka I guputara ahiri okwu ndia. (*Show card to respondent)*  *If she cannot read the whole sentence, probe:* Can you read any part of the sentence to me?  *O buru na ogaghi aguputa ahiriokwu nile edere na akwukwo, probe:* O nwere mkpuruokwu I ga enwe ike I guputa na ahiriokwu a? | 1. Cannot read at all 2. Able to read only part of sentence 3. Able to read whole sentence 4. No card available to show mother | | 🞎 | | |
| *Read:* I would now like to ask you some questions about (your / the family’s) household. Please remember that all information will be kept confidential.  *Read:*  o ga amasi kwa m i ju gi ufodu ajuju gbasara gi / ezinaulo .Biko cheta kwa na ihe obula a natara gi a gaghi egosi ya onye ozo.  *Read "...the family's household.” if you are not conducting the interview at the household where the death was identified.]*  *[Read "...O buru na inaghi aju ajuju na ezinaulo ebe achoputara na madu nwuru anwu.]* | | | | | | |
| G4.6 | How many people live at (this / that) household?  Madu ole bi na ezinaulo a / ezinaulo gi?  *[Read “…at that household?” if you are speaking of “the family’s household.”]*  *[Guo: na ezinulo?” oburu na ina ekwu maka ezinaulo a.”]* | | | __ __ People  *(DK = 99)* | | |
| G4.7 | How many sleeping rooms are in the household?  Onu ulo ole ebe a na arahu ura di na ezinulo a? | | | __ __ Rooms  *(DK = 99)* | | |
| G4.8 | Does the household have a separate room for cooking?  Ezinulo a enwere onu ulo eweputara maka isi nri? | 1. Yes 2. No   9. Don’t know | | 🞎 | | |
| G4.9 | Does the household have:  Ezinuloa e nwere ihe ndi a?:  *[Ask about each possession, and mark each one “Yes,” “No” or “Don’t know.”]* | Ezinaulo a enwere? Does the household have:   1. Oku latrik? electricity? 2. Radio? 3. Onyonyio? a television? 4. Njuoyi-frig? a refrigerator? 5. Igwe wire? a fixed line telephone?   O nwere: Does it have:   1. Ekwa- nti? a mobile telephone? 2. Komputa? a computer? 3. Anyinya- igwe? a bicycle? 4. Ugboala m’obu nke ibu? a car or truck?   Ezinaulo a enwere? Does the household have:   1. Miri pomp esepuru na- ebe obibi a? 2. Miri well (ekpuchiri ekpuchi m’obu ekpuchighi)? 3. Miri mbughari m’obu nke moto miri m’obu nke ololo? 4. Miri n’ekwo ekwo? | | Yes No DK  1. □ 2. □ 9. □  1. □ 2. □ 9. □  1. □ 2. □ 9. □  1. □ 2. □ 9. □  1. □ 2. □ 9. □    1. □ 2. □ 9. □  1. □ 2. □ 9. □  1. □ 2. □ 9. □  1. □ 2. □ 9. □    1. □ 2. □ 9. □  1. □ 2. □ 9. □    1. □ 2. □ 9. □  1. □ 2. □ 9. □ | | |
| G4.10 | What type of toilet does the household have?  Kedu udi ulo mposi ezinulo a nwere? | 1. Mposi eji miri asapu 2. Mposi olulu edoziri edozi 3. Mposi olulu nke odin’ala 4. Ohia/iro/akuku miri 5. Ebe ndi ozo *(specify)*   9. Amaghi | | 🞎  ___________________________ | | |
| G4.11 | What is the main kind of energy the household uses for cooking?  Kedu ihe ezinulo a ji esikari nri? | 1. Unyi 2. Nku 3. Kerozin 4. Oku latrick 5. Gas 6. Nsi ehi 7. Ebe ndi ozo *(specify)*   9. Amaghi | | 🞎  ___________________________ | | |
| G4.12 | What is the main material used for the floor of the house?  Kedu ihe e jiri mee ala ulo ezinulo a? | 1. Aja/Apiti 2. Ntu cement 3. Osisi 4. Tyl 5. Ebe ndi ozo *(specify)*   9. Amaghi | | 🞎  ___________________________ | | |

| **Section 5: Information about others at the interview** | | | | | | | | |
| --- | --- | --- | --- | --- | --- | --- | --- | --- |
| G5.7 | | *INTERVIEWER: Onwere ndi ozo no oge ana aju ajuju a?* | | 1. Yes 2. No | | 🞎 ***2 → GQ5.9*** | | |
| G5.8 | | *INTERVIEWER: Tinyere onye- ana aju ajuju,madu ole ndi ozo no oge ana aju ajuju a?* | | | | __ __ Other people  *(DK = 99)* | | |
| G5. | 9 | *INTERVIEWER: Dee ma onye ana aju ajuju ma o no oge nwa n’aria oria ma o bu oge onwuru.-. Na onye nke ozo nile no-oge ana- aju ajuju juo onye ana aju ajuju ihe jikoro ya na onye nwuru anwu ya na ma ha no oge nwa n’aria oria ya na/m’obu oge onwuru. Maka ndi nwuru n’afo nan di nwuru n’ime out onwa amuchara ha,jukwa ma onye obula (abughi nne nwa) ano oge nne nwaa di ime na mgbe omuru nwa?.* | | | | | | |
|  |  | Relationship of person to the deceased child | Mark (X)  if present at the interview | Stillbirths and neonatal deaths only | | | Neonatal & older child deaths only | |
|  |  |  |  | Present during  the pregnancy:  1. Yes / 2. No | Present at  the delivery:  1. Yes / 2. No | | Present during child’s illness:  1. Yes / 2. No | Present at the child’s death:  1. Yes / 2. No |
|  | .1 | Mother | 🞎 |  |  | | 🞎 | 🞎 |
|  | .2 | Father | 🞎 | 🞎 | 🞎 | | 🞎 | 🞎 |
|  | .3 | Grandmother | 🞎 | 🞎 | 🞎 | | 🞎 | 🞎 |
|  | .4 | Grandfather | 🞎 | 🞎 | 🞎 | | 🞎 | 🞎 |
|  | .5 | Aunt | 🞎 | 🞎 | 🞎 | | 🞎 | 🞎 |
|  | .6 | Uncle | 🞎 | 🞎 | 🞎 | | 🞎 | 🞎 |
|  | .7 | Brother | 🞎 | 🞎 | 🞎 | | 🞎 | 🞎 |
|  | .8 | Sister | 🞎 | 🞎 | 🞎 | | 🞎 | 🞎 |
|  | .9 | Traditional birth attendant | 🞎 | 🞎 | 🞎 | | 🞎 | 🞎 |
|  | .10 | Other male (specify:  __________________) | 🞎 | 🞎 | 🞎 | | 🞎 | 🞎 |
|  | .11 | Other female (specify  __________________) | 🞎 | 🞎 | 🞎 | | 🞎 | 🞎 |

| **VA Section 1: Background (FOR STILLBIRTHS, NEONATAL & CHILD DEATHS 0—59 MONTHS OLD)** | | | | |
| --- | --- | --- | --- | --- |
| V1.1 | Was the deceased a singleton or multiple birth?  Nwaa nwuru anwu o bu ejima k,amuru so ya?  *[If two or more children are born at the same time, it is counted as a multiple birth, even if one or more of the babies are born dead.]* | | 1. Singleton 2. Multiple   9. Don’t know | 🞎 ***1 or 9 → VQ1.3*** |
| V1.2 | Was this the first, second, or later in the birth order?  O bu nwa mbu,nke abua k,obu nke ole n,usoro omumu? | | 1. First 2. Second 3. Third or more   9. Don’t know | 🞎 |
| V1.3 | *If the mother is present, mark “Yes” and do not ask this question.*  Is the mother still alive?  Nne ya odi ndu? | | 1. Yes 2. No | 🞎 ***1 → VQ1.6*** |
| V1.4 | Did the mother die during or after the delivery?  Nne ya onwuru ebe o na-amu nwa ka o bu ka o muchara nwa? | | 1. During 2. After   9. Don’t know | 🞎 ***1 or 9 → VQ1.6*** |
| V1.5 | How long after the delivery did the mother die?  Ogologo oge ole o muchara nwa k o nwuru?  *[Record days if less than 28 days—if less than 24 hours, record “00” days;*  *Record months if 28 days or more]* | | | **__ __** Days  *(DK = 99)* |
|  |  |  |  | **__ __** Months  *(DK = 99)* |
| V1.6 | Where was the deceased born?  Kedu ebe a muru nwata a nwuru anwu? | | 1. Hospital 2. Other health provider or facility 3. On route to a health provider or facility 4. Home 5. Other *(specify)*   9. Don’t know | 🞎  ___________________________ |
| V1.7 | At the time of the delivery was the deceased:  Na oge omumu ya nwata a nwuru anwu :  *[Read the question and slowly read the first four choices. Respondent should hear all four choices & then respond.]*  *[Show photos]* | | 1. Pere mpe nnukwu 2. Pekariri out okwusiri l 3. Out Okwesiri 4. Bukariri out okwesiri   9.Amaghi Don’t know | 🞎 |
| V1.8 | What was the weight of the deceased at birth?  Kedu ihe nwata a nwuru anwu dara mgbe a muru ya? *[Ask to see the child’s health card. If the card is available, record the birth weight from the card.]* | | | **__ __ __ __** Grams  *(DK = 9999)* |
| V1.9 | What was the sex of the deceased?  Nwata a nwuru anwu o bu nwoke ka o bu nwanyi? | | 1. Male 2. Female   9. Don’t know | 🞎 |
| V1.10 | What was the delivery date?  Kedu mgbe amuru nwata a nwuru anwu?  *Compare the delivery date just stated by the respondent to the birth date from the prior record (GQ1.4). Discuss any inconsistency with the respondent to confirm or correct the stated delivery date. You cannot change the prior record’s date.* | | | **__ __/__ __/__ __ __ __**  D D M M Y Y Y Y  *(DK = 99/99/9999)* |
| V1.11 | Was the child born alive or dead?  Mgbe a muputara nwata a,o di ndu ka o nwuru anwu? | | 1. Alive 2. Dead   9. Don’t know | 🞎 |
| V1.12 | Did the baby every cry?  Mgbe a muputara nwata a, o bere akwa? | | 1. Yes 2. No   9. Don’t know | 🞎 |
| V1.13 | Did the baby ever move?  Mgbe a muputara nwata a o di o meghariri ahu? | | 1. Yes 2. No   9. Don’t know | 🞎 |
| V1.14 | Did the baby ever breathe?  Nwata ao di mgbe o kuru ume? | | 1. Yes 2. No   9. Don’t know | 🞎 |
| V1.15 | *Refer to VQ1.11–1.14. If “Dead” & no crying, movement or breathing, mark “Stillbirth.” If “Alive” & VQ1.12–1.14 = “No,” or if “Dead” and VQ1.12, 1.13 or 1.14 = “Yes,” then discuss & correct.* | | 1. Stillbirth 2. Live birth | 🞎 ***2 → VQ1.20*** |
| **Stillbirths** | | | | |
| V1.16 | Were there any bruises or signs of injury on the baby’s body at birth?  Mgbe a muru nwata a, o nwere ihe mmeru ahu ma obu onya n,ahu ya? | | 1. Yes 2. No   9. Don’t know | 🞎 |
| V1.17 | Was the baby’s body (skin and tissue) pulpy?  Ahu nwa ahu (akpukpo ahu m’obu anu ahu)ona ede-ede? | | 1. Yes 2. No   9. Don’t know | 🞎 |
| V1.18 | Was any part of the baby physically abnormal at the time of delivery? (for example: body part too large or too small, additional growth on body)  Mgbe a muputara nwata a o di akuku ahu ya adighi otu okwesiri? (dika o nwere nke bukariri ma obu pekaria otuokwesiri ma obu nke putara ebe o kwesighi I puta) | | 1. Yes 2. No   9. Don’t know | 🞎 ***2 or 9 → SQ3.1*** |
| V1.19 | What were the abnormalities?  Kedu ihe ndi ahu adighi otu o kwesiri?  *Ask for the following abnormalities*  *[Mark all that apply – Show photos]* | | 1. Isi ya opere nnukwu mpe oge amuru ya 2. Isi ya oburu nnukwu ibu oge amuru ya? 3. Enwere orusi di ya n’azu isi m’obu na-ekwe ya? 4. Onwere orusi ozo *(If “Yes,” then specify)* | Yes No  1. □ 2. □    1. □ 2. □  1. □ 2. □  1. □ 2. □ __________________ |
| ***Inst_1: STOP. After completing VQ1.19 → SQ3.1 (Maternal history)*** | | | | |
| **Live births** | | | | |
| V1.20 | | How old was the child when the illness started?  Afo ole ka nwata gbara oge o malitere iria oria?  *[Record days if less than 28 days—if less than 24 hours, record “00” days;*  *Record months if 28 days-11 months;*  *Record years if 1 year or older.]* | | **__ __** Days  *(DK = 99)* |
|  |  |  |  | **__ __** Months  *(DK = 99)* |
|  |  |  |  | **__ __** Years  *(DK = 99)* |
| V1.21 | | How long did the illness last?  Ogologo oge ole ka o riara oria ahu?  *[Record days if less than 28 days—if less than 24 hours, record “00” days;*  *Record months if 28 days or more.]* | | **__ __** Days  *(DK = 99)* |
|  |  |  |  | **__ __** Months  *(DK = 99)* |
| V1.22 | | Where did the deceased die?  Ebe ka nwa a nwuru anwu no nwuo? | 1. Hospital 2. Other health provider or facility 3. On route to a health provider or facility 4. Home 5. Other *(specify)*   9. Don’t know | 🞎  ___________________________ |
| V1.24 | | What was the date of death?  Kedu onwa na afo nwata a nwuru?  *Compare the date of death just stated by the respondent to the date of death from the prior record (GQ1.5). Discuss any inconsistency with the respondent to confirm or correct the stated date. You cannot change the prior record’s date.* | | **__ __/__ __/__ __ __ __**  D D M M Y Y Y Y  *(DK = 99/99/9999)* |
| V1.25 | | ***AGE AT DEATH***  ***Record only the calculated age OR the stated age. First try to calculate the age. If this is not possible, then ask the respondent for the child’s age at death.*** | | |
|  |  | *CALCULATE THE AGE AT DEATH*  *Record the delivery date from VQ1.10:* **__ __/__ __/__ __ __ __**  D D M M Y Y Y Y  *(Don’t Know = 99/99/9999)*  *Record the date of death from VQ1.24:* **__ __/__ __/__ __ __ __**  D D M M Y Y Y Y  *(Don’t Know = 99/99/9999)*  *Now, if possible, calculate the age at death (VQ1.24 – VQ1.10). If only the month and year are known, you may still be able to calculate the approximate age in months or years. Discuss the calculated age with the respondent:* I have calculated that the child was (about) <CALCULATED AGE> at death. Is this correct?  *If the respondent does not agree with the calculated age, then again discuss the delivery date and date of death to make sure that these are correct. If the calculated age at death cannot be resolved, then go below to the “STATED AGE” box.*  *Once the age at death is calculated, check VQ1.20 and VQ1.21 to make sure that the age at illness onset and the illness duration are consistent with the age at death. For example, the age at onset + duration cannot be greater than the age at death.*  *[Record days if less than 28 days—if less than 24 hours, record “00” days; Record months if 28 days-11 months; Record years if 1 year or older.]*  *After recording the calculated age* ***→ VQ1.26*** | | **__ __** Days ***(if < 28 days)***  *(DK = 99)* |
|  |  |  |  | **__ __** Months ***(if 1-11 months)***  *(DK = 99)* |
|  |  |  |  | **__ __** Years ***(if 1 year or older)***  *(DK = 99)* |
|  |  | *STATED AGE AT DEATH (Ask only if the calculated age cannot be determined)*  How old was the deceased at the time of death?  Afo ole ka nwa nwuru anwu gbara oge onwuru?  *Compare the age at death just stated by the respondent to the child’s last known age from the prior record (GQ1.6). Discuss any inconsistency with the respondent to confirm or correct the stated age. You cannot change the prior record’s age. Partly known delivery and death dates might help resolve the stated age. For example, if the child was born and died in the same month, then this is likely a neonatal death.*  *Once the age at death is determined, check VQ1.20 and VQ1.21 to make sure that the age at illness onset and the illness duration are consistent with the age at death. For example, the age at onset + duration cannot be greater than the age at death.*  *[Record days if less than 28 days—if less than 24 hours, record “00” days; Record months if 28 days-11 months; Record years if 1 year or older.]* | | **__ __** Days ***(if < 28 days)***  *(DK = 99)* |
|  |  |  |  | **__ __** Months ***(if 1-11 months)***  *(DK = 99)* |
|  |  |  |  | **__ __** Years ***(if 1 year or older)***  *(DK = 99)* |
| V1.26 | | *Mark the baby’s age at the time of death.*  *[Use the calculated age (VQ1.24 – VQ1.10) if known, or the stated age (VQ1.25). If both the calculated and stated ages are unknown, then use your best judgment to mark the child’s age at death.]* | 1. Less than 28 days old 2. 1-59 months old | 🞎 ***2 → SQ5b.1*** |

| **SA Module 3 and VA Section 2: Maternal history (FOR STILLBIRTHS AND NN DEATHS < 28 DAYS OLD)**  *Read:* Now, I would like to ask you some questions about (your / the mother’s) health and (your / her) pregnancy with <NAME>.  *Read:* Ugbua a chorom iju gi ufodu ajuju gbasara (Ahuike (gi / Ahuike nne ya) mgbe (I / nne ya) di ime Aha.  *Here and in the following questions, read “…the mother…,” “…her…” and “…she…” if the mother is not the respondent.* | | | | |
| --- | --- | --- | --- | --- |
| S3.1 | Before the pregnancy with <NAME>, did (you / the mother) suffer from any of the following known conditions:  Before the pregnancy with <NAME>, did (you / the mother) suffer from any of the following known conditions  Tupu a di ime AHA (I / nee ya o nwere nsogbu ahuike ndi a?  *[Read out all options and check “Yes,” “No” or “Don’t know” for each.]*  *If “Yes,” then ask:* Did (you / she) undergo treatment for this condition during the pregnancy?  *If “Yes,” then ask:* Gi / nne ya o natara nlezi anya gbasara nsogbu ahuikea oge o di ime? | 1. Obara mgbali elu 2. Oria obi 3. Oria suga/Mamiri 4. Akwukwu / ihe odido 5. Ihe ndi ozo------------------------------   *(specify other)* | Suffered from  Yes No DK  1. □ 2. □ 9. □  1. □ 2. □ 9. □  1. □ 2. □ 9. □  1. □ 2. □ 9. □  1. □ 2. □ 9. □ | Treatment  Yes No DK  1. □ 2. □ 9. □  1. □ 2. □ 9. □  1. □ 2. □ 9. □  1. □ 2. □ 9. □  1. □ 2. □ 9. □ |
|  |  |  | ___________________________ | |
| S3.2 | During the pregnancy, did (you / the mother) see anyone for antenatal care?  Na oge afo ime (i/nne ya o natara enyemuaka nke a n, enye umunwanyi di ime? | 1. Yes 2. No   9. Don’t know | 🞎 ***2 or 9 → SQ3.3*** | |
| S3.2.1 | Whom did (you / she) see? Anyone else?  Kedu onye i/nne ya huru? Ewepu onye a,O nwere onye ozo?  *[Probe, and record all persons seen.]* | 1. Health care provider 2. TBA/Religious healer 3. Relative/neighbor/friend 4. Other *(specify)*   *(_____________________________)*  9. Don’t know | 1. □  2. □  **3. □**  4. □  ***SQ3.3***  9. □ | |
| S3.2.2 | How many times did (you / the mother) receive antenatal care from a health care provider during this pregnancy?  Ugboro ole ka i/nne ya natara enyemuaka ahuike ana enye umu nwanyi ndi di ime site na aka onye oru ahuike oge o di ime a? | | __ __ Times  *(DK = 99)* | |
| S3.2.3 | During which month of the pregnancy did (you / the mother) last receive antenatal care from a health care provider?  Na onwa nke ole na afo ime ka (i/nne ya) natara enyemuaka ahuike a n,enye umunwanyi di ime site n,aka onye oru ahuike? | | __ __ Month  *(DK = 99)* | |
| S3.2.4 | During this pregnancy, did the provider do any of the following for (you / the mother) at least once?  Na oge afo ime a,onye oru ahuike o mere gi/nne ya ihe ndia,o pekata mpe ya buru otu ugboro?  *[Read out all options and check “Yes,” “No” or “Don’t know” for each.]*  *[LOCAL ADAPTATION: Additional high energy and high protein foods to mention If the respondent asks]* | 1. Onye oru ahuike ahu o lelere ma obara (gi / ya) ona agbali elu,agbatu agbatu ka –odi out okwesiri? 2. (I / O) nyere amiri ka elele? 3. (I / O) nyere obara ka elele? 4. Onye oru ahuike ahu ogwara (gi / ya) ribi nri n’enye ume di ga si ka <Akpu na ji> na nri di ga si ka <agwa, akwa, anu> karia ka mgbe (I /O) dighi ime? 5. Onye oru ahu ike ahu o gwara (gi / ya) gbasara ihe ekwesigaghi imee na-afo ime? 6. Onye oru ahu ike ahu o gwara (gi / ya) ebe (I / O) ga aga ma ahuta ihe ndia ekwesigaghi ime? | Yes No DK    1. □ 2. □ 9. □  1. □ 2. □ 9. □  1. □ 2. □ 9. □  1. □ 2. □ 9. □  1. □ 2. □ 9. □  1. □ 2. □ 9. □ | |
| S3.3 | Please tell me the danger signs during pregnancy or labor and delivery that you should seek care for immediately  Biko gwa m ihe ndi na adighi mma,inwe mgbe nwanyi di ime,mgbe ime na eme ya na mgbe o na amu nwa,nke ga eme ka o choo nlezi anya site n,aka onye oru ahuike..  *Probe:* O nwere ndi ozo I ga a gwa kwu m..    *Probe:* Can you tell me any others?  Probe:Inwereike igwam ndi ozo?  *[Check each danger sign mentioned.]* | 1. Obara igba na otu 2. Ihe odido 3. Oke isi onwuwa na anya nkochi 4. Fever and too weak to get out of bed 5. Nnukwu afo mgbu 6. Iku ume osisio m’obu iku ume ehi aru 7. Painful contractions every 20 minutes or less for 12 hours or more 8. Broken water for 12 hours or more 9. Bloody, sticky discharge 12 hrs or more 10. No immediate danger sign mentioned | 1. □  2. □  3. □  4. □  5. □ **___** Mentioned  6. □  7. □  8. □  9. □  10. □ | |
| S3.4 | During this pregnancy, (were you / was the mother) given an injection in the arm to prevent the baby from getting tetanus, that is, convulsions after birth?  Na oge afo ime a a gbara gi/nne ya ogwu n,aka iji gbochie nwa ebu n,afo I nweta nje n,eweta ihe odudo ma a amuputa ya? | 1. Yes 2. No   9. Don’t know | 🞎 ***2 or 9 → SQ3.5*** | |
| S3.4.1 | During this pregnancy, how many times did (you / she) get this injection?  Na oge afo ime,ugboro ole ka agbara gi/nne ya ogwu a? | | __ Times  *(DK = 9)* | |
| S3.5 | At any time before this pregnancy, did (you / the mother) receive any tetanus injection, either to protect yourself or another baby?  Tupu I di ime a I di ugbua agbara (gi/nne ya) ogwu maka nje na eweta ihe odudo iji were gbochie gi ma obu nwa gi ihe odudo? | 1. Yes 2. No   9. Don’t know | 🞎 ***2 or 9 → SQ3.6*** | |
| S3.5.1 | Before this pregnancy, how many other times did (you / she) receive a tetanus injection?  Tupu I di ime a I di ugbu a o dila mgbe a gbara (gi/nne ya) ogwu maka nje na eweta ihe odudo?  *[If 7 or more time, record “7.”]* | | __ Times  *(DK = 9)* | |
| S3.6 | *Skip SQ3.6-3.7.1 in areas wo/malaria.*  During this pregnancy, did (you / the mother) sleep under an insecticide treated bednet?  Na oge afo ime a,o dila mgbe (gi/nne ya) I na ehi ura na akwa a eji egbochi anwu nta nke etinyere ogwu? | 1. Yes, usually or always 2. Yes, sometimes 3. Never   9. Don’t know | 🞎 | |
| S3.7 | During this pregnancy, did (you / the mother) take any drug to prevent (you / her) from getting malaria?  N,oge afo ime a,o dila mgbe (gi/nne ya) nuru ogwu ga egbochi (gi/nne ya) I nweta oria iba? | 1. Yes 2. No   9. Don’t know | 🞎 ***2 or 9 → VQ2.1*** | |
| S3.7.1 | During this pregnancy, how many times did (you / she) take this drug?  N,oge afo ime a,ugboro ole ka (gi/nne ya) nuru ogwu a? | | __ __ Times  *(DK = 99)* | |
| V2.1 | Now I’d like to ask you about any problems (you / the mother) might have had during the pregnancy. Was the late part of the pregnancy (defined as the last 3 months), labor or delivery complicated by any of the following problems that started before the baby was delivered?  Ugbua agam acho iju gi ajuju gbasara ufodu nsogbu (gi/nne ya) nwere oge o di ime.Na ngwucha afo ime(nke a kowari di ka onwa ato ikpeazu n,afo ime) oge ime omume ma o bu oge imu nwa enwere nsogbu ndia tupu a muputa nwa?  *[Read each complication and mark “Yes,” “No” or “Don’t know” for each.]*  *[Read “…the mother…” if the mother is not the respondent.]* | Gi bu nne ya onwere:   1. Ihe odido? 2. Obara mgbali elu? 3. Nnukwu obara iko na-ahu? 4. Oya suga/mamiri? 5. Oke isi owuwa? 6. Anya nkochi?   (Gi / m’obu ya):   1. Oke ike ogwugwu ,isi n’akwa bilie?   Gi bun ne ya onwere:   1. Nnukwu afo mgbu? 2. Iku ume osiso m’obu iku ahi aro ? 3. Ihu oziza? 4. Obara iputa na otu n’udi obula tupu ime emewe? 5. Oke obara ogbugba mgbe ime na eme m’obu mgbe ana amu nwa? 6. Onyi omumua? 7. Ihe sin a otu esisie?   onwere   1. Imu nwa abughi is na isi n’oge mbu? 2. Eriri isi nwa? 3. Eriri isi edi na olu?   Gi bun ne ya onwere   1. Udi nsogbu obula?   *(specify the other complication)* | Yes No DK  1. □ 2. □ 9. □  1. □ 2. □ 9. □  1. □ 2. □ 9. □  1. □ 2. □ 9. □  1. □ 2. □ 9. □  1. □ 2. □ 9. □    1. □ 2. □ 9. □  1. □ 2. □ 9. □  1. □ 2. □ 9. □  1. □ 2. □ 9. □  1. □ 2. □ 9. □    1. □ 2. □ 9. □  1. □ 2. □ 9. □  1. □ 2. □ 9. □  1. □ 2. □ 9. □    1. □ 2. □ 9. □  1. □ 2. □ 9. □  1. □ 2. □ 9. □  *____________________________* | |
| V2.2**^*^** | Did (you / the mother) have any of the following problems that started after the delivery?  i/nne ya e nwere nsogbu ndi a nke bidoro ka o muchara nwa?  *[Read each complication and mark “Yes,” “No” or “Don’t know” for each.]*  *[Read “…the mother…” if the mother is not the respondent.]* | Gi bu nne ya onwere:   1. ihe odido? 2. Oke obara iba? 3. Ahu oku,ihe osi na otu abata m’obu afo egbu? | Yes No DK  1. □ 2. □ 9. □  1. □ 2. □ 9. □  1. □ 2. □ 9. □ | |
| V2.2 | How many months long was the pregnancy?  Onwa ole ka afo ime a di? | | **__ __** Months ***≠ 99 → VQ2.4***  *(DK = 99)* | |
| V2.3 | Did the pregnancy end early, on time, or late?  Afo ime a o kwusiri tupu oge ya eruo ma o bu gafee oge ya? | 1. Early 2. On time 3. Late   9. Don’t know | 🞎 | |
| V2.4 | Was the baby moving in the last few days before the birth?  Na mkpuru ubochi ikpeazu tupu omumu nwa eruo nwa a o emeghariri ahu na afo? | 1. Yes 2. No   9. Don’t know | 🞎 | |
| V2.5 | When did (you / the mother) last feel the baby move?  Kedu mgbe ikpeazu (i/nne ya) mara na nwa a meghariri ahu n,ime afo?  *[Read “…the mother…” if the mother is not the respondent.]*  *[Record hours if less than 24 hours; Record days if 1 day or more.]* | | **__ __** Hours before delivery  *(DK = 99)* | |
|  |  |  | **__ __** Days before delivery  *(DK = 99)* | |
| V2.6 | Did the water break before labor or during labor?  Mmiri akpa nwa o gbapuru tupu ime omume,ka o bu mgbe ime omume bidoro?  *[Note: Labor begins when contractions are no more than 20 minutes apart.]* | 1. Before 2. During   9. Don’t know | 🞎 ***2 or 9 → VQ2.8*** | |
| V2.7 | How much time before labor did the water break?  Oge ole tupu ime omume ebido ka mmiri akpa nwa gbapuru?  *[Record “24” if 1 day or more.]* | | **__ __** Hours  *(DK = 99)* | |
| V2.8 | What was the color of the liquor when the water broke?  Mgbe mmiri akpa nwa a gbapuru kedu ka o si acha? | 1. Green or brown 2. Clear (normal) 3. Other *(specify)*   9. Don’t know | 🞎  ____________________________ | |
| V2.9 | Was the liquor foul smelling?  Mmiri akpa nwa a,o na esi isi ojoo? | 1. Yes 2. No   9. Don’t know | 🞎 | |
| V2.10 | How much time did the labor and delivery take?  Oge ole ka ime omume na imu nwa were?  *[Record “00” if less than 1 hour.]* | | **__ __** Hours  *(DK = 99)* | |
| S3.8 | Where did the delivery occur?  Kedu ebe a muru nwa a? | 1. Hospital 2. Other health provider or facility 3. On route to a health provider or facility 4. Home 5. Other *(specify___________________)*   9. Don’t know | 🞎 1-3 = Health provider  ***9 → SQ3.11*** | |
| S3.9 | Who decided that this was the right place to deliver the baby?  Kedu onye kpebiri na ebe ahu bu ebe kwesiri maka omumu nwa a?  *[Record the one main decision maker.]* | 1. The woman, herself 2. Her husband 3. Her mother 4. Her mother-in-law 5. Her father-in-law 6. Other *(specify)*   9. Don’t know | 🞎  ___________________________ | |
| S3.10 | *If she did not go to a health provider or facility (SQ3.8 = 4-5) for the delivery, ask:* Did (you / the mother) have any concerns or problems that kept (you / her) from going to a health provider or facility for the delivery?  *i/nne ya o nwere nsogbu gbochiri (Gi/ya) I ga na nke onye oru ahuike ma o bu ulo ebe ana amu nwa (SQ3.8 = 4-5)*  *(i/nne ya) o nwere ike gabiga nsogbu nke ga egbochi ya I ga na nke onye oru ahuike ma o bu ulo ebe ana amu nwa?*  *If she went or was on route to a health provider or facility (SQ3.8 = 1-3) for the delivery, ask:* Did (you / the mother) have to overcome any concerns or problems to go to health provider or facility for the delivery?  *If she went or was on route to a health provider or facility (SQ3.8 = 1-3) for the delivery, ask:* I/O gabigara ihe mkpobi ukwu obula ma obu nsogbu di anaa i ga n,ulo ahu ike maka imu nwa a? | 1. Yes 2. No   9. Don’t know | 🞎 ***2 or 9 → SQ3.11*** | |
| S3.10.1 | What concerns or problems did (you / she) have?  Kedu ihe bu nsogbu a (i/o) nwere?  *Prompt:* Was there anything else?  *Prompt:* O nwere ihe ozo?  *[Multiple answers allowed.]* | 1. I cheghi na inwere nnukwu nsogbu ahuike nke ga-eme ka I choo enyemaka onye oru ahuike 2. O nweghi onye ga eso ya aga 3. Oge adighi 4. O bu ihe diri onye ozo I kpebi *(specify)* 5. Uzo tere aka 6. Ugboala adighi 7. Oke ngala onu (ugboala, ahuike, ihe ndi ozo) 8. Im nweghi ntukwasi obi na nlezi anya ahuike nke di 9. Nsogbu gasi(s) ahuike a bu nani ndi ogwu odinala ga-enwe ike gwo ya 10. Chere na nsogbu ahuike ya agaghi ekwe ya eme njem 11. Chere na agbaneghi enyemaka ahuike,na ya m’obu nwa ga anwu 12. O bu n’ime abali (ugboala na onye oru ahuike adighi) 13. Egwu iga na nke onye oru ahuike nwoke n’atu ya 14. Ihe ndi ozo *(specify)*   99.Amagahi | 1. □  2. □  3. □  4. □ _______________________  5. □  6. □  7. □  8. □  9. □  10. □  11. □  12. □  13. □  14. □ ______________________  99. □ | |
| S3.11 | Who (at the facility) delivered the baby?  Onye (na ebe ana-amu nwa) nara gi nwa?  *[Read “...at the facility...” if she delivered at a health facility.]* | 1. Doctor 2. Nurse/midwife 3. Relative/neighbor/friend 4. Self (the mother) 5. Traditional birth attendant 6. Other *(specify)*   9. Don’t know | 🞎  ___________________________ | |
| S3.12 | How soon after labor started did the <BIRTH ATTENDANT> first attend the mother?  Mgbe ime omume bidoro,kedu oge mbu <onye na ana nwa> leziri ya anya?  *[Discuss that labor starts with painful contractions every 20 minutes or less.]*  *[Mark days &/or hours as needed: e.g. 00 day, 06 hours]* | | __ __ Days  *(DK = 99)* | |
|  |  |  | __ __ Hours  *(DK = 99)* | |
| S3.13 | Did the birth attendant use a pictorial graph to follow the progress of (your / the mother’s) labor?  Onye ahu na ana nwa o jiri ihe esere were mara mgbe (i/nne ya) bidoro inwe ime omume? | 1. Yes 2. No   9. Don’t know | 🞎 | |
| S3.14 | Did the birth attendant wash her hands with soap and water or wear surgical gloves before assisting with the birth?  Onye ahu na ana nwa o jiri mmiri na ncha kwo aka ya ma obu yiri glovu tupu o naa nwa? | 1. Yes, washed with soap and water 2. Yes, wore surgical gloves 3. No   9. Don’t know | 🞎 | |
| S3.15 | On what surface did (you / the mother) deliver?  Na elu ebee ka a no naa (gi/nne ya ) nwa? | 1. Labor bed 2. Solid floor with mackintosh/cover 3. Solid washed floor 4. Solid unwashed floor 5. Dirt/soil/mud/straw floor 6. Other *(specify)*   9. Don’t know | 🞎  ___________________________ | |
| V2.17 | Was the delivery...?  A muputara nwa ahu---.?  *[Read the choices and mark ONE.]* | 1. Otu nwanyi na enweghi nko igwe 2. Otu nwanyi nwere nko igwe 3. Otu (Amagahi) 4. C-section   9. Amagahi | 🞎 | |
| V2.18 | During labor but before delivery, did (you / the mother) receive any kind of injection?  Tupu amuputa nwa oge ime na-eme (gi/ya) agbara ya udi ogwu obula?  *[Read “…the mother…” if the mother is not the respondent.]* | 1. Yes 2. No   9. Don’t know | 🞎 | |

| **SA Module 4: Careseeking for maternal complications (FOR STILLBIRTHS AND NN DEATHS < 28 DAYS OLD)**  *Read:* Now, I would like to ask you some questions about (your / the mother’s) careseeking during the pregnancy with <NAME>.  *Read:* Ugbua a chorom m I jug i ufodu ajuju gbasara ka nne (gi/ya) siri choga nlezi anya oge odi ime <Aha>. | | | | | | | | |
| --- | --- | --- | --- | --- | --- | --- | --- | --- |
| S4.1 | **Maternal symptoms:**  *First look back at the maternal VA symptoms in GQ1.9. Mark (“X”) these in the “Symptoms in the last 3 months” column.*  *If she had any symptom(s), then read:* Earlier, you mentioned that (you / the mother) had <SYMPTOM(S)> during the last 3 months of the pregnancy or during labor or delivery. Which of the symptoms started before labor? And which started with or during labor or delivery, including any that may have brought on the labor?  *If she had any symptom(s), then read:*  Na mbido i kwuru na (i/o) nwere ufodu <nsogbu (ga)> na ime onwa ato nke ikpeazu na afo ime,ma o bu n,oge ime omume ma o bu oge I na amu nwa.Kedu nsogbu ndi nke I nwere tupu ime omume?kedu ndi I nwere mgbe ime omumu na mgbe omumu nwa?O nwere nke mere ka ime omume malite?  *[Remind the respondent that labor starts with painful contractions every 20 minutes or less. Then review each reported symptom with her to determine which started before labor and which started* with or during labor *or delivery. Do not include any symptoms here that started after the baby was delivered.]* | | | 1. Ihe odido 2. Obara mgbali elu 3. Nnukwu obara iko na-ahu 4. Oya suga/mamiri 5. Oke isi owuwa 6. Anya nkochi 7. Oke ike ogwugwu isi n’akwa bilie 8. Nnukwu afo mgbu (abughi ime-omume) 9. Ume ichioku m’obu inwe ihe nhiam ahu 10. Ihu iza-aza 11. Obara iputa na otu n’udi obula tupu ime emewe 12. Oke obara ogbugba mgbe ime na-eme m’obu mgbe ana-amu nwa. 13. Onyi omuma 14. Ihe isi I puta na otu 15. Ime omume bidoro (n,oge tup onwa itolu) 16. Akpa mmiri nwa igbawa ihe dika awa isi m’obu karia tupu ime emewe 17. Ime omume ihe dika awa iri n’abuo m’obu karia 18. Ihe ndi ozo *(specify)*   *(____________________________)*   1. Enweghi ihe-ama obula n’ime onwa ito ikpazu 2. Enweghi ihe-ama obula tupu ime omume bidio | | Symptoms during last 3 months  Yes  □  □  □  □  □  □  □  □  □  □  □  □  □  □  □  □  □  □  □ ***→ Inst_8*** | | Started (related to labor/delivery)  Before W/D DK  1. □ 2. □ 9. □  1. □ 2. □ 9. □  1. □ 2. □ 9. □  1. □ 2. □ 9. □  1. □ 2. □ 9. □  1. □ 2. □ 9. □  1. □ 2. □ 9. □  1. □ 2. □ 9. □  1. □ 2. □ 9. □  1. □ 2. □ 9. □  1. □ 2. □ 9. □  1. □ 2. □ 9. □  1. □ 2. □ 9. □  1. □ 2. □ 9. □  1. □ 2. □ 9. □  1. □ 2. □ 9. □  1. □ 2. □ 9. □  1. □ 2. □ 9. □  □ ***→ SQ4.11*** |
| S4.2 | Did (you / the mother) seek care from any person or health facility for (any of) the pregnancy symptom(s) that started before labor?  I / nne ya choro enyemuaka site n,aka onye oru ahuike maka <nsogbu m’obu nsogbu ga afo ime ndi bidoro tupu ime omume?  *[Read “…for any of…” if she had more than one pregnancy symptom.]* | | | 1. Yes 2. No   9. Don’t know | | 🞎 ***2 → SQ4.4***  ***9 → Inst_2*** | | |
| S4.2.1 | Where did (you / she) seek this care?  Kedu ebe (i/o) choro udi enyemuaka a?  *Prompt:* Was there anywhere else?  *Prompt:* O nwere ebe ozo?  *[Multiple answers allowed.]* | | | 1. Ulo ogwu 2. NGO or Ulo ogwu nke goometi 3. Dokoti nke na arul onwe ya/ulo ogwu 4. Noose nke obodo m’obu onye na eji ime 5. TBA/dkoti nke obod/onye ana azuru azu/ndi nke m’obu onye dinaala 6. Relative, neighbor or friend 7. Other *(specify)*   *(*______________________________*)*  99. Don’t know | | 1. □  2. □  3. □ = Health provider  4. □  5. □  ***SQ4.4***  6. □  7. □  9. □ ***→ Inst_2*** | | |
| S4.3 | *If more than one symptom started before labor and she sought care from a health provider (SQ4.2.1 = 1-4), ask:*  For which symptom or symptoms that started before labor did (you / she) seek care from a health provider or facility?  *If more than one symptom started before labor and she sought care from a health provider (SQ4.2.1 = 1-4), ask:*  Kedu nsogbu ndi bidoro tupu ime omume nke (i/o) choro enyemuaka site n,aka onye oru ahuike? | | | 1. Ihe odido □ 2. Obara mgbali elu □ 3. Nnukwu obara iko na-ahu □ 4. Oya suga/mamiri □ 5. Oke isi owuwa □ 6. Anya nkochi □ 7. Oke ike ogwugwu isi n’akwa bilie □ 8. Nnukwu afo mgbul (abughi ime-omume) □ 9. Ume ichioku m’obu inwe ihe nhiam ahu n’iku ume □ | | 1. Ihu iza-aza □ 2. Obara put na otu tupu ime-emewe □ 3. – blank – 4. Onyi omuma □ 5. Ihe isi I puta na otu □ 6. – blank 7. Akpa mmiri igbawa >6 hrs bfr. labor □ 8. – blank – 9. Ihe ndi ozo *(specified in SQ4.1)* □ | | |
| S4.4 | *If she never went to a health provider (SQ4.2 = 2 or SQ4.2.1 ≠ 1-4) for any of the pregnancy symptoms, ask:* Did (you / the mother) have any concerns or problems that kept (you / her) from going to a health provider or facility for the symptom(s) that started before labor?  *If she never went to a health provider (SQ4.2 = 2 or SQ4.2.1 ≠ 1-4) for any of the pregnancy symptoms, ask:*  *(*i/nne ya) e nwere ihe mgbochi mere na (i/o) nweghi ike ga nke onye oru ahuike maka nsogbu afo ime ndi bidoro tupu ime omume?  *If she went to health provider (SQ4.2.1 = 1-4) for any pregnancy symptom(s), ask:* Did (you / the mother) have to overcome any concerns or problems to go to a health provider or facility for the symptom(s) that started before labor?  *If she went to health provider (SQ4.2.1 = 1-4) for any pregnancy symptom(s), ask:* i/nne ya) ogabigara ihe mgbochi obula nke eme ka (i/o)ghara I new ike hu onye oru ahuike gbasara nsogbu afo ime nke bidoro tupu ime omume? | | | 1. Yes 2. No   9. Don’t know | | 🞎 ***2 or 9 → Inst_1*** | | |
| S4.4.1 | What concerns or problems did (you / she) have?  Kedu udi ihe nsogbu ma o bu mgbochi (i/o) nwere?  *Prompt:* Was there anything else?  *Prompt:* O nwere ihe ndi ozo?  *[Multiple answers allowed.]* | | | 1. O cheghi na o na-aria oria nke ruru ya I choga nlezi anya ahu ike 2. Onweghi onye no nso iso ya gaa 3. Ga enwepu otutu oge n’ihe o na eme 4. Onye ozo ga enye ikike *(specify)* 5. O tere aka ije 6. Onweghi ihe ga eburu/kworo madu ga 7. Ego eji akwu ugwo 8. Nlezi anya ana-enye n’ulo ahu ike ejughi ya afo 9. Udi ihe amaa kwesiri ka agwo ya n’uzo odinala 10. O chere na o na-arianukwu oria ekwesighi ibu ga njem 11. O chere na ya na nwa ga anwu agbanyeghi nlezi anya 12. Ujo I jekwuru onye nlezi anya ahu ike nwoke 13. Ihe ndi ozo *(specify)*   99.Amaghi | | 1. □  2. □  3. □  4. □ _______________________  5. □  6. □  7. □  8. □  9. □  10. □  11. □  12. □  13. □ ______________________  99. □ | | |
| ***Inst_1: If SQ4.2 = 2 or SQ4.2.1 ≠ 1-4 (Never went to a health provider for any pregnancy symptoms) →*** ***Inst_2*** | | | | | | | | |
| S4.5 | Did any health provider or facility refer (you / her) to another health provider or facility for (any of) the symptom(s) that started before labor?  O nwere onye oru ahuike zigara(gi/ya) ka I hu onye oru ahuike ozo maka nsogbu m’obu nsogbu ga afo ime ndia ndi nke bidoro tupu ime omume? | | | 1. Yes 2. No   9. Don’t know | | 🞎 ***2 or 9 → SQ4.6*** | | |
| S4.5.1 | Did (you / she) go to the provider or facility to which (you were / she was) referred?  i/o o gara na nke onye oru ahuike nke ahu e zigara (gi/ya)? | | | 1. Yes 2. No   9. Don’t know | | 🞎 | | |
| S4.6 | How many different health providers or facilities did (you / the mother) see for the pregnancy symptom(s) that started before labor?  Ndi oru ahuike di iche iche ole ka (i/nne ya huru maka nsogbu m’obu nsogbu ga afo ime nke bidoro tupu ime omume? | | | | | **__ __** Health providers/facilities  *(DK = 99)* | | |
| S4.7 | (Were you / Was the mother) admitted to hospital for (any of) the symptom(s) that started before labor?  O di oge (i/nne ya) gara n,ulo ogwu rahu arahu maka nsogbu m’obu nsogbu ga afo ime ndi nke bidoro tupu ime omume? | | | 1. Yes 2. No   9. Don’t know | | 🞎 | | |
| S4.8 | Please tell me everything that the provider(s) suggested that (you / the mother) do for the pregnancy symptom(s) at home?  Biko gwam ihe nile onye oru ahuike gwara (gi/nne ya) ka o na eme n,ulo maka nsogbu m’obu nsogbu ga afo ime ndia?  *Prompt:* Was there anything else?  *Prompt:* O nwere ihe ozo?  *[Multiple answers allowed.]* | | | 1. Enyere ogwu mgbochi na onu 2. Enyere ogwu iba na onu 3. Enyere ogwu obara mgbali elu 4. Enyere ogwu ndi ozo na onu 5. Izu ike / izu ike na elu akwa / ebilata oru 6. Ilohachi azu 7. Ilohachi m’obu na oka njo 8. Ihe ndi ozo *(specify)* 9. Onweghi   99. Amaghim | | 1. □  2. □  3. □  4. □  5. □  6. □  7. □  8. □ _______________________  9. □ ***→ Inst_2***  99. □ ***→ Inst_2*** | | |
| S4.9 | Were you / Was the mother) able to follow all this advice?  I / nne ya o nwere ike gbasoo usoro ndumodu ndi a? | | | 1. Yes 2. No   9. Don’t know | | 🞎 ***9 → Inst_2*** | | |
| S4.10 | *If not able to follow all the advice, ask:*  Did (you / she) have any concerns or problems that kept (you / her) from following the advice?  *If not able to follow all the advice, ask:*  (I / O) nwere ihe mgbochi mere na (I / O) nweghi ike I gbaso usoro ndumodu ndi a?  *If able to follow all the advice, ask:*  Did (you / she) have to overcome any concerns or problems to follow the advice?  *If able to follow all the advice, ask:*  i/o o nwere ihe ndi mgbochi I gabigara iji nwee ike gbasoo usoro ndumodu ndi a? | | | 1. Yes 2. No   9. Don’t know | | 🞎 ***2 or 9 → Inst_2*** | | |
| S4.10.1 | What concerns or problems did (you / she) have?  Onwere nsogbu i/o nwere?  *Prompt:* Was there anything else?  *Prompt:* O nwere ihe ozo?  *[Multiple answers allowed.]* | | | 1. A ghotaghi ntuzi aka 2. Otutu oge n’ihe o na-eme 3. Onye ozo cheputara(kwulo onye obu 4. O ga efu nnukwu ego 5. Nsogbua kwesiri ka agwo ya n,uzo odi na-ala 6. Ntuzi aka na nlezi anya a adighi mkpa m’obu baa uru 7. Ntuzi aka nlezi anya a nwere ike meruo nwa ebu n’afo ahu 8. Echere na ya na nwa ebu n’afo ga anwu agbanyeghi nlezi anya 9. Ihe ndi ozo *(kowaputa)*   99.Amaghi | | 1. □  2. □  3. □ _______________________  4. □  5. □  6. □  7. □  8. □  9. □ _______________________  99. □ | | |
| ***Inst_2: Refer to SQ4.1: If no labor or delivery symptoms*** ***→******Inst_8*** | | | | | | | | |
| S4.11 | | Now let’s talk about the labor and delivery symptom(s). You said earlier that the symptom(s) that started with or during labor or delivery (was / were) <SYMPTOM(S)>.  Ugbua ka anyi kwuo maka nsogbu ime omume na omumu nwa.I gwara m na nsogbu ndi a bidoro na ime omume na omumu nwa bu.  *[Read and mark the SQ4.1 symptom(s) confirmed by the respondent. Correct the SQ4.1 responses if necessary.]* | | 1. Ihe odido □ 2. Obara mgbali elu □ 3. Nnukwu obara iko na-ahu □ 4. – blank – 5. Oke isi owuwa □ 6. Anya nkochi □ 7. Oke ike ogwugwu isi n’akwa bilie □ 8. Nnukwu afo mgbu (abughi ime-omume) □ 9. Ume ichioku m’obu imwe ihe nhiam ahu n’iku ume □ | | 1. Ihu iza-aza □ 2. Obara I puta n’udi obula tupu ime-emewe □ 3. Oke obara ogbugba mgbe ime na-eme m’obu mgbe ana amu nwa □ 4. Onyi omuma □ 5. Ihe isi I puta na-ahu nwanyi □ 6. Ime omume bidoro n’oge tupu onwa itolu □ 7. Akpa mmiri nwa igbawa ihe dika awa isi m’obu karia tupu ime emewe □ 8. Ime omume ihe dika awa iri n’abuo m’obu karia □ 9. Ihe ndi ozo *(specified in SQ4.1)* □ | | |
| S4.12 | | Where (were you / was the mother) when (this / the first) symptom began?  Kedu ebe (I no / nne ya no) mgbe (nsogbu a / nke mbu) malitere?  *[Read “…the first…” if she had more than one labor or delivery symptom.]* | | 1. N,ulo 2. N’uzo ka o na-aga ebe onye nlezi anya m’obu ulo ahu ike 3. N’ulo onye nlezi anya m’obu n’ulo ahu ike ebe o jere amum nwa/ime omume 4. Ihe ndi ozo *(specify)*   9. Amaghi | | 🞎 ***3 → SQ4.17***  ___________________________ | | |
| S4.13 | | Did (you / she) receive, seek or try to seek any care or treatment for (any of) the labor or delivery symptom(s)?  (I / O) natara, choga, ma o bu gbambo I cho udi nlezi anya ma o bu ogwugwo (o bula) maka nsogbu ime omume ma obu omumu nwa?  *[Read “…any of the symptoms” if she had more than one symptom.]* | | 1. Yes 2. No   9. Don’t know | | 🞎***2 or 9 → SQ4.17*** | | |
| S4.13.1 | | What was the first thing (you / she) did for the symptom(s)?  Kedu ihe mbu (i/o) mere maka nsogbu (ga) ime omume ma obu omumu nwa a?  *[Mark only the first action taken.]* | | 1. Enyere ya ogwu n’ulo ka obu nwanne ya ka obu onye agbata obi nyere ya   Achoro m’obu agbaliri igwo ya n’aka dokinta ndi obodo:   1. Ulo ogwu 2. NGO or ulo ogwu nke gomeeti 3. Private doctor/clinic 4. Community nurse or midwife 5. Pharmacist or drug seller 6. TBA/dokinta ndi obodo/dokinta amaghi anya ogwu/m’obu ndi ogwu odinala 7. Ihe ndi ozo *(specify)*   99. Amaghi | | 🞎🞎***99 → SQ4.16***  __________________________ | | |
| S4.14 | | Who decided that this was the right thing to do at that time?  Kedu onye kpebiri na ihe a bu ihe kwesiri ka eme na oge ahu?  *[Only one response allowed. Record the main decision maker.]* | | 1. The woman, herself 2. Her husband 3. Her mother 4. Her mother-in-law 5. Her father-in-law 6. Other *(specify)*   9. Don’t know | | 🞎  ___________________________ | | |
| S4.15 | | *If she did not go to a health provider (SQ4.13.1 = 1 or 6-8), ask:* Did (you / the mother) have any concerns or problems that kept (you / her) from going to a health provider at that time?  *If she did not go to a health provider (SQ4.13.1 = 1 or 6-8), ask:*  (I / nne) ya o nwere ihe mgbochi mere na (I / O ) nweghi ike ga na nke onye oru ahuike n,oge ahu?  *If she went to a health provider (SQ4.13.1 = 2-5), ask:* Did (you / the mother) have to overcome any concerns or problems to go to the <HEALTH PROVIDER> at that time?  *If she went to a health provider (SQ4.13.1 = 2-5), ask:*  (i/nne ya) o nwere ihe mgbochiri (i/o) gabigara I jiri nwee ike <hu onye oru ahuike> n,oge ahu? | | 1. Yes 2. No   9. Don’t know | | 🞎 ***2 or 9 → Inst_3*** | | |
| S4.15.1 | | What concerns or problems did (you / she) have?  Kedu ihe mgbochi ndi (i/o) nwere?  *Prompt:* Was there anything else?  *Prompt:* O nwere ndi ozo?  *[Multiple answers allowed.]* | | 1. O cheghi na o na –aria oria nke ruru ya I choga nlezi anya ahuike 2. Onweghi onye no nso iso ya gaa 3. Ga enwepu otutu oge n’ihe o na eme 4. Onye ozo ga-enye ikike 5. O tere aka ije 6. Ugboala adighi 7. Ego eji akwu ugwo 8. Nlezi anya ana enye n’ulo ahu ike ejughi ya afo 9. Udi ihe amaa kwesiri ka-agwo ya n’uzo odinala 10. O chere na o na-aria nukwu oria ekwesighi ibu ga njem 11. O chere na ya na nwa ga anwu agbanyeghi nlezi anya 12. Oo ime abali(ergughi ihe ga eburu madu jee m’obu onye nlezi anya a noghi nso) 13. Ujo I jekwuru onye nlezi anya ahuike nwoke 14. Ihe ndi ozo *(specify)*   99.Amaghi | | 1. □  2. □  3. □  4. □ _______________________  5. □  6. □  7. □  8. □  9. □  10. □  11. □  12. □  13. □  14. □ ______________________  99. □ | | |
| ***Inst_3: If SQ4.13.1 = 2-5 (First went to a health provider or facility)*** ***→******SQ4.16.1*** | | | | | | | | |
| S4.16 | | Did (you / she) ever seek or try to seek care from a health provider or facility for (any of) the labor or delivery symptom(s)?  O dila mgbe (i/o) choro ma o bu gbaa mbo I cho nlezi anya site n,aka onye oru ahuike maka (nsogbu obula) ime omume ma o bu nsogbu (ga) omumu nwa? | | 1. Yes 2. No   9. Don’t know | | 🞎***2 or 9 → SQ4.17*** | | |
| S4.16.1 | | Please tell me all the types of health providers and facilities where (you / she) sought or tried to seek care for (any of) the labor or delivery symptom(s).  Biko gwam ndi oru ahuike nile ebe i/o gara ma o bu ebe I gbara mbo I ga maka nlezi anya gbasara (nsogbu obula) ime omume na nsogbu (ga) omumu nwa?.  *Prompt:* Anywhere else?  *Prompt:* O nwere ebe ozo?  *[Multiple answers allowed.]* | | 1. Hospital 2. NGO or government clinic 3. Private doctor/clinic 4. Community nurse or midwife   9. Don’t know | | 1. □  2. □  3. □  4. □  9. □ | | |
| S4.17 | | *Refer to SQ3.8 to determine the delivery place. Discuss with respondent to confirm or correct the delivery place.*  *Discuss & resolve inconsistencies, for example, if SQ4.13 or 4.16 = “No,” but the mother delivered in a health facility.* | | 1. Hospital 2. Other health provider or facility 3. On route to a health provider or facility 4. Home 5. Other *(specify)*   9. Don’t know | | 🞎 1-3 = Health provider  ___________________________ | | |
| S4.18 | | So, including where (you / the mother) went or tried to go for the labor or delivery symptom(s) and for the delivery, how many health providers or facilities did (you / she) go to?  O buru na etinye ebe (i/ nne ya) gara ma o bu gbara mbo I ga maka nsogbu(ga) nakwa maka omumu nwa,?ndi naeji ime ole m’obu ebe ana amu nwa ole ka (I/O) jere?    *[If SQ4.16 = 2 and SQ4.17 = 4 or 5 → record ‘00’ health providers/facilities]*  *[If SQ4.16 = 2 and SQ4.17 = 1-3 → record ‘01’ health provider/facility]*  *[If SQ4.16 = 2 and SQ4.17 = 9 → record ‘99’ health providers/facilities]*  *[If SQ4.16 = 9 → record ‘99’ health providers/facilities]* | | | | **__ __** Health providers/facilities | | |
| ***Inst_4: If SQ4.12 = 3 (Symptoms began at the health provider where she went for normal labor) → SQ4.22*** | | | | | | | | |
| ***Inst_5: If SQ4.16 = 2 or 9 & SQ4.17 = 4-9 (No health provider seen/sought for the symptoms/delivery) → Inst_8*** | | | | | | | | |
| ***Inst_5.5: If SQ4.1 = only 1 labor or delivery symptom OR If SQ4.16 = 2 or 9*** ***→******SQ4.21*** | | | | | | | | |
| S4.19 | Was there any particular symptom or symptoms for which (you / the mother) went to the (first) health provider?  Onwere ihe ama nke bu ya kpatara (i/Nne nwa) jiri choga onye nlezi anya ahu ike (nke izizi)?  *[Read “…the first health provider?” if she went to more than one provider.]* | | | 1. Yes 2. No   9. Don’t know | | 🞎***2 or 9 → SQ4.21*** | | |
| S4.20 | For which symptom(s) did (you / she) go?  Kedu nsogbu (ga) (I/O) jiri maka ya jee? | | | 1. Ihe odido □ 2. Obara mgbali elu □ 3. Nnukwu obara iko na-ahu □ 4. – blank – 5. Oke isi owuwa □ 6. Anya nkochi □ 7. Oke ike ogwugwu isi n’akwa bilie □ 8. Nnukwu afo mgbu (abughi ime-omume □ 9. Ume ichioku m’obu inwe ihe nhiam ahu n’iku ume □ 10. Ihu iza-aza □ 11. Obara I puta na-ahu nwanyi n’udi obula tupu ime-emewe □ | | 1. Oke obara ogbugba mgbe ime na-eme m’obu mgbe ana-amu nwa □ 2. Onyi omuma □ 3. Ihe isi I puta na-ahu nwanyi □ 4. Ime omume bidoro n’oge tupu onwa itolu □ 5. Akpa mmiri nwa igbawa ihe dika awa isi m’obu karia tupu ime emewe □ 6. Ime omume ihe dika awa iri n’abuo m’obu karia □ 7. Ihe ndi ozo *(specified in SQ4.1)* □ | | |
| S4.21 | How long after the labor or delivery symptom(s) began was it decided to go to the (first) health provider?  Ogologo oge ole gachara nsogbu (ga) ime omume ma obu amum nwaa bidoro ka echeputara I choga onye nlezi anya ahu ike (nke izizi)?  *[Read “…to the first…” if she went or tried to go to more than one health provider.*  *[Mark days, hours &/or minutes as needed: e.g. 00 day, 02 hours, 10 minutes]* | | | | | **__ __** Days  *(DK = 99)* | | |
|  |  |  |  |  |  | **__ __** Hours  *(DK = 99)* | | |
|  |  |  |  |  |  | **__ __** Minutes  *(DK = 99)* | | |
| ***Labor and delivery matrix instructions:*** *Ask the following questions for the first and last health providers where she sought/tried to seek care for the labor and delivery symptoms. If she delivered at a health provider/facility or at home or on route while trying to go to a health provider/facility, then that should be the first health provider (if she went to only one) or the last health provider. Ask all the questions for the first provider before going on to the last.*  *Before asking about the first health provider, read:*  Now I would like to ask about (your / the mother’s) visit to the (first) health provider. *[Read “first” if she went or tried to go to more than one provider.]*  *Before asking about the first health provider, read:*  Ugbua a chorom iju ajuju gbasara (oge izizi) (i/o) chogara onye nlezi anya ahu ike. *[Read “first” if she went or tried to go to more than one provider.]*  *Before asking about the last health provider, read:*  Now I would like to ask about (your / the mother’s) visit to the last health provider.  *Before asking about the last health provider, read:*  Ugbua a chorom iju ajuju gbasara oge ikpazu (i/o) chogara onye nlezi anya ahu ike. | | | | | | | | |
| **– LABOR AND DELIVERY MATRIX QUESTIONS –** | | | | | **FIRST HEALTH PROVIDER** | | **LAST HEALTH PROVIDER** | |
| What was the name of the (first / last) health provider or facility where (you / the mother) (sought care for the labor or delivery symptom(s) / delivered the baby / tried to deliver the baby)?  Kedu aha onye nlezi anya ma obu ulo ahu ike (nke izizi/nke ikpazu) (i/Nne nwa a) chogara nlezi anya na ihi ihe ama ime omume ma obu amum nwa/muo nwaa gbalia imu nwa a?  *Probe to identify the type of provider.* | | | 1. Hospital (Government) 2. Hospital (NGO) 3. Hospital (Private) 4. Health center (Government) 5. Health center (NGO) 6. Health post (Government) 7. Health post (NGO) 8. Private doctor/clinic (Formal) 9. Private doctor/clinic (?Formal?) 10. Trained community nurse/midwife   99. Don’t know | | S4.22  🞎🞎  ___________________  (Name of Provider/Facility) | | S4.32  🞎🞎  ___________________  (Name of Provider/Facility) | |
| After (deciding to seek care / being referred), how much time passed before going to the <FIRST/LAST HEALTH PROVIDER>?  Ka I / O (kpebichara I choga nlezi anya/e zigachara gi choga nlezi anya), ogologo oge ole gachara tupu I / O jee <maka nlezi anya ahu ike nke izizi / nke ikpazu>?  *[Discuss that this might include the time needed to arrange for transportation and money to go to the provider/facility, or to provide home care or go to a traditional provider before going to the health provider.]*  *[If she delivered at home, record the time from decision/referral to delivery.]*  *[Mark days, hours &/or minutes as needed: e.g. 00 days, 02 hours, 10 minutes]* | | | | | S4.23  **__ __** Days  *(DK = 99)* | | S4.33  **__ __** Days  *(DK = 99)* | |
|  |  |  |  |  | **__ __** Hours  *(DK = 99)* | | **__ __** Hours  *(DK = 99)* | |
|  |  |  |  |  | **__ __** Minutes  *(DK = 99)* | | **__ __** Minutes  *(DK = 99)* | |
| Was there any cost to travel to the <FIRST/LAST HEALTH PROVIDER> or pay for (your / the mother’s) care there?  Onwere ugwo (i/o) kwuru iji garuo ebe <nke izizi/nke ikpazu> m,obu maka nlezi anya n,ebe ahu? | | | 1. Yes 2. No   9. Don’t know | | S4.24  🞎 ***2 or 9 → SQ4.25*** | | S4.34  🞎 ***2 or 9 → SQ4.35*** | |
| How did (you / the mother) arrange for the money for these expenses?  Kedu ka (i/Nne nwa a) siri nweta ego e jiri kwuo ugwo ndi ahu?  *[Multiple answers allowed.]* | | | 1. Had available 2. Borrowed 3. Sold assets 4. Help from kin/relatives 5. Community fund 6. Govt. scheme 7. Other   9. Don’t know | | S4.24.1  1. □  2. □  3. □  4. □  5. □  6. □  7. □  9. □ | | S4.34.1  1. □  2. □  3. □  4. □  5. □  6. □  7. □  9. □ | |
| What transportation method was used to go there?  Kedu ka esi aga ebe ahu?  *[Multiple answers allowed.]* | | | 1. Walk 2. Bicycle/rickshaw/cart boat 3. Bus 4. Taxi/auto/trecker 5. Ambulance (auto or motorcycle) 6. Other 7. Could not arrange transport   9. Don’t know | | S4.25  1. □ ***If only walk***  2. □ ***→ SQ4.26.1***  3. □  4. □  5. □  6. □  7. □ ***→ SQ4.26.1***  9. □ | | S4.35  1. □ ***If only walk***  2. □ ***→ SQ4.36.1***  3. □  4. □  5. □  6. □  7. □ ***→ SQ4.36.1***  9. □ | |
| How much did the transportation cost?  Ego ole ka-eji eburu madu ruo ebe ahu? | | | | | S4.26  **__ __ __ __** unit  *(DK = 9999)* | | S4.36  **__ __ __ __** unit  *(DK = 9999)* | |
| Did (you / the mother) reach the <FIRST/LAST HEALTH PROVIDER> before delivering the baby?  (i/o) ruru ebe nlezi anya ahu ike< nke izizi/nke ikpazu> tupu a muo nwa ahu?  *If “No,” discuss with respondent to reach correct response: 2, 3 or 4.]* | | | 1. Ee ogaruru tupu amu nwata 2. Mba, o muru ya tupu ogaba 3. Mba, o muru n’oge ona ag na ulo ahuike 4. Mba, Onweghi ike eru n ulo ogwu – o omlatelit njem/lonta na ulo/eme ihe ndi ozo   9. Amaghi | | S4.26.1  🞎 ***2, 3 → Inst_8***  ***4, 9 → Inst_7*** | | S4.36.1  🞎 ***2-9 → Inst_8*** | |
| How long did it take to travel to the <FIRST/LAST HEALTH PROVIDER>?  Ogologo oge ole ka igaruo ebe nlezi anya ahu ike< nke izizi/nke ikpazu> were?  *[Mark hours &/or minutes as needed: e.g. 05 hours, 30 minutes]* | | | | | S4.27  **__ __** Hours  *(DK = 99)* | | S4.37  **__ __** Hours  *(DK = 99)* | |
|  |  |  |  |  | **__ __** Minutes  *(DK = 99)* | | **__ __** Minutes  *(DK = 99)* | |
| What did the <FIRST/LAST HEALTH PROVIDER> do for (your / the mother’s) (labor or delivery symptom(s) / delivery)?  Kedu ihe onye nlezi anya ahu ike <nke izizi/nke ikpazu a> mere banyere nsogbu (ga) ime omume m,obu amum nwa/imu nwa?  *Prompt:* Was there anything else?  *Prompt:* Onwere ihe ozo?  *[Multiple answers allowed.]* | | | 1. Enyere nwata ihe na enyere iku ume aka 2. Enyere ogwu ga mgbochi na onu 3. Enyere ogwu iba na onu 4. Enyere ogwu obara mgbali elu na onu 5. Ogwu ndi ozo na onu 6. Enyere ogwu ekwusi obara igbaa 7. Enyere ogwu ekwusi ihe odido 8. Enyere ogwu Gave medicine to ewelite ike 9. Enyere ogwu ekwusi ime omumue 10. Enyere ogwu maka umogi obi 11. Enyere Ogwu IM 12. Enyere ogwu mmiri ihem’obu ogwu 13. Enyere ya obara 14. A tunyere alo igote na ebe ozo 15. Uterine massage 16. Mere a C-section 17. Awara afo oz *(specify)* 18. Anbatara n’ulo ogwu 19. Ihe ozo *(specify)* 20. Onweghi   99.Amaghim | | S4.28  1. □  2. □  3. □  4. □  5. □ _______________  6. □  7. □  8. □  9. □  10. □  11. □  12. □  13. □  14. □  15. □  16. □  17. □ ______________  18. □ stayed __ __ days  19. □ ______________  20. □ ***→ SQ4.30***  99. □ ***→ SQ4.30*** | | S4.38  1. □  2. □  3. □  4. □  5. □_______________  6. □  7. □  8. □  9. □  10. □  11. □  12. □  13. □  14. □  15. □  16. □  17. □ ______________  18. □ stayed __ __ days  19. □ ______________  20. □ ***→ SQ4.40***  99. □ ***→ SQ4.40*** | |
| How much did (you / the mother) pay for these treatments and other costs related to the health care, including any admission fee, consultation, lab tests, equipment, and room and food for companions?  Ego ole ka (i/Nne nwa) kwuru maka ogwu ndia nakwa ihe ndi ozo gbasara nlezi anya ahu ike tinyekwara ego eji anabata n,ulo ahu ike,ihu dibia bekee,nlele ahu,akara ngwa nile,ulo,nri nakwa nke ndi nlekota? | | | | | S4.29  **__ __ __ __ __** unit  *(DK = 99999)* | | S4.39  **__ __ __ __ __** unit  *(DK = 99999)* | |
| Did the <FIRST/LAST HEALTH PROVIDER> refer (you / the mother) to another health provider or facility?  Ulo ahu ike< nke izizi/nke ikpazu> zipuru(gi/Nne nwa) ga hu onye nlezi anya m,obu ulo ahu ike ozo? | | | 1. Yes 2. No   9. Don’t know | | S4.30  🞎 ***2 or 9 → SQ4.30.2*** | | 4.40  🞎 ***2 or 9 → SQ4.40.2*** | |
| Why (were you / was the mother) referred?  Kedu ihe kpatara eji zipu (gi/Nne nwa a) ga hu onye nlezi anya m,obu ulo ahu ike ozo?  *[Multiple answers allowed.]* | | | 1. Onye oru ahuike enweghi ike ihazi nsogbu ahu ya 2. Ihe ndi achoro dika ogwu ikuku)adighi 3. Akara ngwa achoro dika igwe eji ele ahu adighi 4. Ihe achoro (dika ulo ebe iga awa afo) adighi   9. Amaghim | | S4.30.1  1. □  2. □  3. □  4. □  9. □ | | S4.40.1  1. □  2. □  3. □  4. □  9. □ | |
| Was the baby delivered at the <FIRST/LAST HEALTH PROVIDER>?  A muru nwa ahu n’ebe nlezi anya ahu ike< nke izizi m’obu na nke ikpazu>? | | | 1. Yes 2. No   9. Don’t know | | S4.30.2  🞎 ***1 → Inst_8*** | | S4.40.2  🞎 ***1 → Inst_8*** | |
| ***Inst_6: Check SQ4.18 to determine if she went to another health provider*** | | | | | | |  | |
| *If did not go to another health provider, ask:* Did (you / the mother) have any concerns or problems that kept (you / her) from going to another provider?  *If did not go to another health provider, ask:* Onwere nsogbu m,obu ihe nhia ahu(i/nne nwa) nwere nke gbochiri (gi/ya) iga ebe nlezi anya ahu ike ozo ?  *If went to another health provider, ask:* Did (you / the mother) have to overcome any concerns or problems to go to another provider?  *If went to another health provider, ask:* onwere nsogbu m,obu ihe nhia ahu obula(i/Nne nwa ) nwere n,ihi I ga ebe nlezi anya ahu ike ozo? | | | 1. Yes 2. No   9. Don’t know | | S4.31  🞎***2 or 9 → Inst_7*** | | S4.41  🞎***2 or 9 → Inst_8*** | |
| What concerns or problems did (you / she) have?  Kdeu nsogbu m’ obu ihe nhia ahu ( I / O) nwere?  *Prompt:* Was there anything else?  *Prompt:* Onwere ozo?  *[Multiple answers allowed.]* | | | 1. Oturu alo na achoghi enyemaka 2. Onweghi onye nonso iso nwada jee 3. Onwere ezigbo oge iji ruo oru ya 4. Onye ozo mere nkowa 5. Njem tere aka 6. Ugboala njem adighi 7. Ego (ugboala,uloahuike na ihe ndi ozo) 8. Anyi enweru afor ojuju na nlekota enyere anyi 9. Nsogbua kwesiri ihe odinala 10. Echerem na ahu adighi mma aga ekweya mee njem 11. O chere na nwata ga anwu na agbanye nlete anya 12. Obu na etiti abali 13. O mugo tupu ogawa 14. Ndi ozo*(specify)*   99. Amaghi | | S4.31.1  1. □  2. □  3. □  4. □ ______________  5. □  6. □  7. □  8. □  9. □  10. □  11. □  12. □  13. □ ***→ Inst_8***  14. □ ______________  99. □ | | S4.41.1  1. □  2. □  3. □  4. □ ______________  5. □  6. □  7. □  8. □  9. □  10. □  11. □  12. □  13. □  14. □ ______________  99. □ | |
| ***Inst_7: Check SQ4.18 → If she went to another health provider*** | | | | | ***…go to SQ4.32 (LAST HEALTH PROVIDER)*** | |  | |
| ***Inst_8: STOP – If VQ1.15 = 1 (Stillbirth) → VQ5.4 (Section 5: Health records)*** | | | | | | | | |

| **SA Module 5a: Care of the newborn; and VA Section 3: Neonatal deaths (FOR NN DEATHS <28 DAYS OLD)**  *Read:* Now I would like to ask you about the care of the newborn child.  *Read:* Ugbua achorom iju gi ajuju gbasara nlezi anya nwa ohuru ahu. | | | | | | | |
| --- | --- | --- | --- | --- | --- | --- | --- |
| S5a.1 | What tool was used for cutting the cord?  Kedu ihe ejiri bee eriri nwa? | 1. New/from delivery kit/boiled razor blade 2. Old razor blade 3. Scissors 4. Other *(specify)*   9. Don’t know | 🞎  ___________________________ | | | | |
| S5a.2 | What material was used for tying the cord?  Kedu ihe ejiri kee isi eriri nwa? | 1. Clean/from delivery kit/boiled piece of thread 2. Unclean piece of thread 3. Cord clamp 4. Other *(specify)*   9. Don’t know | 🞎  ___________________________ | | | | |
| S5a.3 | Was anything applied to the umbilical cord stump after birth?  Onwere ihe etere n,isi eriri ihe na eso nwa ka-amuchara nwa? | 1. Yes 2. No   9. Don’t know | 🞎 ***2 or 9 → VQ3.1*** | | | | |
| S5a.3.1 | What was it?  Kedu ihe obu? | 1. Nmanya na aba na anyal/ma na ebughari ahu 2. Ogwu omimi/ude eji agwo nmgbu na/ntu otite 3. Nmanu ogwugwo oria,ude mkpuru okwikwe 4. Nsi anumanu,ihe ruru iyi,na ntu 5. Ihe ndi ozo *(specify)*   9. Amgahi | 🞎  ___________________________ | | | | |
| V3.1 | Were there any bruises or signs of injury on the baby’s body at birth?  Enwegasiri onya ma obu ihe dika ihe meru ahu ndi ozo na-ahu nwa oge amuputara ya? | 1. Yes 2. No   9. Don’t know | 🞎 | | | | |
| V3.2 | Was any part of the baby physically abnormal at the time of delivery? (for example: body part too large or too small, additional growth on body)  Onwere akuku ahu nwa ahu obula nke adighi ka okwesiri oge ahu amuputara ya?ihe dika:akuku ahu ibu oke ibu ma obukwanu pee nukwu mpe,ihe toputa na ahu) | 1. Yes 2. No   9. Don’t know | 🞎 ***2 or 9 → VQ3.4*** | | | | |
| V3.3 | What were the abnormalities?  Kedu ihe bu ihe ndi ahu adighi ka okwesiri?  *Ask for the following abnormalities:*  *[Mark all that apply – Show photos]* | 1. Ogbe isi ya opere nnukwu mpe oge amuru ya? 2. Ogbe isi ya oburu nnukwu ibu oge amuru ya? 3. Enwere nnukwu nsogbu na azu isi ya,m’obu na etiti ogbe azu ya? 4. Enwere ulosi ozo na eme ya?   *(If “Yes,” then specify)* | Yes No  1. □ 2. □    1. □ 2. □  1. □ 2. □  1. □ 2. □  ____________________________ | | | | |
| V3.4 | Did the baby breathe immediately after birth?  Nwa ahu o kuru ume ozugbo amuchara ya? | 1. Yes 2. No   9. Don’t know | 🞎 ***2 → VQ3.6*** | | | | |
| V3.5 | Did the baby have difficulty breathing?  Iku ume ohiara nwa ahu ahu? | 1. Yes 2. No   9. Don’t know | 🞎 | | | | |
| V3.6 | Was anything done to try to help the baby breathe at birth?  Onwere ihe emere iji nyere nwa ahu aka iku ume mgbe amuputara ya? | 1. Yes 2. No   9. Don’t know | 🞎 | | | | |
| V3.7 | Did the baby cry immediately after birth?  Nwa ahu obere akwa ozugbo amuputara ya? | 1. Yes 2. No   9. Don’t know | 🞎 ***1 → VQ3.9*** | | | | |
| V3.8 | How long after birth did the baby first cry?  Ogologo oge ole gachara amuputara nwa a tupu obee akwa?  *[Mark ONE response]* | 1. Within 5 minutes 2. Within 6-30 minutes 3. More than 30 minutes 4. Never   9. Don’t know | 🞎 ***4 → SQ5a.4*** | | | | |
| V3.9 | Did the baby stop being able to cry?  Nwa ahu okwusiri ibenwu akwa? | 1. Yes 2. No   9. Don’t know | 🞎 ***2 or 9 → SQ5a.4*** | | | | |
| V3.10 | How long before the baby died did the baby stop crying?  Ogologo oge ole gachara nwa ahu kwusiri ibenwu akwa ka-onwuru? | 1. Less than one day 2. One day or more   9. Don’t know | 🞎 | | | | |
| S5a.4 | How long after birth was the baby first bathed?  Ogologo oge ole gachara amuputara nwa ahu tupu asaa ya ahu? | 1. Less than 1 hour 2. 1-23 hours 3. 24-72 hours (1-3 days) 4. More than 72 hours (3 days) 5. Not bathed   9. Don’t know | 🞎 | | | | |
| S5a.5 | Was anything done to keep the baby warm on the first day after birth?  Onwere ihe emere,ka ahu nwa ahu di oku ubochi izizi ahu amuchara ya? | 1. Yes 2. No   9. Don’t know | 🞎 ***2 or 9 → SQ5a.6*** | | | | |
| S5a.5.1 | What was done?  Kedu ihe emere?  *[Multiple answers allowed.]*  *For each mentioned, ask:*  Ogologo oge ole gara amuputara nwa tupu eme nke a? | 1. Koro ako na ighichapu 2. Nkechikota na akwa oyi 3. Nchikota ahut 4. Igwe oku eji echekwa nwa 5. Ihe ndi ozo   *(specify other)* | Done  1. □  2. □  3. □  4. □  5. □ | | How soon after birth  <1hr <6 6-24 >24 DK  1.□ 2.□ 3.□ 4.□ 9.□ 1.□ 2.□ 3.□ 4.□ 8.□  1.□ 2.□ 3.□ 4.□ 9.□  1.□ 2.□ 3.□ 4.□ 9.□  1.□ 2.□ 3.□ 4.□ 9.□ | | |
|  |  |  | ____________________________ | | | | |
| S5a.6 | Did (you / the mother) or a wet nurse ever breastfeed the baby?  (I/Nne nwaa) enyere ma obu kwanu nwanyi ozo miri ara ya na-agba onyere ya ara? | 1. Yes 2. No   9. Don’t know | 🞎***2 or 9 → SQ5a.7*** | | | | |
| S5a.6.1 | How long after birth was the baby first put to the breast?  Ogologo oge ole amuputachara nwa ahu ka etinyere ya onu na ara?  *[If immediately or less than 1 hour, record ’00’ hours.]*  *[If less than 24 hours, record hours; otherwise record days.]* | | __ __ Days  *(DK = 99)*  OR | | | | |
|  |  |  | __ __ Hours  *(DK = 99)* | | | | |
| S5a.6.2 | Was the baby being breastfed at the time when the fatal illness began?  A na enyekwa nwa ahu ara n,ime oge a oyia gburu ya bidoro? | 1. Yes 2. No   9. Don’t know | 🞎 | | | | |
| S5a.7 | At the time the fatal illness began, was the baby being given any other liquid, including non-human milk or formula, fruit juice, tea or water, or any semisolid or soft foods such as cereal?  Na ime oge oria gburu nwa ahu bidoro,o nwere ihe mmiri ozo ana enye ya dika mmiri ara ozo abughi nke madu m,obu ihe onunu obula,mmiri mkpuro osisi,tea m,obu mmiri nkiti,nri ndi ozo esichaghi ike dika akamu m,obu ihe yiri ya?  *[Multiple answers allowed. Probe, and record all liquids and foods given.]* | 1. Enweghi nmiri ara ma obu nke ara ehi 2. Ara ehi etinyere nke nmiri 3. Nmanya nmiri uto,nmiri nkiti na obunmanya etinyere nmiri 4. Ogwu ntunye na onu 5. Mkpuru nmiri ma obu ogwu nmiri 6. Nri situru ike m’obu nri di nro 7. Enwesighi ihe ozo,enwepu nmiri ara   9. Don’t know | 1. □  2. □  3. □  4. □  5. □  6. □  7. □  9. □ | | | | |
| V3.11 | Was the baby able to suckle in a normal way during the first day of life?  Nwa ahu onwere ike mia ara,otu  Okwesiri ubochi izizi ahu amuru ya? | 1. Yes 2. No   9. Don’t know | 🞎 ***1 → VQ3.13*** | | | | |
| V3.12 | Did the baby ever suckle in a normal way?  Onwere oge nwa ahu miri ara otu okwesiri? | 1. Yes 2. No   9. Don’t know | 🞎 ***2 or 9 → VQ3.17*** | | | | |
| V3.13 | Did the baby stop being able to suckle in a normal way?  Nwa ahu okwusiri I minwu ara otu okwesiri? | 1. Yes 2. No   9. Don’t know | 🞎 ***2 or 9 → VQ3.17*** | | | | |
| V3.14 | How long after birth did the baby stop suckling?  Ogologo oge ole amuchara nwa ahu ka-okwusiri iminwu ara?  *[Less than 24 hours = “00” days]* | | **__ __** Days  *(DK = 99)* | | | | |
| V3.15 | How long before s/he died did the baby stop suckling?  Ogologo oge ole tupu O nwuo ka O kwusiri imi ara? | 1. Less than one day 2. One day or more   9. Don’t know | 🞎 | | | | |
| V3.16 | Was the baby able to open her/his mouth at the time s/he stopped suckling?  Nwa ahu O na emeghnwu onu ya oge ahu O kwusiri inu ara? | 1. Yes 2. No   9. Don’t know | 🞎 | | | | |
| V3.17 | During the illness that led to death, did the baby have difficult breathing?  Mgbe ahu oria gburu nwa ahu na-aria ya,iku ume o na ahia ya ahu? | 1. Yes 2. No   9. Don’t know | 🞎 ***2 or 9 → VQ3.20*** | | | | |
| V3.18 | At what age did the difficult breathing start?  Afo ole ka o di oge o bidoro nwebe ihia ahu iku ume?  *[Less than 24 hours = “00” days]* | | **__ __** Days  *(DK = 99)* | | | | |
| V3.19 | For how many days did the difficult breathing last?  Mkpuru ubochi ole ka o nwere iku ume ihia ahu a?  *[Less than 24 hours = “00” days]* | | **__ __** Days  *(DK = 99)* | | | | |
| V3.20 | During the illness that led to death, did the baby have fast breathing?  Oge o na aria oria gburu ya,o nwere ume ichi-oku? | 1. Yes 2. No   9. Don’t know | 🞎 ***2 or 9 → VQ3.23*** | | | | |
| V3.21 | At what age did the fast breathing start?  Afo ole k,odi oge ume ichi-oku ahu bidoro?  *[Less than 24 hours = “00” days]* | | **__ __** Days  *(DK = 99)* | | | | |
| V3.22 | For how many days did the fast breathing last?  Mkpuru ubochi ole ka onwere ume ichi oku ahu?  *[Less than 24 hours = “00” days]* | | **__ __** Days  *(DK = 99)* | | | | |
| V3.23 | During the illness that led to death, did the baby have indrawing of the chest?  Oge o na aria oria a gburu ya,obi nwa ahu o na emikpu n,ime?  *[Show photo]* | 1. Yes 2. No   9. Don’t know | 🞎 | | | | |
| V3.24 | During the illness that led to death, did the baby have grunting?  Oge o na aria oria a gburu ya ututu o na etu ya?  *[Demonstrate grunting]* | 1. Yes 2. No   9. Don’t know | 🞎 | | | | |
| V3.25 | During the illness that led to death, did the baby have spasms or convulsions?  Oge o na aria oria a gburu ya ,ihe odido o na adowa ya? | 1. Yes 2. No   9. Don’t know | 🞎 | | | | |
| V3.26 | During the illness that led to death, did the baby have fever?  Oge o na aria oria a gburu ya,o ahu oku orere ya? | 1. Yes 2. No   9. Don’t know | 🞎 ***2 or 9 → VQ3.29*** | | | | |
| V3.27 | At what age did the fever start?  Afo ole k’odi oge ahu ire oku ahu bidoro?  *[Less than 24 hours = “00” days]* | | **__ __** Days  *(DK = 99)* | | | | |
| V3.28 | How many days did the fever last?  Mkpuru ubochi ole ka ahu ire oku ahu rere ya?  *[Less than 24 hours = “00” days]* | | **__ __** Days  *(DK = 99)* | | | | |
| V3.29 | During the illness that led to death, did the baby become cold to touch?  Oge a o n’aria oria gburu ya,ahu nwaa o na-aju oyi m,emutu ya aka? | 1. Yes 2. No   9. Don’t know | 🞎 ***2 or 9 → VQ3.32*** | | | | |
| V3.30 | At what age did the baby start feeling cold to touch?  Afo ole ka nwa ahu di oge o bidoro juwa oyi na emetu ya aka?  *[Less than 24 hours = “00” days]* | | **__ __** Days  *(DK = 99)* | | | | |
| V3.31 | How many days did the baby feel cold to touch?  Mkpuru ubochi ole ka o na aju oyi imetu aka?  *[Less than 24 hours = “00” days]* | | **__ __** Days  *(DK = 99)* | | | | |
| V3.32 | During the illness that led to death, did the baby become lethargic, after a period of normal activity?  Mgbe nwata a n’aria ahu mere ka o nwuo,o nwere oke ike ogwugwu na ime nwayo oge o kwusiri egwu? | 1. Yes 2. No   9. Don’t know | 🞎 | | | | |
| V3.33 | During the illness that led to death, did the baby become unresponsive or unconscious?  Oge a o na aria oria gburu ya,onwere mgbe omaghizi ma madu o na emetu ya-aka m,obu omaghizi onwe ya? | 1. Yes 2. No   9. Don’t know | 🞎 | | | | |
| V3.34 | During the illness that led to death, did the baby have a bulging fontanelle?  Mgbe nwata a n’aria ahu mere ka onwuo,opi isi o toputara karia otu o kwesiri?  *[Show photo]* | 1. Yes 2. No   9. Don’t know | 🞎 | | | | |
| V3.35 | During the illness that led to death, did the baby have pus drainage from the umbilical cord stump?  Oge a o n’aria oriaa gburu ya ihe dika abu osi na isi otubo ya puta? | 1. Yes 2. No   9. Don’t know | 🞎 | | | | |
| V3.36 | During the illness that led to death, did the baby have redness of the umbilical cord stump?  Ogea o n’aria oria gburu ya,isi otubo ya o chara obara obara? | 1. Yes 2. No   9. Don’t know | 🞎 ***2 or 9 → VQ3.38*** | | | | |
| V3.37 | Did the redness of the umbilical cord stump extend onto the abdominal skin?  I cha obara obara ahu ofere ruo n’ahu afo ya? | 1. Yes 2. No   9. Don’t know | 🞎 | | | | |
| V3.38 | During the illness that led to death, did the baby have skin bumps containing pus or a single large area with pus?  Ogea o n’aria oriaa gburu ya etuto abu di n’ime ya otukasiri ya n’ahu m’obu na-otu akuku ahu ya? | 1. Yes 2. No   9. Don’t know | 🞎 | | | | |
| V3.39 | During the illness that led to death, did the baby have ulcer(s) (pits)?  Ogea O n’aria oriaa gburu ya,nwa ahu onwere onyia(gasi) m’obu (etutogasi)? | 1. Yes 2. No   9. Don’t know | 🞎 | | | | |
| V3.40 | During the illness that led to death, did the baby have an area(s) of skin with redness and swelling?  Ogea o n’aria oria gburu ya,O nwere akuku(gasi) ahu nwa ahu ebe n‘acha obara obara ma za aza? | 1. Yes 2. No   9. Don’t know | 🞎 | | | | |
| V3.41 | During the illness that led to death, did s/he have areas of the skin that turned black?  Ogea O n’aria oriaa gburu ya,akuku ahu ya ufodu otughariri jiwe oji? | 1. Yes 2. No   9. Don’t know | 🞎 | | | | |
| V3.42 | During the illness that led to death, did the baby bleed from anywhere?  Ogea O n’aria oriaa gburu ya,obara o si n,akuku ahu ya obula aputa? | 1. Yes 2. No   9. Don’t know | 🞎 ***2 or 9 → VQ3.44*** | | | | |
| V3.43 | Record from where did the baby bleed  Deputa akuku ahu ya ebe obara si aputa: |  | | | | | |
| V3.44 | During the illness that led to death, did s/he have more frequent loose or liquid stools than usual?  Ogea O n’aria oriaa gburu ya,onyuru nsi esighi ike m’obu nke di mmiri mmiri karia otu okwesiri? | 1. Yes 2. No   9. Don’t know | 🞎 ***2 or 9 → VQ3.46*** | | | | |
| V3.45 | How many stools did the baby have on the day that diarrhea/loose liquid stools were most frequent?  Ugboro ole ka nwa jere mposi ubochi ahu oka nyuo afo osisa/nsi mmiri mmiri ahu? | | **__ __** Stools  *(DK = 99)* | | | | |
| V3.46 | During the illness that led to death, did s/he vomit everything?  Ogea o n’aria oriaa gburu ya,o gboputara ihe obula? | 1. Yes 2. No   9. Don’t know | 🞎 | | | | |
| V3.47 | During the illness that led to death, did s/he have yellow skin?  Ogea O n’aria oria gburu ya,akpukpo ahu ya ochara edo-edo? | 1. Yes 2. No   9. Don’t know | 🞎 | | | | |
| V3.48 | During the illness that led to death, did the baby have yellow eyes?  Ogea O n’aria oria gburu ya,mkpuru anya nwa ahu ochara edo-edo? | 1. Yes 2. No   9. Don’t know | 🞎 | | | | |
| V3.49 | Did the infant appear to be healthy and then just die suddenly?  Nwantakiri ahu odicha ka onye ahu di mma onokata nwuo otu ahu? | 1. Yes 2. No   9. Don’t know | 🞎 | | | | |
| S5a.8 | *Check SQ4.17 to determine if the baby was born in a health facility (codes 1-2):* | 1. Yes, born in a health facility 2. Not born in a health facility   9. Don’t know | 🞎***2 or 9 → SQ5a.10*** | | | | |
| S5a.8.1 | Did the baby leave the delivery facility alive or did s/he die in the facility?  Nwa ahu odi ndu ekuruya laa ka onwuru na ulo ahu ike ahu? | 1. Yes, left alive 2. Died in the facility   9. Don’t know | 🞎***2 or 9 → SQ6.1*** | | | | |
| S5a.8.2 | How soon after birth did the baby leave the facility?  Ogologo oge ole amuchara nwa ahu ka ekuru ya laa n’ulo ahu ike ahu?  *[Record hours if less than 24 hours—if less than 1 hour, record ‘00’ hours; Record days if 1 day or more.]* | | **__ __** Days  *(DK = 99)*  OR | | | | |
|  |  |  | **__ __** Hours  *(DK = 99)* | | | | |
| S5a.8.3 | Was the child examined by a health worker prior to discharge?  Onye oru ahu ike olere nwa ahu tupu asi ha laa na ulo ahu ike ahu? | 1. Yes 2. No   9. Don’t know | 🞎 | | | | |
| S5a.9 | What (were you / was she) counselled on?  Kedugasi ndumodu e nyere (gi/Nne nwa a)? | 1. Yes 2. No   9. Don’t know | 🞎***2 or 9 → SQ5a.10*** | | | | |
| S5a.9.1 | What (were you / was she) counselled on?  Kedu ihe agwara (gi/ya) mee na ndumodu ahu?  *[Multiple answers allowed].*  *Probe:* Anything else?  *Probe:* Onwere ihe ozo? | 1. Inye ara 2. Ogwu mgbochi 3. I bia nlekota onye muchara nwa 4. Nziputa oria ogbara ofu 5. Ihe ndi ozo (specify)   9. Amagahi | 1. □  2. □  3. □  4. □  5. □ ________________________  9. □ | | | | |
| S5a.10 | Was the baby ever seen by a health worker or nurse at home or in the community, or by a doctor or nurse at a health facility before the fatal illness began?  Onwere onye oru ahu ike obula ma obu dibia bekee huru nwa ahu,n’ulo n’ime obodo m’obukwanu n,ulo ahu ike obula tupu oria gburu ya bido?  *[Multiple answers allowed.]*  *For each mentioned, ask:*  How many times was the baby seen by a <PROVIDER TYPE at PLACE> before the fatal illness began?  *For each mentioned, ask:*  Ugboro ole ka a huru nwa < na -ulo ahu ike, na udi ebee >,tupu oria bido?  *Then ask:*  When was the baby first seen by (this / any of these) provider(s)?  *Then ask:*  Kedu oge izizi ( onyea / otu n’ime onye olu) ahuike (ga) huru ya? | 1. CHW or nurse at home/in community 2. Doctor or nurse at a health facility 3. Never seen   9. Don’t know | Seen  1. □  2. □  3. □  9. □ | Times  __ __  __ __ | | | First visit  __ __  Days old  *(<1 = 00;*  *DK = 99)* |
| S5a.11 | Before the fatal illness began, did <NAME> suffer from any of the following known conditions:  Tupu oria gburu ya bido <AHA> oriara udi oria ndia :  *[Read out all conditions and check “Yes,” “No” or “Don’t know” for each.]*  *If “Yes,” then ask:* Onwere ihe enyere ya iji gwuo onodu ahu? | 1. Nwa erughieru amu ya    1. Enyekwara ya nri di iche?    2. Ka enyere ya nlekota enweghi isi”? 2. Ezughi ezu biara mgbe ana amu ya:    1. Isi,olu,na ma obu azu    2. Onu,agba    3. Obi    4. Aka na Ukwu 3. Ihe ndi ozo -------------------------------------   *(specify other)* | Suffered from  Yes No DK  1. □ 2. □ 9. □      1. □ 2. □ 9. □  1. □ 2. □ 9. □  1. □ 2. □ 9. □  1. □ 2. □ 9. □  1. □ 2. □ 9. □ | | | Treatment  Yes No DK  1. □ 2. □ 9. □  1. □ 2. □ 9. □  1. □ 2. □ 9. □  1. □ 2. □ 9. □  1. □ 2. □ 9. □  1. □ 2. □ 9. □  1. □ 2. □ 9. □ | |
|  |  |  | _____________________________ | | | | |
| ***Inst_1: STOP – If VQ1.26 = 1 (Neonatal death) →*** ***SQ6.1*** | | | | | | | |

| **SA Module 5b: Preventive care of post-neonates (FOR CHILD DEATHS 28 DAYS—59 MONTHS OLD)**  *Read:* Now I would like to ask you about the care of the child before the fatal illness began.  *Read:* Ugbua a chorom iju gi maka nlezi anya enyere nwa ahu tupu oria gburu ya bido. | | | | | | | | |
| --- | --- | --- | --- | --- | --- | --- | --- | --- |
| S5b.1 | | | Where (do you / does the mother) cook?  Kedu ebe (i/Nne nwa a) na esi nri? | - 1. Inside the house   2. Outside the house   3. In a structure outside the house   9. Don’t know | | 🞎 | | |
| S5b.2 | | | When (you / the mother) cooked, was <NAME> usually beside or carried by (you / her)?  Onwere mgbe (I/Nne nwa) o na -eku (AHA) na akuku ka o na –ano na akuku (gi/ya) mgbe ana esi nri? | 1. Yes 2. No   9. Don’t know | | 🞎 | | |
| S5b.3 | | | *Skip SQ5b.3 in areas wo/malaria.*  Before (her / his) fatal illness began, did <NAME> sleep under an insecticide treated bednet?  Tupu oria gburu (ya) e bido,<AHA> O na –arahu n,ime akwa etere ogwu ichu anwunta ? | 1. Yes, usually or always 2. Yes, sometimes 3. Never   9. Don’t know | | 🞎 | | |
| S5b.4 | | | Did (you / the mother) or a wet nurse ever breastfeed <NAME>?  (I/Nne nwa a) ma obu nwanyi mmiri ara ya na-agba onyere <AHA> ara? | 1. Yes 2. No   9. Don’t know | | 🞎***2 or 9 → SQ5b.5*** | | |
| S5b.4.1 | | | Was <NAME> being breastfed at the time (her / his) fatal illness began?  A na-enye <AHA> ara oge oria gburu (ya) bidoro? | 1. Yes 2. No   9. Don’t know | | 🞎***1 or 9 → SQ5b.5*** | | |
| S5b.4.2 | | | How old was <NAME> when s/he was last breastfed?  Afo ole ka <AHA> di mgbe a kwusiri (ya) ara? | | | **__ __** Months  *(<1 = 00; DK = 99)* | | |
| S5b.5 | | | At the time the fatal illness began, was <NAME> being given any other liquid, including non-human milk or formula, fruit juice, tea or water, or any solid, semisolid, or soft foods?  Na ime ogea oria gburu <AHA> bidoro,ana enye ya udi mmiri ozo gunyere mmiri ara obula abughi nke madu ma obu ihe onunu umuaka ozo,mmiri mkpuru osisi,ihe onunu tea ma obu mmiri,nri ndi siri ike,nri ndi esichaghi ike,ma obu nri ndi di mere mere ?  *[Multiple answers allowed. Probe, and record all liquids and foods given.]* | 1. Ihe n’abughi mmiiri ara madu m’obu nke agwokoro agwoko 2. Mmiri ara nke ntutu etinyere mmiri nkiti 3. Nmanya mmiri uto,mmiri nkiti na obunmanya etinyere mmiri 4. ORS 5. Mkpuru mmiri m’obu ogwu mmiri) 6. Nri situru ike m’obu nri di nro 7. Onweghi ihe ozo karia mmiri ara   9. Amaghi | | 1. □  2. □  3. □ ***SQ5b.6***  4. □  5. □  6. □  7. □  ***SQ5b.6***  9. □ | | |
| S5b.5.1 | | | On most days before the illness began, how many times did <NAME> eat solid, semisolid, or soft foods other than liquids during the day or night?  Na ime otutu ubochi ndi sotara ubochi oria a bidoro,ugboro ole ka<AHA> riri nri siri ike nri ndi esichaghi ike,ma obu nri ndi di mere mere,m,obukwanu ihe onunu na ehihe ma obu na-abali? | | | __ __ Times  *(DK = 99)* | | |
| S5b.5.2 | | | Which of the following food types did <NAME> typically eat every day?  Kedu nri nke <AHA> na-erikari ubochi nile n,ime nri ndia ?  *[Read out all options and check “Yes,” “No” or “Don’t know” for each.]* | 1. Nri dika osikapa,ede ji 2. Agwa na ahuekere 3. Mmiri ara ehi 4. Anu,azu 5. Akwa 6. Mkpuru osisi na akwukwu nri nwere vitamin A 7. Mkpuru osisi na akwukwo nri ndi ozo ga si | | Yes No DK  1. □ 2. □ 9. □  1. □ 2. □ 9. □  1. □ 2. □ 9. □  1. □ 2. □ 9. □  1. □ 2. □ 9. □  1. □ 2. □ 9. □  1. □ 2. □ 9. □ | | |
| S5b.6 | | | Did <NAME> drink any liquids or semi-solid foods from a bottle with a nipple or teat?  <AHA> o jiri akarama nwere ihe dika onu ara nuo nri mmiri mmiri obula m,obu nri di mere mere? | 1. Yes 2. No   9. Don’t know | | 🞎 | | |
| S5b.7 | | | Now I would like to ask about the chlid’s vaccinations. Do you have a card where <NAME>’s vaccinations are written down?  Ugbua a choro iju maka ogwu mgbochi agbara nwata a.i nwere akwukwo ebe e denyere usoro e jiri gbaa < Aha>’ ogwu mgbochi.  *If “Yes,” ask:* May I see it please?  If yes ask: E nwere ike I hu ya? | 1. Yes, seen 2. Yes, but not seen 3. No card | | 🞎 ***2 or 3 → SQ5b.8*** | | |
| S5b.7.1 | | | Did <NAME> receive any vaccinations that are not included on this card, including vaccinations received in a national immunization day campaign?  E nyere <AHA> ogwu mgbochi obula edebaghi na-akwukwuo a tinyere ndi enyere ogea ndi oru ahu ike na-akpaghariwa n,ime ogbe n enye umuaka ogwu?  *If “Yes,” probe for vaccinations received but not recorded on the card.*  *[Record ‘Yes’ only if BCG, Polio 0-3, DPT 1-3 or PENTA 1-3, Measles, Yellow Fever and/or Hepatitis B1-3 vaccine(s) mentioned.]* | 1. Yes (received BCG, Polio 0-3, DPT 1-3 or PENTA 1-3, Measles, Yellow Fever and/or Hep B1-3 vaccinations that are not recorded on the card) 2. No   9. Don’t know | | 🞎 ***1 → Write ‘66’ in the corresponding day column below for each vaccination received but not recorded on the card.*** | | |
|  |  |  | *Copy vaccination date for each vaccine from the card. Record “99” or “9999” for partially unknown dates.*  *Write ‘88’ in ‘day’ column if card shows that a vaccination was given, but no date is recorded.*  *Do not leave any rows blank. Record “00” in the ‘day’ column for each vaccination that was not given.* | BCG  POLIO 0 (given at birth)  POLIO 1  POLIO 2  POLIO 3  DPT 1 / PENTA 1  DPT 2 / PENTA 2  DPT 3 / PENTA 3  MEASLES  YELLOW FEVER  HEPATITIS B1  HEPATITIS B2  HEPATITIS B3 | \|  \|  \|  \|  \|  \|  \|  \|  \| BCG \| \| --- \| --- \| --- \| --- \| --- \| --- \| --- \| --- \| --- \| \|  \|  \|  \|  \|  \|  \|  \|  \| P0 \| \|  \|  \|  \|  \|  \|  \|  \|  \| P1 \| \|  \|  \|  \|  \|  \|  \|  \|  \| P2 \| \|  \|  \|  \|  \|  \|  \|  \|  \| P3 \| \|  \|  \|  \|  \|  \|  \|  \|  \| DPT1 \| \|  \|  \|  \|  \|  \|  \|  \|  \| DPT2 \| \|  \|  \|  \|  \|  \|  \|  \|  \| DPT2 \| \|  \|  \|  \|  \|  \|  \|  \|  \| MSL \| \|  \|  \|  \|  \|  \|  \|  \|  \| YLFV \| \|  \|  \|  \|  \|  \|  \|  \|  \| HEP1 \| \|  \|  \|  \|  \|  \|  \|  \|  \| HEP2 \| \|  \|  \|  \|  \|  \|  \|  \|  \| HEP3 \|   Day Month Year | | | |
| S5b.8 | | | Did <NAME> ever receive any vaccinations to prevent her/him from getting diseases, including vaccinations received in a national immunization day campaign?  <AHA> o natatula ogwu mgbochi obula iji gbochie (ya) ibute oria,gunyekwa ndi enyere ya ogea ndi oru ahu ike na-akpaghariwa enye umuaka ogwu n’ime ogbe? | 1. Yes 2. No   9. Don’t know | | 🞎 ***2 or 9 → SQ5b.10*** | | |
|  |  | | Please tell me if <NAME> received any of the following vaccinations:  Biko gwam ma enyere <AHA> ogwu mgbochi obula n,ime ndia: |  | | | | |
|  | .1 | | A BCG vaccination against tuberculosis, that is, an injection in the arm or shoulder that usually causes a scar?  Ogwu mgbochi BCG imegide ukwara nta,ya bu ogwu ana-agba n,aka m,obu n,isi aka nke na-edebe apa? | 1. Yes 2. No   9. Don’t know | | 🞎 | | |
|  | .2 | | Polio vaccine, that is, drops in the mouth?  Ogwu mgbochi polio,ya bu nke ana atunye na-onu? | 1. Yes 2. No   9. Don’t know | | 🞎***2 or 9 → SQ5b.8.5*** | | |
|  | .3 | | When was the first polio vaccine received, just after birth or later?  Kedu mgbe tunyere ya nke izizi n,onu,ozugbo amuputachara ya k,obu mgbe ozo? | 1. Just after birth 2. Later   9. Don’t know | | 🞎 | | |
|  | .4 | | How many times was the polio vaccine received?  Ugboro ole ka atunyere ya ogwu mgbochi a n,onu? | | | **__ __** Times  *(DK = 99)* | | |
|  | .5 | | A DPT vaccination, that is, an injection given in the thighs or buttocks, sometimes at the same time as polio drops?  Ogwu mgbochi DPT,ya bu ogwu ana-agba na-apata ukwu m,obu n,ike,oge ufodu ana enyeko ya na nke ana I tunye n,onu? | 1. Yes 2. No   9. Don’t know | | 🞎***2 or 9 → SQ5b.8.7*** | | |
|  | .6 | | How many times?  Ugboro ole? | | | **__ __** Times  *(DK = 99)* | | |
|  | .7 | | A measles or MMR injection, that is, a shot in the arm at the age of 9 months or older, to prevent measles?  Ogwu mgbochi arubara m,obu MMR ya bu ogwu ana agba na-aka n,ime onwa itolu igbochi arubara? | 1. Yes 2. No   9. Don’t know | | 🞎 | | |
|  | .75 | | A yellow fever vaccination, that is, an injection given in the arm after the child is 9 months old? | 1. Yes 2. No   9. Don’t know | | 🞎 | | |
|  | .8 | | A Hep B vaccination, that is, an injection in the right thigh, sometimes given at the same time as DPT?  Ogwu mgbochi iba ocha anya,ya bu nke a na-agba na apata ukwu aka nri,oge ufodu a na agbakado ya na DPT? | 1. Yes  2. No  9. Don’t know | | 🞎***2 or 9 → SQ5b.9*** | | |
|  | .9 | | How many times was a Hep B vaccination received?  Ugboro ole ka a gbara ogwu mgbochi iba ochanaya a? | | | **__ __** Times  *(DK = 99)* | | |
| S5b.9 | | | Were any of the vaccinations <NAME> received given as part of a national immunization day campaign?  Ogwu mgbochi ndi a <AHA> natara onwere nke bu n,oge ndi oru ahuike na enye ogwu n,ime obodo ka ha nyere ya? | 1. Yes 2. No   9. Don’t know | | 🞎***2 or 9 → SQ5b.10*** | | |
| S5b.9.1 | | | At which national immunization day campaigns did <NAME> receive vaccinations?  Kedu ngaghari igba ogwu mgbochi nke ha noro gba ya ogwu mgbochi ahu?  *[Record all campaigns mentioned.]* | 1. <CAMPAIGN 1> (TYPE/DATE) 2. <CAMPAIGN 1> (TYPE/DATE) 3. <CAMPAIGN 1> (TYPE/DATE) 4. <CAMPAIGN 1> (TYPE/DATE) | | 1. □  2. □  3. □  4. □ | | |
| S5b.10 | | | (Before / In the six months before) the fatal illness, did <NAME> receive one or more vitamin A doses like this?  (Tupu/’n’ime onwa isi sotere) oria na -onwu<AHA> o natara ogwu mgbochi vitamin A dika nke a?  *[Read “Before…” if the child lived less than 6 months.]*  *[Show ampoule/capsule/syrup]* | 1. Yes, 1 dose 2. Yes, 2 or more doses 3. No   9. Don’t know | | 🞎 | | |
| S5b.11 | | | Before the fatal illness began, did <NAME> suffer from any of the following known conditions:  Tupu oria gburu <AHA> bido,o nwere udi ine ngosiputa onodu dika ndia na-ahu ya:  *[Read out all conditions and check “Yes,” “No” or “Don’t know” for each.]*  *If “Yes,” then ask:* O natara udi ogwugwo obula maka onodu ndia? | 1. Etoghi eto m’obu buo ibu ofuma 2. Nkwaru (ebum puta uwa):    1. isi, olu nakwa m’obu azu    2. Onu/Egbugbere onu    3. Obi    4. Ukwu nakwa/m’obu Aka 3. Asthma 4. Oria obi 5. Ukwara nta 6. Akwukwu/Ihe odido 7. ~~Nje oria mminwu/Oria mminwu~~ 8. Ndi ozo   *(specify other)* | | Suffered from  Yes No DK  1. □ 2. □ 9. □    1. □ 2. □ 9. □  1. □ 2. □ 9. □  1. □ 2. □ 9. □  1. □ 2. □ 9. □  1. □ 2. □ 9. □  1. □ 2. □ 9. □  1. □ 2. □ 9. □  1. □ 2. □ 9. □  1. □ 2. □ 9. □  1. □ 2. □ 9. □ | Treatment  Yes No DK  1. □ 2. □ 9. □    1. □ 2. □ 9. □  1. □ 2. □ 9. □  1. □ 2. □ 9. □  1. □ 2. □ 9. □  1. □ 2. □ 9. □  1. □ 2. □ 9. □  1. □ 2. □ 9. □  1. □ 2. □ 9. □  1. □ 2. □ 9. □  1. □ 2. □ 9. □ | |
|  |  |  |  |  |  | ___________________________ | | |
| **VA Section 4: Infant and child deaths (FOR CHILD DEATHS 28 DAYS—59 MONTHS OLD)**  *Read:* Now I’d like to ask you about <NAME>’s illness.  *Read:* Ugbua achorom iju gi ajuju gbasara oria <AHA> riara. | | | | | | | | |
| V4.1 | | During the illness that led to death, did the <NAME> have a fever?  Oge<AHA> na-aria oria a butere onwu ya onwere ahu ire oku ? | | 1. Yes 2. No   9. Don’t know | | 🞎 ***2 or 9 → VQ4.6*** | | |
| V4.2 | | How many days did the fever last?  Mkpuru ubochi ole ka ahu ire oku a noro?  *[Less than 24 hours = “00” days]* | | | | **__ __** Days  *(DK = 99)* | | |
| V4.3 | | Did the fever continue until death?  Ahu ya oregidere oku onwu o? | | 1. Yes 2. No   9. Don’t know | | 🞎 ***2 or 9 → VQ4.6*** | | |
| V4.4 | | How severe was the fever?  Kedu ka ahu ya si eredebe oku? | | 1. Mild 2. Moderate 3. Severe   9. Don’t know | | 🞎 | | |
| V4.5 | | What was the pattern of the fever?  Kedu ka o na esi emewe? | | 1. Continuous 2. On and off 3. Only at night   9. Don’t know | | 🞎 | | |
| V4.6 | | During the illness that led to death, did <NAME> have more frequent loose or liquid stools than usual?  Ogea o na-aria oria butetre onwu ya <AHA>o na anyu nsi esighi ike m,obu nsi di mmiri mmiri karia otu okwesiri? | | 1. Yes 2. No   9. Don’t know | | 🞎 ***2 or 9 → VQ4.12*** | | |
| V4.7 | | How many stools did <NAME> have on the day that loose liquid stools were most frequent?  Ugboro ole ka <AHA> jere mposi ubochi ahu onyuru nsi ahu esighi ike di mmiri mmiri karia? | | | | **__ __** Stools  *(DK = 99)* | | |
| V4.8 | | How many days before death did the frequent loose or liquid stools start?  Mkpuru ubochi ole tupu onwuo ka nsi esighi ike di mmiri mmiri ahu bidoro?  *[Less than 24 hours = “00” days]* | | | | **__ __** Days  *(DK = 99)* | | |
| V4.9 | | Did the frequent loose or liquid stools continue until death?  O nyugidere nsi esighi ike di mmiri mmiri ahu ruo oge onwuru? | | 1. Yes 2. No   9. Don’t know | | 🞎 ***1 or 9 → VQ4.11*** | | |
| V4.10 | | How many days before death did the loose or liquid stools stop?  Mkpuru ubochi ole tupu onwuo ka okwusiri inyu nsi esighi ike m,obu nke di mmiri mmiri tupu onwu o?  *[Less than 24 hours = “00” days]* | | | | **__ __** Days  *(DK = 99)* | | |
| V4.11 | | Was there visible blood in the loose or liquid stools?  Ana ahuta obara na ime nsi ahu esichagghi ike m,obu nke di mmiri mmiri? | | 1. Yes 2. No   9. Don’t know | | 🞎 | | |
| V4.12 | | During the illness that led to death, did the child have a cough?  Ogea o na-aria oria butere onwu ya nwa ahu onwere ukwara? | | 1. Yes 2. No   9. Don’t know | | 🞎 ***2 or 9 → VQ4.16*** | | |
| V4.13 | | For how many days did the cough last?  Mkpuru ubochi ole ka ukwara ahu noro?  *[Less than 24 hours = “00” days]* | | | | **__ __** Days  *(DK = 99)* | | |
| V4.14 | | Was the cough very severe?  Ukwara ahu o na esesiwe ya ike? | | 1. Yes 2. No   9. Don’t know | | 🞎 | | |
| V4.15 | | Did the child vomit after s/he coughed?  Nwa ahu o na –agbowa agbo ma okwacha ukwara? | | 1. Yes 2. No   9. Don’t know | | 🞎 | | |
| V4.16 | | During the illness that led to death, did <NAME> have difficult breathing?  Ogea o na aria oria butere onwu ya <AHA> enwe ihe nhia ahu iku ume ? | | 1. Yes 2. No   9. Don’t know | | 🞎 ***2 or 9 → VQ4.18*** | | |
| V4.17 | | For how many days did the difficult breathing last?  Mkpuru ubochi ole ka iku ume ihia ahu noro?  *[Less than 24 hours = “00” days]* | | | | **__ __** Days  *(DK = 99)* | | |
| V4.18 | | During the illness that led to death, did <NAME> have fast breathing?  Ogea o na aria oria butere onwu ya, <AHA> onwere ume ichi oku ? | | 1. Yes 2. No   9. Don’t know | | 🞎 ***2 or 9 → VQ4.20*** | | |
| ***Inst_1: If both VQ4.16 and VQ4.18 = 2 or 9 → VQ4.25*** | | | | | | | | |
| V4.19 | | For how many days did the fast breathing last?  Mkpuru ubochi ole ka onwere ume ichi oku ahu?  *[Less than 24 hours = “00” days]* | | | | **__ __** Days  *(DK = 99)* | | |
| V4.20 | | During the illness that led to death, did s/he have indrawing of the chest?  Ogea o na-aria oria butere onwu ya,obi (ya) o na-emikpu n,ime? | | 1. Yes 2. No   9. Don’t know | | 🞎 | | |
| V4.21 | | During the illness that led to death, did her/his breathing sound like any of the following?  Ogea o na-aria oria butere onwu ya,obi (ya) omere udi mkpotua?  *[Demonstrate each sound/show video]* | |  | |  | | |
| V4.22 | | Isu asu Stridor | | 1. Yes 2. No   9. Don’t know | | 🞎 | | |
| V4.23 | | Grunting | | 1. Yes 2. No   9. Don’t know | | 🞎 | | |
| V4.24 | | Ikwo ekwo Wheezing | | 1. Yes 2. No   9. Don’t know | | 🞎 | | |
| V4.25 | | Did <NAME> experience any generalized convulsions or fits during the illness that led to death?  O di mgbe <AHA> nwere nsogbu ihe ndudu n,oge oria a mere ka onwuo? | | 1. Yes 2. No   9. Don’t know | | 🞎 | | |
| V4.26 | | Was <NAME> unconscious during the illness that led to death?  Ogea o na-aria ahu tupu<AHA> onwuo o na o maghizi onwe ya m,obu onye madu bu? | | 1. Yes 2. No   9. Don’t know | | 🞎 ***2 or 9 → VQ4.28*** | | |
| V4.27 | | How long before death did unconsciousness start?  Kedu oge amaghizi onwe m,obu onye madu bu a bidoro tupu onwuo? | | 1. Less than 6 hours 2. 6-23 hours 3. 24 hours or more   9. Don’t know | | 🞎 | | |
| V4.28 | | Did <NAME> have a stiff neck during the illness that led to death?  Mgbe <AHA> na aria oria a mere ka onwuo o na o nweghi ike imeghari onu ya ?  *[Demonstrate/show video]* | | 1. Yes 2. No   9. Don’t know | | 🞎 | | |
| V4.29 | | Did <NAME> have a bulging fontanelle during the illness that led to death?  Mgbe <AHA> na aria oria mere ka o nwuo, o nwere ihe toputere ya n,etiti isi?  *[Show photo]* | | 1. Yes 2. No   9. Don’t know | | 🞎 | | |
| V4.30 | | During the month before s/he died, did <NAME> have a skin rash?  N,ime onwa ahu onwuru,tupu (ya) nwuo ihe o bukasiri <AHA> n,ahu ? | | 1. Yes 2. No   9. Don’t know | | 🞎 ***2 or 9 → VQ4.35*** | | |
| V4.31 | | Where was the rash?  Kedu akuku ahu ya ihe ahu bukasiri? | | 1. Face 2. Trunk/Abdomen 3. Extremities 4. Everywhere   9. Don’t know | | 🞎 | | |
| V4.32 | | Where did the rash start?  Kedu akuku ahu ya ihea buru ya siri bido? | | 1. Face 2. Trunk/Abdomen 3. Extremities 4. Everywhere   9. Don’t know | | 🞎 | | |
| V4.33 | | How many days did the rash last?  Mkpuru ubochi ole ka ihe obubu ahu noro? | | | | **__ __** Days  *(DK = 99)* | | |
| V4.34 | | Did the rash have blisters containing clear fluid?  Ihe obubu ahu onwegasiri onu mmiri dicha n,ime ya? | | 1. Yes 2. No   9. Don’t know | | 🞎 | | |
| V4.35 | | During the illness that led to death, did <NAME>’s limbs (legs, arms) become very thin?  Ogea o na –aria ahu butere onwu ya ukwu na-aka <AHA>’a tara nnukwu ahu?  *[Show photo]* | | 1. Yes 2. No   9. Don’t know | | 🞎 | | |
| V4.36 | | During the illness that led to death, did <NAME> have swollen legs or feet?  Ogea o na-aria ahu butere onwu ya <AHA> ukwu ya o zara aza? | | 1. Yes 2. No   9. Don’t know | | 🞎 ***2 or 9 → VQ4.38*** | | |
| V4.37 | | How long did the swelling last?  Ogologo ubochi ole ka ukwu oziza a noro?  *[Record days or weeks.]* | | | | **__ __** Days  *(DK = 99)* | | |
|  |  |  |  |  |  | **__ __** Weeks  *(DK = 99)* | | |
| V4.38 | | During the illness that led to death, did <NAME>’s skin flake off in patches?  Ogea o na-aria oriaa butere onwu ya akpukpo ahu ya o na-ewechapu nwegasia apa <AHA>’? | | 1. Yes 2. No   9. Don’t know | | 🞎 | | |
| V4.39 | | Did <NAME>’s hair change in color to a reddish or yellowish color?  Ntutu isi <AHA> o chaghariri ka o sib u acha chazia edo-edo m,obu mmee mmee? | | 1. Yes 2. No   9. Don’t know | | 🞎 | | |
| V4.40 | | Did <NAME> have a protruding belly?  Afo <AHA> o toro karia ahu ya ndi ozo? | | 1. Yes 2. No   9. Don’t know | | 🞎 | | |
| V4.41 | | During the illness that led to death, did <NAME> suffer from “lack of blood” or “pallor”?  Ogea o na-aria oria butere onwu ya obara o koro <AHA> na ahu? | | 1. Yes 2. No   9. Don’t know | | 🞎 | | |
| V4.42 | | During the illness that led to death, did <NAME> have swelling in the armpits?  Ogea o na-aria oria butere onwu ya etuto o toro <AHA> na-abu? | | 1. Yes 2. No   9. Don’t know | | 🞎 | | |
| V4.43 | | During the illness that led to death, did <NAME> have a whitish rash inside the mouth or on the tongue?  Ogea o na-aria oria butere onwu ya ihe n,achagasi ocha oburu <AHA> n,onu m,obukwanu n,ire ? | | 1. Yes 2. No   9. Don’t know | | 🞎 | | |
| V4.44 | | During the illness that led to death, did <NAME> bleed from anywhere?  Ogea o na-aria oria a butere onwu ya,obara osi n,akuku ahu <AHA> obula aputa? | | 1. Yes 2. No   9. Don’t know | | 🞎 ***2 or 9 → VQ4.46*** | | |
| V4.45 | | *Record from where s/he bled:* | |  | | | | |
| V4.46 | | During the illness that led to death, did s/he have areas of the skin that turned black?  Ogea o na-aria oria butere onwu (ya),akuku ahu (ya) obula o chaghariri jiwe oji? | | 1. Yes 2. No   8. Don’t know | | 🞎 | | |
| V4.47 | | Did <NAME> suffer from an injury or accident such as…?  <AHA> o nwere ihe mberede m,obu merou ahu n,udi obula dika nkea a?  *[Ask the respondent each in sequence and mark each as “Yes,” “No” or “Don’t know.”]* | | 1. Ihe mgberede okporo uzo/ihe mmeru ahu? 2. odida? 3. idanye? 4. inyegbu?   O tara afufu:   1. otara afufu maka ihe otita m’obu ihe ogbugba? 2. Ihe orire? 3. Ogbaghara ka obu ogburu onwe ya,ka obu (Mkpari)? 4. Onwere ihe meru ahu ozo?   *(If “Yes,” then specify)* | | Yes No DK  1. □ 2. □ 9. □  1. □ 2. □ 9. □  1. □ 2. □ 9. □  1. □ 2. □ 9. □  1. □ 2. □ 9. □  1. □ 2. □ 9. □  1. □ 2. □ 9. □  1. □ 2. □ 9. □ | | ***All = 2 or 9***  ***→ SQ6.1*** |
|  |  |  |  |  |  | ___________________________ | | |
| V4.48 | | Was the injury or accident intentionally inflicted by someone else?  Mmeru ahu ma obu ihe mberede ahu obu ihe onye oze kpachara anya mee? | | 1. Yes 2. No   8. Don’t know | | 🞎 | | |
| V4.49 | | How long did <NAME> survive after the injury or accident?  Ogologo oge ole ka <AHA> noro ndu ka ihe mberede ahu mechara  *[Record hours if less than 24 hours—Less than 1 hour = “00” hours;*  *Record days if 1 day or more.]* | | | | **__ __** Hours  *(DK = 99)* | | |
|  |  |  |  |  |  | **__ __** Days  *(DK = 99)* | | |

| **SA Module 6: Care-seeking for the child’s fatal illness (FOR NN & CHILD DEATHS 0—59 MONTHS OLD)**  ***Read:*** Now, I’d like to ask you about <NAME>’s fatal illness and the care and treatments that s/he received.  ***Read:*** Ugbua a chorom iju gi ihe gbasara oria <AHA> riaria nwuo,nlezi anya nakwa ogwugwo enyere ya. | | | | | | | | | | | | | | | | | | |
| --- | --- | --- | --- | --- | --- | --- | --- | --- | --- | --- | --- | --- | --- | --- | --- | --- | --- | --- |
| S6.1 | Who first noticed that <NAME> was ill?  Kedu onye mbu choputara na <AHA> na aria oria? | | | | | | | | 1. The respondent 2. Other relative, neighbor, friend 3. CHW or nurse at home or in community 4. Doctor or nurse at a health facility 5. Other *(specify)* | | | | | | | 🞎  ___________________________ | | |
| S6.2 | Earlier you said that <NAME> had <SYMPTOM(S)> during her/his illness  Na mbu I gwaram na<AHA> nwere otutu nsogbu (ga) ogea (O) oriara oria .  *[Read back all the child’s symptoms from VA section 3 (for neonates) or VA section 4 (for children).]*  How did <SQ6.1 PERSON> first know that <NAME> was ill? Which of these symptoms did s/he have at that time?  Kedu ka <SQ6. Onye mbu > siri mata na mbu na.<AHA> na-aria oria ?kedu nsogbugasi onwere n’ime oge ahu?  What symptoms did s/he have next? On what day of the illness did these symptoms start?  Kedu ihe ngosi (ama) nke sochiri nke ahu?N,ime ubochi nke ole oria ahu bidoro ka ihe ngosi (ama) ndia ahu bidoro?  *[Probe until all the symptoms are recorded in the order they appeared.]* | | | | | | | | **Symptoms in order of appearance** | | | | | | | **Illness day the symptom started** | | |
|  |  |  |  |  |  |  |  |  | 1. | | | | | | |  | | |
|  |  |  |  |  |  |  |  |  | 2. | | | | | | |  | | |
|  |  |  |  |  |  |  |  |  | 3. | | | | | | |  | | |
|  |  |  |  |  |  |  |  |  | 4. | | | | | | |  | | |
|  |  |  |  |  |  |  |  |  | 5. | | | | | | |  | | |
|  |  |  |  |  |  |  |  |  | 6. | | | | | | |  | | |
|  |  |  |  |  |  |  |  |  | 7. | | | | | | |  | | |
| S6.3 | When <SQ6.1 PERSON> first noticed that <NAME> was ill, was s/he…  Mgbe <SQ6.onye mbu > hutara na –izizi na <AHA> na aria oria (o) na…  *[Read the choices for each condition.]* | | | | | | | | 1. Iri nri nke oma,adighi eri ihe nke oma m’obu odighi eri ihe obula 2. ikienkwucha, ihe na ebu aje, or Omaghi onwe ya 3. Ahu siri ye ike,Ahu esiya ike nke oma ma obu odighi aga aga | | | | | | | Normal Medium Abnormal DK  1. □ 2. □ 3. □ 9. □  1. □ 2. □ 3. □ 9. □  1. □ 2. □ 3. □ 9. □ | | |
| S6.4 | Did <NAME> receive, or did you seek or try to seek, any care or treatment for the fatal illness?  O di mgbe<AHA> natara ma obu mgbe i choro ma obu mgbe I gbara mbo icho nlezi anyanma o bu ogwugwo maka oria gburu nwata a? | | | | | | | | 1. Eee 2. Mbaa—Ochoghi enyemaka,enyehu ya m’obu ochoghi 3. Mba — onwuru ozigbo   9. Amaghi | | | | | | | 🞎***2 → SQ6.6***  ***3 or 9 → VQ5.10*** | | |
| S6.5 | Please tell me everything you did for <NAME>’s fatal illness inside the home and all the places outside the home you took or tried to take (her / him) for health care. Start with the first care or treatment <NAME> received and then, in order, tell me all the other care and treatments s/he received.  Now, what was the first thing you did or tried to do for <NAME>’s illness?  *For neonatal deaths only:* Did the illness begin at the health provider where the child was delivered?  On what day of the illness was the action taken?  For what symptoms was the action taken?  Biko ko charam ihe nile i mere gbasara oria <AHA> riaria nwuo ndi I mere n,ulo n,akwa ndi emere n,ebe ndi ozo nile ikpo (ya) ga ma ebe obula I gbara mbo ikpo ya ga maka nlezi anya ahu ike.Bido na nlezi anya m,obu ogwugwo nke mbu e nyere’<AHA> n,oge ahu,ijirizia usoro na usoro kocharam nlezi anya nakwa ogwugwo ndi ozo nile enyere (ya).  Ugbua kedu ihe izizi I mere m’obu I gbaliri ime maka oria <AHA>  Maka umuazi nwuru n’ime otu onwa:ora ahu o bidoro na-ebe onye oru ahu ike ebe a noro muo nwa ahu?  Kedu ubochi ole oriagoro oria tupu eme ihe emere?  Kedu nsogugasi nke ejiri mee ihe emere?  *[Include any provider <NAME> did not reach because s/he died before leaving home or on route.]*  *Tinye onye nlezi anya obula <AHA> eji maka na (O) nwuru tupu ahapu ulo m’obu oge ano n’uzo?*  *(1) Check one other care or health provider box for each action row. (2) For neonatal deaths only: If the illness began at the health provider where the child was delivered, then mark that as Action 1 and check the “illness began at provider” box. (3) Record the illness day each action was taken. (4) Ensure no action was taken for a symptom before it started (in SQ6.2).* | | | | | | | | | | | | | | | | | |
| **Action #** | **(1)**  **Other care** | | | **(1)**  **Health Providers** | | | | | | | | | | **(3)**  **Illness day the action was taken** | | | **(4)**  **For what symptom(s) was the action taken?** | |
|  | **Home care (own, relative, neigh-bor, friend)** | **Tradi-tional or non-formal provider** | **Phar-macist or drug seller** | **Trained CH**  **Worker, nurse, or midwife** | | **Private doctor**  **(formal/unsure)** | | | | **NGO or govt. clinic** | **Hospital** | **(2)**  **Illness began at provider where child was delivered** | |  |  |  |  |  |
| 1. | 🞎 | 🞎 | 🞎 | 🞎 | | 🞎 | | | | 🞎 | 🞎 | 🞎 | | **__ __**  *(DK = 99)* | | |  | |
| 2. | 🞎 | 🞎 | 🞎 | 🞎 | | 🞎 | | | | 🞎 | 🞎 |  | | **__ __**  *(DK = 99)* | | |  | |
| 3. | 🞎 | 🞎 | 🞎 | 🞎 | | 🞎 | | | | 🞎 | 🞎 |  | | **__ __**  *(DK = 99)* | | |  | |
| 4. | 🞎 | 🞎 | 🞎 | 🞎 | | 🞎 | | | | 🞎 | 🞎 |  | | **__ __**  *(DK = 99)* | | |  | |
| 5. | 🞎 | 🞎 | 🞎 | 🞎 | | 🞎 | | | | 🞎 | 🞎 |  | | **__ __**  *(DK = 99)* | | |  | |
| 6. | 🞎 | 🞎 | 🞎 | 🞎 | | 🞎 | | | | 🞎 | 🞎 |  | | **__ __**  *(DK = 99)* | | |  | |
| 7. | 🞎 | 🞎 | 🞎 | 🞎 | | 🞎 | | | | 🞎 | 🞎 |  | | **__ __**  *(DK = 99)* | | |  | |
| ***Inst_1: (For neonatal deaths only) If illness began at health provider where child was delivered:***  ***And did not fill L&D matrix (module 4) → SQ6.10; And filled L&D matrix (module 4) → SQ6.16*** | | | | | | | | | | | | | | | | | | |
| S6.6 | *If no care given or sought, ask:* Who decided that <NAME> did not need any care or treatment for the illness?  *If no care given or sought, ask:* Kedu onye kpebiri na<AHA> ekwesighi ka enye ya nlezi anya ma obu ogwugwo obula maka oria a ?  *If any care given or sought, ask:* Who decided that <ACTION 1> was the first thing to do for <NAME>’s illness?  *If any care given or sought, ask:* kedu onye kpebiri na nlezi anya na ogwugwo a bu ihe mbu I ga eme <ACTION 1> maka oria a <AHA>’  *[Record the one main decision maker.]* | | | | | | | | 1. Child’s mother 2. Child’s father 3. Child’s aunt 4. Child’s uncle 5. Child’s grandmother 6. Child’s paternal grandfather 7. Child’s maternal grandfather 8. Other *(specify)*   9.Don’t know | | | | | | | 1. □  2. □  3. □  4. □  5. □  6. □  7. □  8. □ _______________________  9. □ | | |
| S6.7 | *If never taken to a health provider, ask:* Did you have any concerns or problems that kept you from taking <NAME> to a health provider during his/her illness?  *If never taken to a health provider, ask:* I nwere nsogbu ma obu ihe gbochiri gi I kpo nwata a gaa na nke ndi oru ahuike oge o no n,aria?  *If taken to a health provider, ask:* Did you have to overcome any concerns or problems to take <NAME> to the (first) health provider?  *If taken to a health provider, ask:* O nwere ihe mgbochi ma obu nsogbu I gabigara tupu I nwee ike kpoga<AHA>na nke onye oru ahuike nke mbu? | | | | | | | | 1. Yes 2. No   9. Don’t know | | | | | | | 🞎 ***2 or 9 → Inst_2*** | | |
| S6.7.1 | What concerns or problems did you have?  Kedu ihe bu ihe mgbochi na nsogbu I nwegasiri?  *Prompt:* Was there anything else?  *Prompt:* O nwere ihe ndi ozo?  *[Multiple answers allowed.]* | | | | | | | | 1. O cheghi na o na –aria oria nke ruru I choga nlezi anya ahuike 2. Onweghi onye no nso iso ya gaa 3. Ga enwepu otutu oge n’ihe o na eme 4. Onye ozo ga-enye ikike 5. O tere aka ije 6. Onweghi ihe ga eburu /kworo madu ga 7. Ego eji akwu ugwo 8. Nlezi anya ana-enye n’ulo ahuike ejughi ya afo 9. Udi ihe amaa kwesiri ka agwo ya n’uzo odinala 10. O chere na o na-aria nukwu oria ekwesighi ibu ga njem 11. Ochere na ya na nwa ga anwu agbanyeghi nlezi anya 12. OO ime abali erughi ihe ga eburu madu jee m’obu onye nlezi anya a noghi nso 13. Ihe ndi ozo *(specify)*   99.Amaghi | | | | | | | 1. □  2. □  3. □  4. □ _______________________  5. □  6. □  7. □  8. □  9. □  10. □  11. □  12. □  13. □ ______________________  99. □ | | |
| ***Inst_2: If SQ6.4 = 2 (No care given) or***  ***If SQ6.5 ≠ “Health Provider” (Never took and never tried to take to a health provider) → SQ6.39*** | | | | | | | | | | | | | | | | | | |
| S6.8 | *Refer to SQ6.5 for the first health provider and related symptoms:*  You mentioned that you took <NAME> to the (first) health provider, I mean the <FIRST HEALTH PROVIDER> for <SYMPTOM(S)>. How long had <NAME> had (this / these) symptom(s) when it was decided to take him/her to the <FIRST HEALTH PROVIDER>?  < Onye nlezi anya nke izizi > nye<nsogbu (ga)> ogologo oge ole <AHA> nwegoro (Nsogbua/nsogbu (ga) tupu ekpebie ikpoga (ya) na nke <onye nlezi anya nke izizi>?  *[Read “…to the first…” if took or tried to take to more than one health provider.]*  *[Mark days, hours &/or minutes as needed: e.g. 00 day, 02 hours, 10 minutes]* | | | | | | | | | | | | | | | **__ __** Days  *(DK = 99)* | | |
|  |  |  |  |  |  |  |  |  |  |  |  |  |  |  |  | **__ __** Hours  *(DK = 99)* | | |
|  |  |  |  |  |  |  |  |  |  |  |  |  |  |  |  | **__ __** Minutes  *(DK = 99)* | | |
| ***Child illness matrix instructions:*** *Ask the following questions for the first and last health providers where care was sought or tried to be sought for the fatal illness. Ask all the questions for the first provider before going on to the last.*  *Before asking about the first health provider, read:*  Now I would like to ask you about your visit to the (first) health provider. *[Read “first” if went or tried to go to more than one provider.]*  Ugbua a gam a cho iju gi gbasara otu I siri gaa na nke onye oru ahuike nke ikpeazu*.]*  *Before asking about the last health provider, read:*  Now I would like to ask you about your visit to the last health provider, I mean the <LAST HEALTH PROVIDER>.  Ugbua agam acho iju gi gbasara otu isiri gaa na nke onye oru ahuike <nke ikpeazu>. | | | | | | | | | | | | | | | | | | |
| **– CHILD ILLNESS MATRIX QUESTIONS –** | | | | | | | | | | | | | **FIRST HEALTH PROVIDER** | | | | | **LAST HEALTH PROVIDER** |
| At the time when it was decided to take <NAME> to the <FIRST/LAST HEALTH PROVIDER>, was s/he…  Mgbe e kpebiri I kporo “AHA” gaa na nke onye oru ahuike <nke mbu/nke ikapzu> o na…  *[Read the choices for each condition.]* | | | | | 1. Iri nri nke oma adighi eri ihe nke oma m’obu adighi eri ihe obula 2. Ike nkwucha, ihe na ebu aju, m’obu omaghi onwe ya 3. Ahu siri ya ike,ahu esiya ike nke oma m’obu odighi aga aga | | | | | | | | S6.9  Nrml Med Abnrm DK  1. □ 2. □ 3. □ 9. □  1. □ 2. □ 3. □ 9. □  1. □ 2. □ 3. □ 9. □ | | | | | S6.24  Nrml Med Abnrm DK  1. □ 2. □ 3. □ 9. □  1. □ 2. □ 3. □ 9. □  1. □ 2. □ 3. □ 9. □ |
| What was the name of the <FIRST/LAST HEALTH PROVIDER> where you took <NAME>?  Kedu aha ebe nlezi anya ahu ike <nke mbu/nke ikpazu> I kporo <AHA> gaa?  *Probe to identify the type of provider.* | | | | | 1. Hospital (Government) 2. Hospital (NGO) 3. Hospital (Private) 4. Health center (Government) 5. Health center (NGO) 6. Health post (Government) 7. Health post (NGO) 8. Private doctor/clinic (Formal) 9. Private doctor/clinic (?Formal?) 10. Trained community health worker, nurse, or midwife   99. Don’t know | | | | | | | | S6.10  🞎🞎  ___________________  (Name of Provider or Facility) | | | | | S6.25  🞎🞎  ___________________  (Name of Provider or Facility) |
| After (deciding to seek care / being referred), how much time passed before going to the <FIRST/LAST HEALTH PROVIDER>?  Ka ekpebichara I je choga nlezi anya ahu ike/e zigachara gi I ga hu onye/ulo nlezi anya ahu ike ogologo oge ole gachara tupu I gaa ebe nlezi anya <nke mbu/nke ikpazu >?  *[Discuss that this might include the time needed to arrange for transportation and money to go to the provider/facility, or to provide home care or go to a traditional provider before going to the health provider.]*  *[If the child died at home, record the time from decision/referral to death.]*  *[Mark days, hours &/or minutes as needed: e.g. 01 day, 05 hours, 30 minutes]* | | | | | | | | | | | | | S6.11  **__ __** Days  *(DK = 99)* | | | | | S6.26  **__ __** Days  *(DK = 99)* |
|  |  |  |  |  |  |  |  |  |  |  |  |  | **__ __** Hours  *(DK = 99)* | | | | | **__ __** Hours  *(DK = 99)* |
|  |  |  |  |  |  |  |  |  |  |  |  |  | **__ __** Minutes  *(DK = 99)* | | | | | **__ __** Minutes  *(DK = 99)* |
| Was there any cost to travel to the <FIRST/LAST HEALTH PROVIDER> or pay for the child’s care there?  O di ugwo a kwuru iji mee njem gaa na nke onye oru ahuike <nke mbu/nke ikpazu> ma o bu maka nlezi anya nwata a ebe ahu? | | | | | 1. Yes 2. No   9. Don’t know | | | | | | | | S6.12  🞎 ***2 or 9 → SQ6.13*** | | | | | S6.27  🞎 ***2 or 9 → SQ6.28*** |
| How did you arrange for the money for these expenses?  Kedu ka I siri nweta ego I jiri kwuo ugwo a?  *[Multiple answers allowed.]* | | | | | 1. Had available 2. Borrowed 3. Sold assets 4. Help from kin/relatives 5. Community fund 6. Govt. scheme 7. Other   9. Don’t know | | | | | | | | S6.12.1  1. □  2. □  3. □  4. □  5. □  6. □  7. □  9. □ | | | | | S6.27.1  1. □  2. □  3. □  4. □  5. □  6. □  7. □  9. □ |
| What transportation method was used to go there?  Kedu udi ugbo I jiri mee njem ebe ahu?  *[Multiple answers allowed.]* | | | | | 1. Walk 2. Bicycle/rickshaw/cart/ boat 3. Bus 4. Taxi/auto/trecker 5. Ambulance (auto or motorcycle) 6. Other 7. Could not arrange transport   9. Don’t know | | | | | | | | S6.13  1. □ ***If only walk***  2. □ ***→ SQ6.14.1***  3. □  4. □  5. □  6. □  7. □ ***→ SQ6.14.1***  9. □ | | | | | S6.28  1. □ ***If only walk***  2. □ ***→ SQ6.29.1***  3. □  4. □  5. □  6. □  7. □ ***→ SQ6.29.1***  9. □ |
| How much did the transportation cost?  Ego ole ka I kwuru maka njem ahu? | | | | | | | | | | | | | S6.14  **__ __ __ __** unit  *(DK=9999)* | | | | | S6.29  **__ __ __ __** unit  *(DK = 9999)* |
| Did the child reach the <FIRST/LAST HEALTH PROVIDER> before s/he died?  Nwa ahu oruru ebe nlezi anya ahuike <nke mbu/nke ikpazu> tupu onwuo?  *[If “No,” discuss with respondent to determine correct response: 2, 3 or 4.]* | | | | | 1. Ee oruru tupu nwata a nwuo 2. Mba, onwuru tupu o puwa 3. Mba , onwuru n’uzo tupu erute 4. Mba, onweghi ike iru n’ebe ndi ulo ahuike/agaghi aga/olaghachiteghi ulo/omere ihe ndi ozo   9. O maghi | | | | | | | | S6.14.1  🞎 ***2, 3 → SQ6.39***  ***4, 9 → Inst_4*** | | | | | S6.29.1  🞎 ***2-9 → SQ6.39*** |
| How long did it take to travel to the <FIRST/LAST HEALTH PROVIDER>?  Ogologo oge ole ka o were iji ruo na nke onye oru ahuike <nke mbu/nke ikpazu>?  *[Mark hours &/or minutes as needed: e.g. 02 hours, 10 minutes]* | | | | | | | | | | | | | S6.15  **__ __** Hours  *(DK = 99)* | | | | | S6.30  **__ __** Hours  *(DK = 99)* |
|  |  |  |  |  |  |  |  |  |  |  |  |  | **__ __** Minutes  *(DK = 99)* | | | | | **__ __** Minutes  *(DK = 99)* |
| What did the <FIRST/LAST HEALTH PROVIDER> do for <NAME>’s problem?  Kedu ihe onye oru ahuike <nke mbu/nke ikpazu> mere maka nsogbu nwata a <AHA>?  *Prompt:* Was there anything else?  *Prompt:* O nwere ihe ozo?  *[Multiple answers allowed.]* | | | | | 1. Onyere ikuku 2. Ojiri akpa m’obu obu ihe nmehir onu nyere aka iku ume 3. Osiri n’onu nye ya ihe mmiri 4. Enyere ya ogwu mgbochi oria site na onu 5. Nyere ogwu mgbochi oria ofufe na onu 6. Nyere ogwu nwete ike ORS 7. Enyere Vitamin A 8. Onyere egwu ndi ozo site n’onu 9. Enyere IM medicine 10. Enyere mmiri ihe ndi ozo m’obu ogwu 11. Aduru ya odu ka ozuo ogwu ebe no abughi ogige ulo ogwu 12. Awara ya afo *(specify)* 13. Anabatara na ulo ogwu 14. Ihe ndi ozo *(specify)* 15. Onweghi   99.Amgahi | | | | | | | | S6.16  1. □  2. □  3. □  4. □  5. □  6. □  7. □  8. □ _______________  9. □  10. □  11. □  12. □ ______________  13. □ stayed __ __ days  14. □ ______________  15. □ ***→ SQ6.18***  99. □ ***→ SQ6.18*** | | | | | S6.31  1. □  2. □  3. □  4. □  5. □  6. □  7. □  8. □ _______________  9. □  10. □  11. □  12. □ ______________  13. □ stayed __ __ days  14. □ ______________  15. □ ***→ SQ6.33***  99. □ ***→ SQ6.33*** |
| How much did you pay for these treatments and other costs related to the health care, including the admission fee, consultation, lab tests, equipment, and room and food for companions?  Ego ole ka I kwuru maka ogwugwo a na ihe ndi ozo metutara nlezi anya nwata a,ma etinyere,ego nnabata,ego iji hu onye oru ahuike,nyocha,ngwa oru onu ulo na ihe oriri? | | | | | | | | | | | | | S6.17  **__ __ __ __ __** unit  *(DK = 99999)* | | | | | S6.32  **__ __ __ __ __** unit  *(DK = 99999)* |
| Did the <FIRST/LAST HEALTH PROVIDER> refer <NAME> to another health provider or facility?  Ebe nlezi anya <nke mbu/nke ikpazu> ozipuru ka a kporo <AHA> ga hu onye nlezi anya m,obu ulo ahu ike ozo? | | | | | 1. Yes 2. No   9. Don’t know | | | | | | | | S6.18  🞎 ***2 or 9 → SQ6.19*** | | | | | S6.33  🞎 ***2 or 9 → SQ6.34*** |
| Why was <NAME> referred?  Kedu ihe o jiri ziga <AHA> ebe ozo?  *[Multiple answers allowed.]* | | | | | 1. Onye oru ahuike enweghi ike ihazi nsogbu ahu 2. Ihe ndi achoro (dika egwu,ikiku) adighi 3. Akara ngwa achoro (dika igwe) adighi   9. Amaghim | | | | | | | | S6.18.1  1. □  2. □  3. □  9. □ | | | | | S6.33.1  1. □  2. □  3. □  9. □ |
| Did <NAME> leave the <FIRST/LAST HEALTH PROVIDER> alive?  <AHA> o puru na nke onye oru ahuike <nke mbu/nke ikpazu> na ndu? | | | | | 1. Yes, left alive 2. No, died at this provider | | | | | | | | S6.19  🞎 ***2 → VQ5.4*** | | | | | S6.34  🞎 ***2 → VQ5.4*** |
| Did the <FIRST/LAST HEALTH PROVIDER> suggest that you do anything for <NAME>’s illness after leaving?  Onye oru ahuike <nke mbu/nke ikpazu> o nwere ihe o kwuru ka I mee maka oria<AHA>’ tupu i laa? | | | | | 1. Yes 2. No   9. Don’t know | | | | | | | | S6.20  🞎 ***2 or 9 → SQ6.22*** | | | | | S6.35  🞎 ***2 or 9 → SQ6.37*** |
| What did the <FIRST/LAST HEALTH PROVIDER> suggest that you do?  Kedu ihe ebe nlezi anya < nke mbu/nke nke ikpazu > gwara gi mee?  *Prompt:* Was there anything else?  *Prompt:* Onwere ihe ozo?  *[Multiple answers allowed.]* | | | | | 1. Inyekwa ara 2. Nyekwa nmiri 3. Kana enyekwa ara 5. Nye ogwu omimi na onu 6. Nye ogwu mgbochi aru oku na onu 7. Tinye ya ogwu mgbochi anya nmgbu na onu 8. Loghachite maka nleta 9. Loghachi,ma obu ibia ma okaria 10. Bia ugboro ole agwara gi 11. Ihe ndi ozo*(specify)*   99. Amaghi | | | | | | | | S6.20.1  1. □  2. □  3. □  4. □  5. □  6. □  7. □  8. □  9. □  10. □  11. □ ______________  99. □ ***→ SQ6.22*** | | | | | S6.35.1  1. □  2. □  3. □  4. □  5. □  6. □  7. □  8. □  9. □  10. □  11. □ ______________  99. □ ***→ SQ6.37*** |
| Were you able to follow all the advice?  I nwere ike mee ihe ahu nile o duru gi ndumodu ka I mee? | | | | | 1. Yes 2. No   9. Don’t know | | | | | | | | S6.21  🞎 ***9 → SQ6.22*** | | | | | S6.36  🞎 ***9 → SQ6.37*** |
| *If not able to follow all the advice, ask:*  Did you have any concerns or problems that kept you from following the advice?  O di ihe mere ma obu nsogu gbochiri gi I gbaso ndumodu ahu?  *:* Did you have to overcome any concerns or problems to follow the advice?  *If able to follow all the advice, ask:* O di ihe gbochiri ma obu nsogbu I gabigara ka i were gbaso ndumodu ahu? | | | | | 1. Yes 2. No   9. Don’t know | | | | | | | | S6.21.1  🞎 ***2 or 9 → SQ6.22*** | | | | | S6.36.1  🞎 ***2 or 9 → SQ6.37*** |
| What concerns or problems did you have?  Kedu ihe bu nsogbu m,obu ihe ndahachi azu ahu?  *Prompt:* Was there anything else?  *Prompt:* Onwere ihe ozo?  *[Multiple answers allowed.]* | | | | | 1. Ohotaghi ntuzi aka 2. Ezigbo ohere n’ulo oru 3. Onye ozo tuputara alo 4. Obu n’ukwu ego 5. Nsogbua kwesiri ihe odinala 6. Achozighi atumatu ozo 7. Oke nkechi nwere ike imebi nwata 8. Oto osorogi jide nwata agaghi egbochi onwu 9. Oghere adighi iga n’ozo 10. Nwata nwuru na oge 11. Kwuputa ndi ozo *(specify)*   99 Amgahi | | | | | | | | S6.21.2  1. □  2. □  3. □ _______________  4. □  5. □  6. □  7. □  8. □  9. □  10. □  11. □ ______________  99. □ | | | | | S6.36.2  1. □  2. □  3. □ _______________  4. □  5. □  6. □  7. □  8. □  9. □  10. □  11. □ ______________  99. □ |
|  | | | | | | | | | | | | | | | | | |  |
| At the time of leaving the <FIRST HEALTH PROVIDER> / <LAST HEALTH PROVIDER>), was <NAME>…  Na oge ana hapu (<ebe nlezi anya nke mbu/nke ikpazu> / <LAST HEALTH PROVIDER>), <AHA> o na…  *[Read the choices for each condition.]* | | | | | 1. Iri nri nke oma,adighi eri eri nke oma,m’obu adighi eri ihe obula 2. Ike nkwuchat, ihe na ebu aju, m’obu omaghi onwe ya 3. Ahu siri ya ike,ahu esiya ike nke oma m’obu odighi aga aga | | | | | | | | S6.22  Nrml Med Abnrm DK  1. □ 2. □ 3. □ 9. □  1. □ 2. □ 3. □ 9. □  1. □ 2. □ 3. □ 9. □ | | | | | S6.37  Nrml Med Abnrm DK  1. □ 2. □ 3. □ 9. □  1. □ 2. □ 3. □ 9. □  1. □ 2. □ 3. □ 9. □ |
| *If not taken to another health provider, ask :*  Did you have any concerns or problems that kept you from taking <NAME> to another health provider?  O nwere nsogbu nke gbochiri gi I kporo <AHA> gaa na nke onye oru ahuike ozo?  *If taken to another health provider, ask:*  Did you have to overcome any concerns or problems to take <NAME> to another health provider?  O nwere nsogbu ma o bu ihe mgbochi I gabigara iji nwee ike I kporo <AHA> gaa na nke onye oru ahuike ozo ? | | | | | 1. Yes 2. No   9. Don’t know | | | | | | | | S6.23  🞎 ***2 or 9 → Inst_4*** | | | | | S6.38  🞎 ***2 or 9 → SQ6.39*** |
| What concerns or problems did you have?  Kedu ihe bu ihe mgbochi na nsogbu nke I nwere?  *Prompt:* Was there anything else?  *Prompt:* o nwere ozo?  *[Multiple answers allowed.]* | | | | | 1. Oturu alo na achoghi enyemaka 2. Onweghi onye nonso iso nwada jee 3. Onwere ezigbo oge iji ruo oru ya 4. Onye ozo mere nkowa 5. Njem tere aka 6. Ugboala njem adighi 7. Ego (ugboala,ulo ahuike,na ihe ndi ozo) 8. Anyi enweru afor ojuju na nlekota enyere anyi 9. Nsobua kwesiri ihe odinala 10. Echerem na ahu adighi mma aga ekwe ya mee njem 11. O chere na nwata ga anwu na agbanye nlete anya 12. Obu na etiti abali   Nwata anwuola tupu anyi agawa   1. Ndi ozo *(specify)*   99.Amaghi | | | | | | | | S6.23.1  1. □  2. □  3. □  4. □ _______________  5. □  6. □  7. □  8. □  9. □  10. □  11. □  12. □  13. □ ***→ SQ6.39***  14. □ ______________  99. □ | | | | | S6.38.1  1. □  2. □  3. □  4. □ _______________  5. □  6. □  7. □  8. □  9. □  10. □  11. □  12. □  13. □  14. □ ______________  99. □ |
| ***Inst_4: Check SQ6.5 → If taken to another health provider…*** | | | | | | | | | | | | | ***…go to SQ6.24***  ***(LAST PROVIDER)*** | | | | |  |
| S6.39 | How many days after (first noticing the illness / <LAST ACTION SQ6.5> / leaving the first/last health provider) did <NAME> die?  Mkpuru ubochi ole gachara( a choputatara oria ahu / <ihe ikpazu emere SQ6.5/ hapu ebe nlezi anya nke mbu/nke ikpazu) ka <AHA> nwuru?  *[If SQ6.4 = 2 (No care given), then read: “…first noticing the illness…”]* | | | | | | | | | | | | | | **__ __** Days  *(<1 = 00; DK = 99)* | | | |
| ***Inst_5: If SQ6.4 = 2 (No care given) or***  ***if SQ6.5 ≠ “Health Provider” (Never took and never tried to take to a health provider) → VQ5.10*** | | | | | | | | | | | | | | | | | | |
| **VA Section 5: Health records (FOR STILLBIRTHS, NEONATAL & CHILD DEATHS 0—59 MONTHS OLD)** | | | | | | | | | | | | | | | | | | |
| V5.4 | Do you have any health records that belonged to the deceased?  I nwere akwukwo ahuike ebe e deturu ihe nile gbasara oria nwata a nwuru anwu? | | | | | | 1. Yes 2. No   9. Don’t know | | | | | | | | | 🞎 ***2 or 9 → VQ5.10*** | | |
| V5.5 | Can I see the health records?  E nwere m ike ihu akwukwo ahuike a? | | | | | | 1. Yes 2. No | | | | | | | | | 🞎 ***2 → VQ5.10*** | | |
| V5.6 | *Record the dates of the two most recent visits* | | | | | | | | | | | | | | | **__ __/__ __/__ __ __ __**  D D M M Y Y Y Y  *(DK = 99/99/9999)* | | |
|  |  |  |  |  |  |  |  |  |  |  |  |  |  |  |  | **__ __/__ __/__ __ __ __**  D D M M Y Y Y Y  *(DK = 99/99/9999)* | | |
| V5.7 | *Record the two most recent weights on those dates* | | | | | | | | | | | | | | | **__ __ __ __** Grams  *(DK = 9999)* | | |
|  |  |  |  |  |  |  |  |  |  |  |  |  |  |  |  | **__ __ __ __** Grams  *(DK = 9999)* | | |
| V5.8 | *Record the date of the last note* | | | | | | | | | | | | | | | **__ __/__ __/__ __ __ __**  D D M M Y Y Y Y  *(DK = 99/99/9999)* | | |
| V5.9 | *Transcribe the note* | | | | | | | | | | | | | | | | | |
|  | ________________________________________________________________________________________________  ________________________________________________________________________________________________  ________________________________________________________________________________________________  ________________________________________________________________________________________________  ________________________________________________________________________________________________  ________________________________________________________________________________________________  ________________________________________________________________________________________________ | | | | | | | | | | | | | | | | | |
| V5.10 | Was a death certificate issued?  E nyere ya asambodo onwu? | | | | | | | 1. Yes 2. No   9. Don’t know | | | | | | | | 🞎 ***2 or 9 → SQ1.1*** | | |
| V5.11 | Can I see the death certificate?  E nwere ike ihu asambodo onwu a? | | | | | | | 1. Yes 2. No | | | | | | | | 🞎 ***2 → SQ1.1*** | | |
| V5.12 | *Record the immediate cause of death from the death certificate* | | | | | | |  | | | | | | | | | | |
| V5.13 | *Record the first underlying cause of death from the death certificate* | | | | | | |  | | | | | | | | | | |
| V5.14 | *Record the second underlying cause of death from the death certificate* | | | | | | |  | | | | | | | | | | |
| V5.15 | *Record the third underlying cause of death from the death certificate* | | | | | | |  | | | | | | | | | | |
| V5.16 | *Record the contributing cause of death from the death certificate* | | | | | | |  | | | | | | | | | | |

| **SA Module 1: The mother and her household (FOR STILLBIRTHS, NN & CHILD DEATHS 0—59 MONTHS OLD)**  *Read:* Now I would like to ask you some other questions about (yourself / the child’s mother).  *Read:* Ugbua choro m I jug i ufodu ajuju maka (onwe gi/nne nwata a).  *[Read “…the child’s mother.” If the respondent is not the mother.* | | | | | |
| --- | --- | --- | --- | --- | --- |
| ***Inst_1: If GQ4.3 = 1 (Respondent is the mother) →*** ***SQ1.4*** | | | | | |
| S1.2 | How old (is the child’s mother / was the child’s mother when she died)?  Afo ole ka (nne nwata a di/nne nwata a gbara tupu o nwuo?  *[Read “…was the child’s mother…” if she died.]* | | | __ __ Years  *(DK = 99)* | |
| S1.3 | How many years of school did the mother complete?  Kedu akwukwo kacha elu nne ya gupuru? | | | **__ __** Years  *(<1 = 00; DK = 99)* |  |
| S1.4 | Are you / Is/Was the child’s mother)…  I bu / o gi bu nne nwa ahu)…  *[Read “…Is/Was the child’s mother…” if the respondent is not the mother.]*    *[Read the choices to the respondent.]* | 1. Inuola di ? 2. Gin a nwoke o bi? 3. Di gi onwuola? 4. Ahu agbasala,ekewala? 5. Nwagbogho ,okorobia?   9. Amgahi | | 🞎 ***5 or 9 → Inst_2*** | |
| S1.4.1 | How old (were you when you / was she when she) first married (or lived with a man)?  Afo ole ka(I di mgbe i/o di mgbe o) gara di na mbu(ma obu binyere nwoke)?  *[Read “…was she when she…” if the respondent is not the mother.]*  *[Read “…married or lived with a man?” if SQ1.4 = “2. Living with a man”]* | | | __ __ Years  *(DK = 99)* | |
| S1.4.2 | How many years of school did (your / her) (husband / partner) complete?  Afo akwukwo ole ka (di gi / di ya) ( di / onye otu) guchara ?  *[Read “…her…” if the respondent is not the mother.]*  *[Read “…partner…” if she is living with a man.]* | | | **__ __** Years  *(<1 = 00; DK = 99)* | |
| ***Inst_2:*** *Read:* Now I would like to ask you some questions about (your / the mother’s) household. Please remember that all information will be kept confidential.    *Read:* Ugbua a gam aju gi ufodu ajuju maka ezinulo (gi/nne ya). Biko chetakwa na ihe nile anyi natara gi,agaghi egosi ya onye ozo.    *[SBs & NN deaths: If the respondent is not the mother, read “…the mother’s…;” and ask SQ1.5–1.11 about the mother’s household.*  *Older deaths: Always read “…your…;” and ask SQ1.5–1.11 about the respondent’s household.]* | | | | | |
| S1.5 | Who was the main breadwinner of (your / the mother’s) family during the (last days of the pregnancy / child’s fatal illness)  Kedu onye n,ewetakari ihe ezinulo (gi/nne ya) n,eri n,oge (ime omumu/nwata n,aria oria a gburu ya)  *[SBs~~/~~NN deaths: Read “…last days…”;*  *Older deaths: Read “…child's…”]* | 1. Child’s father 2. Child’s mother 3. Other   9. Don’t know | | 🞎 ***9 → SQ1.7*** | |
| S1.6 | At that time, what kind of work did the main breadwinner mostly do?  Na oge ahu kedu udi oru onye a n,ewetakari ihe ezinulo n,eri na aru kari? | 1. Onye oru ugbo/onye na aruru madu oru ugbo 2. Onye na azu okuko m’obu aturu 3. Onye oru nlekota ulo 4. Onye na aruputa ihe,bi na ulo 5. Onye oru asughi azu 6. Onye oru enyeturu ozuzu/onye oru oru 7. Ndi oru na ulo nmeputa ihe ,onye oru onyibe 8. Onye new oru 9. Onye guru akwukwuo m’obu oru ofuma/Onye muru oru ndozi igwe 10. Ihe ndi ozo *(specify)* 11. Onye oru obodo oyibo   99. Amagahi | | 🞎🞎  ___________________________ | |
| S1.7 | Is this the house (where we are now) where (you / the mother) stayed during the (last days of the pregnancy / child’s fatal illness)?  Ulo a (anyi no ugbua) obu ebe (I/nne nwa a) noro n’ime (oge ikpazu nke afo ime/oria gburu nwa)?  *[SBs/NN deaths: Read “…last days…”*  *Older deaths: Read “…child's…”]*  *[Read “…where we are now…” if needed to clarify which house you are talking about.]* | 1. Yes 2. No   9. Don’t know | | 🞎 ***1 → SQ1.10***  ***9 → VQ5.17*** | |
| S1.8 | Where did (you / the mother) stay at that time?  Kedu ebe (i/nne ya) noro na oge ahu?    *Probe:* Where did (you / the mother) stay during the illness events?  *Probe:* Ebe ka (i/nne nwa) bi na oge nrianria ahu?  *[Mark “1” only if her usual residence was not her in-laws or other relatives.]* | 1. N’ulo nke ya oge ahu na abughi ulo ogo ya oju ajuju were usoro ichoputa ma opugo tupu onwuo*.)* 2. N’ulo ndi ogo ya 3. N’ulo ndi muru ya 4. N,ulo nwa nne ya nwoke 5. Ma ndi ozo*(specify)*   9. Amaghi | | 🞎 ***9 → VQ5.17***  ___________________________ | |
| S1.9 | What is the address of the place where (you / she) stayed?  Kedu ejiri mara a ga eji chota ebe ahu (i/ o) bi oge ahu ? | State ____________________________  LGA _______________________ | | 🞎🞎🞎  🞎🞎🞎 | |
| S1.10 | At the time of the illness events, how long had (you / the mother / your <RELATIVES> / the mother’s <RELATIVES>) been living continuously in (this / that) community?  N,oge a nwata a n,aria oriaa,ogologo oge ole ka (I /nne ya /<umunne>gi / < umunne>nne ya) binyere n’esepughi aka na obodo(a/ahu)?  *[Read “…<RELATIVES…” if SQ1.8 = 2-5 (s/he stayed with her/his relatives)].* | | | __ __ Years  *(<1 = 00; DK =99)* | |
| S1.11 | How long does it take to reach the health provider or facility where (you / the mother) normally (go(es) / went) from (this / that) place?  Ogologo oge ole ka o na ewe iji ruo na nke onye oru ahuike ma obu ulo ahuike ebe (I / nne ya) na-aga (kari) / gara?  *[Mark hours &/or minutes as needed: e.g. 01 hour, 30 minutes]* | | | **__ __** Hours  *(DK = 99)* | |
|  |  |  |  | **__ __** Minutes  *(DK = 99)* | |
| ***Inst_3 → SQ2.1.1 (if including optional Module 2) or VQ5.17*** | | | | | |
| **SA Module 2: Social capital (FOR SBs, NN & CHILD DEATHS 0–59 MONTHS OLD)**  *Read:* Now, I have some questions about (your / the mother’s / your <RELATIVES’> / the mother’s <RELATIVES’>) community.  *Read:* Ugbua enwere m ufodu ajuju gbasara obodo (gi/nne ya/ < umunne> gi/<umunne> nne ya).  *[SBs and NN deaths: If the respondent is not the mother, read “…the mother’s…” or “…the mothers’ <RELATIVES’>...;” and ask SQ2.1.1–SQ2.3.1 about the mother and her community or her relatives’ community.*  *Older deaths: Always read “…your…” or “…your <RELATIVES’>…;” and ask SQ2.1.1–SQ2.3.1 about the respondent and her/his community or her/his relatives’ community.*  *All deaths: Ask about the relatives’ community if s/he stayed with her/his relatives during the illness events.]* | | | | | |
| S2.1.1 | In the last 3 years, did the people in the (village / neighborhood) work together on any of the following issues that affect the entire community or part of the community?  N,ime Afo ato gara aga,o dila mgbe ndi madu bi (n,ime obodo/agbata obi a) jikoro aka onu were ruo oru maka ihe ndi metutara oha obodo ma-obu otu akuku obodo?  *Read all the issues and mark (“X”) Yes, No or DK for each one; then enter the code.]* | | 1. Agumagu/ulo akwukwo 2. Ahukie/Ulo ahuike 3. Ohere inweta oru ana akwu ugwo 4. Igbazinye Ego 5. Uzo 6. Iga njem 7. Ike mmiri 8. Mkpochi na nzacha 9. Oru ugbo 10. Ikpe nkwumoto/ ikpezi esemokwu 11. Nchekwa/Enyemaka ndi uwe ojii 12. Ulo uka/ikpo isi na ala 13. Ihe ndi ozo   *(specify)* | Yes No DK  1. □ 2. □ 9. □  1. □ 2. □ 9. □  1. □ 2. □ 9. □  1. □ 2. □ 9. □  1. □ 2. □ 9. □  1. □ 2. □ 9. □  1. □ 2. □ 9. □  1. □ 2. □ 9. □  1. □ 2. □ 9. □  1. □ 2. □ 9. □  1. □ 2. □ 9. □  1. □ 2. □ 9. □  1. □ 2. □ 9. □  ___________________________ | |
|  |  |  | *Code:*   1. One or more issues identified 2. No issue identified | 🞎 | |
| S2.2 | (Were you / Was the mother) able to turn to any persons, groups or organizations in the community for help during (the pregnancy / (or) the child’s fatal illness)?  (i/nne ya) e nwere ike gakwuru ndi madu,ma obu ogbako obula na obodo maka enyemaka na oge (o di ime/ma o bu) nwata a n,aria oria a gburu)?  *[Read “…the pregnancy?” for SBs; or “…the pregnancy or the child’s fatal illness?” for NN deaths; or “…the child’s fatal illness for older deaths.]* | | 1. Yes 2. No   9. Don’t know | 🞎 ***2 or 9 → SQ2.3.1*** | |
| S2.2.1 | Did (you / she) turn to any of the following for help?  (i /o ) gakwuru onye/otu obula dika ndia maka enye aka ?  *[Read all the options and mark (“X”) Yes, No or DK for each; then enter the code.]* | | 1. Ezinaulo 2. Ndi agabtaobi 3. Enyi 4. Ndi isi ndi Uka m’obu otu 5. Ndi isi ndi obodo 6. Iwe ojii 7. Nna ndumodu onye oru na onye nwetere ihe 8. Onyi isi ochichi 9. Nkwekota/nkwagide o no na ya 10. Otu enyemaka nke onoghi na ya 11. Other   *(specify)* | Yes No DK  1. □ 2. □ 9. □  1. □ 2. □ 9. □  1. □ 2. □ 9. □  1. □ 2. □ 9. □  1. □ 2. □ 9. □  1. □ 2. □ 9. □  1. □ 2. □ 9. □  1. □ 2. □ 9. □  1. □ 2. □ 9. □  1. □ 2. □ 9. □  1. □ 2. □ 9. □  1. □ 2. □ 9. □  ___________________________ | |
|  |  |  | *Code:*   1. One person/group identified 2. Two or more persons/groups identified 3. No person/group identified | 🞎***3 → SQ2.3.1*** | |
| S2.2.2 | (Is this / Are these) the same person(s) or group(s) (you / she) would usually turn to for help with a serious problem?  (Ndi a/Ndi ahu) a bu otu ndi a (i/o) na agakwuru mgbe obula I choro enyemaka gbasara nsogbu ndi siri ike? | | 1. Yes 2. No   9. Don’t know | 🞎 | |
[truncated: 164,950 more chars]
