# Supplementary material for: Direct estimates of cause-specific mortality fractions and rates of under-five deaths in the northern and southern regions of Nigeria by verbal autopsy interview
Source: PLoS One. 2017 May 31;12(5):e0178129. doi: 10.1371/journal.pone.0178129 (PMC5451023; doi:10.1371/journal.pone.0178129)
Supplement: S2 Appendix — (DOCX) [file pone.0178129.s002.docx]

**Adewemimo A, et al. Direct estimates of cause-specific mortality fractions and rates of under-five deaths in the northern and southern regions of Nigeria by verbal autopsy interview.**

**Appendix 2: Age and sex distributions and mortality rates of neonatal and 1-59 month old deaths in Nigeria with the geographic zones re-distributed by under-five mortality levels**

**Comparing the three highest (3HM) to the three lowest (3LM) under-five mortality zones**

**Age distributions**

As seen in table 1, re-categorizing the six geographical zones according to their under-five mortality levels, by grouping the relatively high mortality South East zone with the highest mortality North West and North East (three highest mortality=3HM), and the relatively low mortality North Central zone with the lowest mortality South South and South West (three lowest mortality=3LM), resulted in a mild increase (over the South-North proportion described in the main paper) in the proportion of neonatal deaths in the lowest compared to the highest mortality area (3HM=472/2,071=22.8% vs. 3LM=251/709=35.4%, X^2^=27.9, p<0.001); and both neonates (X^2^=3.0, p=0.082) and 1-59 month olds (X^2^=26.2, p<0.001) in the lowest mortality area died at younger ages than in the highest mortality area, though, as in the South, still not significantly so for neonates.

**Sex distributions**

As shown in table 1, the re-categorization also did not modify the findings for sex. There were still significantly more deaths of male than female neonates in the three highest (57.1% vs. 42.9%, X^2^=7.2, p=0.007) mortality zones and nearly so in the three lowest (56.7% vs. 43.3%, X^2^=3.6, p=0.059) mortality zones. And there were still no differences in the percent of deaths of male and female post-neonates, neither in the three highest nor three lowest mortality zones nor in Nigeria as a whole.

**Table 1. Demographic characteristics of deceased neonates and children in Nigeria’s three highest (North West, North East, South East) and three lowest (North Central, South South, South West) under-five mortality zones.**

| **Characteristic** | **3HM:**  **Three highest mortality zones**  **N (%)** | **3LM:**  **Three lowest mortality zones**  **N (%)** | **Total**  **N (%)** | **X^2^, p-value*** |
| --- | --- | --- | --- | --- |
| Neonates |  |  |  |  |
| Age (days) |  |  |  |  |
| 0-6 | 336 (71.2) | 198 (78.9) | 534 (73.9) | 3.0, 0.082 |
| 7-27 | 136 (28.8) | 53 (21.1) | 189 (26.1) |  |
| Sex**^±^** |  |  |  |  |
| Male | 269 (57.1) | 142 (56.7) | 411 (56.9) | 0.0, 0.934 |
| Female | 202 (42.9) | 109 (43.3) | 311 (43.1) |  |
| Total neonates | 472 (65.2) | 251 (34.8) | 723 (100.0) | 27.9, <0.001 |
| 1-59 month olds |  |  |  |  |
| Age (months) |  |  |  |  |
| 1-5 | 218 (13.7) | 108 (23.7) | 326 (15.8) | 26.2, <0.001 |
| 6-11 | 284 (17.7) | 87 (18.9) | 371 (18.1) |  |
| 12-23 | 440 (27.5) | 129 (28.1) | 569 (27.6) |  |
| 24-59 | 657 (41.1) | 134 (29.3) | 791 (38.5) |  |
| Sex**^±^** |  |  |  |  |
| Male | 813 (50.9) | 238 (51.9) | 1051 (51.1) | 0.1, 0.711 |
| Female | 785 (49.1) | 220 (48.1) | 1005 (48.9) |  |
| Total 1-59 months | 1599 (77.7) | 458 (22.3) | 2057 (100.0) |  |
| Total deaths | 2,071 (100.0) | 709 (100.0) | 2,780 (100.0) |  |

*All statistical tests are of 3HM-3LM differences. The test of ‘Total neonates’ is for the proportion of under-five deaths that were neonatal; ^±^1 with missing data in the 3HM region.

**Mortality rates**

Table 2 displays age-specific mortality rates for neonates and 1-59 month olds in the 3HM and 3LM zones. The trend toward an increasing 3HM/3LM hazard ratio with increasing age was more exaggerated than in the North-South regional division, with the ratio of the NNMR/159MR ratios equal to 0.5 (1.1/2.2) compared to the North-South’s 0.6 (1.1/1.8). Hence, the pattern observed in the North-South differences in age-specific proportions of deaths being due to similar neonatal mortality in the 3HM and 3LM areas but significantly higher child mortality in the 3HM area was maintained. Deviation from this pattern by the early and late neonatal deaths corresponded to exchange of the South East zone, with relatively high NNMR, and the North Central zone, with lower NNMR, between the areas, as did the shift of birth injury/asphyxia deaths from the North to the 3HM area as seen below in Appendix 2. However, the differences between the North/South and 3HM/3LM hazard ratios for early and late neonatal deaths were small and might be due at least in part to the low late neonatal mortality rates and consequent chance associations.

**Table 2. Age-specific neonatal and child mortality rates* in Nigeria’s three highest (North West, North East, South East) and three lowest (North Central, South South, South West) under-five mortality zones.**

| **Age** | **3HM:**  **Three highest mortality zones**  **Estimate (95% CI)** | **3LM:**  **Three lowest mortality zones**  **Estimate (95% CI)** | **Total**  **Estimate (95% CI)** | **Ratio**  **3HMR / 3LMR**  **Est (95% CI)** | **Z, p-value^±^** |
| --- | --- | --- | --- | --- | --- |
| Neonates (days) |  |  |  |  |  |
| 0-6 | 33.4 (31.0, 35.9) | 28.3 (25.7, 31.0) | 31.6 (29.8, 33.5) | 1.2 (1.1, 1.3) | 2.8, 0.005 |
| 7-27 | 5.9 (2.8, 9.1) | 6.4 (2.5, 10.3) | 6.0 (3.5, 8.5) | 0.9 (0.4, 2.3) | -0.2, 0.873 |
| Total neonates^β^ | 39.2 (35.3, 43.0) | 34.5 (29.7, 39.4) | 37.5 (34.3, 40.5) | 1.1 (1.0, 1.3) | 1.5, 0.141 |
| 1-59 month olds (months) |  |  |  |  |  |
| 1-5 | 17.0 (14.8, 19.2) | 13.0 (10.4, 15.7) | 15.6 (13.8, 17.3) | 1.3 (1.0, 1.7) | 2.2, 0.031 |
| 6-11 | 20.7 (17.9, 23.5) | 10.2 (7.8, 12.6) | 16.9 (14.9, 18.9) | 2.0 (1.5, 2.7) | 5.1, < 0.001 |
| 12-23 | 33.7 (30.1, 37.2) | 14.9 (11.8, 18.0) | 26.8 (24.1, 29.4) | 2.3 (1.8, 2.9) | 6.8, < 0.001 |
| 24-59 | 49.7 (44.1, 55.2) | 14.6 (11.7, 17.6) | 36.9 (32.9, 40.8) | 3.4 (2.7, 4.3) | 10.1, < 0.001 |
| Total 1-59 month olds^β^ | 116.0 (107.3, 124.7) | 51.7 (46.0, 57.4) | 92.9 (86.5, 99.2) | 2.2 (2.0, 2.6) | 11.9, < 0.001 |

*All mortality rates are calculated from the NDHS birth history data for the same prior 5-year period as the VASA deaths**; ^±^**Equivalence tested with an observed Z statistic, with a normal approximation using the bootstrap of survey primary sampling units [33]; ^β^Total neonatal mortality rate per 1,000 live births; Total 1-59 month mortality rate, calculated as: 1-((1-U5MR)/(1-NMR)); Each age-specific rate estimated directly from the cohort that survived to that age.

**Comparing the two highest (NWNE) to the four lowest (4LM) under-five mortality zones**

**Age distributions**

As is evident in table 3, moving the South East zone back to the low mortality group, so as to compare the highest mortality North West and North East zones (NWNE) to the four lowest mortality zones (4LM), also minimally altered the relative age distributions of the deaths. Neonatal deaths still contributed more to under-five mortality in the low mortality zones (NWNE=416/1,829=22.8% vs. 4LM=307/951=32.3%, X^2^=19.5, p<0.001); and both neonates (X^2^=2.9, p=0.087) and 1-59 month olds (X^2^=52.8, p<0.001) in the lowest mortality area died at younger ages than in the highest mortality area, though, as in the South, still not significantly so for neonates.

**Sex distributions**

Also as seen in table 3, re-categorizing the zones in this way also did not modify the findings for sex. There were still significantly more deaths of male than female neonates, both in the two highest (57.3% vs. 42.7%, X^2^=6.7, p=0.010) and four lowest (56.5% vs. 43.5%, X^2^=4.2, p=0.041) mortality zones. And there were still no differences in the percent of deaths of male and female post-neonates, neither in the highest nor lowest mortality zones nor in the country as a whole.

**Table 3. Demographic characteristics of deceased neonates and children in Nigeria’s two highest (North West, North East) and four lowest (North Central, South East, South South, South West) under-five mortality zones.**

| **Characteristic** | **NWNE:**  **Two highest mortality zones**  **N (%)** | **4LM:**  **Four lowest mortality zones**  **N (%)** | **Total**  **N (%)** | **X^2^, p-value*** |
| --- | --- | --- | --- | --- |
| Neonates |  |  |  |  |
| Age (days) |  |  |  |  |
| 0-6 | 295 (70.9) | 239 (78.0) | 534 (73.9) | 2.9, 0.087 |
| 7-27 | 121 (29.1) | 68 (22.0) | 189 (26.1) |  |
| Sex**^±^** |  |  |  |  |
| Male | 238 (57.3) | 173 (56.5) | 411 (56.9) | 0.0, 0.847 |
| Female | 177 (42.7) | 134 (43.5) | 311 (43.1) |  |
| Total neonates | 416 (57.5) | 307 (42.5) | 723 (100.0) | 19.5, <0.001 |
| 1-59 month olds |  |  |  |  |
| Age (months) |  |  |  |  |
| 1-5 | 177 (12.5) | 150 (23.3) | 327 (15.9) | 52.8, <0.001 |
| 6-11 | 230 (16.3) | 140 (21.8) | 370 (18.0) |  |
| 12-23 | 389 (27.5) | 180 (27.9) | 569 (27.6) |  |
| 24-59 | 617 (43.7) | 174 (27.0) | 791 (38.5) |  |
| Sex**^±^** |  |  |  |  |
| Male | 721 (51.0) | 330 (51.2) | 1051 (51.1) | 0.0, 0.943 |
| Female | 691 (49.0) | 314 (48.8) | 1005 (48.9) |  |
| Total 1-59 months | 1,413 (68.7) | 644 (31.3) | 2057 (100.0) |  |
| Total deaths | 1,829 (100.0) | 951 (100.0) | 2,780 (100.0) |  |

*All statistical tests are of NWNE-4LM differences. The test of ‘Total neonates’ is for the proportion of under-five deaths that were neonatal; ^±^1 with missing data in the NWNE region.

**Mortality rates**

Table 4 displays age-specific mortality rates for neonates and 1-59 month olds in the NWNE and 4LM zones. The tendency for the NWNE/4LM hazard ratio to increase with increasing age was similar to that for the 3HM/3LM areas and North/South regions. And once again the pattern of similar neonatal mortality but significantly higher child mortality in the high mortality NWNE area led to the area-wide differences in age-specific proportions of deaths. The deviation from this pattern by the early and late neonatal deaths diminished from that seen in the 3HM-3LM areas, corresponding to movement of the South East, with its mid-level NNMR, to the 4LM area. As before, the differences between the areas’ hazard ratios for early and late neonatal deaths were small and might be due at least in part to the low late neonatal mortality rates and consequent chance associations.

**Table 4. Age-specific neonatal and child mortality rates* in Nigeria’s two highest (North West, North East) and four lowest (North Central, South East, South South, South West) under-five mortality zones.**

| **Age** | **NWNE:**  **Two highest mortality zones**  **Estimate (95% CI)** | **4LM:**  **Four lowest mortality zones**  **Estimate (95% CI)** | **Total**  **Estimate (95% CI)** | **Ratio**  **NWNEMR / 4LMR**  **Est (95% CI)** | **Z, p-value^±^** |
| --- | --- | --- | --- | --- | --- |
| Neonates (days) |  |  |  |  |  |
| 0-6 | 33.7 (31.0, 36.5) | 29.0 (26.6, 31.4) | 31.6 (29.8, 33.5) | 1.2 (1.0, 1.3) | 2.5, 0.011 |
| 7-27 | 6.0 (2.7, 9.4) | 6.1 (2.5, 9.7) | 6.0 (3.5, 8.5) | 1.0 (0.4, 2.4) | -0.1, 0.978 |
| Total neonates^β^ | 39.6 (35.3, 43.8) | 35.0 (30.8, 39.1) | 37.5 (34.3, 40.5) | 1.1 (0.9, 1.3) | 1.5, 0.124 |
| 1-59 month olds (months) |  |  |  |  |  |
| 1-5 | 16.3 (14.0, 18.7) | 14.7 (12.8, 17.3) | 15.6 (13.8, 17.3) | 1.1 (0.9, 1.4) | 0.9, 0.362 |
| 6-11 | 20.6 (17.6, 23.6) | 12.4 (9.9, 14.8) | 16.9 (14.9, 18.9) | 1.7 (1.3, 2.1) | 3.3, 0.001 |
| 12-23 | 34.8 (30.9, 38.8) | 17.0 (14.1, 19.9) | 26.8 (24.1, 29.4) | 2.0 (1.7, 2.5) | 6.9, < 0.001 |
| 24-59 | 53.9 (47.8, 60.0) | 15.9 (13.0, 18.7) | 36.9 (32.9, 40.8) | 3.4 (2.7, 4.2) | 11.1, < 0.001 |
| Total 1-59 month olds^β^ | 120.3 (110.8, 129.8) | 58.6 (52.8, 64.4) | 92.9 (86.5, 99.2) | 2.1 (1.8, 2.3) | 11.2, < 0.001 |

*All mortality rates are calculated from the NDHS birth history data for the same prior 5-year period as the VASA deaths**; ^±^**Equivalence tested with an observed Z statistic, with a normal approximation using the bootstrap of survey primary sampling units [33]; ^β^Total neonatal mortality rate per 1,000 live births; Total 1-59 month mortality rate, calculated as: 1-((1-U5MR)/(1-NMR)); Each age-specific rate estimated directly from the cohort that survived to that age.
