# Supplementary material for: Direct estimates of cause-specific mortality fractions and rates of under-five deaths in the northern and southern regions of Nigeria by verbal autopsy interview
Source: PLoS One. 2017 May 31;12(5):e0178129. doi: 10.1371/journal.pone.0178129 (PMC5451023; doi:10.1371/journal.pone.0178129)
Supplement: S3 Appendix — (DOCX) [file pone.0178129.s003.docx]

**Adewemimo A, et al. Direct estimates of cause-specific mortality fractions and rates of under-five deaths in the northern and southern regions of Nigeria by verbal autopsy interview.**

**Appendix 3: Expert algorithm verbal autopsy causes of neonatal and 1-59 month old deaths in the North and South of Nigeria and with the geographic zones re-distributed by under-five mortality levels**

**Comparing the causes of neonatal deaths in the North and South of Nigeria, and with the geographic zones redistributed by under-five mortality levels**

Re-categorizing the six geographic zones according to their under-five mortality levels yielded a similar ‘North’-‘South’ mortality pattern, but with some adjustments. Grouping the relatively high under-five mortality South East zone with the highest mortality North West and North East (three highest mortality=3HM), and the relatively lower mortality North Central zone with the lowest mortality South South and South West (three lowest mortality=3LM), neutralized the North–South birth injury/asphyxia mortality differential (3HM=22.0% vs. 3LM=22.7%, X^2^=0.03, p=0.867), but had minimal effect on preterm delivery (3HM=1.1% vs. 3LM=3.5%, X^2^=4.41, p=0.036). Moving the South East zone back to the lowest mortality group, in order to compare the highest mortality North West and North East zones (NWNE) to the four lowest mortality zones (4LM), returned the birth injury/asphyxia differential to close to its original level (N=20.3% vs. S=24.9%, X^2^=1.49, p=0.223), while lessening the disparity in deaths due to preterm delivery (NWNE=1.2% vs. 4LM=2.9%, X^2^=2.21, p=0.137).

**Table 1. Expert algorithm verbal autopsy causes of neonatal death in northern and southern Nigeria.**

|  | **North (N=511)** | **South (N=212)** |  |
| --- | --- | --- | --- |
| **Cause of death** | **% (CLs)** | **% (CLs)** | **Chi-square, p-value** |
| NNT | 1.3 (0.3-2.2) | 1.1 (0.0-2.5) | 0.0, 0.901 |
| Malformation | 0.8 (0.00-2.0) | 1.2 (0.0-2.8) | 0.1, 0.731 |
| Birth injury/asphyxia | 20.6 (16.3, 24.9) | 26.2 (18.9, 33.6) | 1.8, 0.179 |
| Meningitis | 1.3 (0.0, 2.6) | 1.0 (0.0, 2.9) | 0.1, 0.815 |
| Diarrhea | 3.3 (1.5, 5.0) | 2.0 (0.1, 3.9) | 0.8, 0.360 |
| Pneumonia | 20.2 (16.4, 23.9) | 19.1 (13.4, 24.9) | 0.1, 0.772 |
| Sepsis | 31.7 (27.1, 36.4) | 31.1 (23.2, 38.9) | 0.0, 0.891 |
| Other | 5.6 (3.6, 7.5) | 2.8 (0.4, 5.2) | 2.2, 0.140 |
| Preterm | 1.2 (0.3, 2.1) | 3.7 (0.5, 7.0) | 4.1, 0.042 |
| Unspecified | 14.2 (10.2, 18.1) | 11.7 (7.1, 16.3) | 0.6, 0.433 |
| Total | 100 | 100 |  |

**Table 2. Expert algorithm verbal autopsy causes of neonatal death in Nigeria’s three highest (North West, North East, South East) and three lowest (North Central, South South, South West) under-five mortality zones.**

|  | **3HM: Three highest mortality zones (N=472)** | **3LM: Three lowest mortality zones (N=251)** |  |
| --- | --- | --- | --- |
| **Cause of death** | **% (CLs)** | **% (CLs)** | **Chi-square, p-value** |
| NNT | 1.4 (0.4, 2.5) | 0.8 (0.0, 1.9) | 0.6, 0.438 |
| Malformation | 1.0 (0.0, 2.3) | 0.7 (0.0, 1.9) | 0.1, 0.752 |
| Birth injury/asphyxia | 22.0 (17.4, 26.7) | 22.7 (16.4, 28.9) | 0.0, 0.867 |
| Meningitis | 1.2 (0.0, 2.6) | 1.2 (0.0, 2.9) | 0.0, 0.974 |
| Diarrhea | 3.1 (1.5, 4.7) | 2.5 (0.2, 4.8) | 0.2, 0.685 |
| Pneumonia | 21.2 (17.3, 25.0) | 17.4 (12.0, 22.8) | 1.2, 0.273 |
| Sepsis | 29.8 (25.1, 34.5) | 34.7 (27.5, 42.0) | 1.3, 0.251 |
| Other | 5.8 (3.7, 8.0) | 2.8 (0.7, 4.9) | 3.2, 0.075 |
| Preterm | 1.1 (0.2, 2.0) | 3.5 (0.7, 6.4) | 4.4, 0.036 |
| Unspecified | 13.3 (9.0, 17.6) | 13.7 (9.6, 17.7) | 0.0, 0.903 |
| Total | 100 | 100 |  |

**Table 3. Expert algorithm verbal autopsy causes of neonatal death in Nigeria’s two highest (North West, North East) and four lowest (North Central, South East, South South, South West) under-five mortality zones.**

|  | **NWNE: Two highest mortality zones (N=416)** | **4LM: Four lowest mortality zones (N=307)** |  |
| --- | --- | --- | --- |
| **Cause of death** | **% (CLs)** | **% (CLs)** | **Chi-square, p-value** |
| NNT | 1.5 (0.3, 2.7) | 0.8 (0.0, 1.7) | 0.9, 0.337 |
| Malformation | 0.9 (0.0, 2.3) | 0.9 (0.0, 2.1) | 0.0, 0.998 |
| Birth injury/asphyxia | 20.3 (15.6, 25.0) | 24.9 (19.0, 30.9) | 1.5, 0.223 |
| Meningitis | 1.4 (0.0, 2.9) | 1.0 (0.0, 2.4) | 0.1, 0.700 |
| Diarrhea | 3.1 (1.4, 4.9) | 2.6 (0.5, 4.6) | 0.1, 0.702 |
| Pneumonia | 20.2 (16.2, 24.2) | 19.4 (14.4, 24.5) | 0.1, 0.820 |
| Sepsis | 30.9 (25.7, 36.0) | 32.4 (26.1, 38.7) | 0.1, 0.716 |
| Other | 6.3 (3.9, 8.7) | 2.6 (0.8, 4.5) | 5.2, 0.023 |
| Preterm | 1.2 (0.2, 2.3) | 2.9 (0.5, 5.2) | 2.2, 0.137 |
| Unspecified | 14.2 (9.5, 18.8) | 12.5 (8.9, 16.1) | 0.3, 0.569 |
| Total | 100 | 100 |  |

**Comparing the causes of 1-59 month deaths in the North and South of Nigeria, and with the geographic zones redistributed by under-five mortality levels**

Similar to the finding for birth injury/asphyxia among the neonatal deaths, combining the anomalously high under-five mortality South East zone with the highest mortality North West and North East zones (3HM) and the lower mortality North Central zone with the lowest mortality South South and South West zones (3LM), decreased the variation in the North-South cause-specific mortality proportions for all causes, leaving watery diarrhea as the only cause with a significant difference between the two areas (3HM=24.1% vs. 3LM=16.1%, X^2^=10.3, p=0.001), and the pneumonia differential neutralized (3HM=16.1% vs. 3LM=17.7%, X^2^=0.5, p=0.499). In addition, the near-significant excess of injury deaths in the North was reversed (3HM=2.5% vs. 3LM=3.9%, X^2^=2.1, p=0.151). Moving the South East zone back to the lowest mortality group, in order to compare the highest mortality North West and North East (NWNE) to all other zones combined (4LM), reinstated all the ‘North’-‘South’ cause of death differentials to their original levels observed in the strictly geographic division of the zones, with the exception of injury deaths, which remained neutralized (NWNE=2.6% vs. 4LM=3.2%, X^2^=0.4, p=0.508).

**Table 4. Expert algorithm verbal autopsy causes of 1-59 month death in northern and southern Nigeria.**

|  | **North (N=1616)** | **South (N=441)** |  |
| --- | --- | --- | --- |
| **Cause of death** | **% (CLs)** | **% (CLs)** | **Chi-square, p-value** |
| Injury | 3.1 (2.2, 4.1) | 1.7 (0.5, 2.8) | 2.9, 0.090 |
| AIDS | 0.8 (0.3, 1.2) | 0.2 (0.0, 0.6) | 2.0, 0.159 |
| Malnutrition | 0.4 (0.2, 0.7) | 1.2 (0.0, 2.4) | 2.6, 0.109 |
| Measles | 2.6 (1.7, 3.4) | 0.2 (0.0, 0.5) | 14.5, <0.001 |
| Meningitis | 6.4 (5.1, 7.8) | 2.8 (0.7, 4.9) | 4.9, 0.026 |
| Dysentery | 3.9 (2.9, 4.9) | 3.2 (1.4, 5.0) | 0.4, 0.514 |
| Diarrhea | 24.8 (22.3, 27.3) | 13.2 (9.6, 16.8) | 21.6, <0.001 |
| Pertussis | 0.6 (0.2, 1.0) | 0.5 (0.0, 1.2) | 0.1, 0.819 |
| Pneumonia | 15.0 (13.0, 17.1) | 21.6 (17.2, 26.0) | 8.2, 0.004 |
| Malaria | 34.7 (31.9, 37.5) | 42.2 (37.2, 47.2) | 6.8, 0.009 |
| Other | 2.8 (1.9, 3.7) | 3.8 (2.0, 5.7) | 1.3, 0.253 |
| Unspecified | 4.9 (3.7, 6.0) | 9.6 (6.6, 12.6) | 11.6, <0.001 |
| Total | 100 | 100 |  |

**Table 5. Expert algorithm verbal autopsy causes of 1-59 month death in Nigeria’s three highest (North West, North East, South East) and three lowest (North Central, South South, South West) under-five mortality zones.**

|  | **3HM: Three highest mortality zones (N=1600)** | **3LM: Three lowest mortality zones (N=457)** |  |
| --- | --- | --- | --- |
| **Cause of death** | **% (CLs)** | **% (CLs)** | **Chi-square, p-value** |
| Injury | 2.5 (1.7, 3.3) | 3.9 (1.8, 6.0) | 2.1, 0.151 |
| AIDS | 0.8 (0.4, 1.2) | 0.1 (0.0, 0.4) | 3.9, 0.047 |
| Malnutrition | 0.5 (0.2, 0.7) | 1.1 (0.0, 2.3) | 2.3, 0.128 |
| Measles | 2.3 (1.5, 3.2) | 1.1 (0.0, 2.1) | 2.2, 0.140 |
| Meningitis | 6.1 (4.8, 7.6) | 3.9 (1.9, 5.9) | 2.7, 0.101 |
| Dysentery | 4.1 (3.0, 5.1) | 2.6 (1.1, 4.2) | 1.8, 0.183 |
| Diarrhea | 24.1 (21.5, 26.6) | 16.1 (12.4, 19.9) | 10.3, 0.001 |
| Pertussis | 0.6 (0.2, 1.1) | 0.3 (0.0, 0.9) | 0.5, 0.502 |
| Pneumonia | 16.1 (14.0, 18.1) | 17.7 (13.3, 22.1) | 0.5, 0.499 |
| Malaria | 35.3 (32.5, 38.2) | 39.8 (34.7, 44.9) | 2.3, 0.130 |
| Other | 3.2 (2.2, 4.2) | 2.2 (1.0, 3.4) | 1.4, 0.236 |
| Unspecified | 4.4 (3.2, 5.6) | 11.1 (8.0, 14.2) | 23.4, <0.001 |
| Total | 100 | 100 |  |

**Table 6. Expert algorithm verbal autopsy causes of 1-59 month death in Nigeria’s two highest (North West, North East) and four lowest (North Central, South East, South South, South West) under-five mortality zones.**

|  | **NWNE: Two highest mortality zones (N=1414)** | **4LM: Four lowest mortality zones (N=643)** |  |
| --- | --- | --- | --- |
| **Cause of death** | **% (CLs)** | **% (CLs)** | **Chi-square, p-value** |
| Injury | 2.6 (1.8, 3.5) | 3.2 (1.7, 4.8) | 0.4, 0.508 |
| AIDS | 0.8 (0.4, 1.3) | 0.2 (0.0, 0.6) | 3.2, 0.074 |
| Malnutrition | 0.5 (0.2, 0.8) | 0.8 (0.0, 1.7) | 0.6, 0.458 |
| Measles | 2.6 (1.7, 3.6) | 0.8 (0.0, 1.5) | 6.0, 0.014 |
| Meningitis | 6.6 (5.1, 8.1) | 3.7 (1.9, 5.4) | 5.0, 0.025 |
| Dysentery | 4.0 (2.9, 5.1) | 3.1 (1.7, 4.6) | 0.9, 0.343 |
| Diarrhea | 26.0 (23.3, 28.8) | 14.1 (11.2, 17.1) | 29.8, <0.001 |
| Pertussis | 0.7 (0.2, 1.7) | 0.3 (0.0, 0.8) | 0.8, 0.369 |
| Pneumonia | 14.7 (12.6, 16.8) | 20.2 (16.4, 24.0) | 7.0, 0.008 |
| Malaria | 34.1 (31.1, 37.1) | 41.2 (37.1, 45.4) | 7.7, 0.006 |
| Other | 3.0 (2.0, 4.0) | 3.0 (1.7, 4.3) | 0.0, 0.987 |
| Unspecified | 4.3 (3.1, 5.5) | 9.3 (6.9, 11.8) | 16.4, <0.001 |
| Total | 100 | 100 |  |
